# Formation of mono- and dual-labelled antibody fragment conjugates *via* reversible site-selective disulfide modification and proximity induced lysine reactivity†

**DOI:** 10.1039/d4sc06500j

**Published:** 2025-01-06

**Authors:** Ioanna A. Thanasi, Nathalie Bouloc, Clíona McMahon, Ning Wang, Peter A. Szijj, Tobias Butcher, Léa N. C. Rochet, Elizabeth A. Love, Andy Merritt, James R. Baker, Vijay Chudasama

**Affiliations:** a Department of Chemistry, University College London 20 Gordon Street London WC1H 0AJ UK v.chudasama@ucl.ac.uk j.r.baker@ucl.ac.uk; b LifeArc, Accelerator Building Open Innovation Campus Stevenage SG1 2FX UK

## Abstract

Many protein bioconjugation strategies focus on the modification of lysine residues owing to the nucleophilicity of their amine side-chain, the generally high abundance of lysine residues on a protein's surface and the ability to form robustly stable amide-based bioconjugates. However, the plethora of solvent accessible lysine residues, which often have similar reactivity, is a key inherent issue when searching for regioselectivity and/or controlled loading of an entity. A relevant example is the modification of antibodies and/or antibody fragments, whose conjugates offer potential for a wide variety of applications. Thus, research in this area for the controlled loading of an entity *via* reaction with lysine residues is of high importance. In this article, we present an approach to achieve this by exploiting the quantitative and reversible site-selective modification of disulfides using pyridazinediones, which facilitates near-quantitative proximity-induced reactions with lysines to enable controlled loading of an entity. The strategy was appraised on several clinically relevant antibody fragments and enabled the formation of mono-labelled lysine-modified antibody fragment conjugates *via* the formation of stable amide bonds and the use of click chemistry for modular modification. Furthermore, through the use of multiple cycles of this novel strategy, an orthogonally bis-labelled lysine-modified antibody fragment conjugate was also furnished.

## Abstract 2

Exploiting quantitative and reversible site-selective disulfide modification as a means for selective lysine functionalisation on clinically relevant antibody fragments.

## Graphical abstract

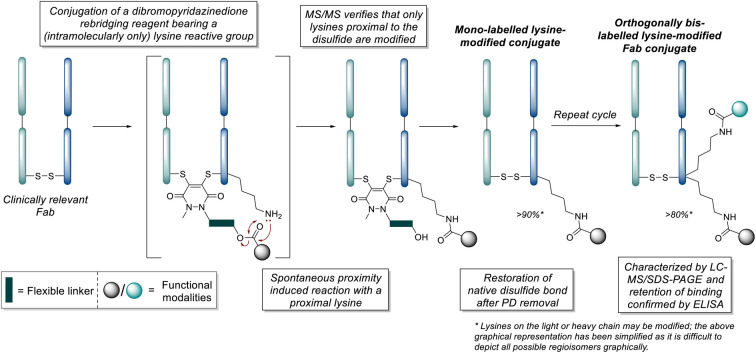

## Introduction

Over the past few decades, there has been growing interest in the field of protein modification owing to the opportunities it enables in therapeutics, diagnostics, imaging, *etc.*^1–5^ Consequently, a plethora of protein modification strategies have been reported in the literature as valuable tools that have found use in multiple applications.^6–9^ Notably, and most directly related to this work, antibody and antibody fragment modification/bioconjugation has enabled crucial breakthroughs in cancer therapeutic treatments such as antibody–drug conjugates (ADCs),^10–13^ diagnostic tools for biomarker detection,^14^ imaging of tumours (*e.g.*, positron emission tomography (PET) techniques),^15–18^ drug delivery (*e.g.*, antibody-conjugated nanoparticles),^19^*etc.* As substantiated by prior studies in the field, site-selective antibody modification is key to attaining antibody conjugates with predictable and optimal pharmacokinetic/*in vivo* profiles.^20–23^ It also minimises issues associated with having antibody conjugate mixtures where there is a wide distribution of loadings (*e.g.*, batch-to-batch variability, no- or low-loaded conjugates limiting efficiency, high-loaded conjugates being ineffective due to them being rapidly cleared from the body, *etc.*).^11,24^

Several methodologies have been developed to accomplish site-selective antibody modification, including genetic engineering for the incorporation of unnatural amino acids, cysteine mutants^24–26^ or through the use of enzymatically activatable tags.^27^ Undoubtedly, these strategies have paved the way for a high degree of site-selective modification of antibodies. However, none of them employ a native antibody scaffold and are thus limited by the generally lower protein expression yields and higher costs/extended periods of time required to produce the desired antibody mutants. Other limitations also include (in some cases) the greater potential for protein aggregation and unfavourable/undesired disulfide scrambling (especially when using cysteine mutants).^28,29^

An interesting alternative is the modification of native antibodies, which is a rapidly growing area of interest due to its advantages including generally low cost, better scalability and better applicability of conditions to multiple proteins without the need for bespoke optimisation. Several groups have reported using reagents for the site-selective modification of the native interstrand disulfide bond(s) on an antibody (or relevant antibody fragment) by reduction followed by reaction with electrophiles bearing one or two electrophilic centres.^30–48^ Crucially, the antibody's structural integrity can remain intact when using certain disulfide rebridging reagents such as certain PD reagents under certain reaction conditions that obviate disulfide scrambling issues.^49,50^ However, when modifying individual cysteine residues liberated from disulfide reduction with electrophiles bearing only one electrophilic centre,^51^ this leads to a loss of a covalent link between the antibody chains and can result in *in vivo* instability.^52^ The modification of native antibodies *via* the primary amino group of lysine residues has also been extensively investigated. This is especially owing to the diverse array of acylating agents that can be employed for the formation of robustly stable amide-bonded conjugates; many of these conjugates have been clinically validated.^53^ However, the high abundance of accessible lysine residues on an antibody's surface represents a double-edged sword. While offering multiple available sites for modification, regio-selectivity is very difficult to achieve as many of the lysines harbour similar reactivity. This leads to the formation of highly heterogenous conjugates when targeting a conjugate loading lower than the total number of solvent accessible lysines, which is almost always the case as modifying all the lysines would cause significant issues to the protein (*e.g.*, solubility). Moreover, by the very nature of the modification strategy, it results in a highly varied and difficult to predict pharmacokinetic profile for the plethora of conjugates formed in the product mixture.^54^ More recently, and to obviate some of the aforementioned issues, several efforts toward enabling site-selective lysine modification and/or lysine-modified mono-labelling have been explored, these include: kinetically controlled labelling,^55–63^ affinity binding-directed modification,^64–80^ cooperative stapling^81–84^ (involving a lysine residue with another residue such as cysteine, arginine, tyrosine *etc.*),^85,86^ automated synthesis of stoichiometrically conjugated antibodies,^87,88^ linchpin-based strategies^89–92^ and modified group transfer strategies (rapid and reversible modification of a nearby amino acid (typically cysteine) to transfer reactive moieties to lysine(s)).^93–95^ These methods aim to achieve site-specific modification of lysine(s) with direct applications in the areas of ADCs,^96^ peptide–drug conjugates and the development of covalently lysine modified drugs.^64,65,97,98^ Despite many advancements, challenges still exist, *e.g.*, substrate tolerance (some of the proposed methods are limited to application to specific proteins or peptides, and thus lack versatility), reaction efficiency (low yields, long reaction times, complicated synthesise of key reagents, the need for bespoke protein-specific optimisation (time-consuming and costly) and/or high concentrations of reagents, which can limit practical utility), operational complexity (complex procedures can affect scalability), *etc.* These general issues necessitate the further refinement of strategies for site-selective lysine modification and/or lysine-modified mono-labelling of proteins to enable broader applications to be realised.

**Fig. 1:**
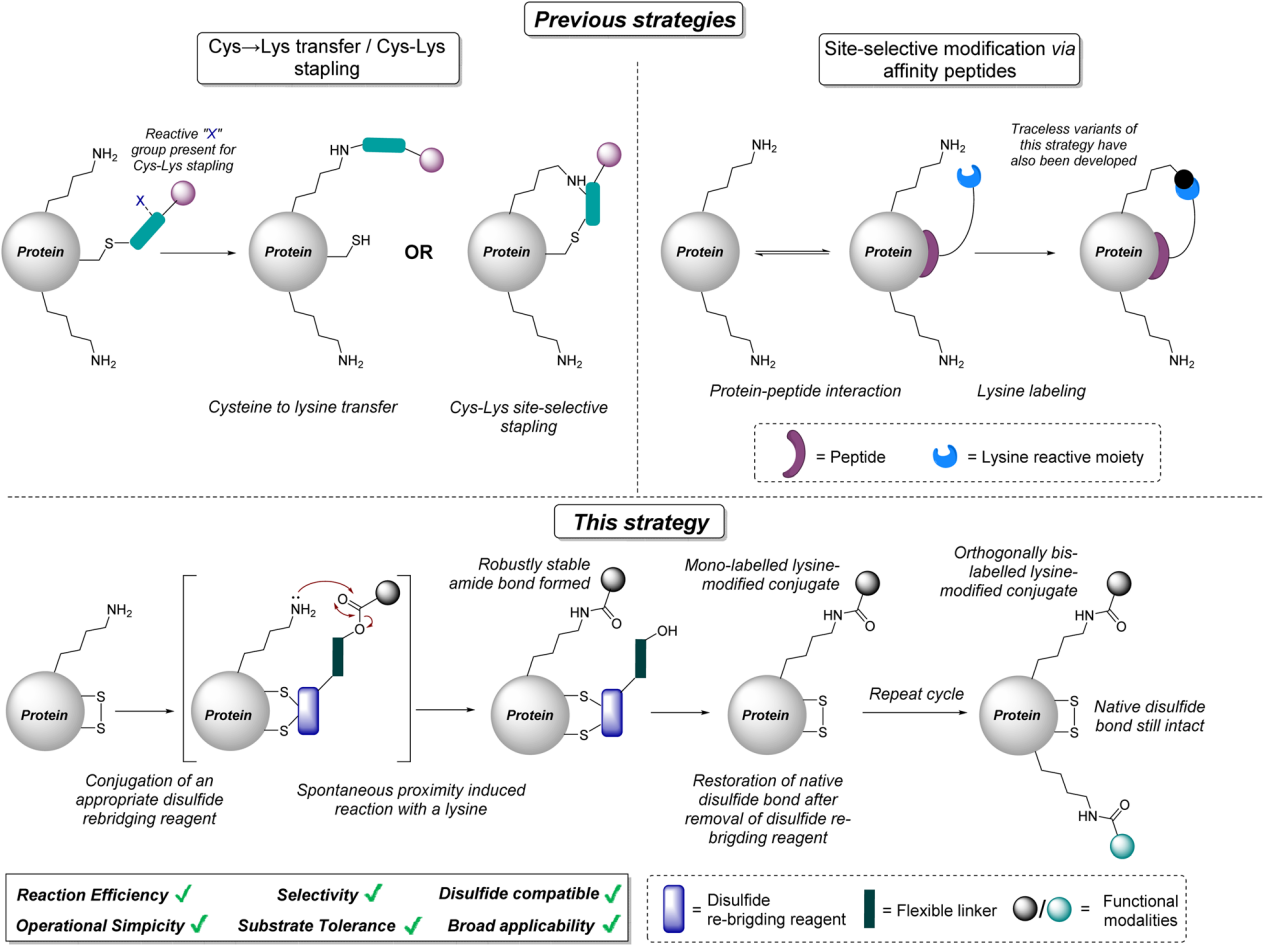
Existing strategies for site-selective lysine modification, and a newly proposed strategy to enable selective lysine modification on proteins *via* a combination of quantitative and reversible disulfide modification and proximity-induced intramolecular reactivity for the formation of mono-labelled lysine-modified protein conjugates, which can then be re-subjected to the reaction protocol using a different functional modality to yield a dually-labelled lysine-modified conjugates.

In this article, we overcome some of the aforementioned limitations (see Fig. 1) by exploiting (i) the controlled reversible site-selective modification of disulfides with dibromopyridazinediones (PDs) and (ii) proximity induced reactions with proximal lysines. Through careful optimisation of conjugation to disulfides and neighbouring lysines by limiting side-reactions, our methodology achieves lysine-modified mono-labelling of various proteins *via* amide bond formation reactions. The use of a click handle on the newly formed amide bond enables facile modular modification. The strategy was exemplified on multiple clinically relevant antibody Fabs with evidence of proximity induced reactivity (distal from the antibody fragment complementarity-determining region (CDR)), retention of the native disulfide bond, and retention of binding of the antibody conjugates to their native target verified by MS/MS, SDS-PAGE, LC-MS and/or ELISA analysis.

## Results and discussion

**Fig. 2:**
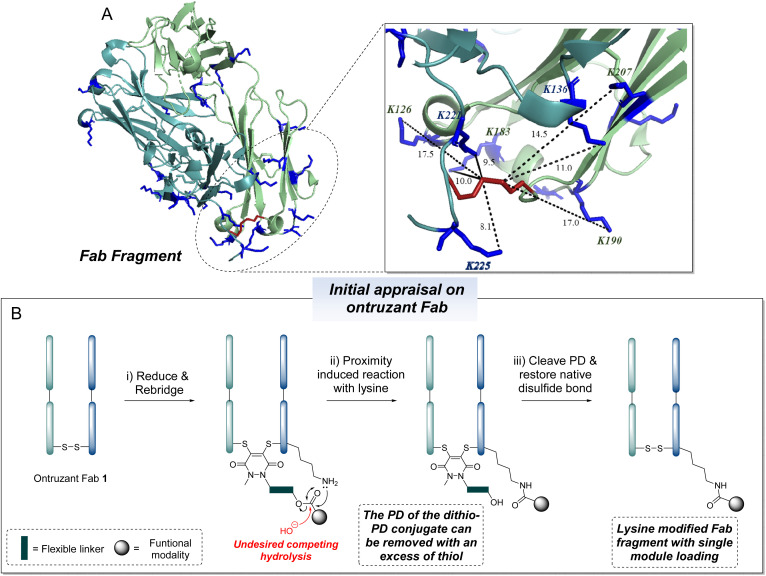
(A) Overlaid Fab crystal structure, derived from PDB files 1HZH (Human IgG against HIV-1, lacking four amino acids DKTH, K225) and 6BAE (trastuzumab Fab); interstrand disulfide bond cysteine side-chains are coloured in red, and lysine side-chains are coloured in blue. The distance (measured in Å) from the disulfide bond to the nitrogen of the side chain of the lysine are displayed to indicate relative proximity, while acknowledging the flexibility of the protein – it is acknowledged that these distances are approximate/dynamic as the protein is flexible. (B) Schematic representation of the proposed proximity induced lysine transfer strategy on Ontruzant Fab 1. Lysines on the light or heavy chain may be modified; the above graphical representation has been simplified as it is difficult to depict all possible regioisomers graphically.

Our study began by selecting an appropriate protein to appraise our proposed methodology. We chose the Fab fragment of Ontruzant (a biosimilar of Herceptin™/trastuzumab) as Herceptin™/trastuzumab has been used in numerous applications such as drug delivery and imaging (thus highlighting its clinical importance),^2,12,18,51,99–103^ it can be obtained in excellent quality and in high quantity (to facilitate thorough analysis, see ESI† for details), and as it contains a single disulfide bond and 26 lysine residues (thus enabling thorough appraisal/investigation of the viability of our proposed strategy). Computational analysis of crystallographic data derived from PDB file 6BAE (trastuzumab Fab) and PDB file 1HZH (human IgG1 against HIV-1, containing the final four amino acids DKTH on the heavy chain where K is Lys-225) identified the lysine residues in the region of the disulfide bond of the Fab. As shown in Fig. 2A, there are three lysines on the heavy chain (K136, K221, K225) and four lysines on the light chain (K126, K190, K183, K207) that are in relatively close proximity to the disulfide bond on the Fab – it is acknowledged that these distances are approximate/dynamic as the protein is flexible.

We next needed to appraise what disulfide modification technology would be appropriate. The proposed strategy (see Fig. 2B) requires a reagent that enables chemoselective disulfide rebridging and can be quantitatively removed with a specific trigger to re-liberate the native disulfide bond. An appropriate class of reagent are PDs, which are excellent candidates for re-bridging a reduced disulfide and can also be quantitatively removed with an excess of thiol to restore a native disulfide linkage.^104^ As such, as shown in Fig. 2B, a general method could be envisaged for the aforementioned strategy to mono-label Ontruzant Fab 1*via* lysine-based modification; it is based on: (i) a PD unit (to chemoselectively react with the reduced disulfide of a Fab and then be selectively cleaved in the final step to restore the native disulfide bond); (ii) a lysine-reactive moiety (that should only react with a lysine *via* proximity induced intramolecular reactivity); and (iii) a flexible linker to connect the two reactive groups (this can potentially be used to tune the length/rigidity of the molecule to enable efficient proximity induced reactivity depending on the protein system that is being used).

Phenyl esters and *ortho*-modified derivatives thereof have been reported in the literature as reagents that can be used for lysine modification including applications in labelling,^105–107^ as well as in peptide and antibody modification.^108–111^ Therefore, we initially focused on making constructs based on having a phenolic ester as our lysine reactive group. That being said, it was appreciated from the outset that phenyl esters are prone to hydrolysis and that this was a potential limitation that we may need to address. Reaction of 4-(aminomethyl)phenol with a carboxylic acid-bearing PD led to the formation of a PD featuring a phenol moiety. This molecule was subsequently coupled with various carboxylic acids to form PD-ester derivatives 2, including PD-ester azides 2b–d which would enable subsequent and facile modular Cu-free strain-promoted azide–alkyne click chemistry (SPAAC) functionalisation. The preparation of various PD-ester-azides would enable investigation into how the sterics and distance between the electron-withdrawing azide^112^ may affect conjugation and/or hydrolysis.

**Fig. 3:**
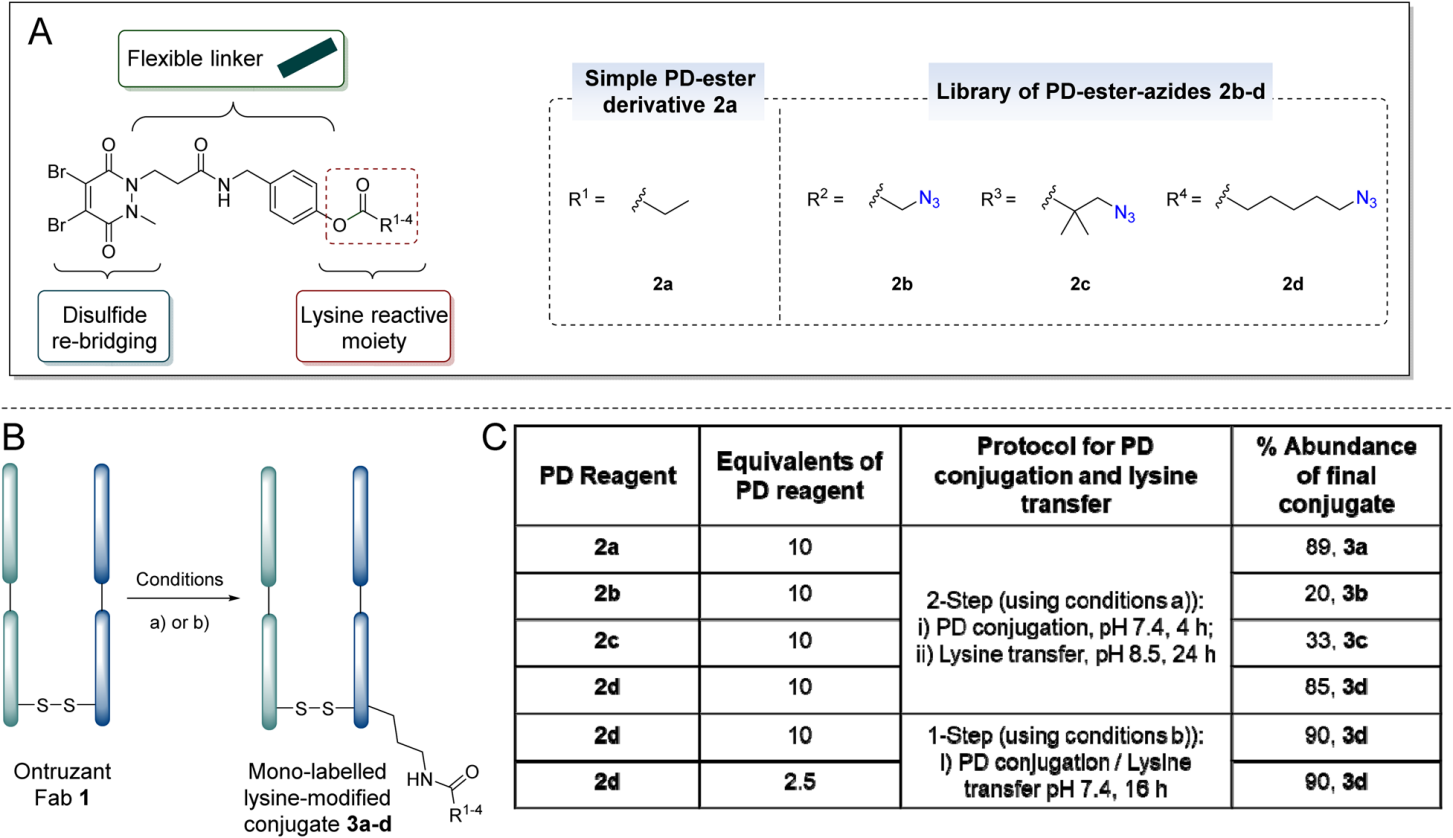
(A) A general PD-ester scaffold highlighting its two key features and the specific DiBrPD-ester reagents 2a–d that were made. (B) Reaction scheme for conversion of Ontruzant Fab 1 to mono-labelled lysine-modified conjugates 3a–d: (a) reaction conditions using 2-step PD conjugation and lysine transfer protocol: (i) TCEP (10 eq., 20 mM in DI H_2_O), 90 min and then addition of compound 2a–2d (10 eq., 10 mM in MeCN) in PBS (pH 7.4, 2 mM EDTA), 4 h, 37 °C, (ii) incubation in pH 8.5 (2 mM EDTA), 24 h, (iii) DTT (175 eq., 50 mM in DI H_2_O), 1 h; followed by oxidation in BBS (pH 8.0, no EDTA). (b) Reaction conditions using 1-step PD conjugation and lysine transfer protocol: (i) TCEP (10 eq., 20 mM in DI H_2_O), 90 min and then addition of compound 2d (2.5 eq., 10 mM in MeCN) in PBS (pH 7.4, 2 mM EDTA), 16 h, 37 °C, (iii) DTT (175 eq., 50 mM in DI H_2_O), 1 h; followed by oxidation in BBS (pH 8.0, no EDTA). All reactions were conducted at 37 °C. (C) Table depicting the % abundance of mono-labelled final conjugates 3a–d, equivalents used, and what protocol was employed. Lysines on the light or heavy chain may be modified; the above graphical representation has been simplified as it is difficult to depict all possible regioisomers graphically.

To appraise the feasibility of the strategy, and optimise the reaction conditions, non-azide bearing PD-ester 2a was initially reacted with Ontruzant Fab 1 (reagent 2a, Fig. 3C). As mentioned earlier, the strategy consists of the following steps: (i) conjugation of PD to the reduced disulfide, (ii) proximity induced reaction of the ester with a lysine residue, (iii) removal of the PD and restoration of the native disulfide bond. A series of reaction parameters for the conjugation and lysine reaction step were appraised (*e.g.*, pH, duration of reaction, temperature, *etc.*). The most optimal conditions were found to be the use of 10 eq. of reagent 2a in PBS (pH 7.4, 2 mM EDTA) for 4 h for the conjugation step, and the use of BBS (pH 8.5, 2 mM EDTA) for 24 h for the proximity induced lysine reaction step. The conditions for the removal of the PD were found to be the use of dithiothreitol (DTT) at 175 eq. in BBS (pH 8.0, no EDTA). Finally, we discovered that the restoration of the native disulfide bond can be achieved *via* two simple options: oxidation could be achieved by incubation in an EDTA-free buffer at 37 °C for 3 h, or it could be carried out using 200 equivalents of dehydroascorbic acid (DHA, 50 mM in DI H_2_O) in BBS (pH 8.0, no EDTA) for 2 h.

Initial studies using reagent 2a suggested near quantitative conjugation of the PD onto Fab as determined by LC-MS and SDS-PAGE analysis (see ESI† for details). In the conjugation step, the formation of a side product (*ca.* 10%) resulting from the hydrolysis of the ester moiety was observed (see Fig. 2B, red arrow). Upon buffer swapping to pH 8.5, the desired proximity induced lysine reaction was achieved whilst the hydrolysed ester conjugate persisted (*ca.* 11%). Upon addition of DTT and then subsequent purification into EDTA-free water to restore the native disulfide bond, LC-MS analysis revealed the formation of desired conjugate 3a in 89% conversion, with the remaining 11% abundance of native Fab presumably stemming from the hydrolysis side-product.

The preliminary studies on reagent 2a demonstrated the feasibility of the strategy, resulting in the successful formation of mono-labelled lysine-modified Fab conjugate 3a in high conversion, but it also highlighted a hydrolysis side-reaction (see Fig. 2B, red). We next appraised PD-ester-azide reagents 2b–2d in the aforementioned optimised reaction conditions (Fig. 3B). We anticipated that PD-ester-azide 2b, which harbours the shortest alkyl chain length from ester to azide, would have the most reactive ester group. This hypothesis was soon confirmed, as when conjugating PD-ester-azide 2b to reduced Ontruzant Fab 1, additional acylations were observed. This indicated that unselective intermolecular reactions were taking place between the Fab and the ester component of reagent 2b. Substantial hydrolysis of the ester was also observed when using this reagent (*ca.* 55%). Reagent 2c, bearing a geminal dimethyl group to confer improved hydrolytic stability in the PD conjugation step, was next to be appraised.^106^ Whilst efficient PD disulfide re-bridging was observed with a small amount of hydrolysis (*ca.* 10%), lysine transfer was substantially inhibited (only *ca.* 33% conversion to desired conjugate 3c was observed).

The application of reagent 2d, which has a longer chain length, in the aforementioned reaction sequence yielded the highest abundance of desired singly modified Fab 3d for all the azide-based reagents (*ca.* 85%) with only *ca.* 15% of native Fab (see Fig. 3C). The longer alkyl chain length proved to be optimal in achieving a good rate of lysine transfer *vs.* unwanted hydrolysis whilst also facilitating the attachment of an azide click handle. Having identified reagent 2d, which facilitated high formation of the desired mono-labelled lysine-modified conjugate, it was decided to investigate the feasibility of achieving conjugation and lysine reaction processes in a single step reaction. Such an approach would enhance efficiency and may result in a higher conversion. As described in the ESI (Section 2.4),† a series of reactions were conducted involving the reaction of reagent 2d with reduced Fab using 1-step for PD conjugation and lysine reaction – it was concluded that such a procedure could be enabled when the conjugation and lysine transfer reactions took place at pH 7.4 for 16 h. This simpler and faster procedure resulted in an improved 90% abundance of mono-labelled conjugate 3d as determined by LC-MS. Moreover, in the course of these experiments, it was determined that only 2.5 eq. of reagent 2d was needed to achieve *ca.* 89% of mono-labelled conjugate 3d.

**Fig. 4:**
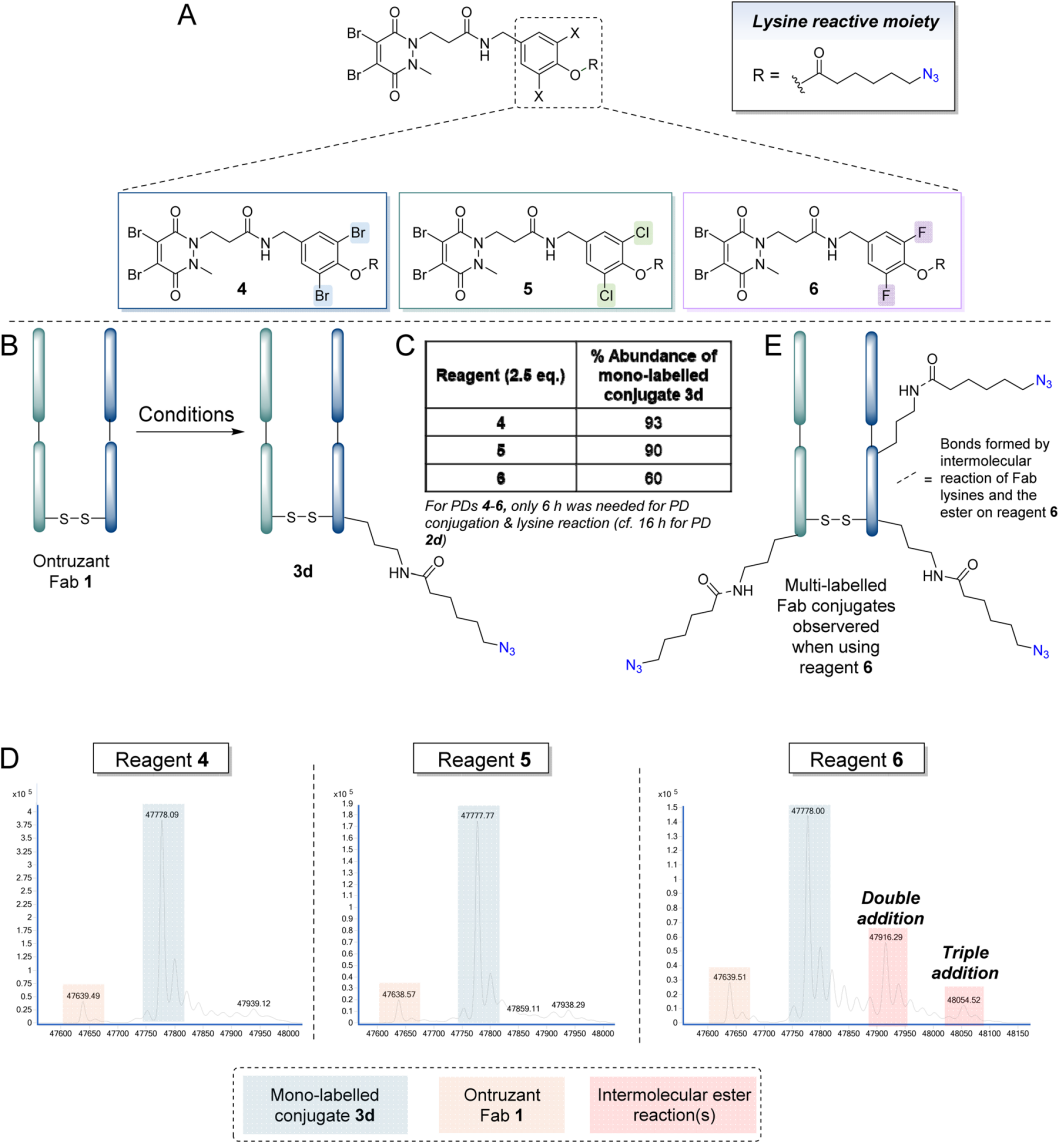
(A) Structures of reagents 4–6. (B) Reaction of reagents 4–6 in the following protocol: (i) TCEP (10 eq., 20 mM in DI H_2_O), 90 min and then addition of compound 4, 5 or 6 (2.5 eq., 10 mM in MeCN) in PBS (pH 7.4, 2 mM EDTA), 6 h, (iii) DTT (175 eq., 50 mM in DI H_2_O), 1 h; followed by incubation in BBS (pH 8.0, no EDTA). All reactions were conducted at 37 °C. Lysines on the light or heavy chain may be modified; the above graphical representation has been simplified as it is difficult to depict all possible regioisomers graphically. (C) Table depicting the % abundance of mono-labelled final conjugate 3d for each reagent used. (D) Deconvoluted MS data for after the final step to make conjugate 3d (zoom in of mass range of Fab region) when using reagents 4, 5 and 6. (E) Multi-labelled Fab conjugates observed when using reagent 6. Lysines on the light or heavy chain may be modified; the above graphical representation has been simplified as it is difficult to depict all possible regioisomers graphically.

We next turned our attention to the phenolic component to try to fine tune the reaction further. Inspired by reports on the use of *ortho*-substituted di-halogen phenyl esters for the generation of protein conjugates modified at lysine(s), due to apparent increased efficiency in terms of lysine reactivity and/or increased hydrolytic stability of the ester,^106^ it was decided to create a library of *ortho*-substituted di-halogen analogues of reagent 2d, *i.e.*, *ortho*-dibromophenolate 4, *ortho*-dichlorophenolate 5 and *ortho*-difluorophenolate 6. The experimental pKa values of *ortho*-dibromophenol, *ortho*-dichlorophenol and *ortho*-difluorophenol and phenol are 6.67, 6.78, 7.51 and 9.90 respectively.^113^ Whilst the dibromo and dichloro reagents could be expected to be the most reactive on the basis of this data, the opposing effect of sterics made it difficult to *prima facie* predict what reagent would fare best. The study commenced with *ortho*-dibromo reagent 4 following the protocol described in Fig. 4A, based on reports of the increased hydrolytic stability of this moiety.^90,105^ Use of 2.5 eq. of reagent 4 and incubation for only 6 h (*cf.* 16 h) in PBS (pH 7.4, 2 mM EDTA) to achieve PD conjugation and lysine modification (presumably due to the ester being more reactive) was enough to achieve a ∼91% abundance percentage of final, mono-azide-labelled conjugate 3d. Reagents 5 and 6 were also appraised and whilst *orth*o-dichloro reagent 5 (2.5 eq.) resulted in a similar outcome (∼90% mono-labelled Fab conjugate 3d), the most reactive difluoro ester reagent 6 afforded double and triple labelled products that were almost certainly derived from additional intermolecular reactions between the ester component of reagent 6 and lysine residues on the Fab (Fig. 4D and E). This hypothesis was supported by control reactions of PD reagents 4–6 with native Fab under the reaction conditions showing reactivity only when using PD reagent 6 (see ESI† for details).

In order to determine the rate of inherent ester hydrolysis of the reagent 4 in aqueous buffer conditions alone, a stability study on reagent 4 was conducted *via* NMR spectrometry analysis. In order to mimic the bioconjugation reaction conditions, reagent 4 (5 mM final solution) was incubated in PBS (pH 7.4, 2 mM EDTA) : CD_3_CN (50 : 50) at 37 °C (300 rpm). Time points at *t* = 0, 1, 6, 24 and 48 h were collected (see ESI† for details). Based on the experimental data, reagent 4 exhibited no hydrolysis up to the 48 h time point, demonstrating hydrolytic stability in the buffer (see ESI† for details). This study suggested that the premature hydrolysis may be due to the protein microenvironment.

**Fig. 5:**
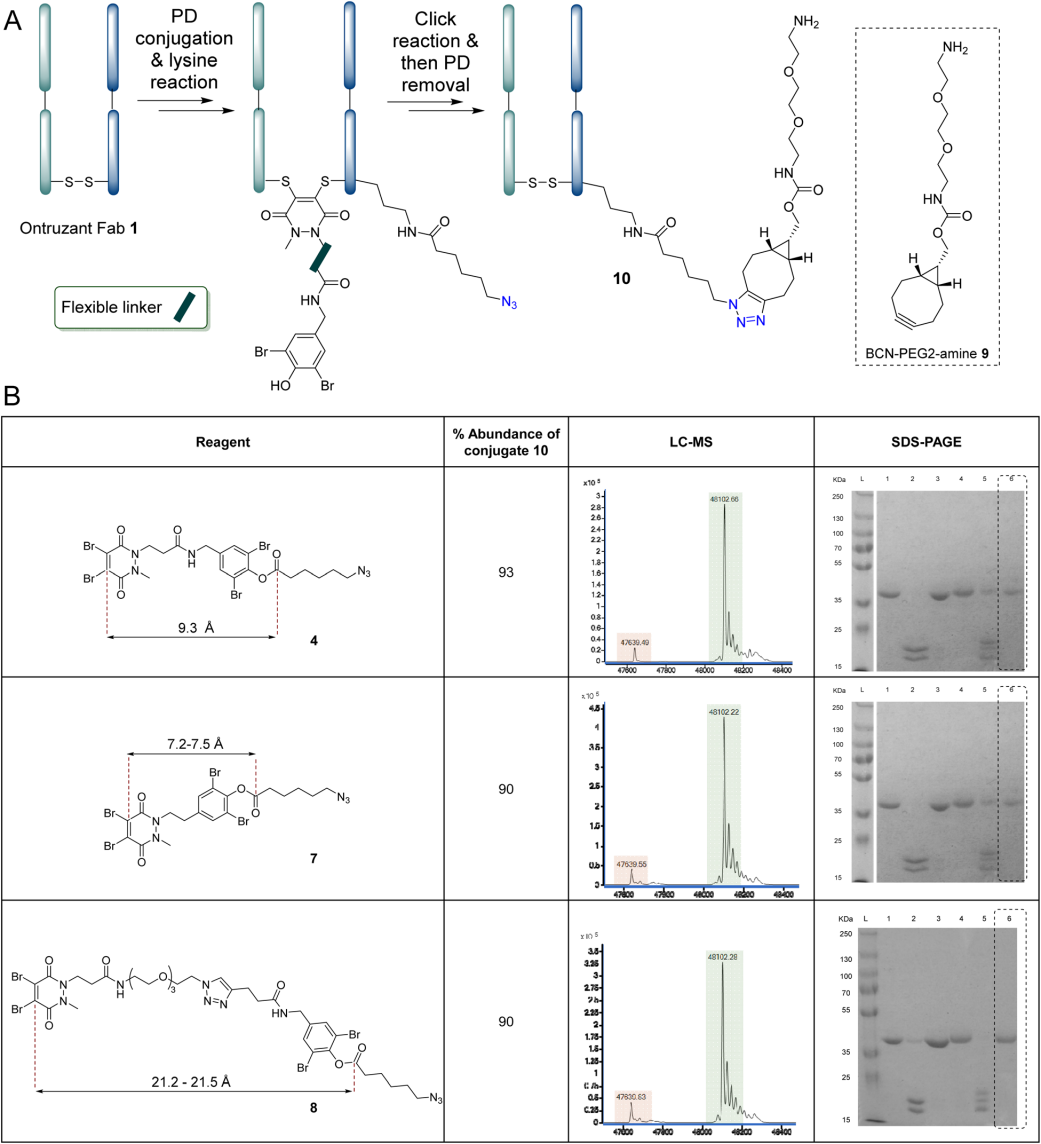
(A) General scheme for the conversion of Ontruzant Fab 1 to conjugate 10 using the following conditions: (i) TCEP (10 eq., 20 mM in DI H_2_O), 90 min and then addition of compound 4, 7 or 8 (2.5 eq., 10 mM in MeCN) in PBS (pH 7.4, 2 mM EDTA), 6 h, (ii) BCN-PEG2-amine 10 (10 eq., 10 mM in DMSO) in BBS (pH 8.0, no EDTA), 16 h, (iii) DTT (175 eq., 50 mM in DI H_2_O), 2 h for reagents 4 and 8, 5 h for reagent 7 followed by oxidation in BBS (pH 8.0, no EDTA). All reactions were conducted at 37 °C. Lysines on the light or heavy chain may be modified; the above graphical representation has been simplified as it is difficult to depict all possible regioisomers graphically. (B) Table displaying the structures of reagents 4, 7 and 8, as well as (for each reagent) the % abundance of conjugate 10 and its analysis by LC-MS and SDS-PAGE (L: Ladder, 1: Ontruzant Fab 1, 2: Reduction of Fab, 3: PD 4, 7 or 8 conjugation, 4: Click reaction, 5: PD removal, 6: Restoration of disulfide bond).

We next set out to explore the effect of the flexible linker component of the scaffold on the strategy. We wanted to investigate whether adjusting the length/rigidity of the molecule would result in more, less or similarly efficient proximity-induced modification *vs.* competing ester hydrolysis. To appraise this, reagents 7 and 8 (see Fig. 5B) were synthesised (see ESI† for details). The library of reagents 4, 7 and 8 would thus enable us to investigate the relationship between linker length and the effectiveness of the protocol described above. According to the LC-MS results, mono-azide-labelled lysine conjugate 3d was detected with abundance of ∼89% and ∼90% when using reagents 7 and 8 (respectively), suggesting they had a similar reactivity profile to reagent 4 (see ESI† for details). Nonetheless, we assumed that the shorter linker species may result in only the most proximal lysines being modified and we next appraised if this assumption would be borne out by carrying out the click reaction prior to the final PD removal step as this would exacerbate any potential differences in sterics (see Fig. 5A). Moreover, performing the click reaction prior to PD removal (see Fig. 5A) is ideal (more generally) due to the potential issues of DTT compromising the stability of certain azides^114,115^ and/or other click handles (*e.g.* strained alkynes, *etc.*) if they were to be used in the linker. In view of this, a simple BCN-PEG2-amine 9 (10 eq., 10 mM in DMSO) was added after the lysine transfer step and the reaction was incubated in the dark for 16 h at 37 °C prior to PD removal and disulfide bond restoration. According to LC-MS, all the click reactions proceeded in quantitative conversion, but a difference was observed in the following DTT-based PD removal step where a longer time was needed for the conjugate made using the short linker (PD 7, 5 h was required compared to 2 h for the medium and longer linkers PDs 4 and 8). Nonetheless, as shown in Fig. 5B, the final mono-clicked lysine modified Fab conjugate 10 was obtained in conversions of 90–93% for reagents 4, 7 and 8.

To appraise the exact sites of modification, the final conjugates 10 obtained from reaction with reagents 4, 7 and 8 were subjected to chymotryptic digestions and analysed by LC-MS/MS analysis (see ESI† for details). Consistent with our expectations (see Fig. 2A), four lysine residues on the light chain (K126, K183, K190, K207) and three on the heavy chain (K136, K221, K225) were modified with the presumably most proximal/reactive lysine (K225) being modified most (*ca.* 25%) in all cases (see ESI† for details). Thus, all modifications occurred distant from the CDR region and proximal to the disulfide, but, and perhaps somewhat surprisingly, a significant difference in the distributions of the lysines modified in relation to the three different length linkers was not observed. Nonetheless, as expected, since the modification took place distal from the CDR, the final lysine-modified conjugates (10) formed from using reagents 4, 7 and 8 demonstrated full retention of binding activity relative to the Ontruzant Fab 1 (see ESI† for details). Based on the optimisation studies on the flexible linker outlined earlier, it was concluded that the linker length had no significant impact on proximal lysine reaction *vs.* hydrolysis. Due to the extended time required for removal of the PD when using reagent 7, the potential risks *in vivo* (*i.e.* immunogenicity) associated with the PEG chain employed for reagent 8,^116^ the higher complexity of the multi-step synthesis required of both reagents 7 and 8, and the higher conversion when using reagent 4, it was decided to proceed with reagent 4 for the remainder of the studies. Nonetheless, these results do demonstrate that the strategy is amenable to using linkers with different lengths and for systems where a lysine is not proximal to a disulfide (or cysteine) modification site, a longer length linker entity could be used.

**Fig. 6:**
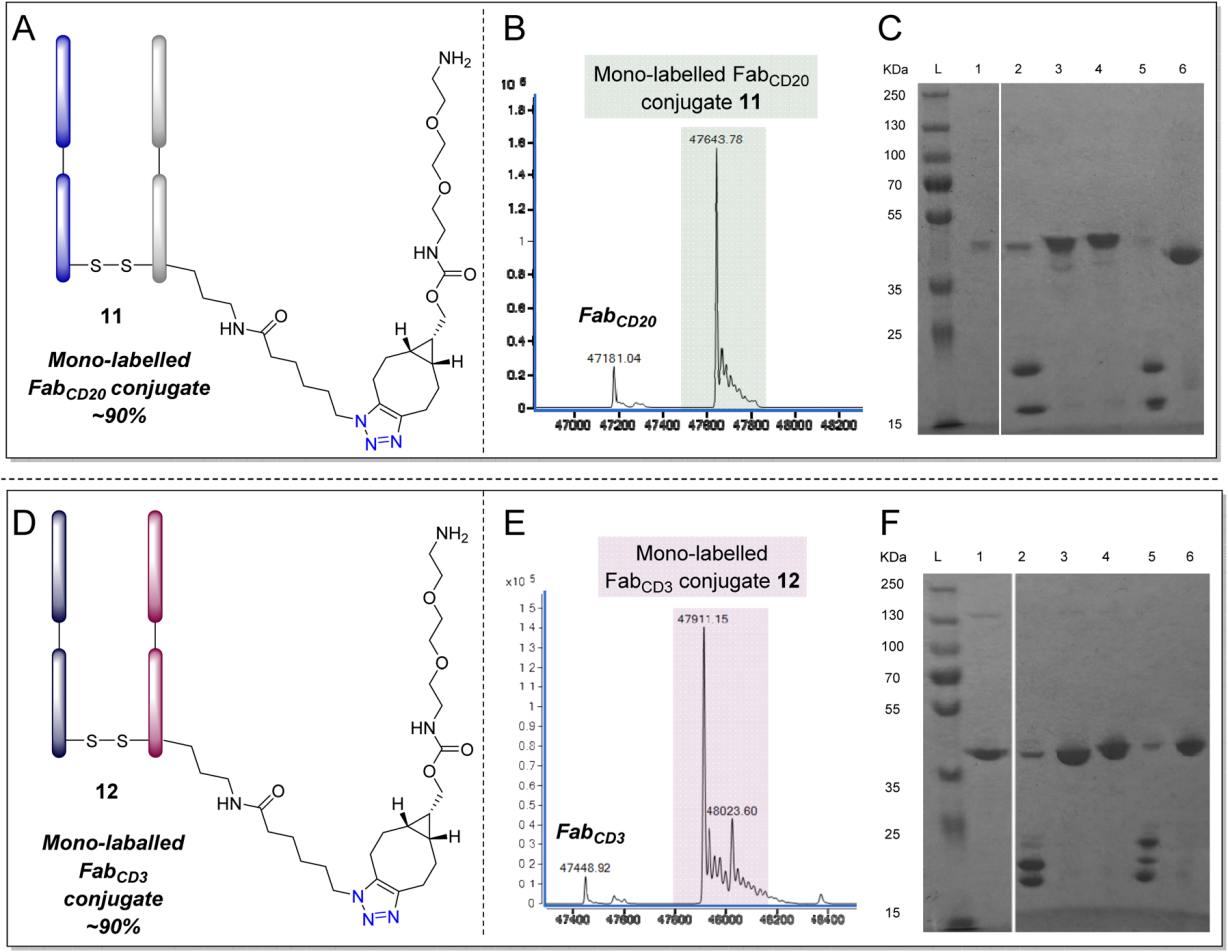
Application of the optimised protocol for mono-labelling and clicking of a Fab to Fab_CD20_ and Fab_CD3_ – “optimised protocol”: (i) TCEP (10 eq., 20 mM in DI H_2_O), 90 min and then addition of compound 4 (2.5 eq., 10 mM in MeCN) in PBS (pH 7.4, 2 mM EDTA), 6 h, (iii) BCN-PEG2-amine 10 (10 eq., 10 mM in DMSO) in BBS (pH 8.0, no EDTA), 16 h, (iv) DTT (175 eq., 50 mM in DI H_2_O), 2 h; followed by oxidation in BBS (pH 8.0, no EDTA). (A) Structure of clicked mono-labelled lysine modified Fab_CD20_11. Lysines on the light or heavy chain may be modified; the above graphical representation has been simplified as it is difficult to depict all possible regioisomers graphically; (B) LC-MS analysis of Fab_CD20_ conjugate 11 formed in the final step; (C) SDS-PAGE analysis (L: Ladder, 1: Fab_CD20_, 2: Reduction of Fab, 3: PD 4 conjugation, 4: Click reaction, 5: PD removal, 6: Restoration of disulfide bond); (D) structure of clicked mono-labelled lysine modified Fab_CD3_12. Lysines on the light or heavy chain may be modified; the above graphical representation has been simplified as it is difficult to depict all possible regioisomers graphically; (E) LC-MS analysis of Fab_CD3_ conjugate 12 formed in the final step, (F) SDS-PAGE analysis (L: Ladder, 1: Fab_CD3_, 2: Reduction of Fab, 3: PD 4 conjugation, 4: Click reaction, 5: PD removal, 6: Restoration of disulfide bond). Note that in lane 2 of both SDS-PAGE some oxidised Fab is observed, this is likely a feature of oxidation taking place when preparing the sample.

To appraise the broader application of the proposed strategy, it was decided to investigate the application of PD reagent 4 on other readily available antibody Fabs of therapeutic relevance such as Fab_CD20_ and Fab_CD3_.^117–119^ Rituximab is a chimeric mAb against CD20, primarily located on the surface of B cells and is widely employed to treat cancer and autoimmune diseases.^118^ Muromonab, or Orthoclone OKT3, is a murine anti-CD3 antibody and was the first monoclonal antibody approved for preventing organ transplant rejection.^120^ Both Rituximab and OKT3 fragments have been engineered and utilized in various bispecific antibodies to overcome resistance mechanisms.^121^ Fab_CD20_ and Fab_CD3_ fragments were obtained *via* enzymatic digestions of commercially available native anti-CD20 (rituximab) and anti-CD3 (OKT3) respectively, adhering to previously outlined methods.^122,123^ As depicted in Fig. 6, clicked mono-labelled lysine modified Fab_CD20_ conjugate 11 and Fab_CD3_ conjugate 12 were both formed in 90% abundance percentage as determined by LC-MS and SDS-PAGE when using the optimised reaction protocol (see Fig. 6 caption).

**Fig. 7:**
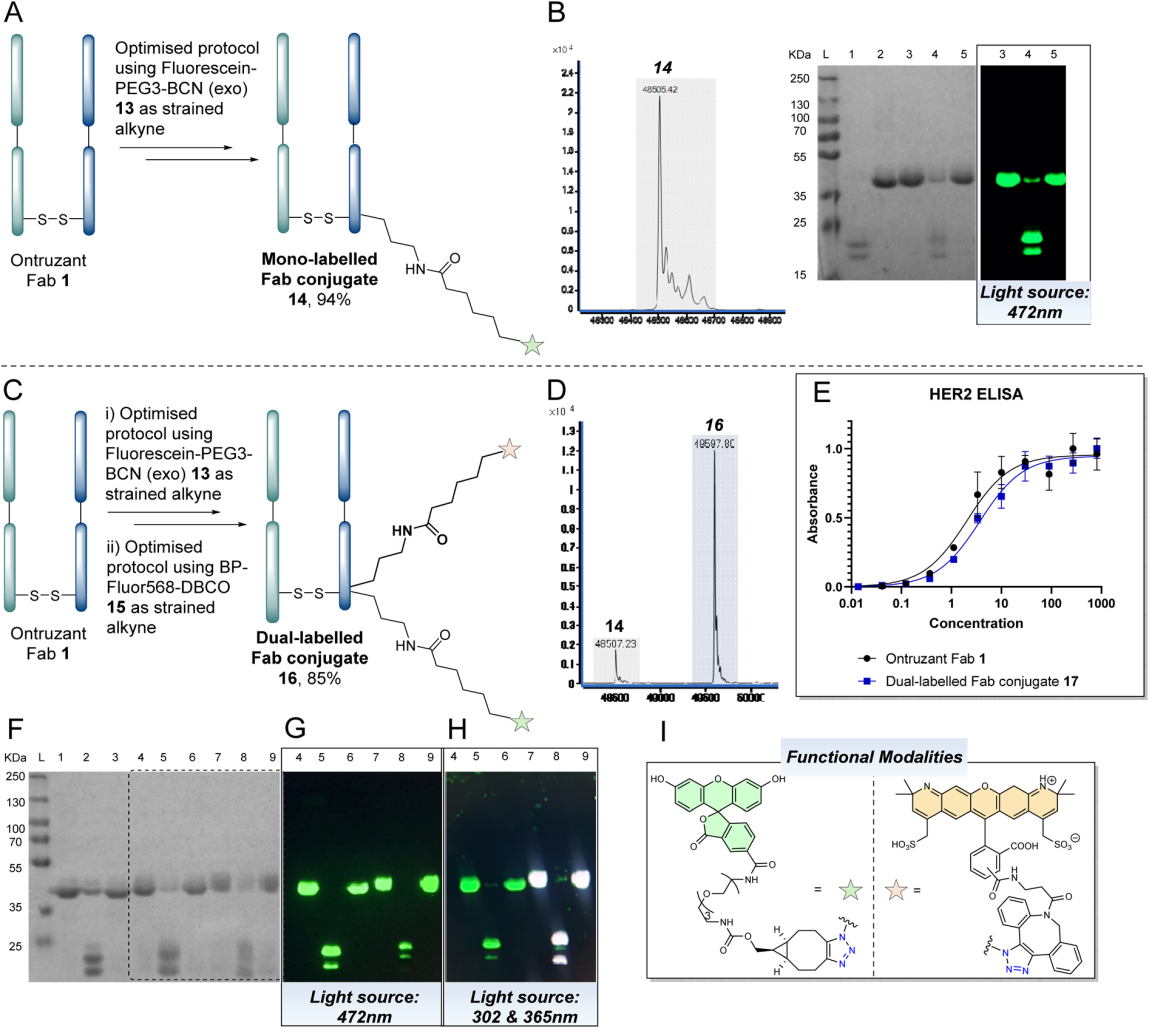
(A) Application of the optimised protocol for mono-labelling and clicking of a Fab (see Fig. 6 for details) on Ontruzant Fab 1 using 5-FAM-PEG3-BCN (exo) 13 as the strained alkyne component to afford mono-labelled Fluorescein Fab conjugate 14. Lysines on the light or heavy chain may be modified; the above graphical representation has been simplified as it is difficult to depict all possible regioisomers graphically; (B) LC-MS and SDS-PAGE analysis of mono-labelled fluorescein Fab conjugate 14. The final % abundance of conjugate 14 was determined by analysis of a previous conjugate in the sequence as the disulfide bond of the Fab did not appear to be fully reformed in this particular experiment (see ESI† for details). (C) Application of the optimised protocol for mono-labelling and clicking of a Fab (see Fig. 6 for details) on Ontruzant Fab 1 in two cycles using 5-FAM-PEG3-BCN (exo) 13 (in cycle 1) and BP-Fluor568-DBCO 15 (in cycle 2) as the strained alkyne components and bypassing the disulfide restoration step at the end of cycle 1 to afford dually-labelled Fab conjugate 16. Lysines on the light or heavy chain may be modified; the above graphical representation has been simplified as it is difficult to depict all possible regioisomers graphically; (D) LC-MS analysis of dually-labelled Fab conjugate 16, (E) ELISA study on a dually-labelled Fab conjugate 17 prepared using BCN-PEG2-amine 9 and a DBCO-biotin (see ESI† for details), (F–H) SDS-PAGE analysis of protocol (L: Ladder, 1: Ontruzant Fab 1, 2: Reduction of Fab, 3: PD 4 conjugation, 4: Click reaction with 5-FAM-PEG3-BCN (exo) 13, 5: PD removal, 6: PD 4 conjugation, 7: Click reaction with BP-Fluor-DBCO 15, 8: PD removal, 9: Restoration of disulfide bond) using various light sources for analysis. (I) Functional modalities present on dual clicked Fab conjugate 16.

With the above discoveries in mind, we attempted to form a dually modified Fab bearing two different fluorophores by using cycles of the optimised reaction protocol. To the best of our knowledge, this would be the first example of controlled differential modification of the solvent accessible lysines on a native antibody fragment. The first stage involved application of the proposed strategy on Ontruzant Fab 1 using a click reaction with 5-FAM-PEG3-BCN (exo) 13, as shown in Fig. 7A. LC-MS and SDS-PAGE analysis confirmed the formation of the mono-labelled BCN-fluorescein clicked conjugate 14 in *ca.* 94% abundance (Fig. 7B). We next repeated two cycles of the optimisation protocol on Ontruzant Fab 1 using 5-FAM-PEG3-BCN (exo) 13 for the first click reaction and BP-Fluor568-DBCO 15 for the second click reaction. We chose to use two different strained alkynes to demonstrate tolerability. To our delight, LC-MS and SDS-PAGE analysis of the final conjugate revealed the formation of dually modified Fab 16 in *ca.* 85% abundance.

To further exemplify the technology and to enable ELISA analysis (*i.e.*, to prevent UV-vis absorption interference with the ELISA analysis), a further dually-modified Fab conjugate (17) was prepared using BCN-PEG2-amine 9 and DBCO-biotin (see ESI† for details) as the click partners. Pleasingly, this conjugate was also made in 85% abundance, and the ELISA assay indicated no decrease in binding relative to native Fab (Fig. 7E). As with the previous reactions, SDS-PAGE analysis corroborated these findings (Fig. 7F). As shown in Fig. 7, visualisation of the gel using a light source of 472 nm (Fig. 7G) and 302 nm + 365 nm (Fig. 7H) showed differentiation between the fluorescein only modification and when both fluorophores were attached to the Fab (see ESI† for further details). It is acknowledged that the signal intensity of the mass spectra of the conjugates decreases as one goes through the reaction sequence – we believe this is due to the loss of material over the sequence of steps.

We finally appraised our optimised protocol on the full antibody of Ontruzant, a multi-disulfide system, by scaling the amount of PD 4 per disulfide bond. However, use of these conditions resulted in a complex mixture of products (see ESI† for details) that could perhaps be anticipated/rationalised by competing thiolate-based reaction of the ester moiety of PD 4 post-conjugation to a reduced disulfide bond, *i.e.*, once the first PD reacts with a reduced disulfide bond, the ester reacts (intramolecularly) with a reduced disulfide bond thiolate faster than the thiolate conjugates to another molecule of PD 4 (intermolecularly). As such, there is still some work to be carried out to adapt this strategy to multi-disulfide systems.

## Conclusion

Bioconjugation to native solvent-accessible lysines on antibodies (and other proteins) has been utilized for many years, however, numerous limitations have been identified owing to lack of selective and/or controlled modification (*e.g.*, batch-to-batch variability, propensity for aggregation, reduced protein function, *etc.*). Whilst strategies have been developed to try and address this, in this manuscript, we provide a new platform to help overcome some of these limitations by presenting a novel strategy to obtain lysine-modified mono-labelled Fab fragments *via* the formation of stable amide bonds. The selective modification of lysine residues is based on exploiting the quantitative and reversible site-selective modification of disulfides by using pyridazinediones that can enable near-quantitative proximity induced reactions with lysines on several antibody Fab fragments. The strategy was shown to be amenable to modular Cu-free click chemistry, proven to only modify lysines that are proximal to the native single disulfide bond (and thus distal from the CDR), and the final conjugates were shown to retain the original disulfide bond. The strategy was successfully demonstrated on three clinically relevant antibody Fab fragments (Fab_HER2_, Fab_CD20_ and Fab_CD3_). Moreover, through the use of multiple cycles of the aforementioned strategy, we were able to furnish a dually-labelled lysine-modified Fab fragments bearing two different functional modalities (using BCN and DBCO derivatives) in excellent conversion. The Fab conjugates were analysed *via* LC-MS, SDS-PAGE and ELISA studies and MS–MS analysis to determine the exact sites of modification and this verified the principle design of the strategy. It is envisaged that this methodology, which facilitates the straightforward formation of mono- and dual-labelled lysine-modified Fab conjugates in excellent conversions, will have broad ranging applications owing to the need for novel site-selective lysine modification strategies to enable novel applications in therapeutics, diagnostics, imaging and related fields.

## Data availability

Synthetic chemistry experimental details, including synthetic procedures and compound characterizations, *i.e.*, NMR, IR & MS spectra, chemical biology experimental details, including bioconjugation procedures, details on antibody digestion, LC-MS methodology, full LC-MS spectra including TIC and raw data, and LC-MS/MS have been included in the Electronic Supplementary Information (ESI) file.†

## Author contributions

I. A. T. synthesised the small molecules. I. A. T. carried out the bioconjugation experiments on the Fabs. T. B. carried out some of the experiments on small molecules re. stability studies by NMR. P. A. S. generated the FabCD_20_ fragment. L. N. C. R. generated the Fab_CD3_ fragment. C. M., N. W. and I. A. T. conducted the ELISA studies. I. A. T. conducted the Fab digestion protocols N. B. analysed the LC-MS/MS data. N. B., E. A. L. and A. M. helped to support I. A. T. in their time at LifeArc and during their PhD. I. A. T., J. R. B. and V. C. conceived and designed the project/experiments. I. A. T. and V. C. co-wrote the manuscript. All authors read and approved the final manuscript.

## Conflicts of interest

There are no conflicts to declare, but we highlight that V. C. and J. R. B. are directors of UCL spin-out ThioLogics.

## Supplementary Material

SC-OLF-D4SC06500J-s001
