# Supplementary material for: Formation of mono- and dual-labelled antibody fragment conjugates via reversible site-selective disulfide modification and proximity induced lysine reactivity
Source: Chem Sci. 2025 Jan 6;16(6):2763–76. doi: 10.1039/d4sc06500j (PMC11726237; doi:10.1039/d4sc06500j)
Supplement: SC-016-D4SC06500J-s001 [file SC-016-D4SC06500J-s001.pdf]

Supplementary Information (SI) for Chemical Science.
This journal is © The Royal Society of Chemistry 2025

Formation of mono- and dual-labelled antibody fragment conjugates via reversible site-selective disulfide modification and proximity induced lysine reactivity

Ioanna A. Thanasi,[a] Nathalie Bouloc,[b] Clíona McMahon,[a] Ning Wang,[a] Peter A. Szijj,[a] Tobias Butcher,[a] Lea N. C. Rochét,[a] Elizabeth A. Love,[b] Andy Merritt,[b] James R. Baker,[a,]* and Vijay Chudasama[a,]*

[a] Department of Chemistry, University College London, 20 Gordon Street, London WC1H 0AJ, UK.

[b] LifeArc, Accelerator Building Open Innovation Campus, Stevenage SG1 2FX, UK.

* Correspondence: v.chudasama@ucl.ac.uk, j.r.baker@ucl.ac.uk

General experimental

Chemicals

All reagents were purchased from Sigma Aldrich, Fluorochem Ltd, Alfa Aesar or Lumiprobe and were used as received without purification, unless stated otherwise.

Chromatography

All small molecule reactions were monitored by thin-layer chromatography (TLC) on pre-coated SIL G/UV254 silica gel plates (254 μm) purchased from VWR. TLC plates were initially examined under short wave UV light and then developed using aqueous potassium permanganate or ninhydrin stains, when appropriate. Flash column chromatography was carried out with pre-loaded FlashPure flash cartridges on a Biotage® Isolera Spektra One flash chromatography system.

Spectroscopy

^{1}H and ^{13}C NMR spectra were recorded at room temperature on a Bruker Avance 300 instrument operating at a frequency of 300 MHz for ^{1}H and 75 MHz for ^{13}C, a Bruker Avance 500 instrument operating at a frequency of 500 MHz for ^{1}H and 125 MHz for ^{13}C, and a Bruker Avance 600 instrument operating at a frequency of 600 MHz for ^{1}H and 150 MHz for ^{13}C, a Bruker Avance 700 instrument operating at a frequency of 700 MHz for ^{1}H and 175 MHz for ^{13}C in $CDCl_3$, MeOD or DMSO-d_6 (as indicated below). The chemical shifts (δ) for ^{1}H and ^{13}C are quoted relative to residual signals of the solvent on the ppm scale. ^{1}H NMR peaks are reported as singlet (s), doublet (d), triplet (t), quartet (q), quintet (qn), m (multiplet), br (broad) and doublet of quartets (dq). Where amide rotamers are the case, unless stated otherwise, only the major rotamer has been assigned for chemical shifts, and areas underneath all rotameric peaks have been considered for integration calculations. Coupling constants (J values) are reported in Hertz (Hz) and are H-H coupling constants unless

otherwise stated. Where rotamer peaks are presented, analysis was conducted by integration of all rotamer peaks. Chemical shifts of only the major rotamer peaks are reported. Infrared spectra were obtained on a Perkin Elmer Spectrum 100 FTIR spectrometer operating in ATR mode. UV-Vis spectroscopy was used to determine PDs, peptides, proteins and peptide/protein conjugates concentrations using a NanoDrop OneC spectrophotometer (ThermoScientific) operating at 21 °C or 37 °C. Sample buffer was used as a blank for baseline correction.

Protein LC-MS

Molecular masses of proteins (<60K) were measured using Agilent 6530 QTOF LCMS (Agilent, UK). Agilent 1290 Infinity II UHPLC system was equipped with an Agilent PLRP-s, 1000 Å, 8 µm 50 mm × 2.1 mm column. 2 µL of protein sample (diluted to 0.2 mg/mL in LCMS grade water) was separated on the column using mobile phase A (water,0.1% formic acid) and B (acetonitrile, 0.1% formic acid) with an eluting gradient (shown below). Flow rate was at 0.8 mL/min and oven temperature was maintained at 60 °C.

LCMS mobile phase gradient for A/B elution:

Time (min)	Solvent A (%)	Solvent B (%)
0	80	20
1	80	20
6.5	40	60
7.5	40	60
7.6	80	20
8.5	80	20

Agilent 6530 QTOF mass spectrometer was operated in positive polarity mode, coupled with an ESI ion source. The ion source parameters were set up with a VCap of 4000 V, a gas temperature at 350 °C, a dry gas flow rate at 10 L/min and a nebulizer of 35 psig. MS TOF was acquired under conditions of a fragmentor at 175 V, a skimmer at 65 V and an acquisition rate at 1 spectra/s in a profile mode, within a scan range between 100 and 7000 m/z. The data were then analysed by deconvoluting a spectrum to a zero-charge mass spectrum using a maximum entropy deconvolution algorithm within the MassHunter software version B.07.00. Deconvoluted spectra were avoided where possible in the quantification of conjugates due to differing ionisation tendencies between species with significantly different masses.

LCMS analyses are shown as mentioned here: TIC LCMS trace (top), non-deconvoluted LC-MS trace (upper middle), wide range deconvoluted MS data (lower middle) and zoomed in deconvoluted data (bottom) for each species. It is acknowledged that the signal intensity of the mass spectra of

conjugates decreases as one goes through a reaction sequence - we believe this is due to the loss material over the sequence of steps.

SDS-PAGE

Non-reducing glycine-SDS-PAGE 12% acrylamide (10% for Fab) gels were performed following standard lab procedures. A 6% stacking gel was used and a broad-range molecular weight marker (10-250 kDa, Prestained PageRuler Plus Protein Standards, ThermoScientific) was run alongside the samples to estimate protein weights. Samples (10 µL at ~ 6 µM) were mixed with loading buffer (2 µL, composition for 5 × SDS: 1 g SDS, 3 mL glycerol, 6 mL 0.5 M Tri buffer pH 6.8, 2 mg bromophenol blue in 10 mL DI H_2O), heated at 80 °C for 5 min, and centrifuged at 10,000 RPM for 5 min. Samples were subsequently loaded into the wells in a volume of 6 µL. All gels were run at a constant current of 30 mA for 40 min using 1 × SDS running buffer. Gels were stained using a modified Coomassie stain (25 g ammonium sulfate, 250 mg Coomasie G-250, 8.8 mL 85% ortho-phosphoric acid, 50 mL ethanol, made up to a total of 250 mL with DI H_2O) at 21 °C for 16 h. The fluorescent bands were visualised using an AZURE 300®.

UV-Vis spectroscopy

UV-Vis spectroscopy UV-Vis spectroscopy was used to determine PDs and protein conjugates concentrations using a NanoDrop OneC spectrophotometer (ThermoScientific) operating at 21 °C. Sample buffer was used as a blank for baseline correction.

Protein	Extinction Coefficient ε_{280} (M^{-1} cm^{-1})
Trastuzumab Fab **25**	71000
Fab_{CD20} **152**	82905
Fab_{CD3} **157**	70000

Payload	Extinction Coefficient (M^{-1} cm^{-1})				Correction factor for 280 nm
	ε_{280}	ε_{335}	ε_{493}	ε_{578}	
Pyridazinedione scaffolds	2275	9100	-	-	0.25
5-FAM-PEG3-BCN (exo)	14774	-	83000	-	0.178
BP Fluor 568 DBCO	40480	-		88000	0.46

Corrected A_{280} = Experimental A_{280} – (A_{max} × Correction Factor)

Regioisomers for all lysine-modified Fab conjugates

For all lysine-modified Fab conjugates, lysines on the light or heavy chain may be modified; the below graphical representations have been simplified as it is difficult to depict all possible regioisomers graphically.

1.0 Synthesis and characterization of compounds

1.1 Synthesis of reagents **2a-2d**

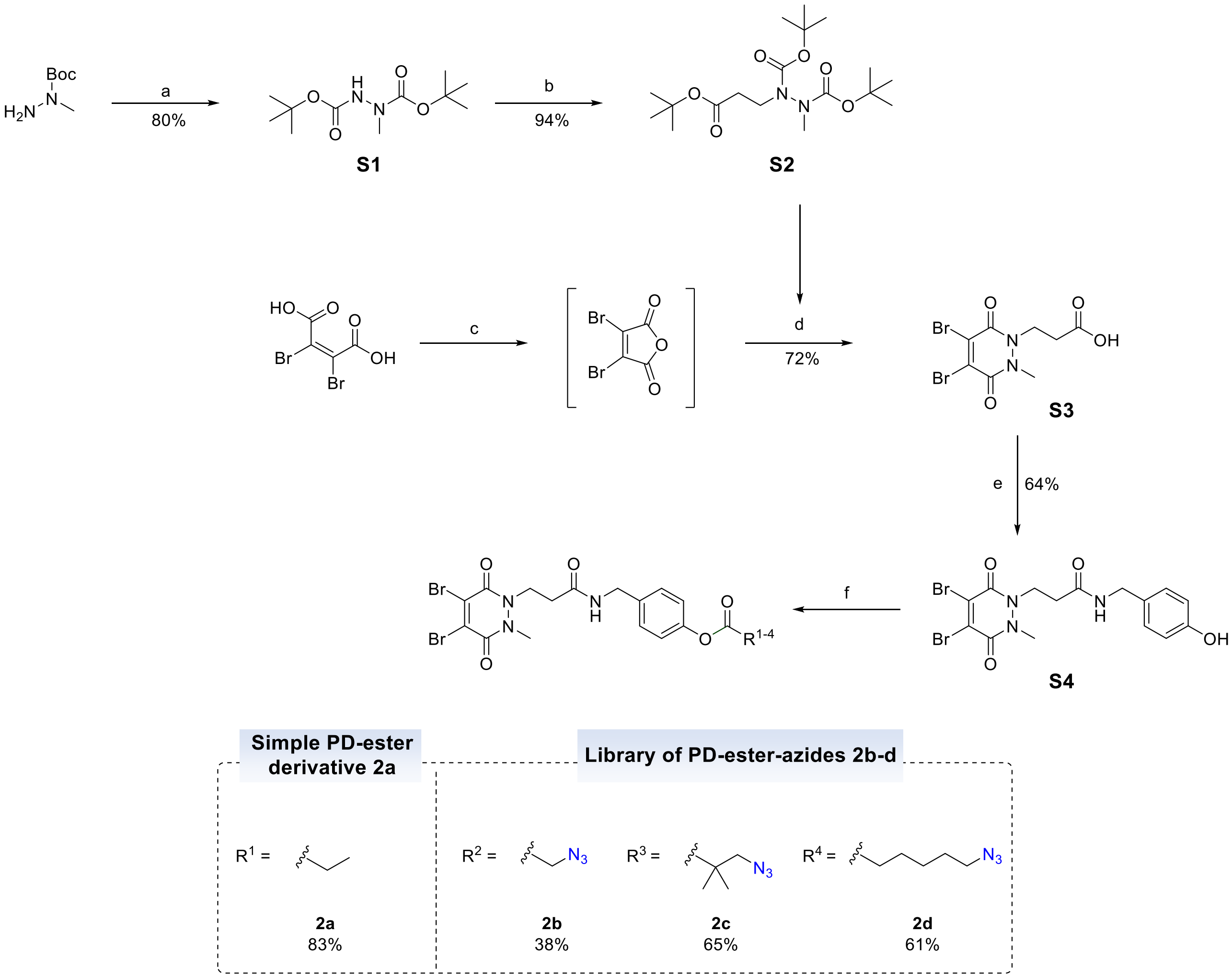

Scheme S1: Synthesis of reagents **2a-d**. Reagents and conditions: a) Boc_2O, Isopropanol, DCM, b) *tert*-butyl acrylate, *t*-BuOH, NaOH, reflux, c) AcOH, reflux, 30 mins, d) Reagent **S2**, AcOH, reflux, 6 h, e) 4-hydroxybenzylamine, NEt_3, DMF, 21°C, 4 h f) i) For **2a**: Propionic anhydride, DMAP, NEt_3, DMF, 21°C, 16 h, ii) For **2b-2d**: Respective azide containing acid, EDC·HCl, NEt_3, DMF, 21°C, 1-16 h.

Di-*tert*-butyl-1-methylhydrazine-1,2-dicarboxylate **S1**

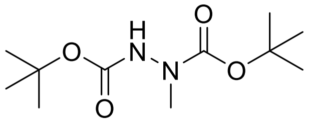

S1

Di-*tert*-butyl dicarbonate (10.00 g, 45.60 mmol) pre-dissolved in in CH_2Cl_2 (13 mL) was added over 30 min to a stirred solution of methyl hydrazine (0.96 mL, 18.20 mmol) in IPA (13 mL). The reaction was stirred at 21 °C for 16 h. The solvents were removed in vacuo and the crude residue was purified by flash column chromatography (0% to 20% EtOAc/Cyclohexane) to afford di-*tert*-butyl-1-methylhydrazine1,2-dicarboxylate **S1** (2.1 g, 14.2 mmol, 78%) as a white solid. **^{1}H NMR** (600 MHz, $CDCl_3$, rotamers) δ 6.37–6.10 (m, 1H), 3.11 (s, 3H), 1.47–1.46 (m, 18H). **^{13}C NMR** (150 MHz, $CDCl_3$, rotamers) δ 155.9 (C), 81.3 (C), 37.6 (CH_3), 28.3 (CH_3). **IR (thin film)**: 3316, 2980, 1701 cm^{-1}.

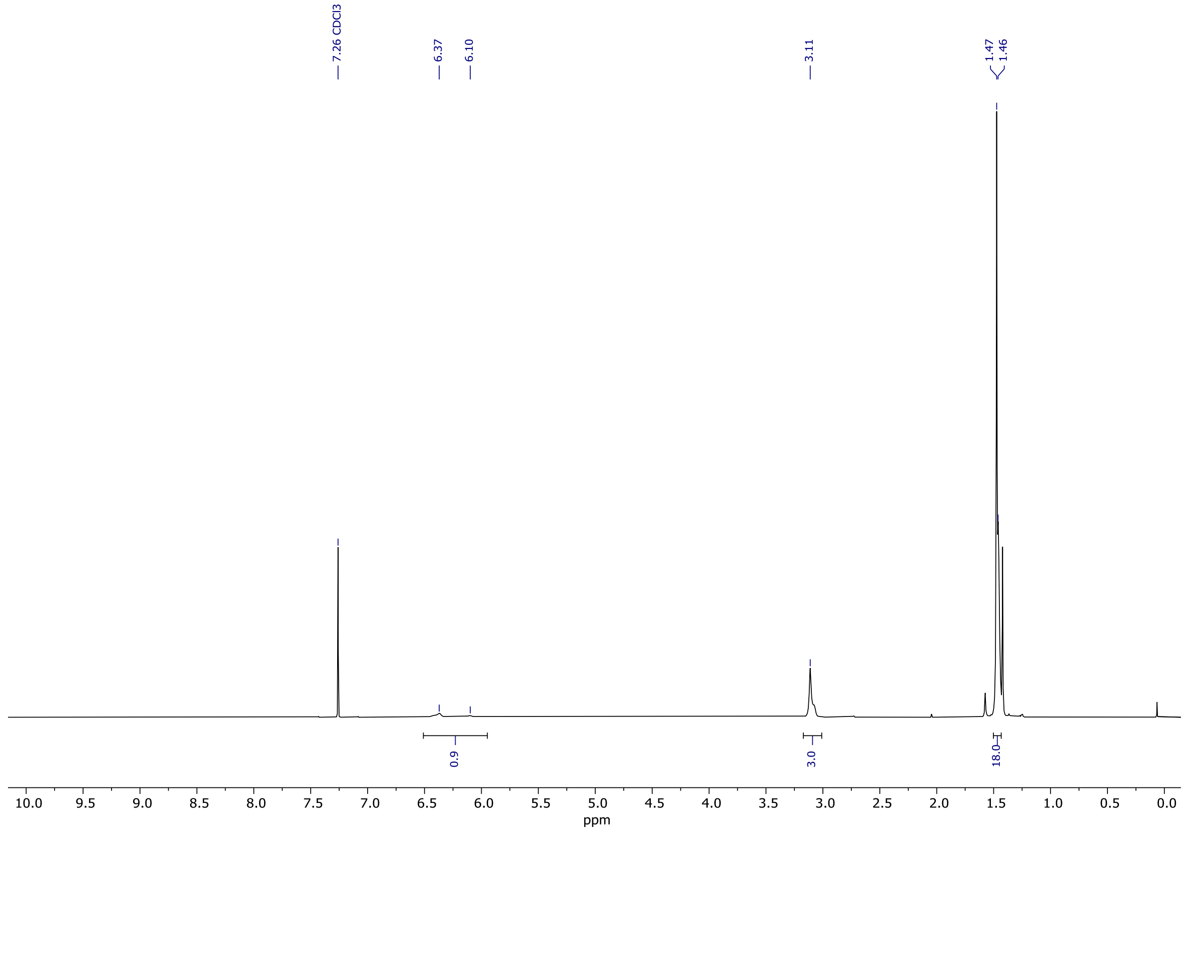

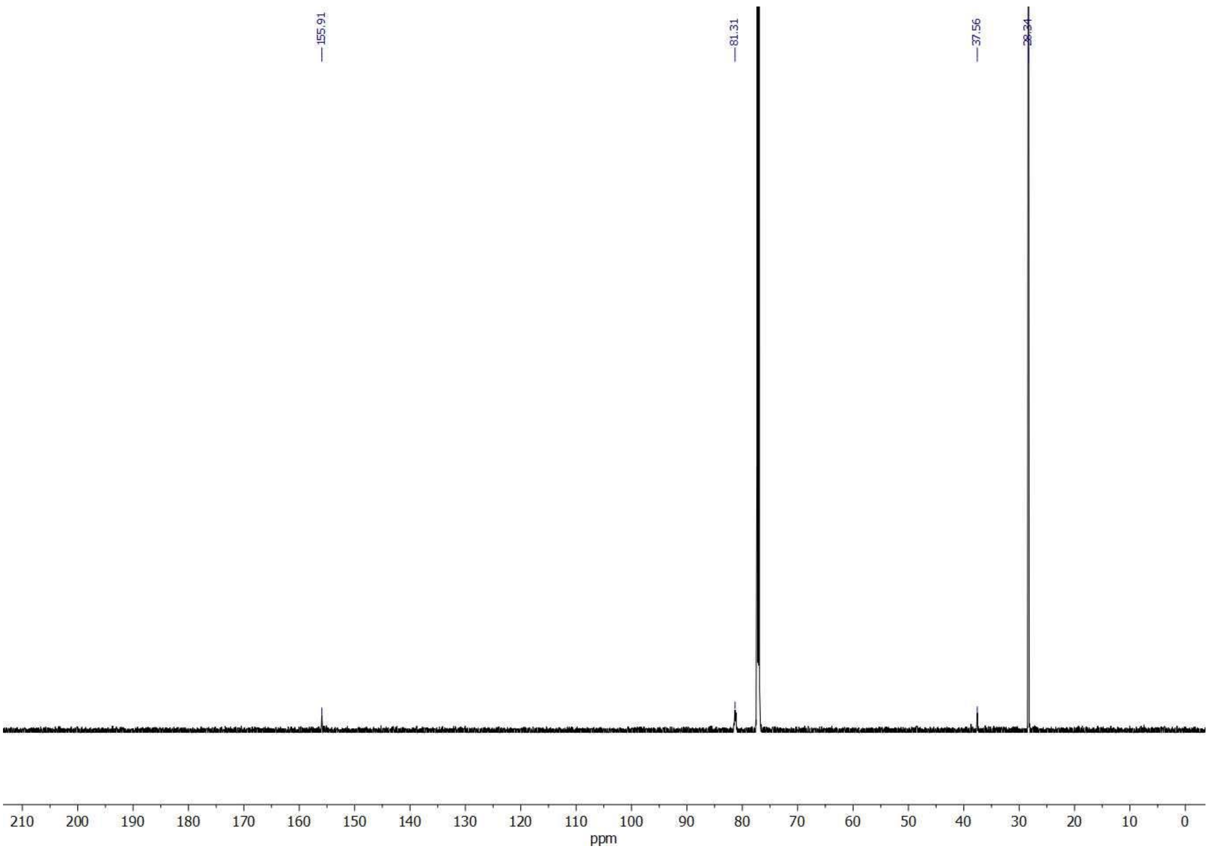

Figure S1**:** ^{1}H and ^{13}C NMR for reagent **S1**.

Di-*tert*-butyl-1-(3-(tert-butoxy)-3-oxopropyl)-2-methylhydrazine-1,2-dicarboxylate **S2**

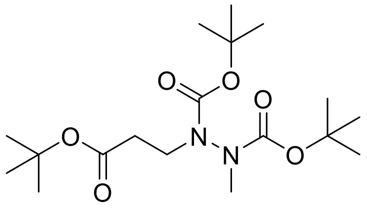

S2

To a solution of di-*tert*-butyl 1-methylhydrazine-1,2-dicarboxylate **S1** (2.00 g, 8.10 mmol) in *tert*-butanol (14 mL), was added 2 M NaOH (0.3 mL) and the reaction mixture stirred at 21 °C for 10 min. *Tert*-butyl acrylate (3.50 mL, 24.30 mmol) was added to the solution and the reaction mixture was heated under reflux for 72 h. The solvent was removed *in vacuo* and the residue dissolved in H_2O (50 mL) and extracted with EtOAc (3 × 50 mL). The combined organic phases were dried ($MgSO_4$), filtered and the solvent was removed *in vacuo* to afford di-*tert*-butyl-1-(3-(*tert*-butoxy)-3-oxopropyl)-2- methylhydrazine-1,2-dicarboxylate **S2** (2.90 g, 7.70 mmol, 94%) as a clear oil. ^{1}H NMR (600 MHz, $CDCl_3$, rotamers) δ 3.85–3.53 (m, 2H), 3.07–3.00 (m, 3H), 2.55-2.51 (m, 2H), 1.49–1.43 (m, 26H). ^{13}C NMR (150 MHz, $CDCl_3$, rotamers) δ 169.2 (C), 153.6 (C), 152.5 (C), 79.2 (C), 42.7 (CH_2), 34.8 (CH_3), 32.3 (CH_2), 26.5 (CH_3). **IR** (solid) 2977, 2934, 1708 cm^{-1}.

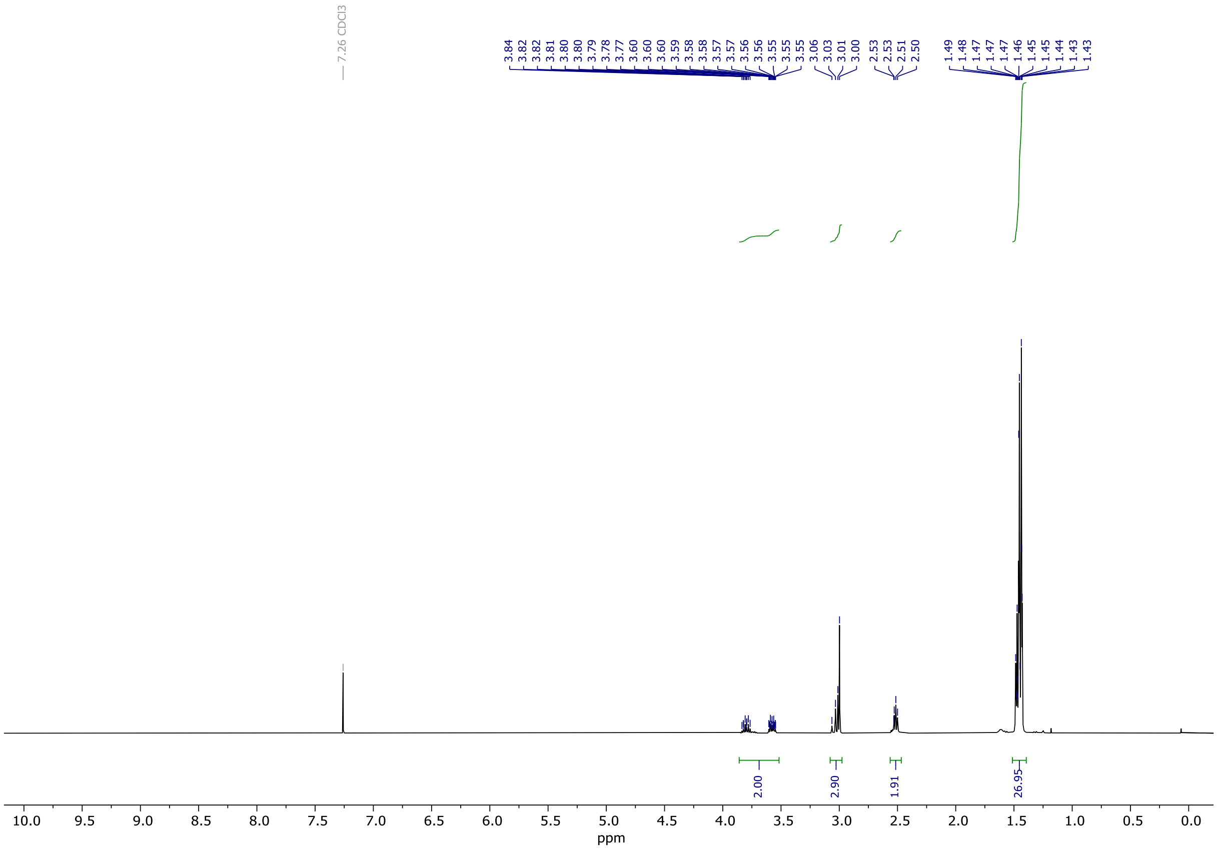

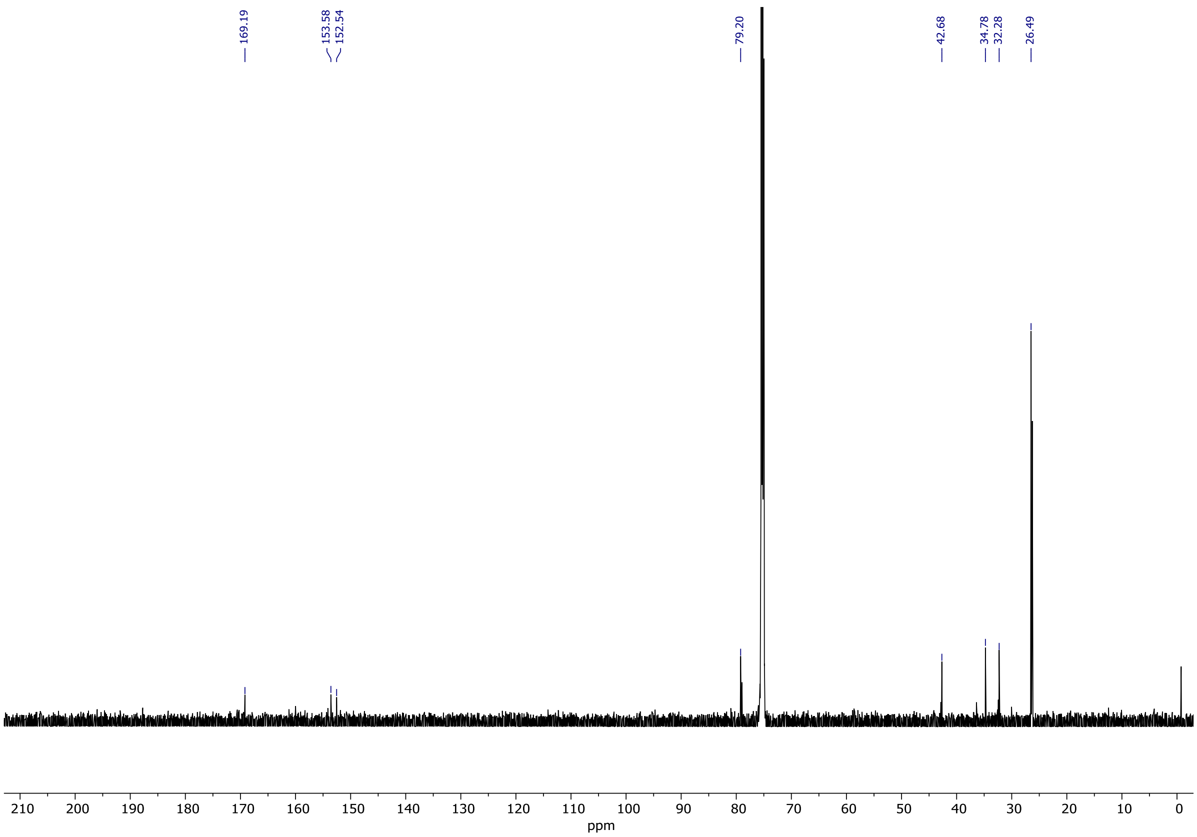

Figure S2: ^{1}H and ^{13}C NMR for reagent **S2**.

3-(4,5-Dibromo-2-methyl-3,6-dioxo-3,6-dihydropyridazin-1(2*H*)-yl) propanoic acid **S3**[1]

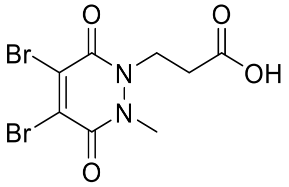

S3

Dibromomaleic acid (1.82 g, 6.70 mmol) was dissolved in AcOH (75 mL) and heated under reflux for 30 min. Di-*tert*-butyl-1-(3-(*tert*-butoxy)-3-oxopropyl)-2-methylhydrazine-1,2-dicarboxylate **S2** (3.00 g, 8.00 mmol) was added and the reaction was heated under reflux for a further 18 h. The solvent was removed *in vacuo* with toluene co-evaporation (3 × 30 mL, as an azeotrope). The crude was purified by flash column chromatography (0% to 70% EtOAc/ cyclohexane (1% AcOH)) to afford 3-(4,5-dibromo-2-methyl-3,6-dioxo-3,6-dihydropyridazin-1(2*H*)-yl) propanoic acid **S3** (1.70 g, 4.80 mmol, 72%) as a yellow solid. **^{1}H NMR** (600 MHz, MeOD) δ 4.41 (t, *J* = 7.3, 2H), 3.69 (s, 3H), 2.73 (t, *J* = 7.3, 2H). **^{13}C NMR** (150 MHz, MeOD) δ 173.8 (C), 154.7 (C), 154.5 (C), 136.7 (C), 136.4 (C), 44.9 (CH_3), 35.4 (CH_2), 32.5 (CH_2). **IR** (solid): 3045, 1726, 1607, 1571 cm^{-1}.

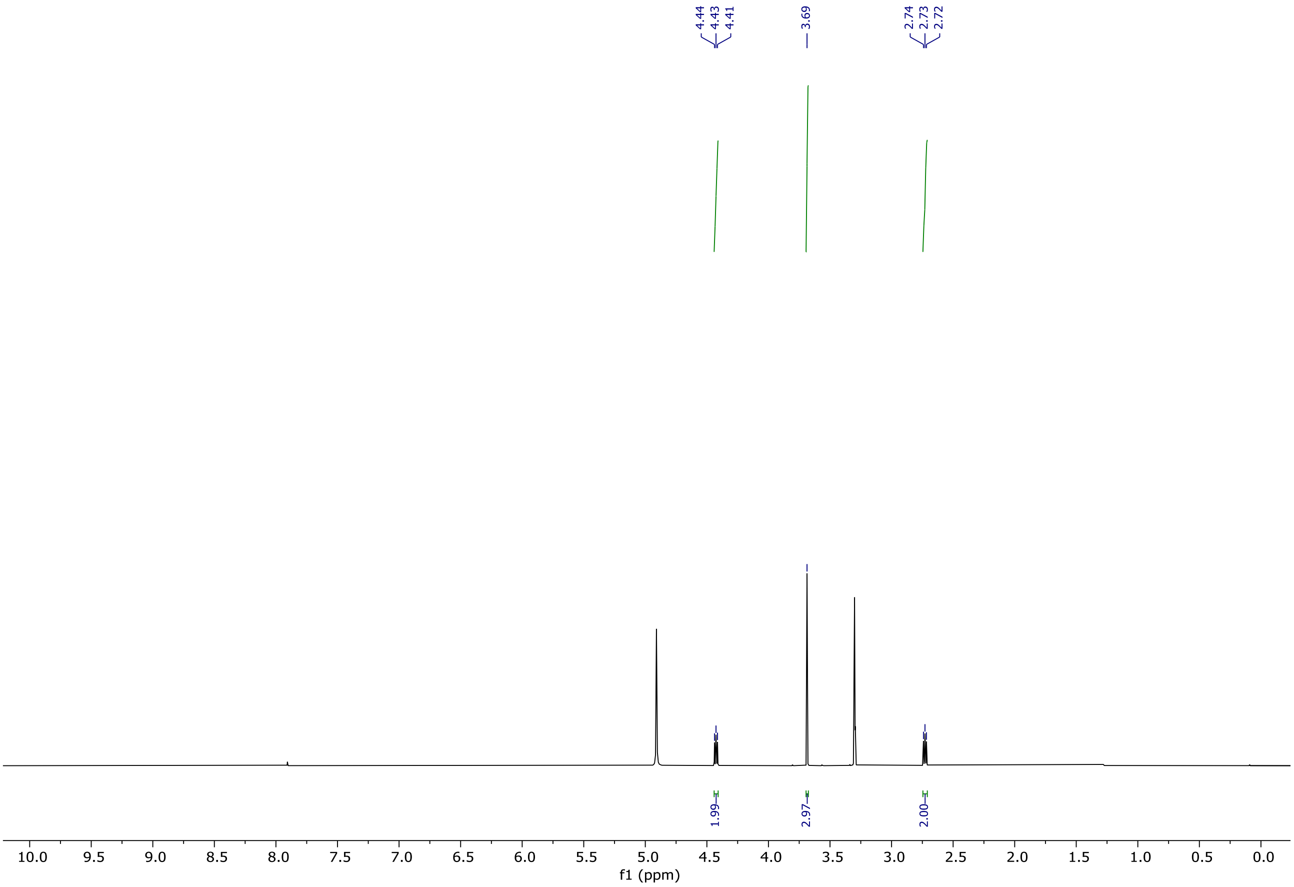

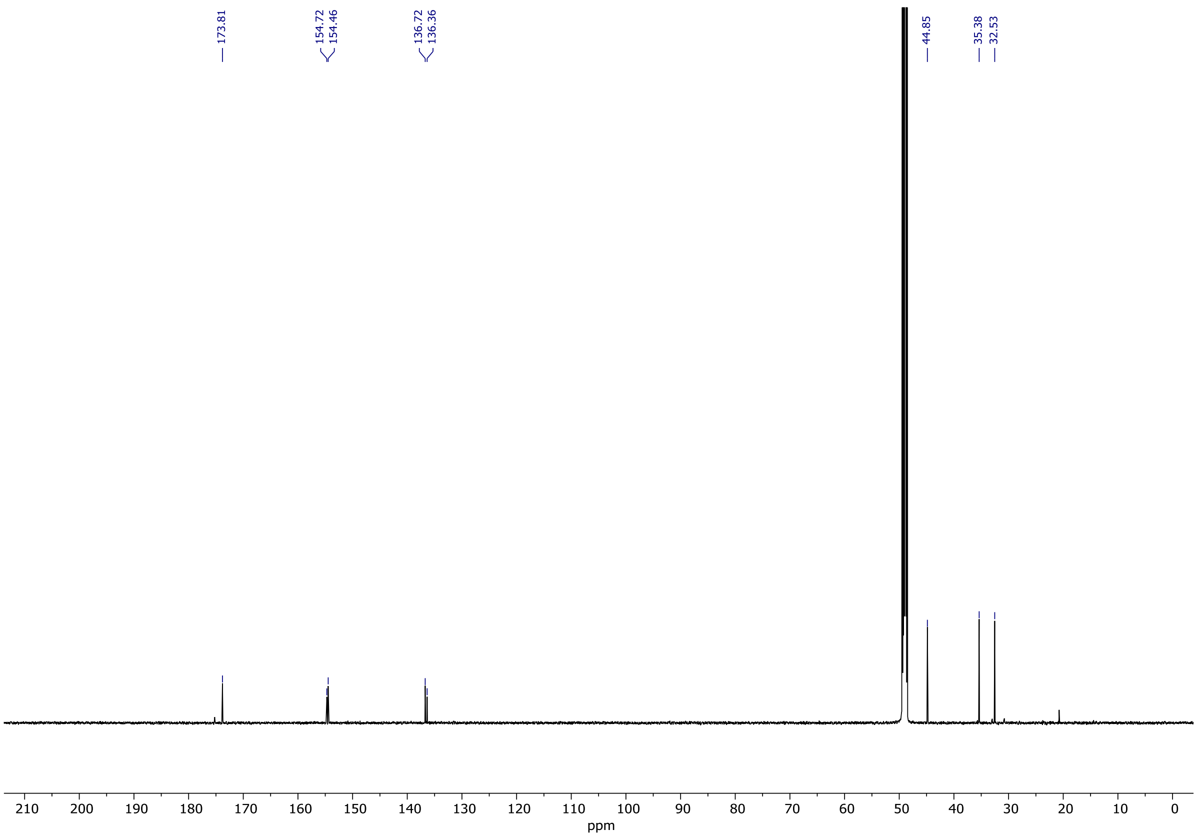

Figure S3: ^{1}H and ^{13}C NMR for reagent **S3**.

3-(4,5-Dibromo-2-methyl-3,6-dioxo-3,6-dihydropyridazin-1(2*H*)-yl)-*N*-(4-hydroxybenzyl)propanamide **S4**

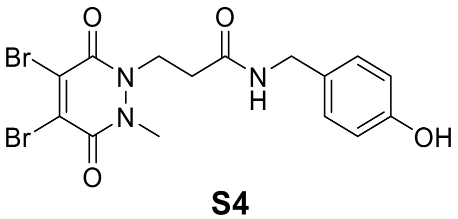

S4

To a solution of 3-(4,5-dibromo-2-methyl-3,6-dioxo-3,6-dihydropyridazin-1(2*H*)-yl) propanoic acid **S3** (1.00 g, 2.81 mmol) in DMF (10 mL) was added EDC·HCl (592 mg, 3.09 mmol). The reaction mixture was then stirred for 30 mins at 0 °C under argon. After this time, to this solution, was added dropwise a pre-mixed solution of 4-hydroxybenzylamine (381 mg, 3.09 mmol) and DIPEA (0.50 mL, 3.09 mmol) in anh. DMF (5 mL) under argon. After this, the reaction mixture was stirred at 21 °C for 4 h under argon. The reaction mixture was then concentrated *in vacuo* with toluene co-evaporation (3 × 30 mL, as an azeotrope). The crude residue was purified by flash column chromatography (0% to 20% EtOAc/MeOH) to afford 3-(4,5-dibromo-2-methyl-3,6-dioxo-3,6-dihydropyridazin-1(2*H*)-yl)-*N*-(4-hydroxybenzyl)propanamide **S4** (830 mg, 1.80 mmol, 64%) as a yellow solid. **^{1}H NMR** (500 MHz, CD_3CN) δ 7.06 (d, *J* = 7.9 Hz, 2H), 6.86 (s, 1H), 6.74 (d, *J* = 9.0 Hz, 2H), 4.34 (t, *J* = 7.0 Hz, 2H), 4.18 (d, *J* = 5.9 Hz, 3H), 3.57 (s, 2H), 2.51 (t, *J* = 7.1 Hz, 2H). **^{13}C NMR** (126 MHz, CD_3CN) δ 169.2 (C), 156.0 (C), 153.1 (C), 152.8 (C), 135.6 (C), 135.2 (C), 130.1 (C), 128.9 (CH), 115.1 (CH), 44.0 (CH_2), 42.3 (CH_2), 34.6 (CH_3), 33.4 (CH_2). **IR (solid)** 3323, 2921, 2852, 1756, 1634, 1195 cm^{-1}. **LRMS (ESI)** 464 (50, $[M^{81}Br^{81}Br+H]^+$), 462 (100, $[M^{79}Br^{81}Br+H]^+$), 460 (50, $[M^{79}Br^{79}Br+H]^+$); **HRMS (ESI)** calcd for $C_{15}H_{15}Br_2N_3O_4$ $[M^{79}Br^{81}Br+H]^+$ 461.9487; observed 461.9482.

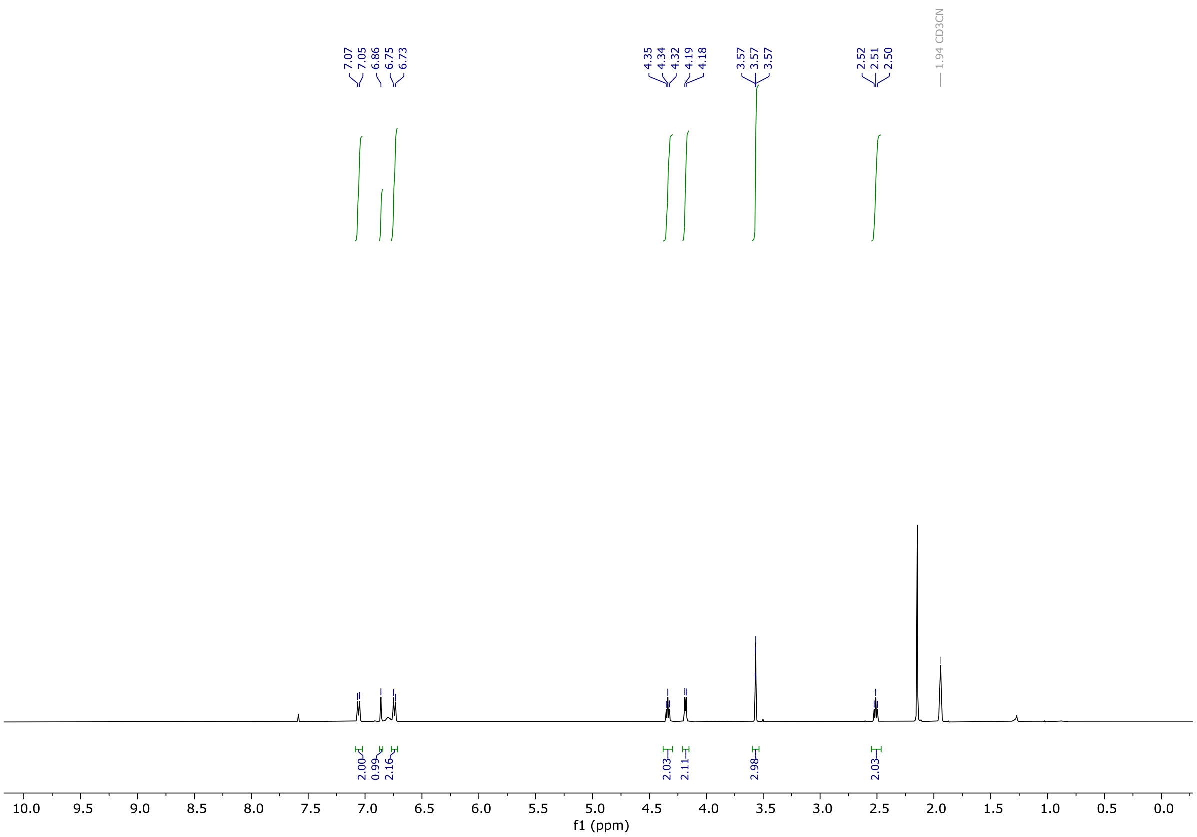

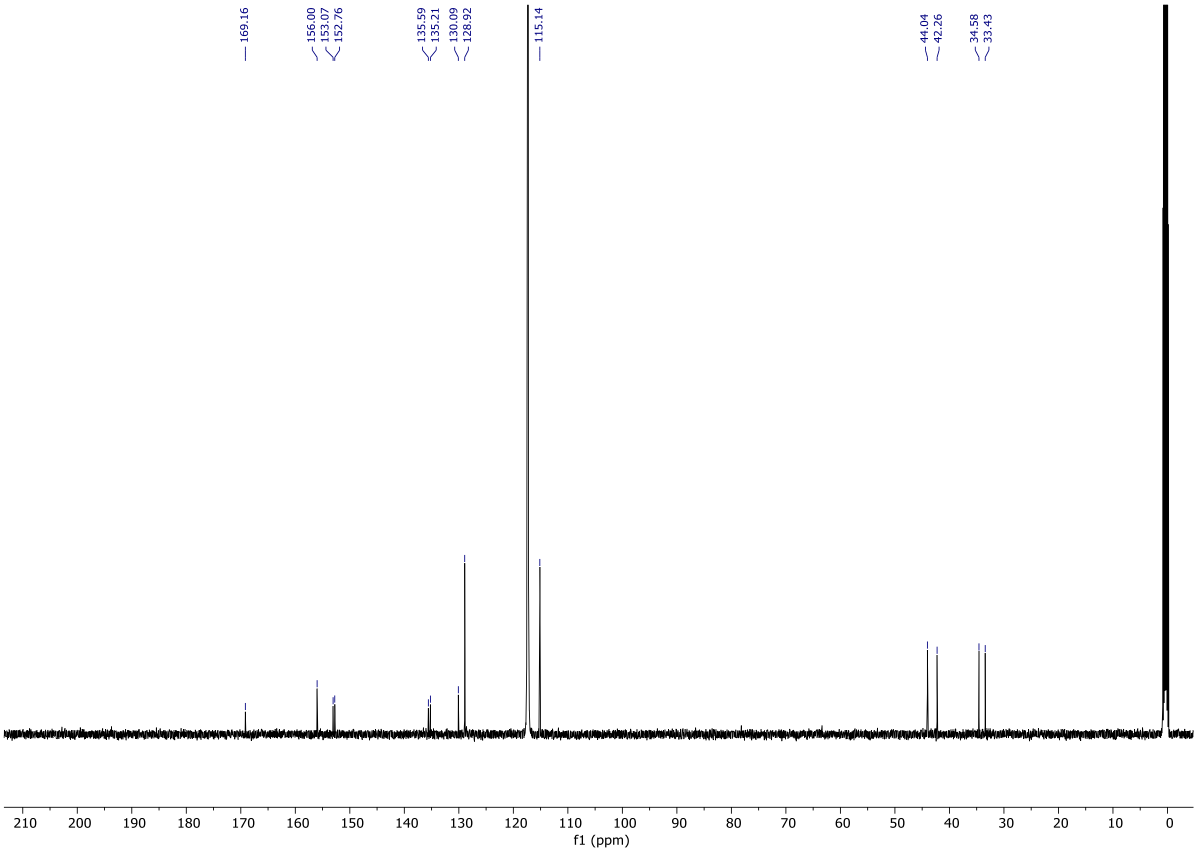

Figure S4: ^{1}H and ^{13}C NMR for reagent **S4**.

4-((3-(4,5-Dibromo-2-methyl-3,6-dioxo-3,6-dihydropyridazin-1(2*H*)-yl)propanamido)methyl)phenyl propionate **2a**

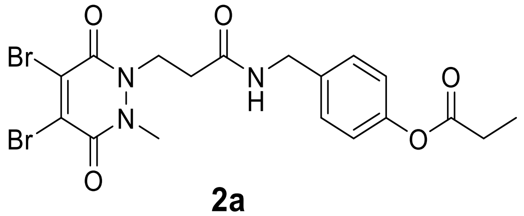

2a

To a solution of 3-(4,5-dibromo-2-methyl-3,6-dioxo-3,6-dihydropyridazin-1(2*H*)-yl)-*N*-(4-hydroxybenzyl)propenamide **S4** (150 mg, 0.33 mmol) in DMF (5 mL) was added DMAP (8 mg, 0.07 mmol) and NEt_3 (50 µL, 0.36 mmol). Solution was stirred at 0 °C for 10 mins under argon. Propionic anhydride (47 mg, 0.36 mmol) was added to the solution and was left to stir for 6 h under argon. After this time, the solvent was removed *in vacuo* with toluene co-evaporation (3 × 30 mL, as an azeotrope) and the crude residue was purified by flash column chromatography (0% to 20% DCM/MeOH) to afford 4-((3-(4,5-Dibromo-2-methyl-3,6-dioxo-3,6-dihydropyridazin-1(2*H*)-yl)propanamido)methyl)phenyl propionate **2a** (141 mg, 0.27 mmol, 83%) as a white solid. **^{1}H NMR** (500 MHz, MeOD) δ 7.27 (d, *J* = 8.5 Hz, 2H), 7.04 (d, *J* = 8.5 Hz, 2H), 4.46 (t, *J* = 6.8 Hz, 2H), 4.31 (s, 2H), 2.73 – 2.51 (m, 4H), 1.22 (t, *J* = 7.5 Hz, 2H). **^{13}C NMR (**126 MHz, MeOD) δ 174.6 (C), 172.0 (C), 154.7 (C), 154.4 (C), 151.5 (C), 137.2 (C), 136.7 (C), 136.4 (C), 129.8 (2 × CH), 122.9 (2 × CH), 45.4 (CH_2), 43.7 (CH_2), 35.5 (CH_3), 34.6 (CH_2), 28.3 (CH_2), 9.3 (CH_3). **IR** (solid): 3310, 2920, 2850, 1752, 1631, 1190 cm^{-1}.

LRMS (ESI) 520 (50, $[M^{81}Br^{81}Br+H]^+$), 518 (100, $[M^{79}Br^{81}Br+H]^+$), 516 (50, $[M^{79}Br^{79}Br+H]^+$; **HRMS (ESI)** calcd for $C_{18}H_{19}Br_2N_3O_5$ $[M^{79}Br^{81}Br+H]^+$ 517.9749; observed 517.9728.

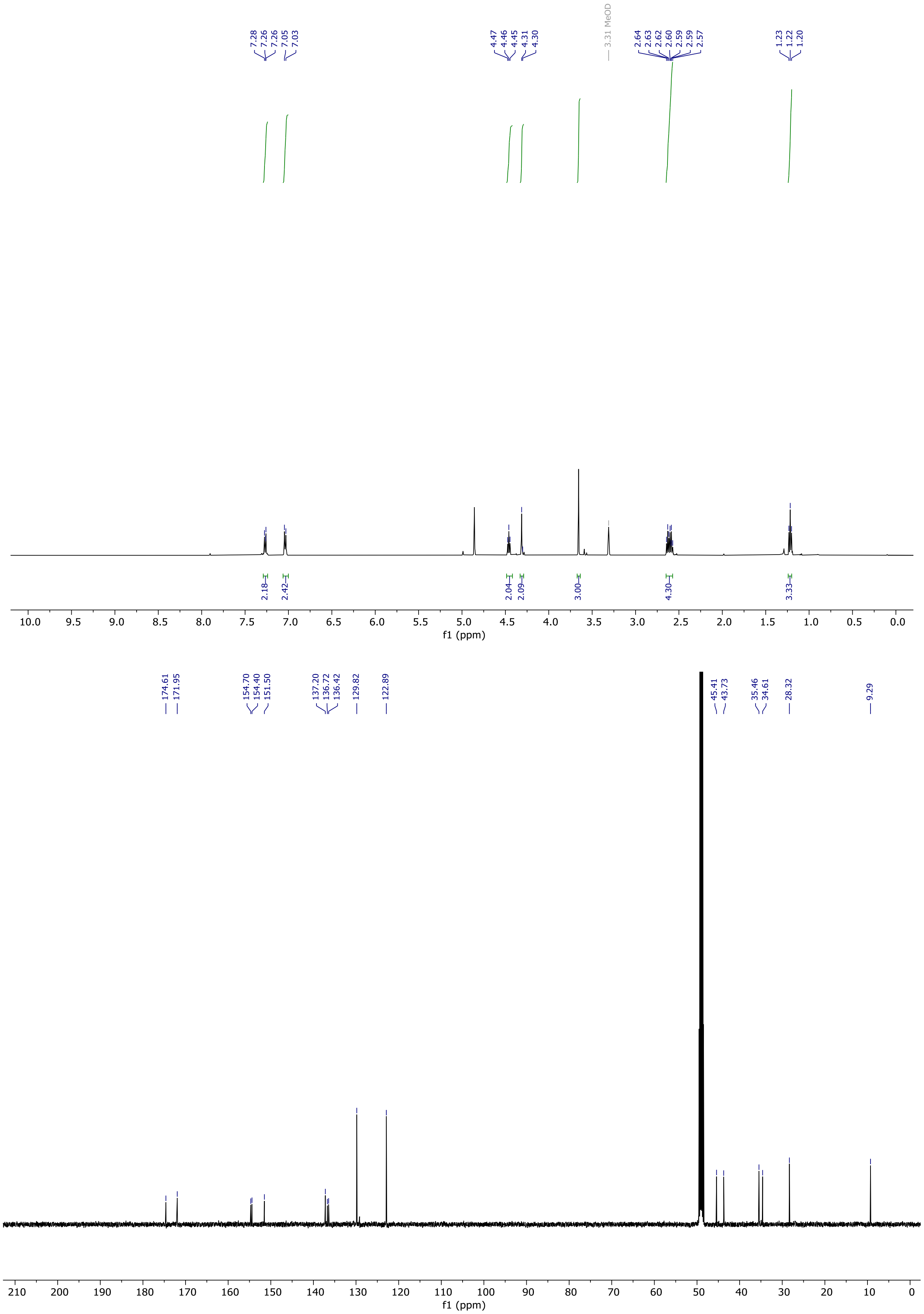

Figure S5: ^{1}H and ^{13}C NMR for reagent **2a**.

4-((3-(4,5-Dibromo-2-methyl-3,6-dioxo-3,6-dihydropyridazin-1(2*H*)-yl)propanamido)methyl)phenyl 2-azidoacetate **2b**

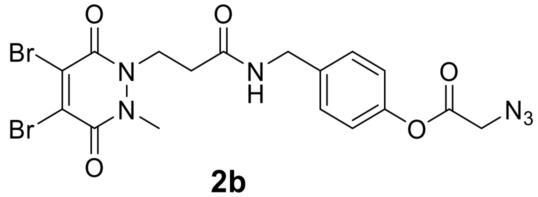

2b

To a solution of 2-azidoacetic acid (150 mg, 1.48 mmol) in anh. DMF (3 mL) was added EDC·HCl (312 mg, 1.63 mmol).The reaction mixture was stirred at 0 °C for 30 mins under argon. After this time, to this solution, was added dropwise a pre-mixed solution of 3-(4,5-dibromo-2-methyl-3,6-dioxo-3,6-dihydropyridazin-1(2*H*)-yl)-*N*-(4-hydroxybenzyl)propenamide **S4** (228 mg, 0.45 mmol) and DIPEA (94 µL, 69 mg, 0.54 mmol) in anh. DMF (2 mL). After this, the reaction mixture was stirred at 21 °C for 16 h under argon. The solvent mixture was then concentrated *in vacuo* with toluene co-evaporation (3 × 30 mL, as an azeotrope) and the crude residue purified by flash column chromatography (0% to 30% DMC/MeOH) to afford 4-((3-(4,5-dibromo-2-methyl-3,6-dioxo-3,6-dihydropyridazin-1(2*H*)-yl)propanamido)methyl)phenyl 2-azidoacetate **2b** (90 mg, 0.17 mmol, 38%) as a yellow oil. **^{1}H NMR** (700 MHz, MeOD) δ 7.90 (s, 1H), 7.30 (d, *J* = 8.4 Hz, 2H), 7.12 (d, *J* = 8.4 Hz, 2H), 4.47 (t, *J* = 7.0 Hz, 2H), 4.33 (s, 2H), 4.24 (s, 2H), 3.66 (s, 3H), 2.64 (t, *J* = 7.0 Hz, 2H). **^{13}C NMR** (176 MHz, MeOD) δ 171.9 (CO), 169.0 (CO), 154.6 (C), 154.3 (C), 150. 9 (C), 137.7 (C), 136.6 (2×CH), 136.3 (2×CH), 129.8 (CH), 122.6 (CH), 51.0 (CH_2), 45.3 (CH_2), 43.6 (CH_2), 35.3 (CH_3), 34.5 (CH_2). **IR** (thin film): 3308, 2916, 2849, 2108, 1764, 1626, 1166 cm^{-1}. **LRMS (ESI)** 547 (50, $[M^{81}Br^{81}Br+H]^{+}$), 545 (100, $[M^{79}Br^{81}Br+H]^{+}$), 543 (50, $[M^{79}Br^{79}Br+H]^{+}$); **HRMS (ESI)** calcd for $C_{17}H_{16}Br_2N_6O_5$ $[M^{79}Br^{81}Br+H]^{+}$ 544.9607; observed 544.96012 .

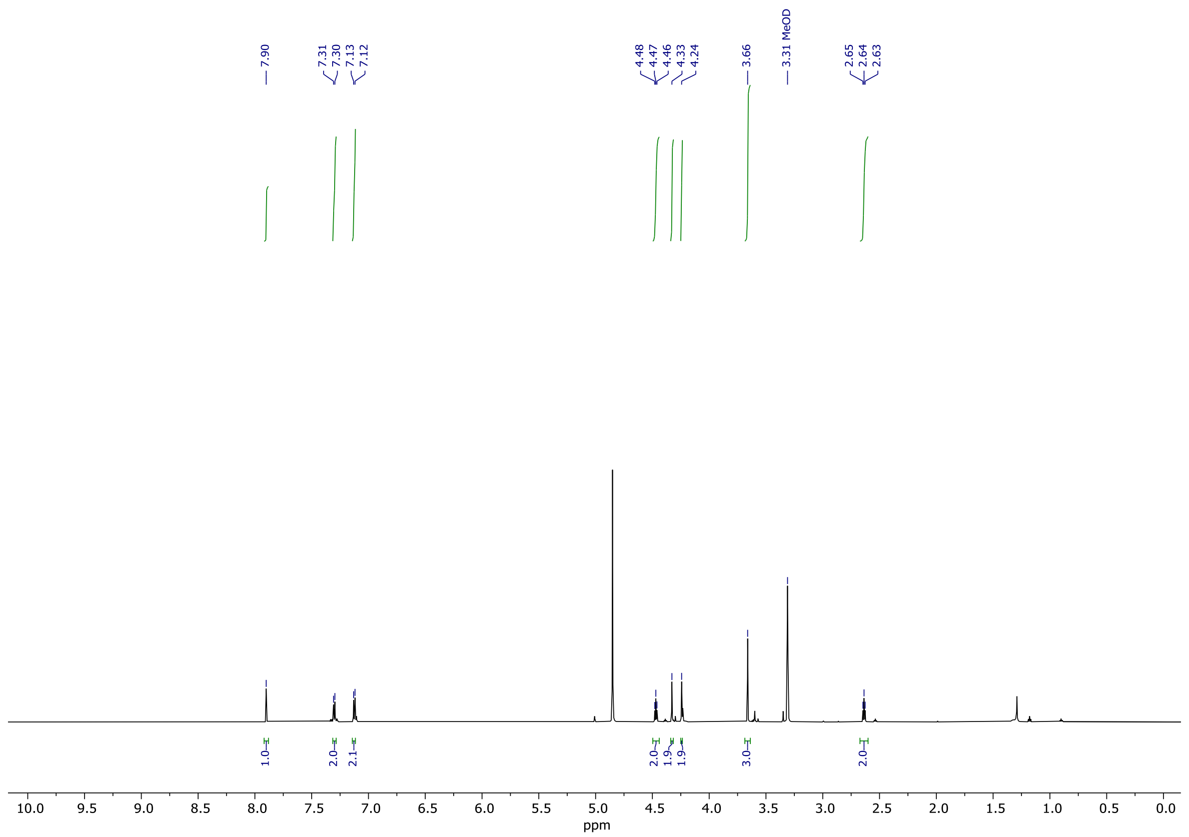

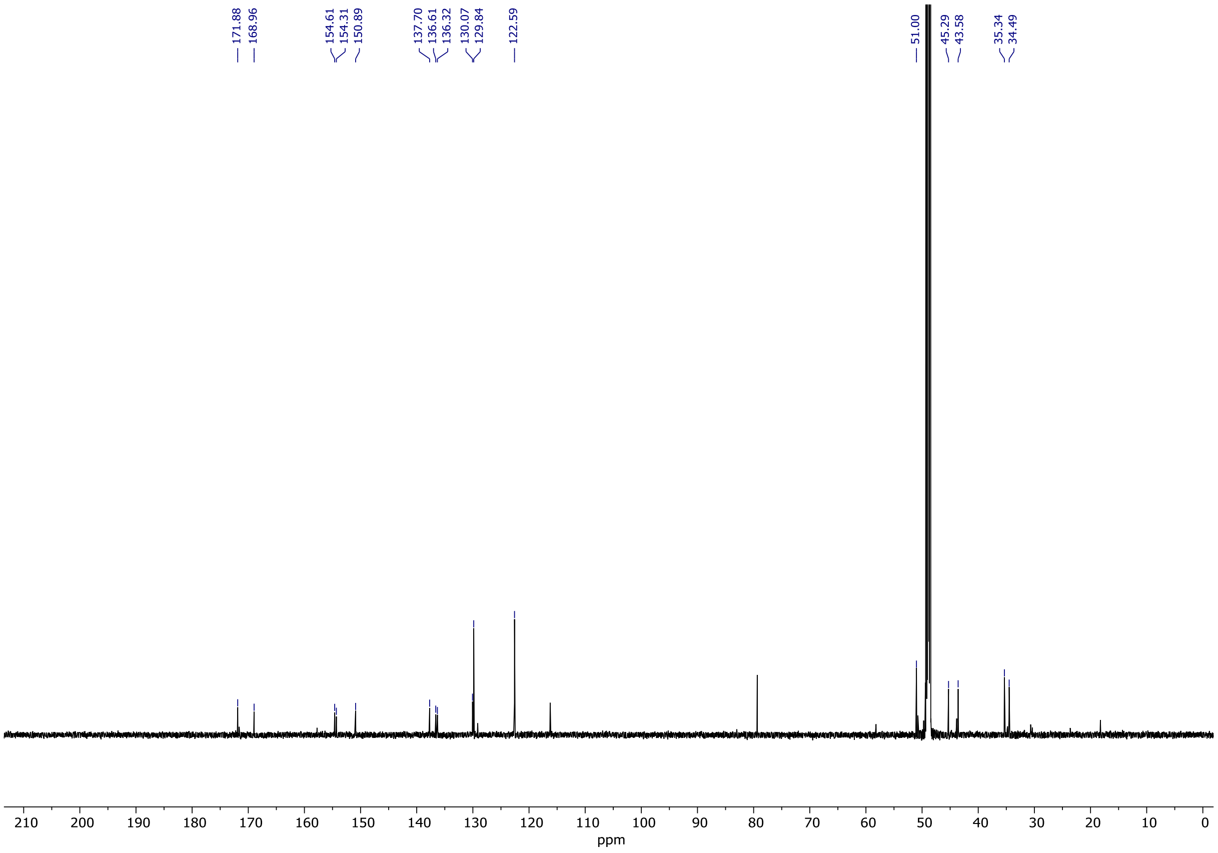

Figure S6: ^{1}H and ^{13}C NMR for reagent **2b**.

3-Azido-2,2-dimethylpropanoic acid **S5**[2]

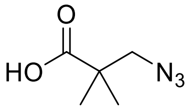

S5

3-Chloro-2,2-dimethylpropanoic acid (1.00 g, 7.32 mmol) and sodium azide (2.38 g, 36.6 mmol) in water (40 mL) were refluxed for 23 h. After this time, to the reaction mixture was added conc. HCl (12 mL). The product was then extracted into DCM (4 × 30 mL), and the combined organic layers washed with brine (1 × 30 mL), dried ($MgSO_4$) and concentrated *in vacuo* to afford 3-azido-2,2-dimethylpropanoic acid **S5** (965 mg, 6.74 mmol, 92%) as a brown oil. **^{1}H NMR** (400 MHz, $CDCl_3$) δ 3.44 (s, 2H), 1.26 (s, 6H). **^{13}C NMR** (101 MHz, $CDCl_3$) δ 182.0 (C), 59.3 (CH_3), 43.6 (C), 23.0 (2×CH_3).

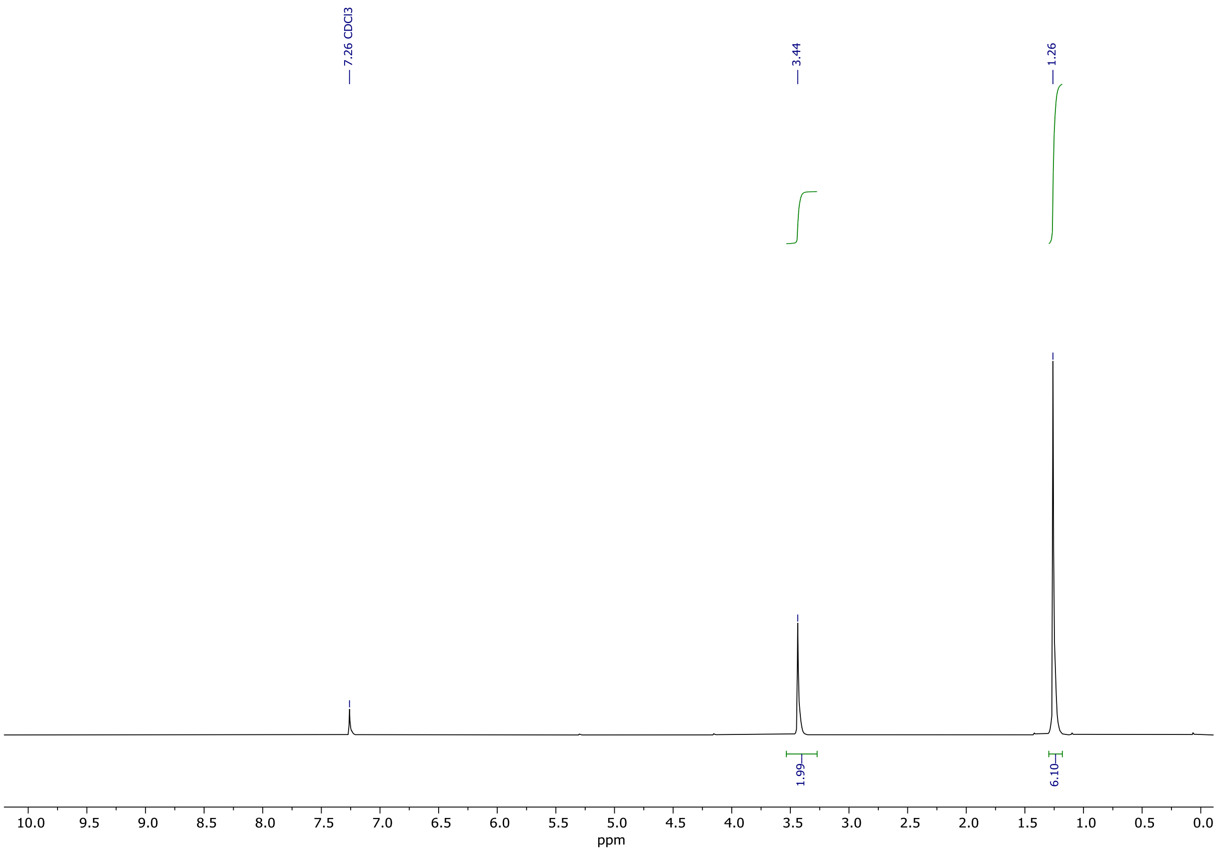

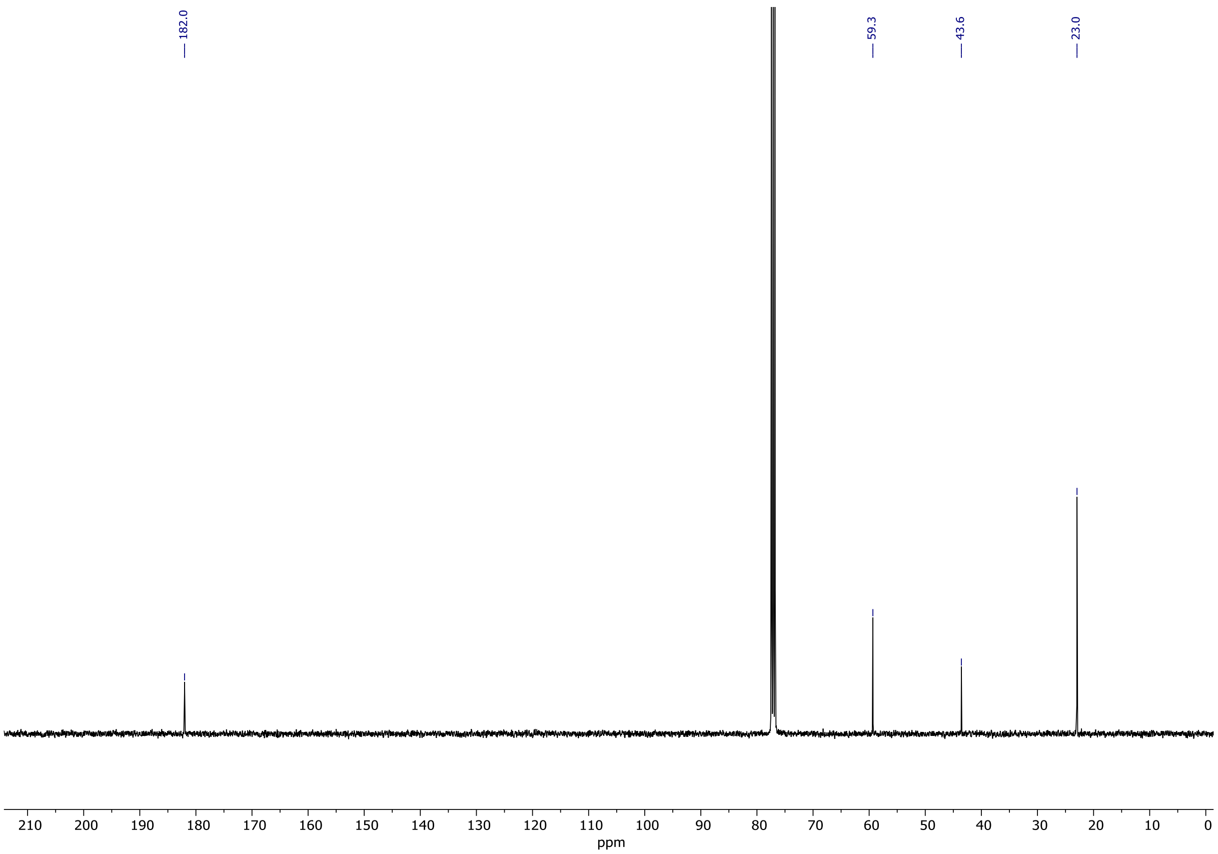

Figure S7: ^{1}H and ^{13}C NMR for reagent **S5**.

4-((3-(4,5-Dibromo-2-methyl-3,6-dioxo-3,6-dihydropyridazin-1(2*H*)-yl)propanamido)methyl)phenyl 3-azido-2,2-dimethylpropanoate **2c**

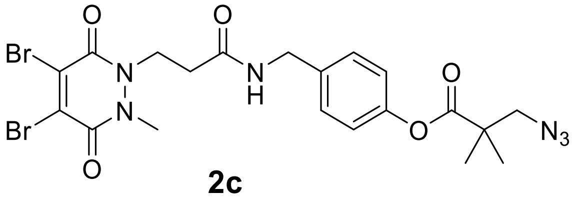

2c

To a solution of 3-azido-2,2-dimethylpropanoic acid **S5** (78 mg, 0.55 mmol) in anh. DMF (3 mL) was added EDC·HCl (127 mg, 0.66 mmol). The reaction mixture was then stirred at 0 °C for 30 min under argon. After this time, to this solution, was added dropwise a pre-mixed solution of 3-(4,5-dibromo-2-methyl-3,6-dioxo-3,6-dihydropyridazin-1(2*H*)-yl)-*N*-(4-hydroxybenzyl)propenamide **S4** (100 mg, 0.22 mmol) and NEt_3 (46 µL, 0.33 mmol) in anh. DMF (3 mL). After this, the reaction mixture was stirred at 21 °C for 1 h under argon. The solvent mixture was then concentrated *in vacuo* with toluene co-evaporation (3 × 30 mL, as an azeotrope) and the crude residue purified by flash column chromatography (0% to 30% DMC/MeOH) to afford 4-((3-(4,5-dibromo-2-methyl-3,6-dioxo-3,6-dihydropyridazin-1(2*H*)-yl)propanamido)methyl)phenyl 2-azidoacetate **2c** (84 mg, 0.143 mmol, 65%) as a colourless oil. **^{1}H NMR** (500 MHz, CD_3CN) δ 7.27 (d, *J* = 8.4 Hz, 2H), 7.02 (d, *J* = 8.4 Hz, 2H), 4.34 (t, *J* = 6.9 Hz, 2H), 4.29 (d, *J* = 6.0 Hz, 2H), 3.58 (s, 2H), 3.57 (s, 3H), 2.55 (t, *J* = 6.9 Hz, 2H), 1.33 (s, 6H). **^{13}C NMR** (126 MHz, CD_3CN) δ 175.6 (C), 170.4 (C), 154.1 (C), 153.8 (C), 150.9 (C), 137.9 (C), 136.6 (C), 136.2 (C), 129.6 (2×CH), 122.6 (2×CH), 60.3 (CH_2), 45.0 (CH_2), 44.8 (C), 43.2 (CH_2), 35.6 (CH_3), 34.4 (CH_2), 23.2 (2×CH_3). **IR** (thin film): 3308, 2925, 2854, 2103, 1749, 1631, 1195, 1165, 1115 cm^{-1}. **LRMS (ESI)** 585 (50, $[M^{81}Br^{81}Br+H]^+$), 587 (100, $[M^{79}Br^{81}Br+H]^+$), 585 (50, $[M^{79}Br^{79}Br+H]^+$); **HRMS (ESI)** calcd for $C_{20}H_{22}Br_2N_6O_5$ $[M^{79}Br^{81}Br+H]^+$ 587.0076; observed 587.0071.

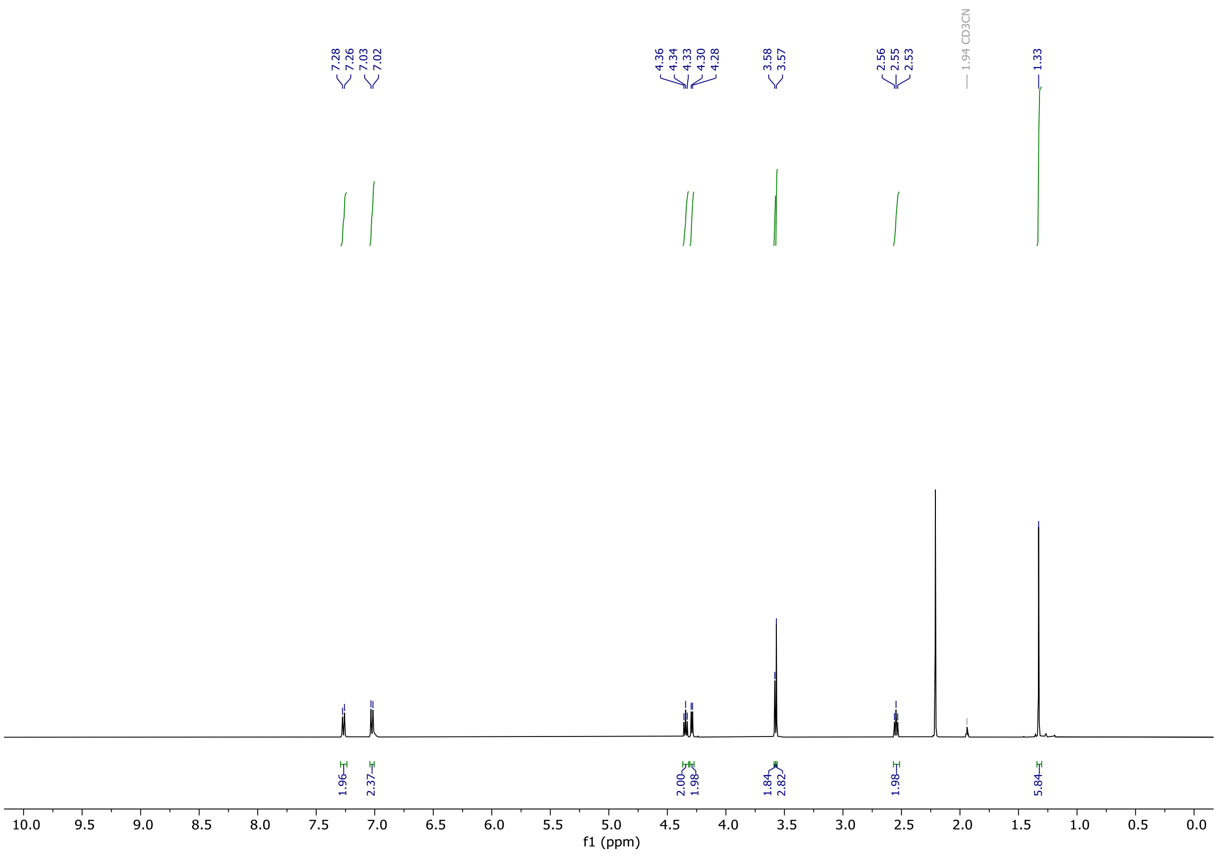

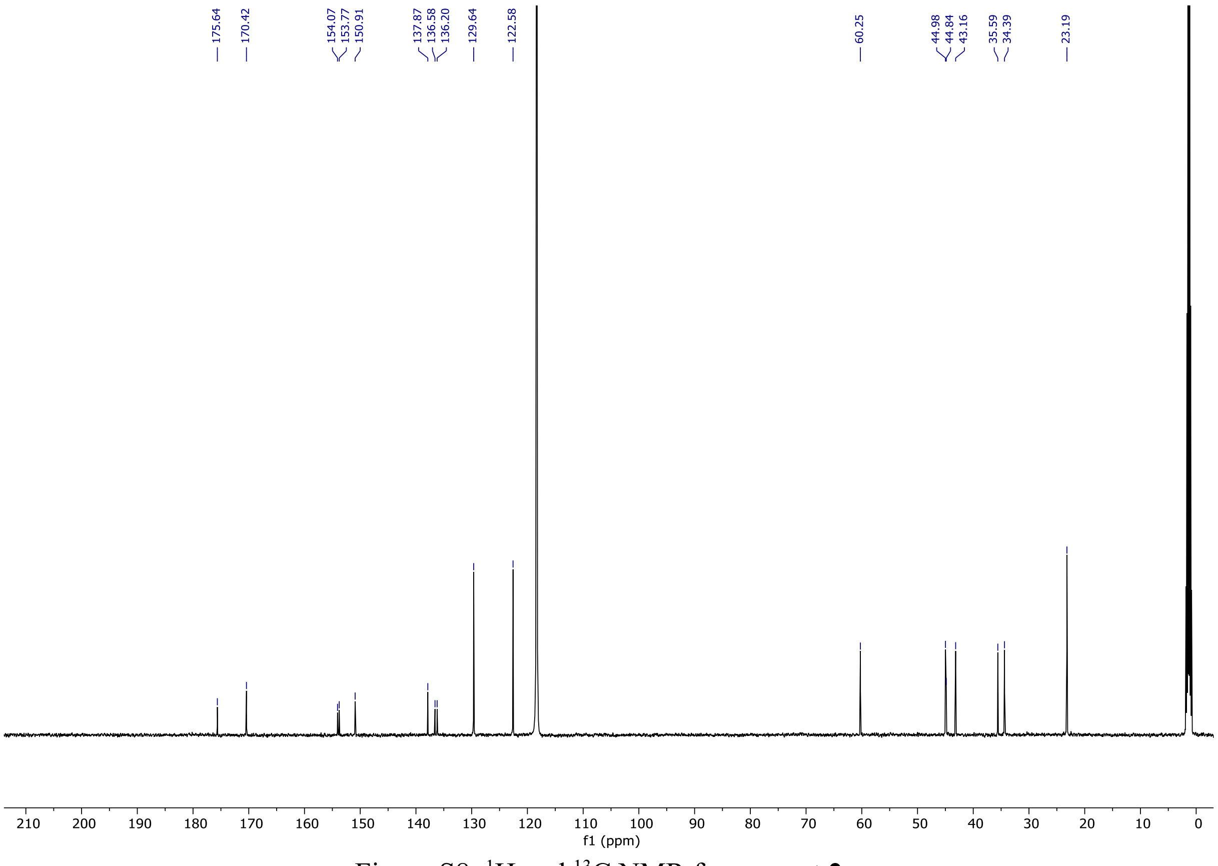

Figure S8: ^{1}H and ^{13}C NMR for reagent **2c**.

6-Azidohexanoic acid **S6**[3]

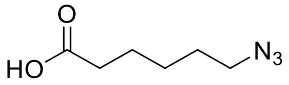

S6

To a solution of 6-bromohexanoic acid (3.00 g, 15.4 mmol) in 1:1 acetone: H_2O (3 mL:3 mL) was added sodium azide (1.24g, 19.0 mmol). The solution was heated to 40 °C and left to stir for 16 h. After this time, the crude product was extracted into EtOAc (3 × 15 mL). The combined organic layers were washed with H_2O (10 mL), sat. NaCl (10 mL), dried ($MgSO_4$) and concentrated *in vacuo* to afford 6-azidohexanoic acid **S6** (1.35 g, 8.59 mmol, 91%) as a colourless oil. **^{1}H NMR** (600 MHz, $CDCl_3$) δ 3.28 (t, J = 6.9 Hz, 2H), 2.38 (t, J = 7.4 Hz, 2H), 1.72 – 1.54 (m, 4H), 1.50 – 1.39 (m, 2H). **^{13}C NMR** (151 MHz, CDCl3) δ 179.2 (C), 51.3 (CH_2), 33.8 (CH_2), 28.6 (CH_2), 26.2 (CH_2), 24.2 (CH_2). **IR** (thin film): 2939, 2867, 2092, 1704, 1256 cm^{-1}.

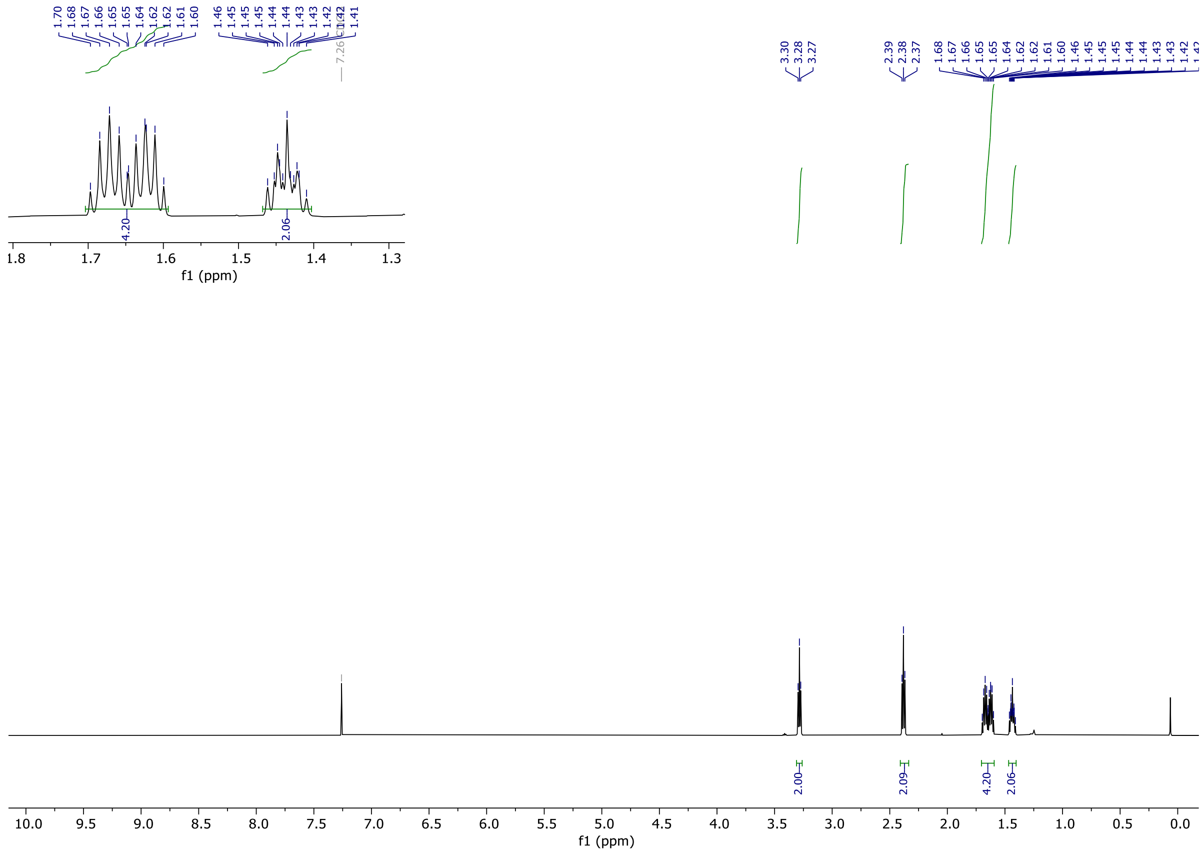

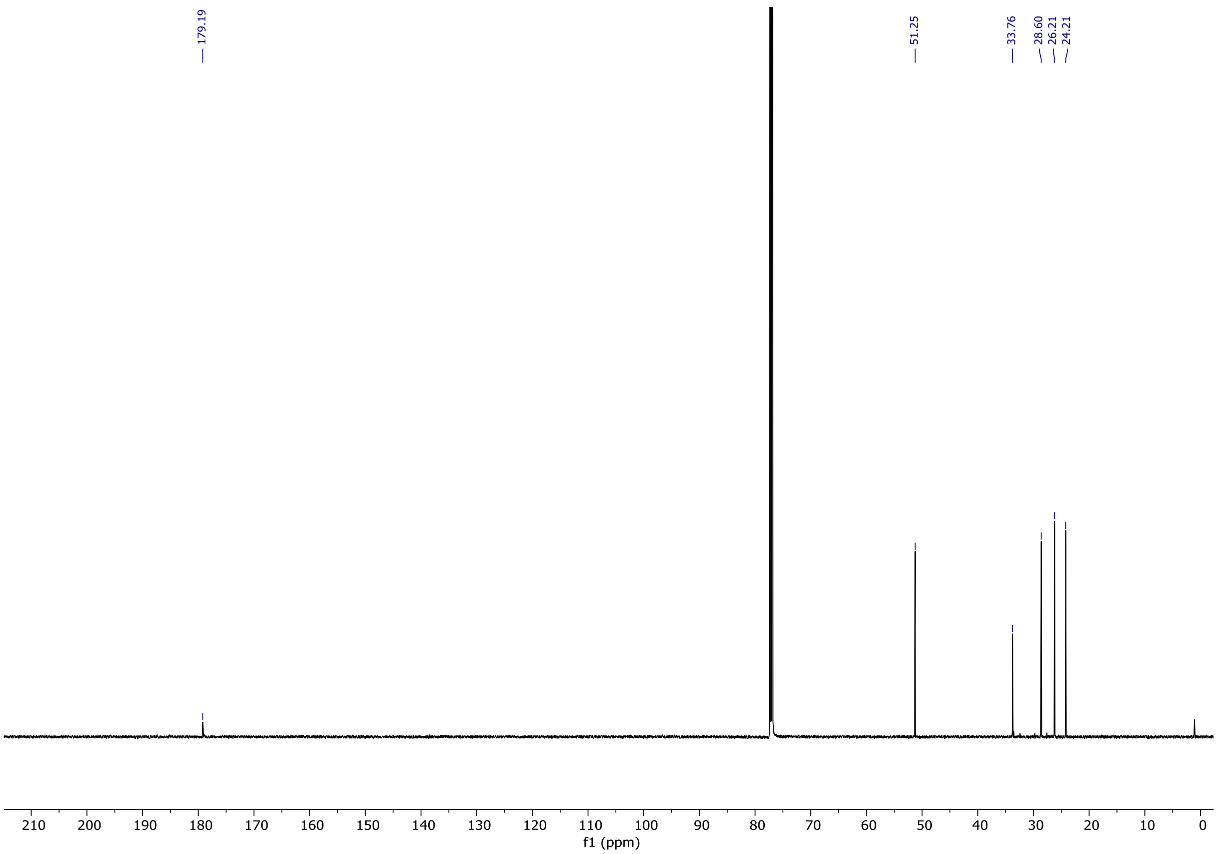

Figure S9: ^{1}H and ^{13}C NMR for reagent **S6**.

4-((3-(4,5-Dibromo-2-methyl-3,6-dioxo-3,6-dihydropyridazin-1(2*H*)-yl)propanamido)methyl)phenyl 6-azidohexanoate **2d**

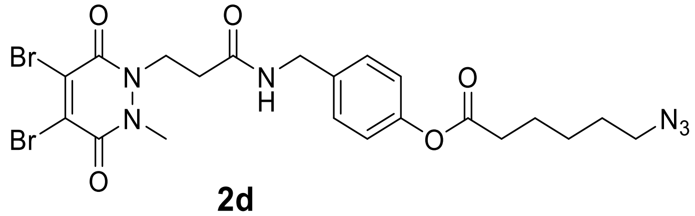

2d

To a solution of 6-azidohexanoic acid **S6** (69 mg, 0.44 mmol) in anh. DMF (4 mL) was added EDC·HCl (127 mg, 0.66 mmol) and DMAP (5 mg, 0.04 mmol). The reaction mixture was then stirred for 30 mins at 0 °C under argon. After this time, to this solution, was added dropwise a pre-mixed solution of 3-(4,5-dibromo-2-methyl-3,6-dioxo-3,6-dihydropyridazin-1(2H)-yl)-N-(4-hydroxybenzyl)propenamid **S4** (80 mg, 0.13 mmol) and NEt_3 (46 µL, 0.33 mmol) in anh. DMF (4 mL). After this, the reaction mixture was stirred at 21 °C for 16 h under argon. The reaction mixture was then concentrated *in vacuo* with toluene co-evaporation (3 × 30 mL, as an azeotrope). The crude residue was purified by flash column chromatography (0% to 20% MeOH/EtOAc) to afford 2,6-dibromo-4-((3-(1-(15-(4,5-dibromo-2-methyl-3,6-dioxo-3,6-dihydropyridazin-1(2*H*)-yl)-13-oxo-3,6,9-trioxa-12-azapentadecyl)-1*H*-1,2,3-triazol-4-yl)propanamido)methyl)phenyl 6-azidohexanoate **2d** (62 mg, 0.13 mmol, 52%) as a yellow oil. **^{1}H NMR** (500 MHz, $CDCl_3$) δ 7.25 (d, J = 8.3 Hz, 2H), 7.01 (d, J = 8.3 Hz, 2H), 4.34 (t, J = 6.9 Hz, 2H), 4.28 (d, J = 6.0 Hz, 2H), 3.56 (s, 3H), 3.32 (t, J = 6.9 Hz,21H), 2.58–2.33 (m, 4H), 1.71 (p, J = 7.5 Hz, 2H), 1.63 (p, J = 7.1 Hz, 2H), 1.49–1.43 (m, 2H). **^{13}C NMR** (126 MHz, CD_3CN) δ 172.2, 169.4, 153.1, 152.8, 149.9, 136.7, 135.6, 135.2, 128.6 (2×CH), 121.8 (2×CH), 51.9 (CH_2), 44.0 (CH_2), 42.2 (CH_2), 34.6 (CH_3), 33.6 (CH_2), 33.4 (CH_2), 28.2 (CH_2), 25.9 (CH_2), 24.1 (CH_2). **IR** (thin film): 3326, 2927, 2864, 2095, 1753, 1630 cm^{-1}. **LRMS (ESI)** (50, $[M^{81}Br^{81}Br+H]^+$), (100, $[M^{79}Br^{81}Br+H]^+$), (50, $[M^{79}Br^{79}Br+H]^+$); **HRMS (ESI)** calcd for $[M^{79}Br^{81}Br+H]^+$; observed.

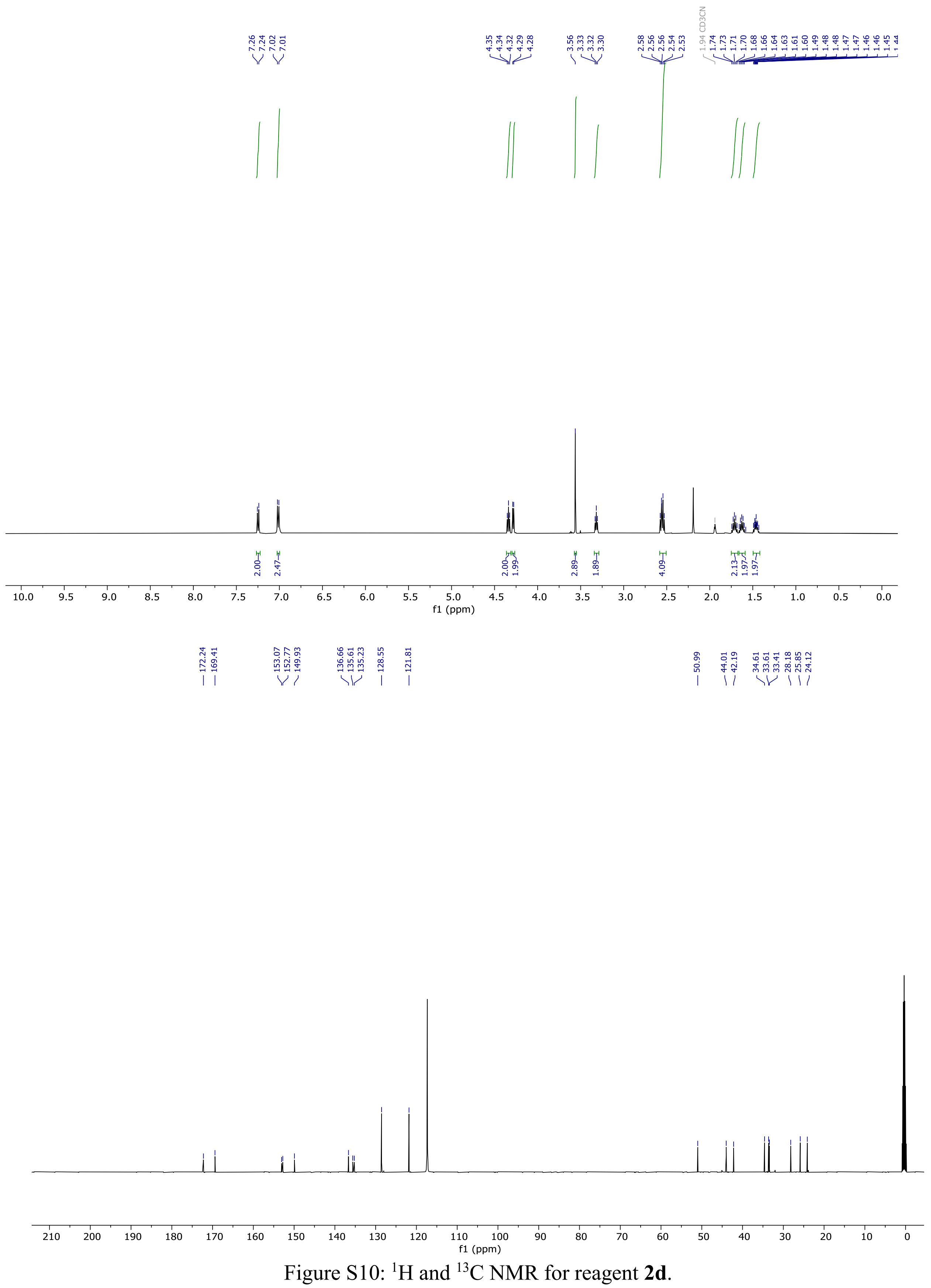

Figure S10: ^{1}H and ^{13}C NMR for reagent **2d**.

1.2 Synthesis of reagents **4**, **5** and **6**

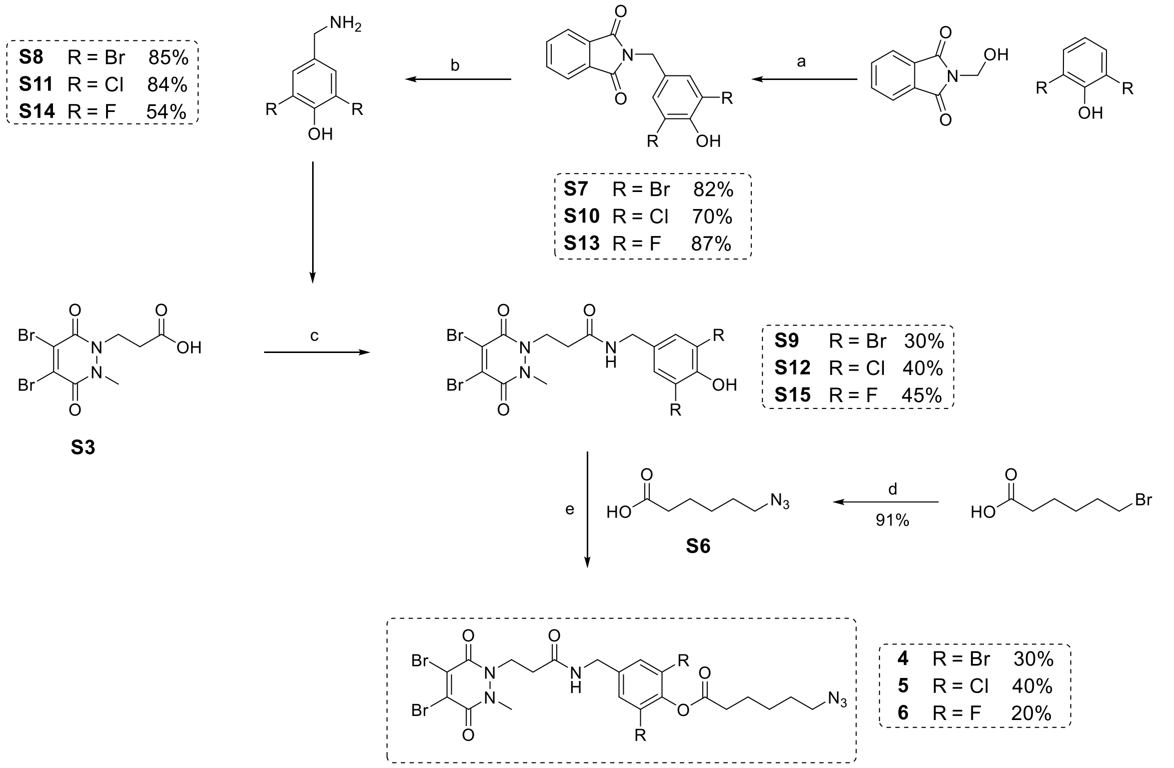

Scheme S2: a) H_2SO_4, THF, rt, 1 h, b) hydrazine, HCl, MeOH, reflux, 16 h, c) EDC·HCl, NEt_3, anh. DMF, 21°C, 4 h, d) NaN_3, Acetone:H_2O, 16 h, e) EDC·HCl, DMAP, NEt_3, anh. DMF, 21°C, 16 h.

2-(3,5-Dibromo-4-hydroxybenzyl)isoindoline-1,3-dione **S7**[2]

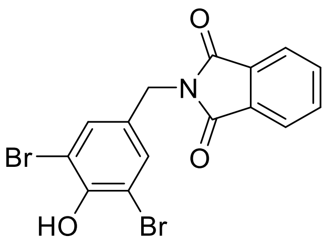

S7

To a solution of 2,6-dibromophenol (3.00 g, 11.95 mmol) in THF (50 mL) was added *N*-(hydroxymethyl)phthalimide (2.32 g, 13.10 mmol) at 0 °C. H_2SO_4 (30 mL) was slowly added, and reaction mixture was stirred at 0 °C for 5 mins and then at 21 °C for 16 h. After this time, EtOAc (50 mL) and H_2O (50 mL) were added to the solution and the resulting white precipitate was filtered and washed with EtOAc and H_2O to afford 2-(3,5-dibromo-4-hydroxybenzyl)isoindoline-1,3-dione **S7** (4.00 g, 9.73 mmol, 82%) as a white solid. **^{1}H NMR** (400 MHz, DMSO) δ 9.96 (s, 1H), 7.97 – 7.71 (m, 4H), 7.50 (s, 2H), 4.67 (s, 2H). **^{13}C NMR** (101 MHz, DMSO) δ 167.7 (C), 150.1 (C), 134.6 (CH), 131.7 (C), 131.6 (CH), 131.1 (C), 123.3 (CH), 111.9 (C), 39.3 (CH_2). **IR** (solid): 3326, 1763, 1704, 1429, 1142, 1103, 724, 709 cm^{-1}.

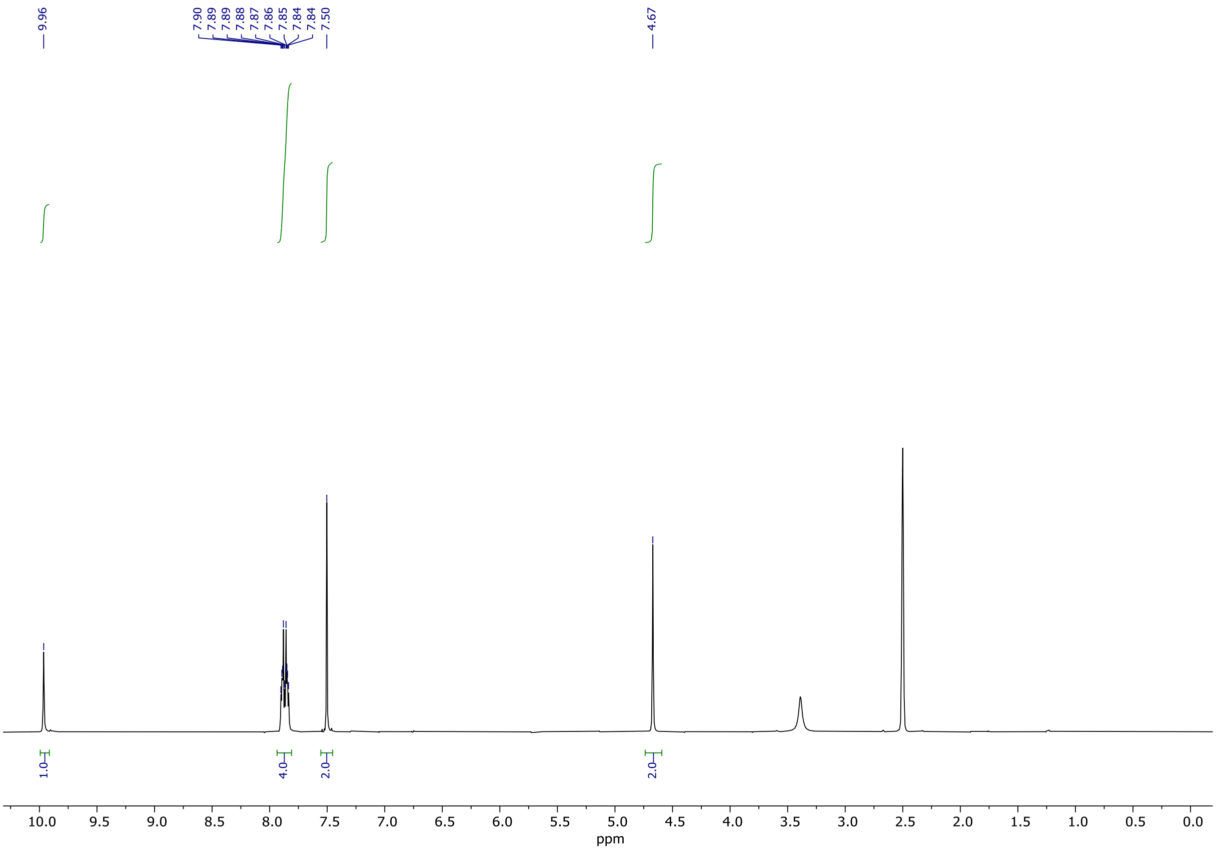

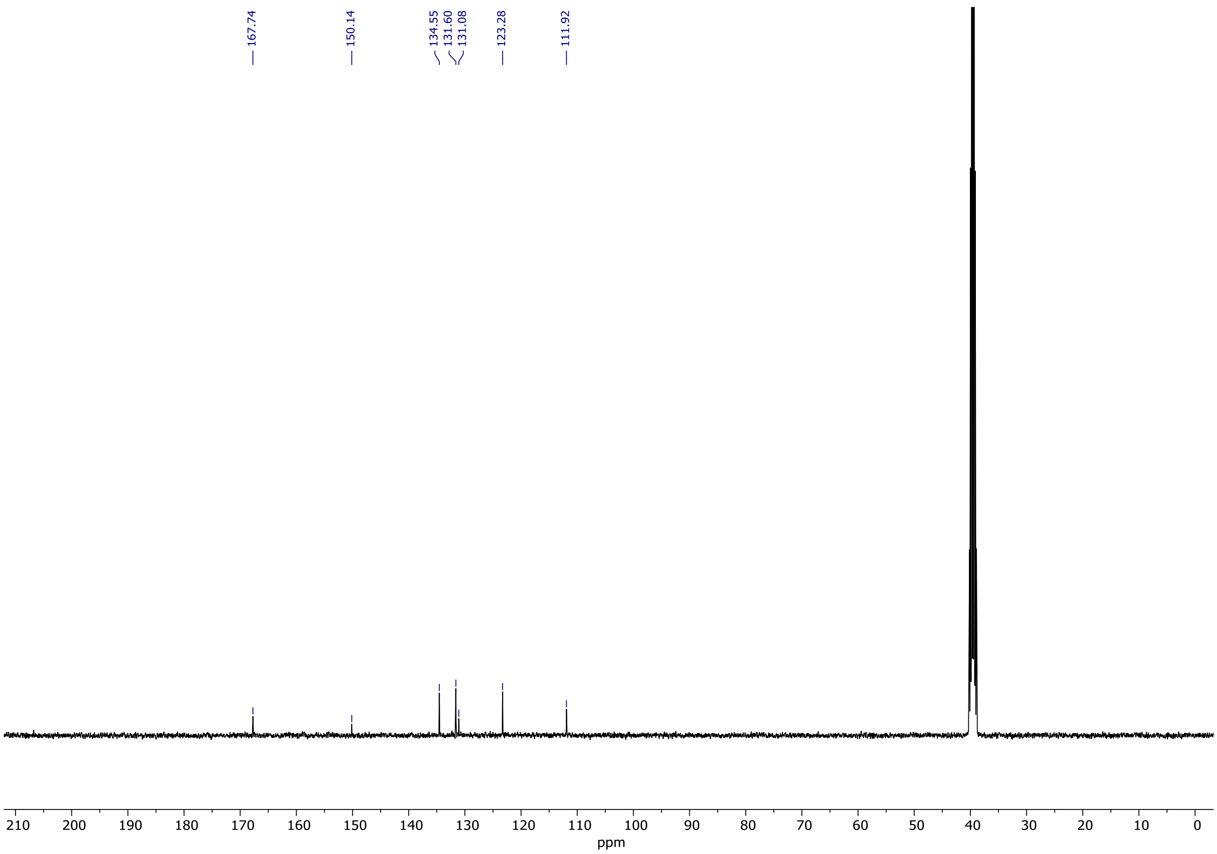

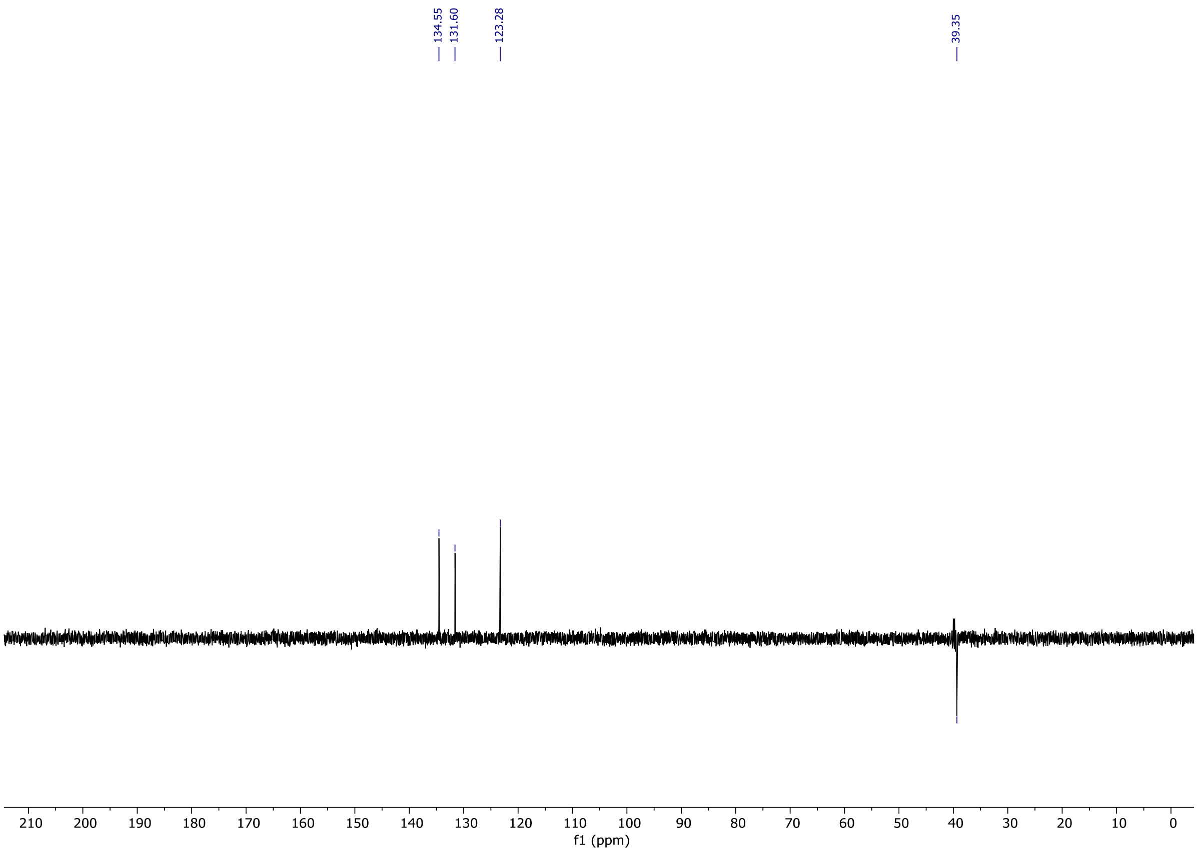

Figure S11: ^{1}H, ^{13}C NMR and 135DEPT for reagent **S7**.

4-(Aminomethyl)-2,6-bromophenol **S8**[2]

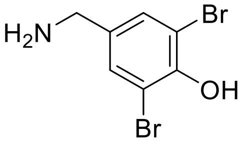

S8

To a stirred solution of 2-(3,5-dibromo-4-hydroxybenzyl)isoindoline-1,3-dione **S7** (3.17 g, 7.71 mmol) in MeOH (71 mL) was added $N_2H_4 \cdot H_2O$ (64%) (3.55 mL) at 0 °C and the reaction mixture was refluxed for 4 h. The mixture was cooled to 0 °C and conc. HCl (15 mL) was added. The mixture was refluxed for 16 h. The solution was cooled to 0 °C, filtered, and washed with cold MeOH (50 mL). The solution was concentrated, and the residue was purified via reverse phase column chromatography (0-100% H_2O:MeCN) to afford 4-(aminomethyl)-2,6-bromophenol **S8** (1.85 g, 6.58 mmol, 85%) as a white solid. **^{1}H NMR** (600 MHz, MeOD) δ 7.62 (s, 2H), 4.00 (s, 2H). **^{13}C NMR** (151 MHz, MeOD) δ 153.3 (C), 134.3 (CH), 128.2 (C), 112.4 (C), 42.7 (CH_2). **IR** (solid): 3332, 2974, 1477, 1131, 772 cm^{-1}.

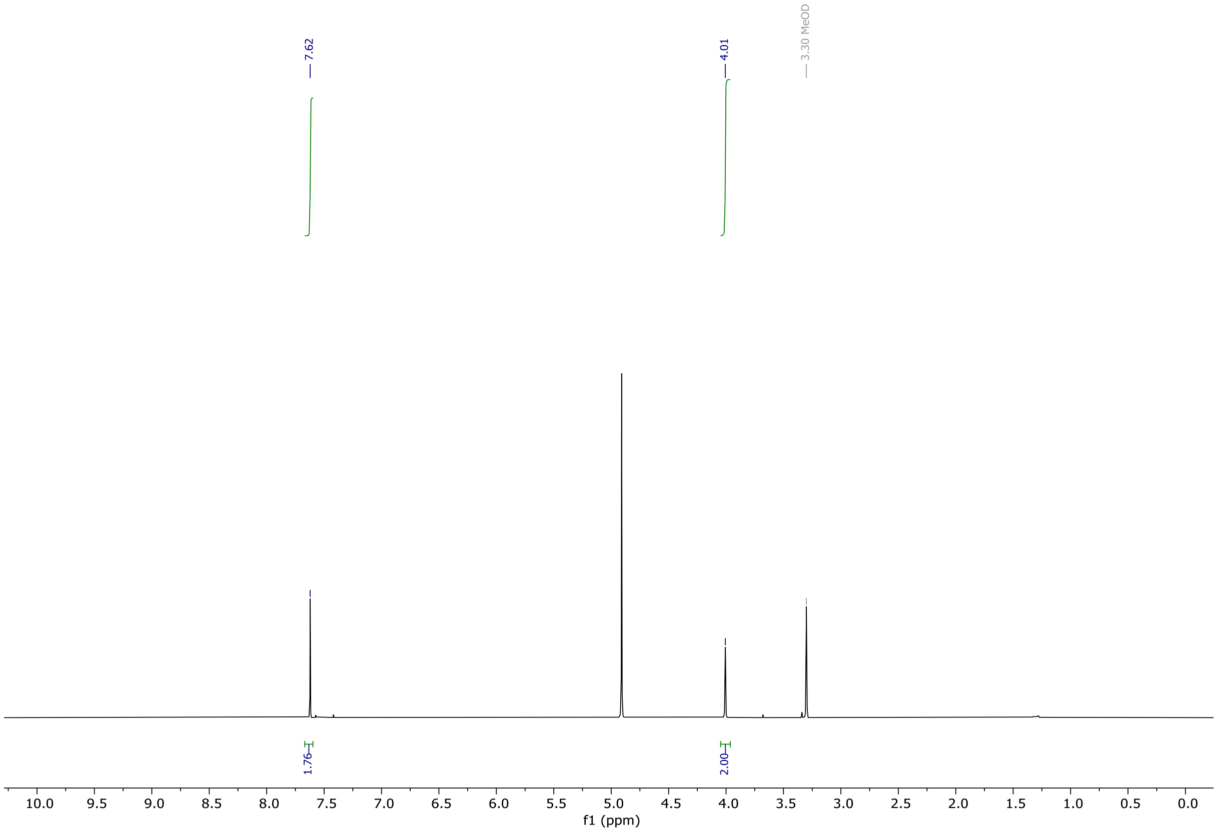

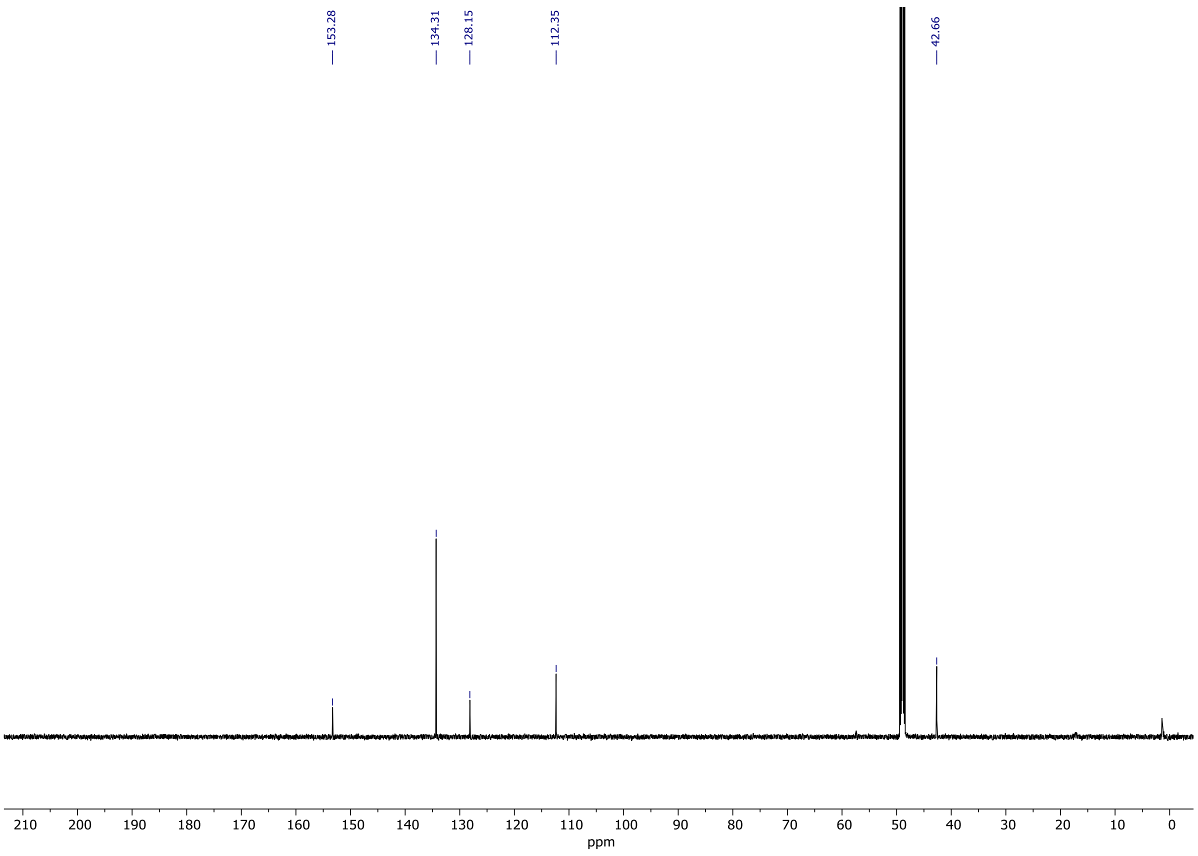

Figure S12: ^{1}H and ^{13}C NMR for reagent **S8**.

3-(4,5-Dibromo-2-methyl-3,6-dioxo-3,6-dihydropyridazin-1(2*H*)-yl)-*N*-(3,5-dibromo-4-hydroxybenzyl)propenamide **S9**

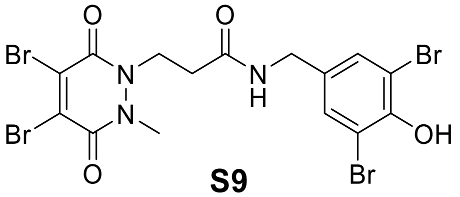

S9

To a solution of 3-(4,5-dibromo-2-methyl-3,6-dioxo-3,6-dihydropyridazin-1(2*H*)-yl) propanoic acid **S3** (1.00 g, 2.81 mmol) in anh. DMF (6 mL) was added EDC·HCl (357 mg, 1.86 mmol) under argon. The reaction mixture was then stirred at 0 °C for 30 mins. After this time, to this solution, was added dropwise a pre-mixed solution of 4-(aminomethyl)-2,6-bromophenol **S8** (373 mg, 1.86 mmol) and NEt_3 (0.30 mL, 2.00 mmol) in anh. DMF (6 mL). After this, the reaction mixture was stirred at 21 °C for 3 h under argon. The reaction mixture was then concentrated *in vacuo* with toluene co-evaporation (4 × 10 mL, as an azeotrope). The crude residue was purified by flash column chromatography (0% to 20% EtOAc (1% AcOH)/MeOH) to afford 3-(4,5-dibromo-2-methyl-3,6-dioxo-3,6-dihydropyridazin-1(2*H*)-yl)-*N*-(3,5-difluoro-4-hydroxybenzyl)propenamide **S9** (0.20 mg, 0.29 mmol, 30%) as a yellow solid. **^{1}H NMR** (500 MHz, MeOD) δ 7.57 (s, 1H), 7.40 (s, 2H), 4.45 (t, *J* = 6.9 Hz, 2H), 4.19 (s, 2H), 3.67 (s, 3H), 2.63 (t, *J* = 6.9 Hz, 2H). **^{13}C NMR** (126 MHz, MeOD) δ 172.0 (C), 154.7 (C), 154.5 (C), 136.7 (C), 136.3 (C), 133.9 (C), 132.9 (2×CH), 112.2 (2×C), 45.5 (CH_2), 42.8 (CH_2), 35.4 (CH_3), 34.6 (CH_2). **IR (solid)**: 3282, 1766, 1615 1565, 1240, 1138, 708 cm^{-1}. **LRMS (ESI)** 624 (16, $[M^{81}Br_4+H]^+$), 622 (65, $[M^{79}Br^{81}Br_3+H]^+$), 620 (100, $[M^{79}Br_2{}^{81}Br_2+H]^+$), 618 (69, $[M^{79}Br_3{}^{81}Br+H]^+$), 616 (18, $[M^{79}Br_4+H]^+$); **HRMS (ESI)** calcd for $C_{15}H_{13}Br_4N_3O_4$ $[M^{79}Br_2{}^{81}Br_2+H]^+$ 619.7677; observed 619.7671.

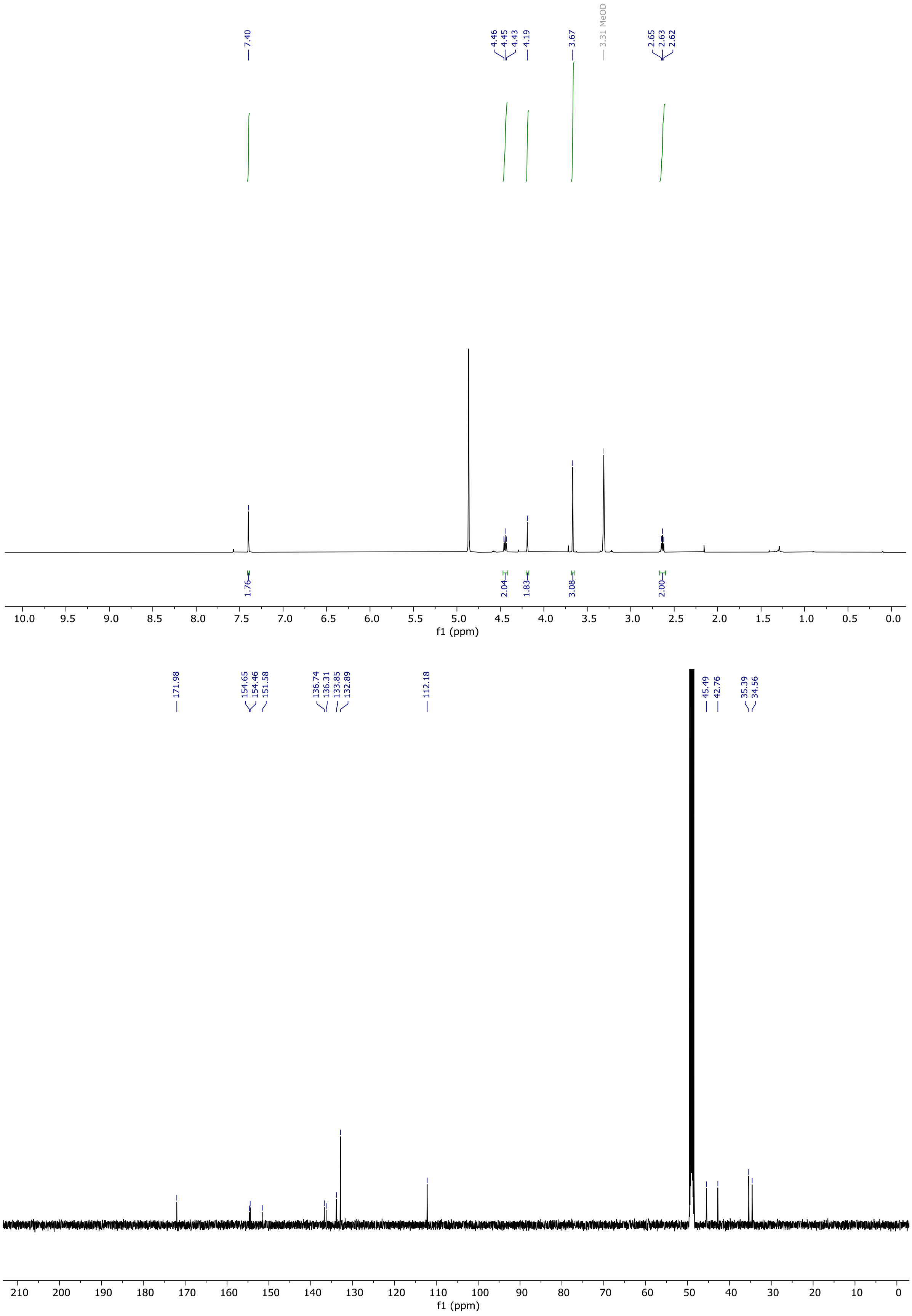

Figure S13: ^{1}H and ^{13}C NMR for reagent **S9**.

2,6-Dibromo-4-((3-(4,5-dibromo-2-methyl-3,6-dioxo-3,6-dihydropyridazin-1(2*H*)-yl)propanamido)methyl)phenyl 6-azidohexanoate **4**

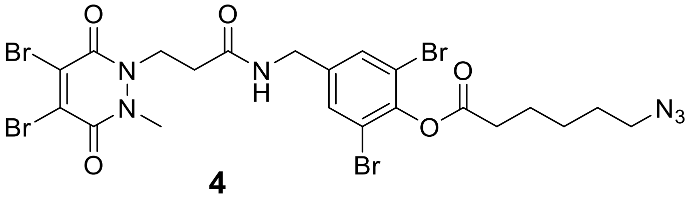

4

To a solution of 6-azidohexanoic acid **S6** (41 mg, 0.26 mmol) in anh. DMF (4 mL) was added EDC·HCl (75 mg, 0.39 mmol) and DMAP (24.4 mg, 0.20 mmol). The reaction mixture was stirred at 0 °C for 30 min under argon. After this time, to this solution, was added dropwise a pre-mixed solution of 3-(4,5-dibromo-2-methyl-3,6-dioxo-3,6-dihydropyridazin-1(2*H*)-yl)-*N*-(3,5-dibromo-4-hydroxybenzyl)propenamide **S9** (80 mg, 0.13 mmol) and NEt_3 (28 µL, 0.20 mmol) in anh. DMF (4 mL). After this, the reaction mixture was stirred at 21 °C at for 16 h. Upon completion, the solvent mixture was concentrated *in vacuo* with toluene co-evaporation (3 × 30 mL, as an azeotrope) and the crude residue purified by flash column chromatography (0% to 20% DMC/MeOH) to yield 2,6-dibromo-4-((3-(4,5-dibromo-2-methyl-3,6-dioxo-3,6-dihydropyridazin-1(2*H*)-yl)propanamido)methyl)phenyl 6-azidohexanoate **4** (30 mg, 0.04 mmol, 30%) as a colourless oil. ^{1}**H NMR** (600 MHz, CD_3CN) δ 7.54 (s, 2H), 7.13 – 6.82 (m, 1H), 4.33 (t, *J* = 7.0 Hz, 2H), 4.26 (d, *J* = 6.1 Hz, 2H), 3.57 (s, 3H), 3.32 (t, *J* = 6.8 Hz, 2H), 2.69 (t, *J* = 7.3 Hz, 2H), 2.56 (t, *J* = 7.0 Hz, 2H), 1.78 (p, *J* = 7.4 Hz, 2H), 1.68 – 1.56 (m, 2H), 1.56 – 1.45 (m, 2H). ^{13}**C NMR** (151 MHz, CD_3CN) δ 171.2 (C), 170.7 (C), 154.0 (C), 153.8 (C), 145.8 (C), 141.43, 136.6 (C), 136.1 (C), 132.4 (2×CH) , 118.1 (C), 51.9 (CH_2), 44.9 (CH_2), 42.3 (CH_2), 35.5 (CH_3), 34.2 (CH_2), 34.2 (CH_2), 29.1 (CH_2), 26.8 (CH_2), 25.0 (CH_2). **IR** (thin film): 3325, 2930, 2094, 1769, 1709, 1628, 1536, 1239, 1116 cm^{-1}. **LRMS (ESI)** 763 (18, $[M^{81}Br_4+H]^+$), 761 (65, $[M^{79}Br^{81}Br_3+H]^+$) 756 (100, $[M^{79}Br_2{}^{81}Br_2+H]^+$), 756 (69, $[M^{79}Br_3{}^{81}Br+H]^+$) 755 (18, $[M^{79}Br_4+H]^+$); **HRMS (ESI)** calcd for $C_{17}H_{15}Br_4N_6O_5$ $[M^{79}Br_2{}^{81}Br_2+H]^+$ 758.8423; observed 758.8417.

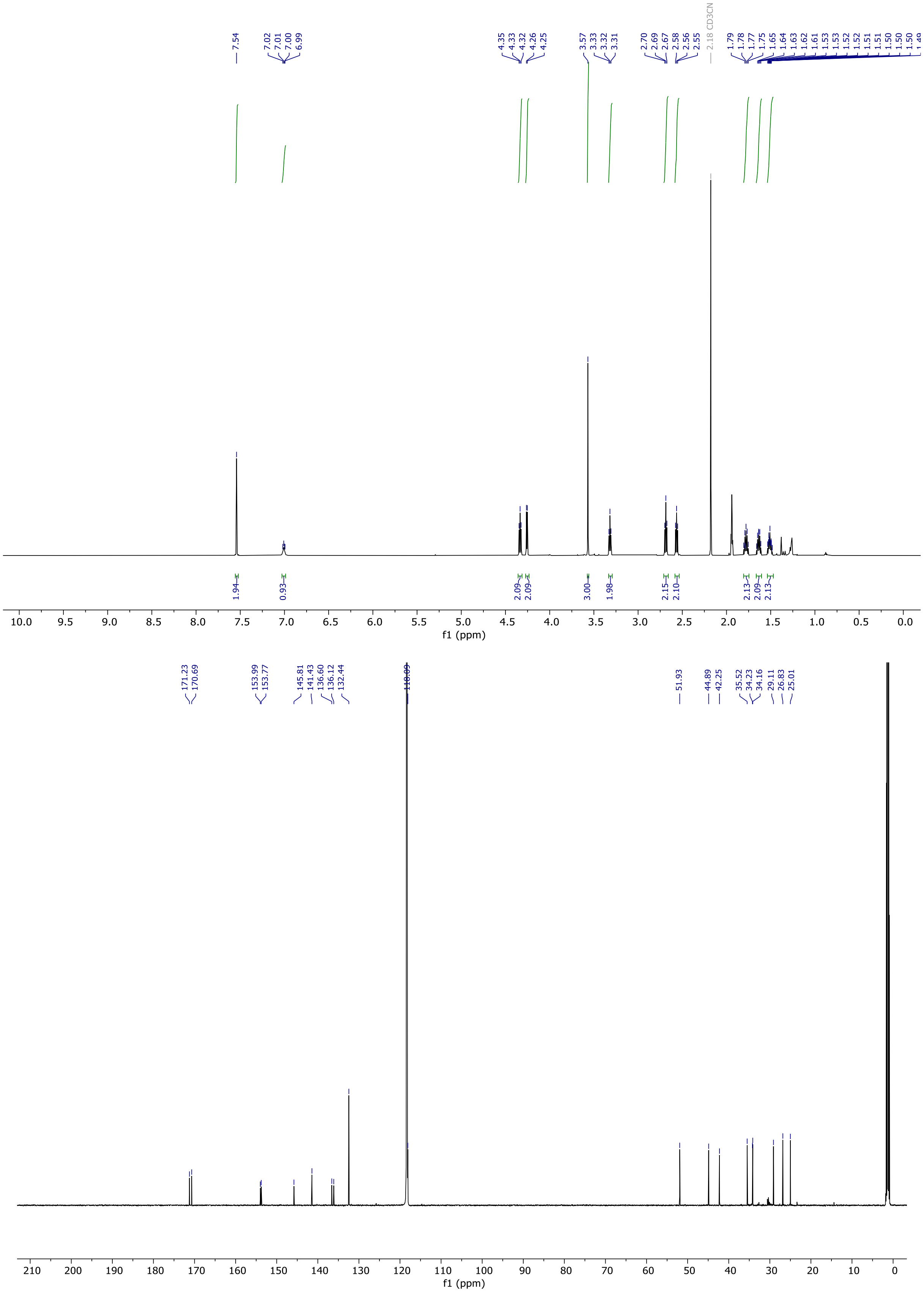

Figure S14: ^{1}H and ^{13}C NMR for reagent **4**.

2-(3,5-Dichloro-4-hydroxybenzyl)isoindoline-1,3-dione **S10**[2]

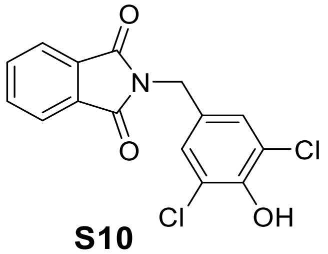

S10

To a solution of 2,6-dichlorophenol (2.00 g, 12.30 mmol) in DI H_2O (15 mL) was added *N*-(hydroxymethyl)phthalimide (2.40 g, 13.50 mmol) at 0 °C. H_2SO_4 (35 mL) was added slowly and the reaction mixture was stirred at 0 °C for 5 min and then at 21 °C for 16 h. After this time, EtOAc (30 mL) and H_2O (30 mL) were added to the solution and the resulting white precipitate was filtered and washed with EtOAc and H_2O to afford 2-(3,5-dichloro-4-hydroxybenzyl)isoindoline-1,3-dione **S10** (2.76 g, 8.57 mmol, 70%) as a white solid. **^{1}H NMR** (600 MHz, DMSO) δ 7.91 – 7.86 (m, 2H, Ar-H), 7.88 – 7.82 (m, 2H, Ar-H), 7.33 (s, 2H, Ar-H), 4.67 (s, 2H, CH_2). **^{13}C NMR** (151 MHz, DMSO) δ 167.8 (C), 148.4 (C), 134.6 (CH), 131.7 (C), 129.6 (C), 127.9 (C), 123.3 (CH), 122.2 (C), 39.6 (CH_2). **IR** (solid): 3339, 1764, 1702, 1232, 1146, 949, 709 cm^{-1}.

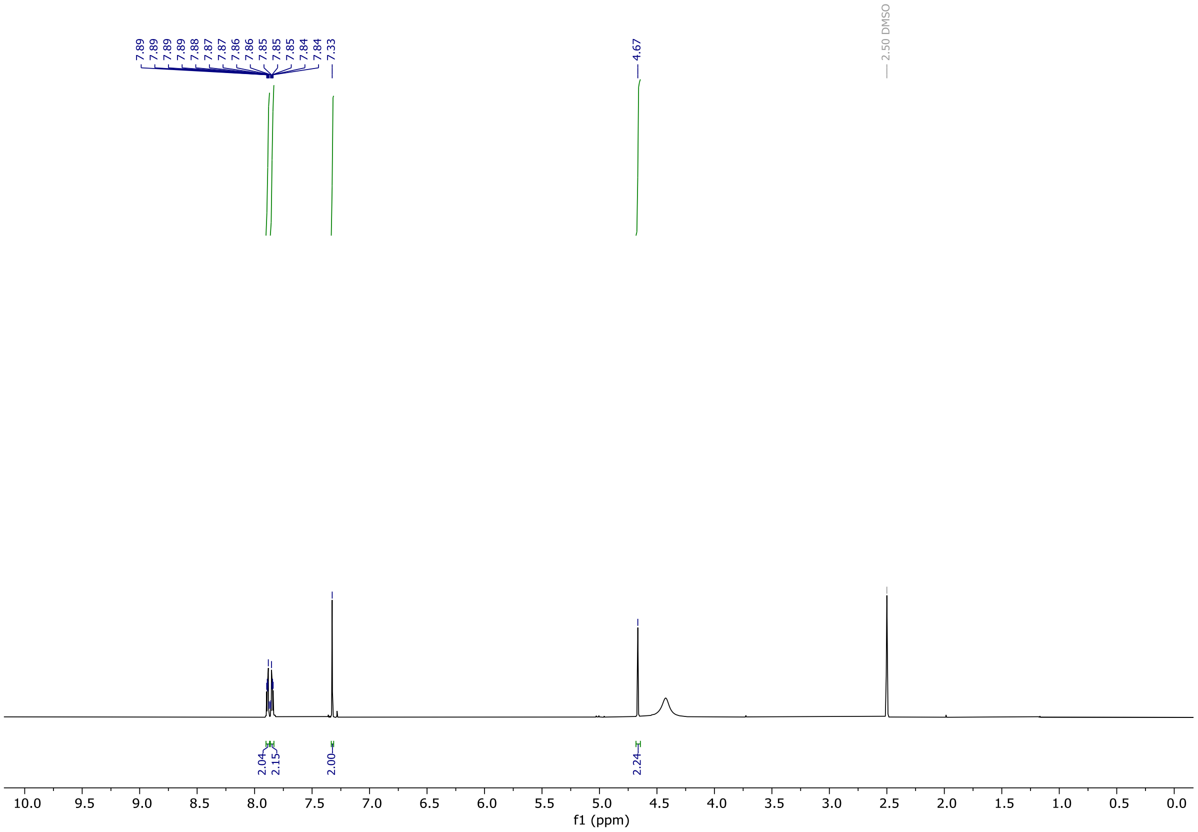

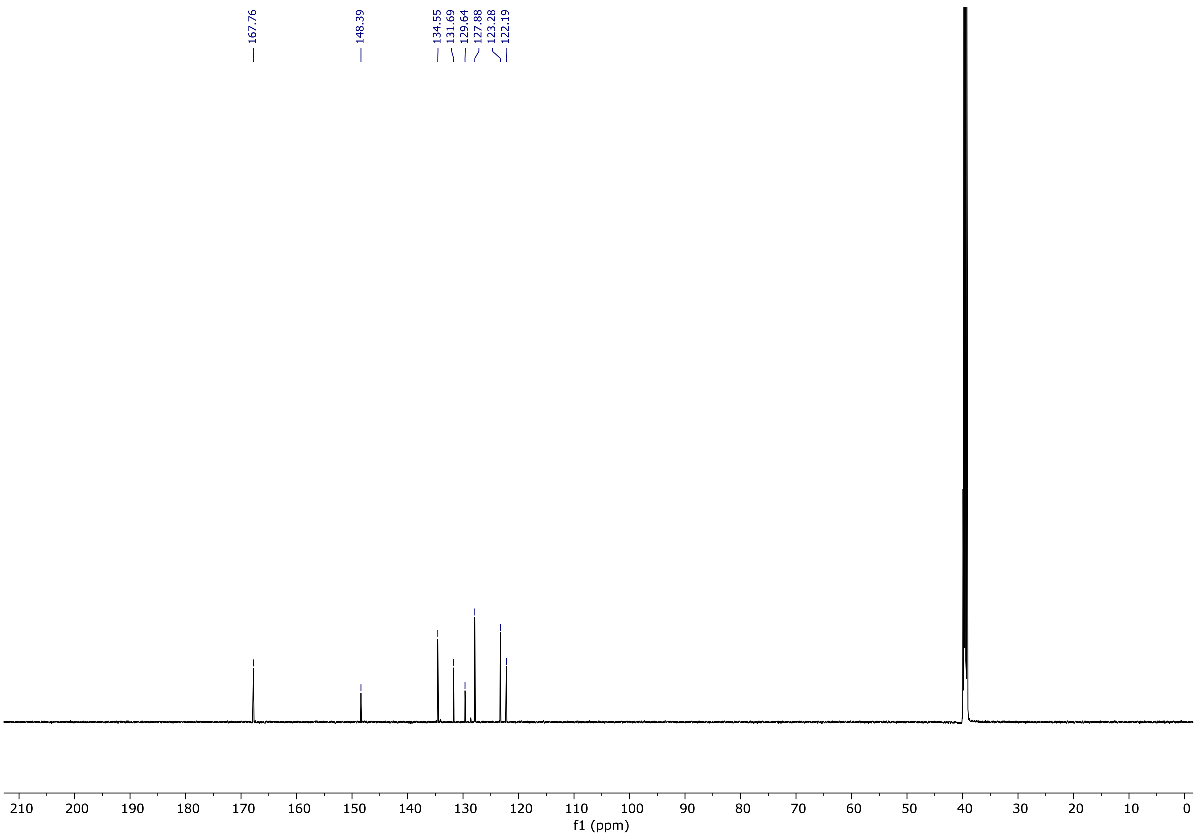

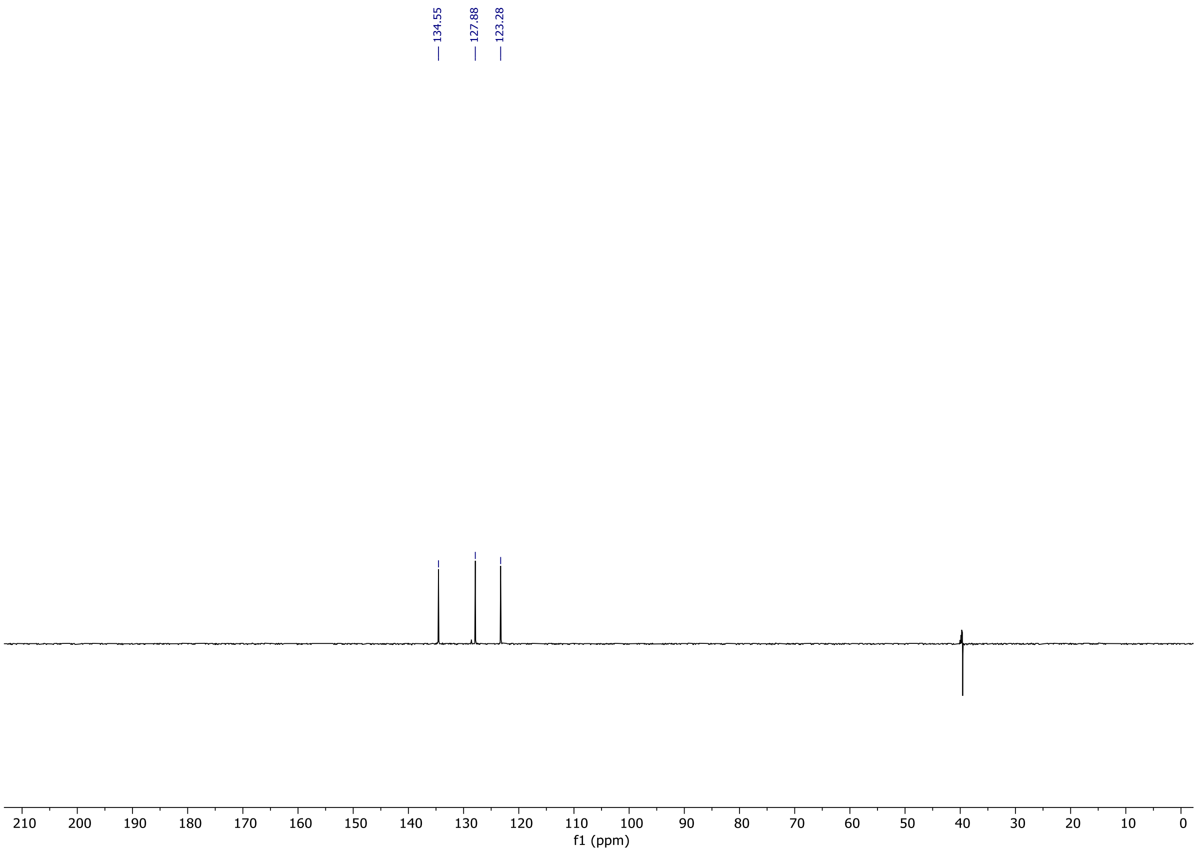

Figure S15: ^{1}H, ^{13}C NMR and 135DEPT for reagent **S10**.

4-(Aminomethyl)-2,6-dichlorophenol **S11**[2]

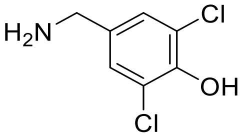

S11

To a stirred solution of 2-(3,5-dichloro-4-hydroxybenzyl)isoindoline-1,3-dione **S10** (2.2 g, 6.83 mmol) in MeOH (45 mL) was added $N_2H_4 \cdot H_2O$ (64%) (2.59 mL) at 0 ˚C and the reaction mixture was refluxed for 4 h. The mixture was cooled to 0 °C and conc. HCl (15 mL) was added. The mixture was refluxed overnight. After this time, the solution was cooled to 0 ˚C, filtered and washed with cold EtOAc (30 mL). The solution was concentrated, and the residue was purified *via* reverse phase column chromatography (0-10% H_2O:MeCN) to afford 4-(aminomethyl)-2,6-dichlorophenol **S11** (1.10 g, 5.73 mmol, 84%) as a white solid. **^{1}H NMR** (600 MHz, MeOD) δ 7.43 (s, 1H), 4.02 (s, 1H). **^{13}C NMR** (151 MHz, MeOD) δ 151.5 (C), 130.5 (C), 127.1 (CH), 123.6 (C), 42.9 (CH_2). **IR** (solid): 3522, 3396, 1618, 1097, 596 cm^{-1}.

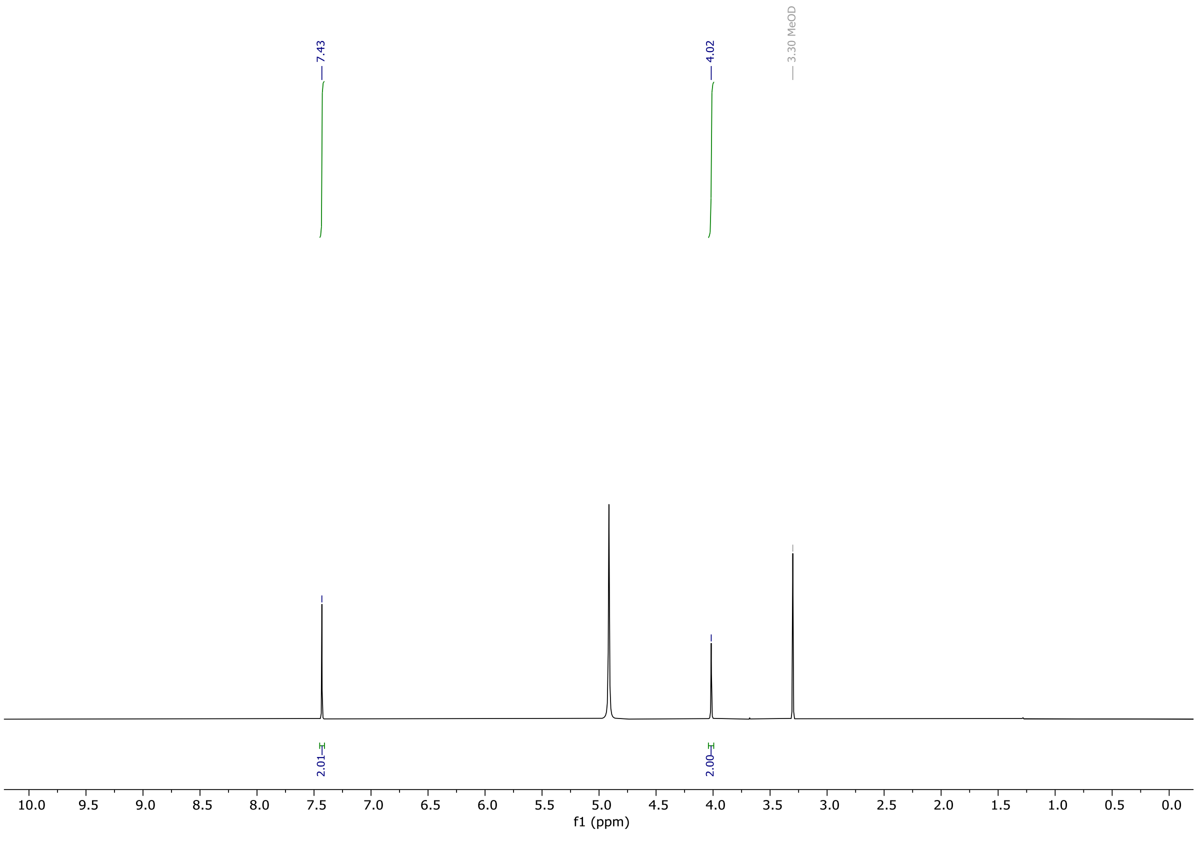

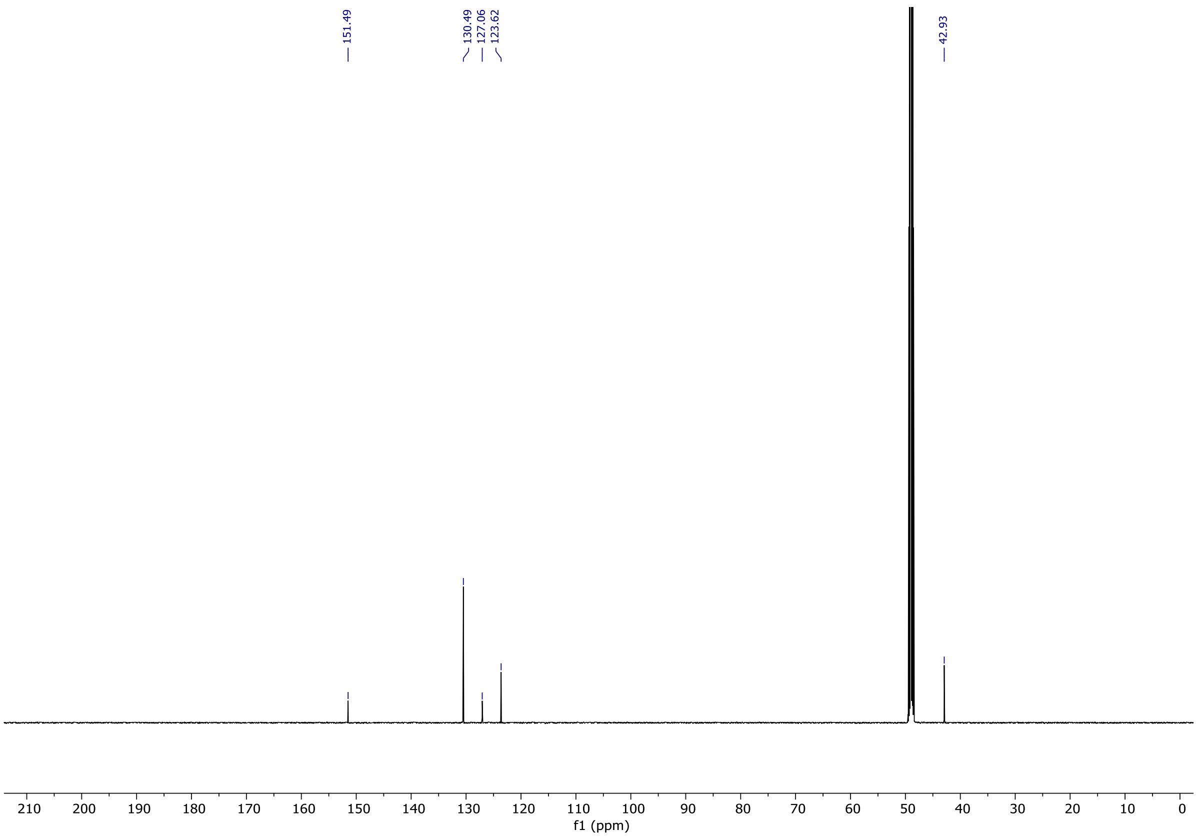

Figure S16: ^{1}H and ^{13}C NMR for reagent **S11**.

3-(4,5-Dibromo-2-methyl-3,6-dioxo-3,6-dihydropyridazin-1(2*H*)-yl)-*N*-(3,5-dichloro-4-hydroxybenzyl)propenamide **S12**

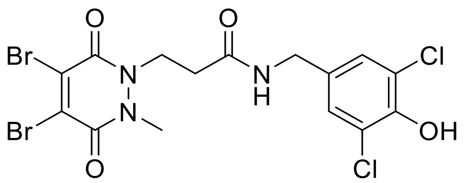

S12

To a solution of 3-(4,5-dibromo-3,6-dioxo-2-phenyl-3,6-dihydropyridazin-1(2*H*)-yl)propanoic acid **S3** (270 mg, 0.76 mmol) in anh. DMF (8 mL) was added EDC·HCl (174 mg, 0.91 mmol). The reaction mixture was then stirred for 30 mins at 0 °C under argon. After this time, to this solution, was added dropwise a pre-mixed solution of 4-(aminomethyl)-2,6-dichlorophenol **S11** (160 mg, 0.83 mmol) and NEt_3 (0.13 mL, 0.91 mmol) in anh. DMF (4 mL) under argon. After this, the reaction mixture was stirred at 21 °C for 3.5 h under argon. The reaction mixture was then concentrated *in vacuo* with toluene co-evaporation (3 × 30 mL, as an azeotrope). The crude residue was then purified by flash column chromatography (0% to 80% EtOAc (1% AcOH) /cyclohexane) to afford 3-(4,5-dibromo-2-methyl-3,6-dioxo-3,6-dihydropyridazin-1(2*H*)-yl)-*N*-(3,5-dichloro-4-hydroxybenzyl)propenamide **S12** (160 mg, 0.30 mmol, 40%) as a yellow solid. **^{1}H NMR** (600 MHz, MeOD) δ 7.92 (s, 1H), 7.20 (s, 2H), 4.45 (t, *J* = 6.9 Hz, 2H), 4.19 (s, 2H), 3.67 (s, 3H), 2.63 (t, *J* = 6.9 Hz, 2H). **^{13}C NMR** (151 MHz, MeOD) δ 172.0 (C), 154.6 (C), 154.4 (C), 149.9 (C), 136.8 (C), 136.3 (C), 132.5 (C), 129.0 (2×CH), 123.3 (C), 45.5 (CH_2), 43.0 (CH_2), 35.4 (CH_3), 34.6 (CH_2). **IR** (solid): 3294, 2926, 1630, 1609, 1567, 707 cm^{-1}. **LRMS (ESI)** 536 (6, $[M^{81}Br_2{}^{35}Cl^{37}Cl+H]^+$), 534 (32, $[M^{81}Br_2{}^{35}Cl^{37}Cl+H]^+$), 532 (64, $[M^{79}Br^{81}Br^{35}Cl^{37}Cl+H]^+$), 530 (100, $[M^{79}Br^{81}Br^{35}Cl_2+H]^+$), 528 (51, $[M^{79}Br_2{}^{35}Cl_2+H]^+$); **HRMS (ESI)** calcd for $C_{15}H_{13}Br_2Cl_2N_3O_4$ $[M^{79}Br^{81}Br^{35}Cl_2+H]^+$ 529.8708; observed 528.8702.

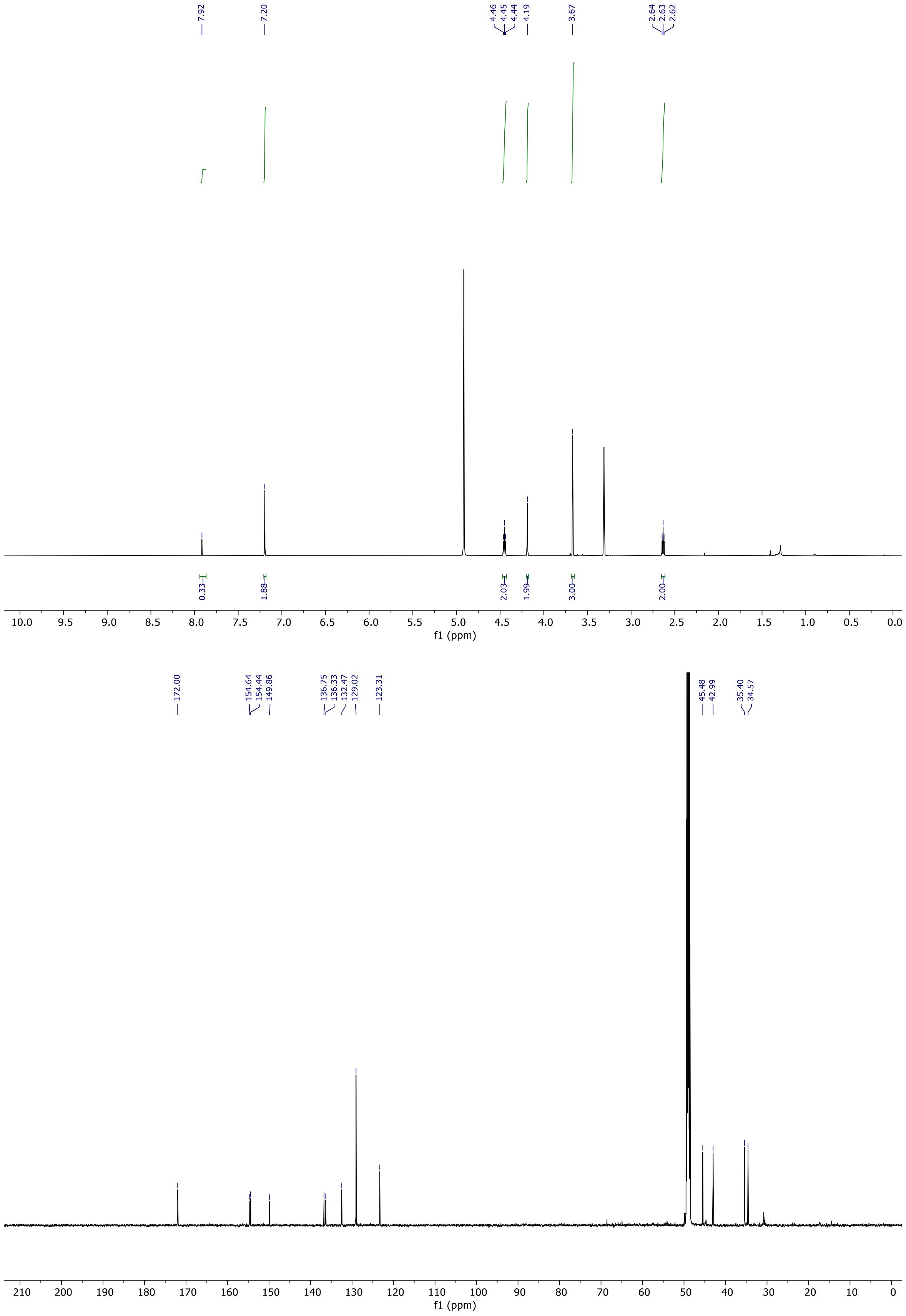

Figure S17: ^{1}H and ^{13}C NMR for reagent **S12**.

2,6-Dichloro-4-((3-(4,5-dibromo-2-methyl-3,6-dioxo-3,6-dihydropyridazin-1(2*H*)-yl)propanamido)methyl)phenyl 6-azidohexanoate **5**

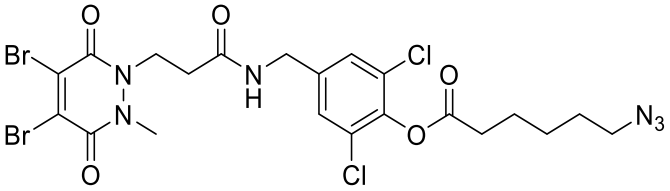

5

To a solution of 6-azidohexanoic acid **S6** (60.0 mg, 0.44 mmol) in anh. DMF (2 mL) was added EDC·HCl (127 mg, 0.66 mmol), and the reaction mixture was stirred for 30 mins at 0 °C under argon. After this time, to this solution, was added dropwise a pre-mixed solution of 3-(4,5-dibromo-2-methyl-3,6-dioxo-3,6-dihydropyridazin-1(2*H*)-yl)-*N*-(3,5-dichloro-4-hydroxybenzyl)propenamide **S12** (100 mg, 0.19 mmol) and NEt_3 (40 µL, 0.29 mmol) in anh. DMF (4 mL). After this, the reaction mixture was stirred at 21 °C for 6 h under argon. After this time, the reaction mixture was then concentrated *in vacuo* with toluene co-evaporation (3 × 30 mL, as an azeotrope). The crude residue was purified by flash column chromatography (0% to 10% DCM/MeOH) to afford 2,6-dichloro-4-((3-(4,5-dibromo-2-methyl-3,6-dioxo-3,6-dihydropyridazin-1(2*H*)-yl)propanamido)methyl)phenyl 6-azidohexanoate **5** (50 mg, 0.08 mmol, 40%) as a yellow oil. **^{1}H NMR** (600 MHz, CD_3CN) δ 7.36 (s, 1H), 7.16 – 7.03 (m, 1H), 4.34 (t, J = 7.0 Hz, 1H), 4.26 (d, J = 6.0 Hz, 1H), 3.57 (s, 1H), 3.32 (t, J = 6.9 Hz, 2H), 2.69 (t, J = 7.3 Hz, 2H), 2.58 (t, J = 7.0 Hz, 1H), 1.77 (p, J = 7.4 Hz, 1H), 1.63 (p, J = 7.0 Hz, 1H), 1.55 – 1.42 (m, 1H). **^{13}C NMR** (151 MHz, CD_3CN) δ 171.3 (C), 170.7 (C), 154.0 (C), 153.8 (C), 143.5 (C), 140.6 (C), 136.6 (C), 136.1 (C), 128.7 (2×CH), 51.9 (CH_2), 44.9 (CH_2), 42.5 (CH_2), 35.5 (CH_3), 34.3(CH_2), 33.9 (CH_2), 29.1 (CH_2), 26.8 (CH_2), 25.1 (CH_2). **IR** (thin film): 3327, 2932, 1770, 1633, 1252, 709 cm^{-1}. **LRMS (ESI)** 675 (5, $[M^{81}Br_2{}^{37}Cl_2+H]^+$), 673 (31, $[M^{81}Br_2{}^{35}Cl^{37}Cl+H]^+$), 671 (63, $[M^{79}Br^{81}Br^{35}Cl^{37}Cl+H]^+$), 669 (100, $[M^{79}Br^{81}Br^{35}Cl_2+H]^+$), 667 (51, $[M^{79}Br_2{}^{35}Cl_2+H]^+$); **HRMS (ESI)** calcd for $C_{15}H_{13}Br_2C_{l2}N_3O_4$ $[M^{79}Br^{81}Br+H]^+$ 668.9453 ; observed 668.9448.

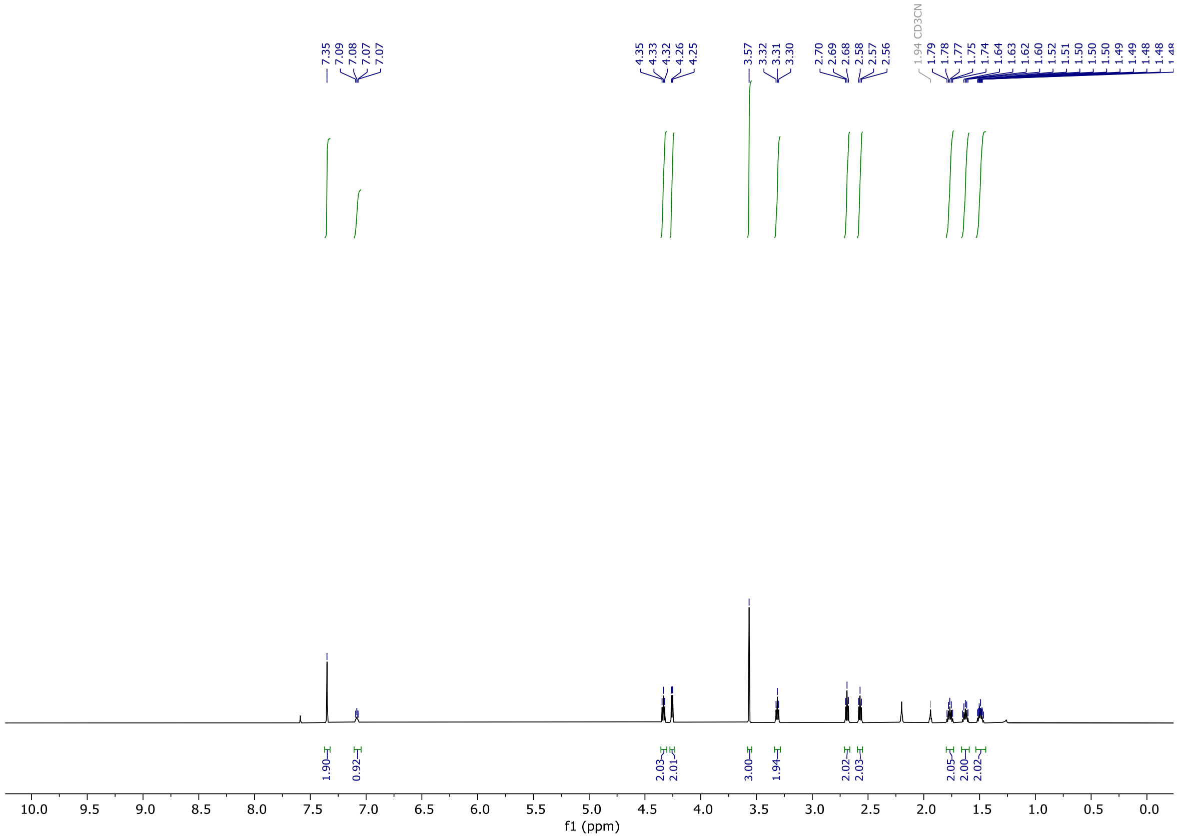

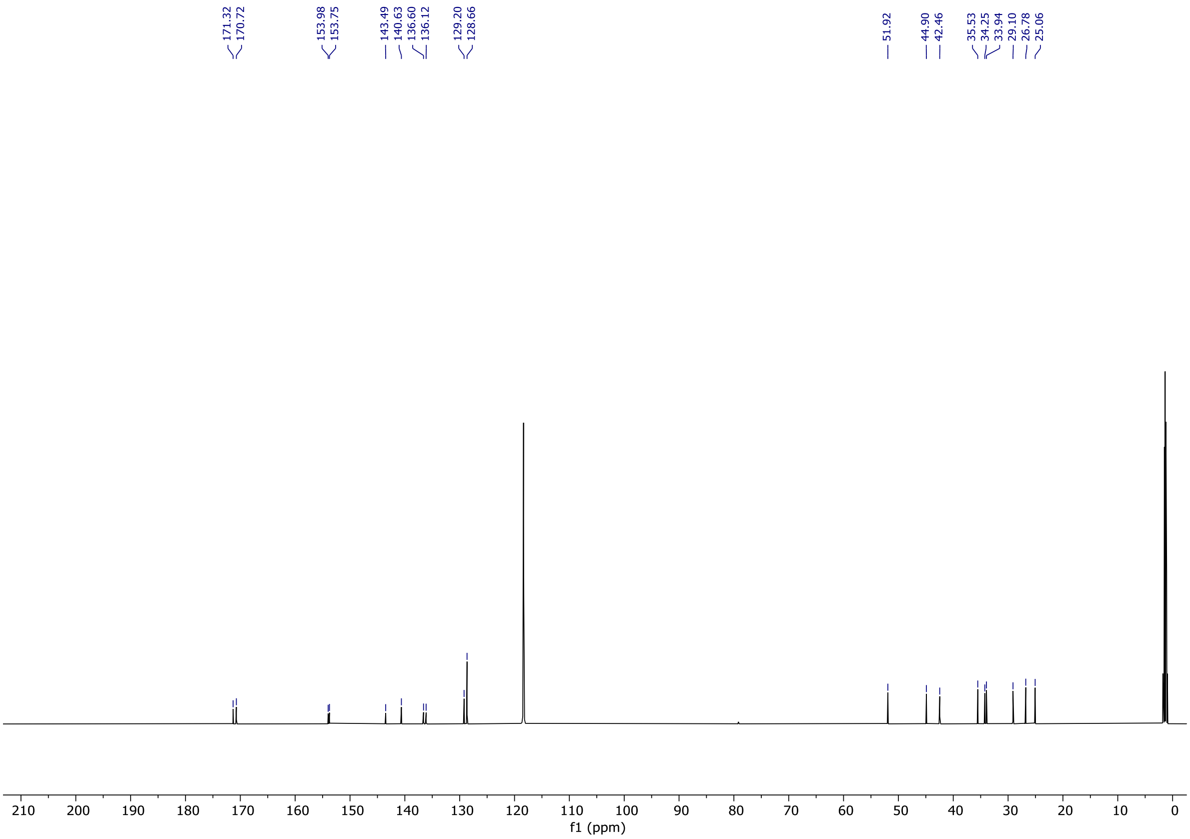

Figure S18: ^{1}H and ^{13}C NMR for reagent **5**.

2-(3,5-Difluoro-4-hydroxybenzyl)isoindoline-1,3-dione **S13**[2]

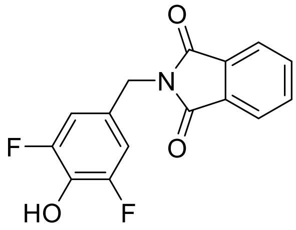

S13

To a solution of 2,6-difluorophenol (1.00 g, 7.70 mmol) and *N*-(hydroxymethyl)phthalimide (1.36 g, 7.70 mmol) in 7 mL H_2O was added conc. H_2SO_4 (20 mL) at –5 °C and left to stir for 4 h and then at 21 °C for 16 h. After this time, H_2O (30 mL) was added and the resulting precipitate was filtered and washed with cold H_2O (30 mL) to afford 2-(3,5-difluoro-4-hydroxybenzyl)isoindoline-1,3-dione **S13** (1.93 g, 6.67 mmol, 87%) as a white solid. **^{1}H NMR** (500 MHz, DMSO) δ 10.2 (s, 1H), 7.91 – 7.82 (m, 4H), 6.99 (dd, J = 7.8, 1.5 Hz, 2H), 4.67 (s, 2H). **^{13}C NMR** (126 MHz, DMSO) δ 167.7 (2×C), 152.1 (dd, J = 242.3, 7.1 Hz, 2×C), 134.5 (2×CH), 132.9 (t, J = 16.1 Hz, C), 131.7 (2×C), 127.4 (t, J = 7.9 Hz ,C), 123.3 (2×CH), 111.1 (dd, J = 16.2, 6.5 Hz, 2×CH), 39.8 (CH_2). **IR** (solid): 3338, 1767, 1700, 1610, 1530, 1398, 1317 cm^{-1}.

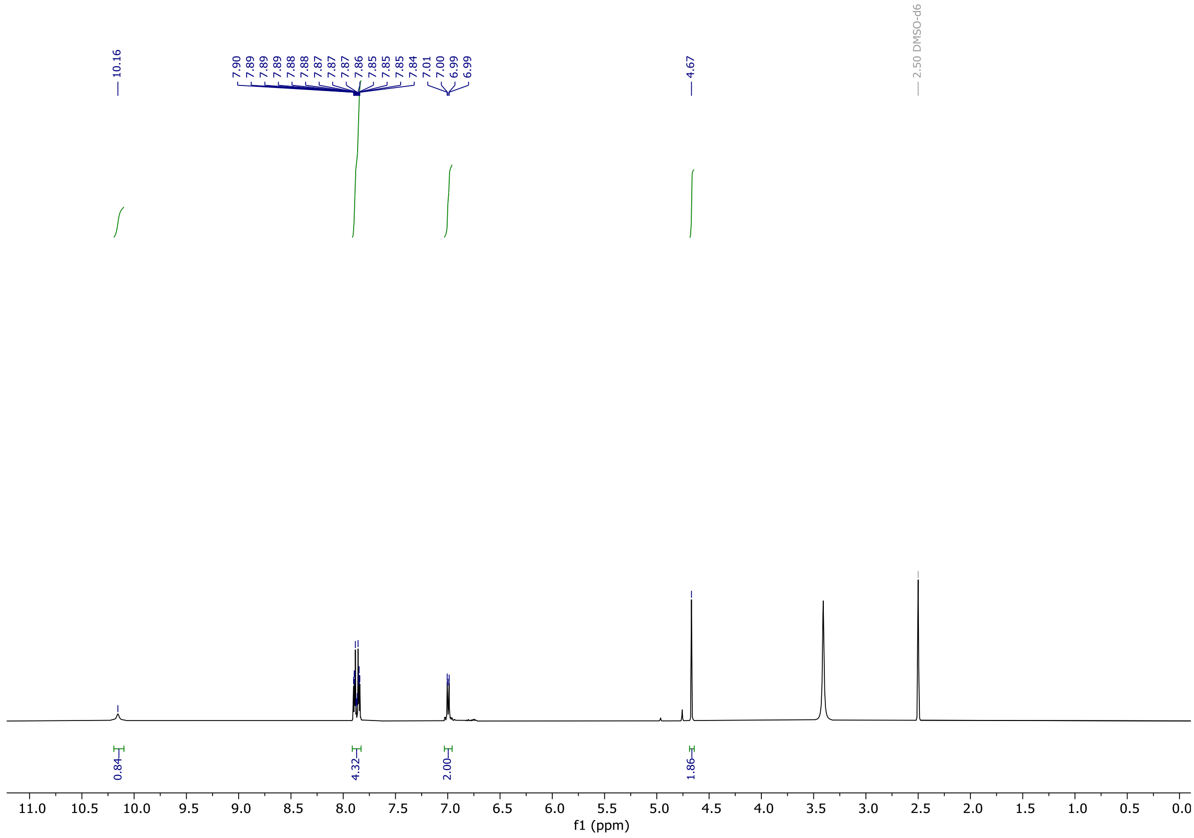

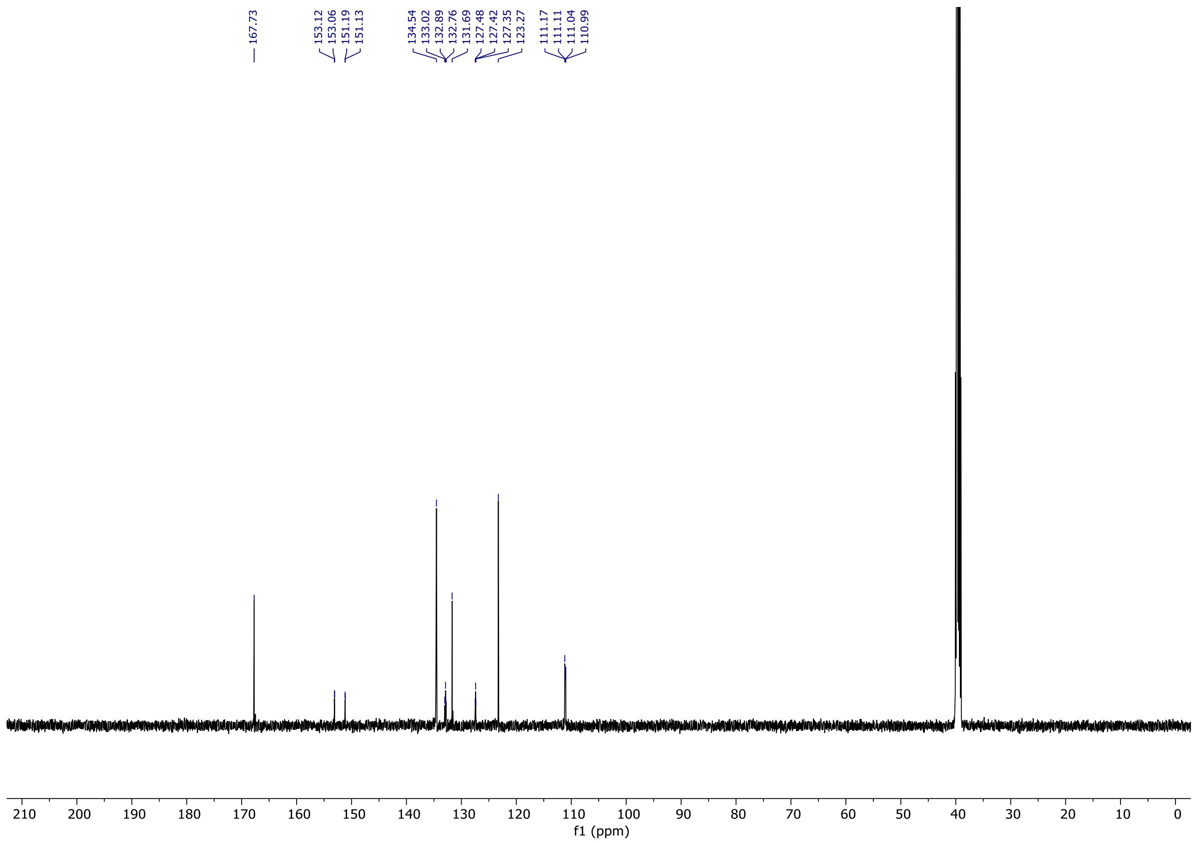

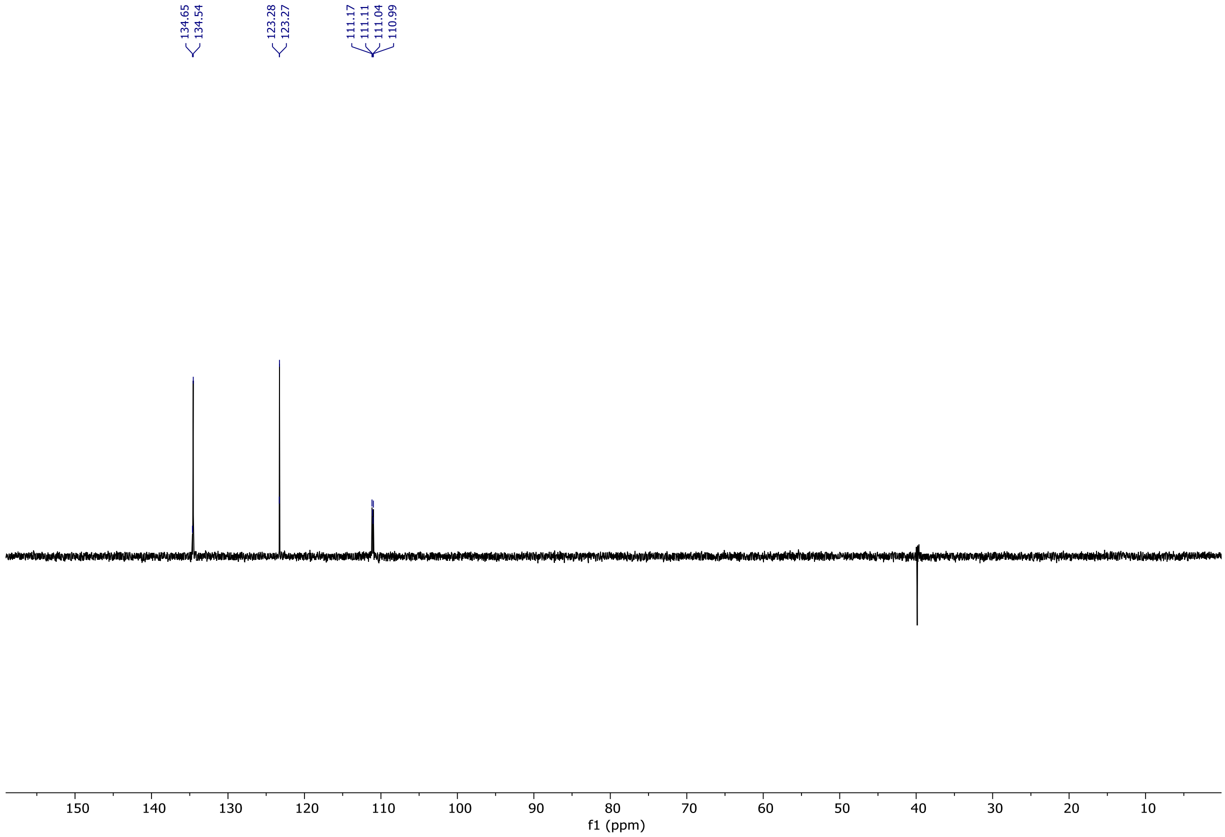

Figure S19: ^{1}H, ^{13}C NMR and 135DEPT for reagent **S13**.

4-(Aminomethyl)-2,6-difluorophenol **S14**

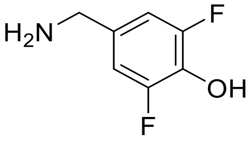

S14

To a stirred solution of 2-(3,5-difluoro-4-hydroxybenzyl)isoindoline-1,3-dione **S13** (500 mg, 1.73 mmol) in MeOH (20 mL) was added $N_2H_4{\cdot}H_2O$ (64%) (0.85 mL) and the reaction mixture was refluxed for 4 h. The mixture was cooled to 0 °C and conc. HCl (5 mL) was added. The mixture was refluxed for 16 h. The solution was cooled to 0 °C, filtered and washed with cold MeOH. The solution was concentrated, and the residue was purified via reverse phase column chromatography (0-100% H_2O:MeCN) to afford 4-(aminomethyl)-2,6-difluorophenol **S14** (150 mg, 0.94 mmol, 54%) as a white solid. **^{1}H NMR** (700 MHz, MeOD) δ 7.06 (dd, J = 7.2, 1.7 Hz, 1H), 4.02 (s, 1H). **^{13}C NMR** (176 MHz, MeOD) δ 153.8 (dd, J = 243.4, 7.3 Hz, 2×C), 136.2 (t, J = 15.8 Hz, C), 124.6 (t, J = 8.4 Hz, C), 113.6 (dd, J = 17.5, 5.6 Hz, 2×CH), 43.2 (CH_2). **IR** (solid): 2953, 2843, 1598, 1528, 1012 cm^{-1}

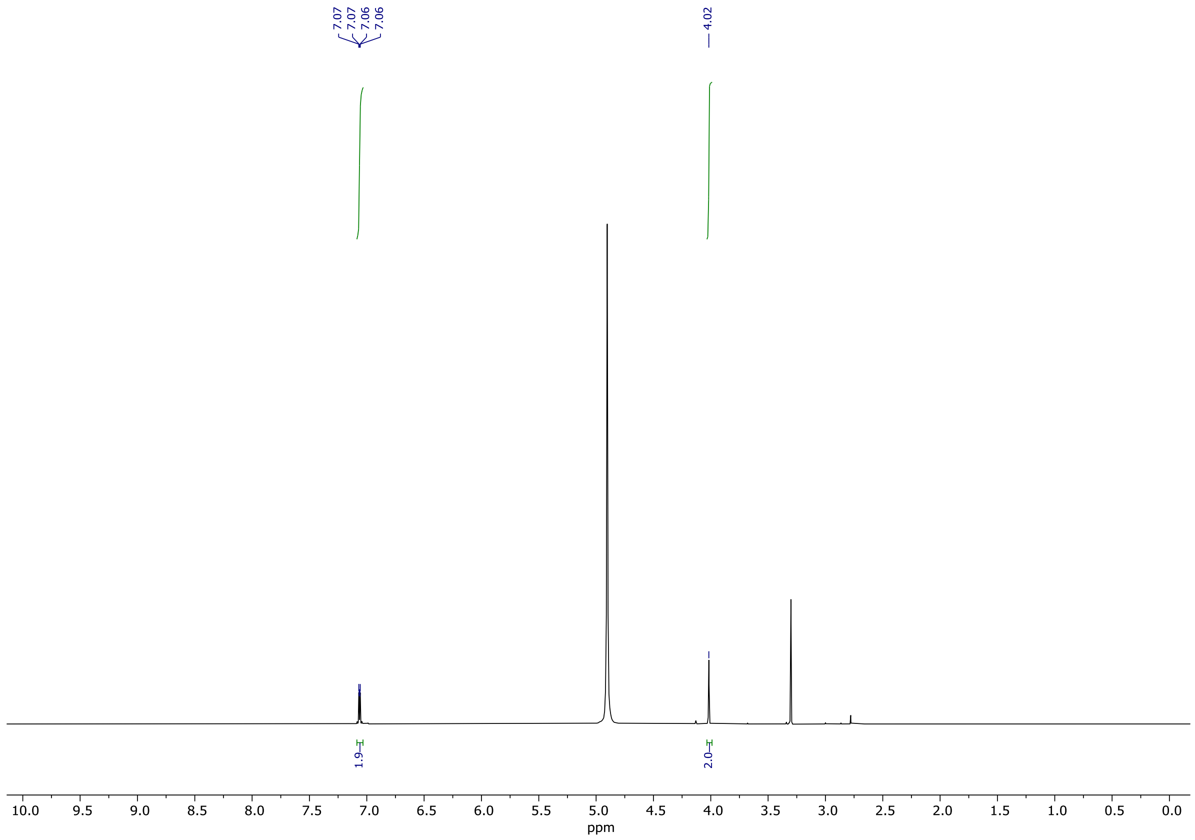

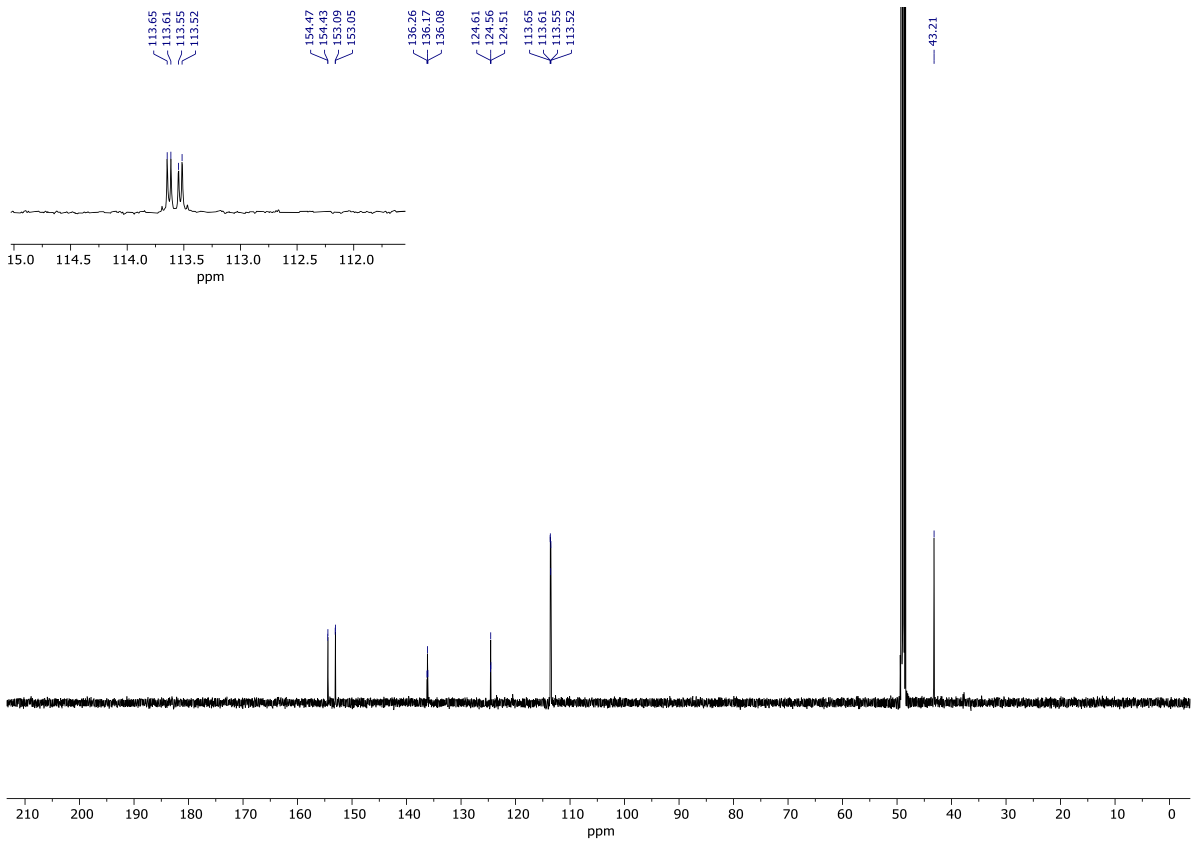

Figure S20: ^{1}H and ^{13}C NMR for reagent **S14**.

3-(4,5-Dibromo-2-methyl-3,6-dioxo-3,6-dihydropyridazin-1(2*H*)-yl)-*N*-(3,5-difluoro-4-hydroxybenzyl)propenamide **S15**

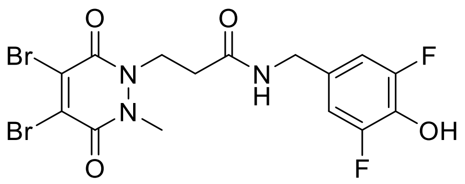

S15

To a solution of 3-(4,5-dibromo-2-methyl-3,6-dioxo-3,6-dihydropyridazin-1(2*H*)-yl) propanoic acid **S3** (600 mg, 1.69 mmol) in anh. DMF (6 mL) was added EDC·HCl (357 mg, 1.86 mmol). The solution was stirred at 0 °C for 30 min under argon. After this time, to this solution, was added dropwise a pre-mixed solution of 4-(aminomethyl)-2,6-,difluorophenol **S14** (373 mg, 1.86 mmol) and NEt_3 (0.30 mL, 2.00 mmol) in anh. DMF (6 mL). After this, the reaction mixture was stirred at 21 °C for 3 h under argon. The reaction mixture was then concentrated *in vacuo* with toluene co-evaporation (3 × 30 mL, as an azeotrope). The crude residue was then purified by flash column chromatography (0% to 20% EtOAc/MeOH) to afford 3-(4,5-dibromo-2-methyl-3,6-dioxo-3,6-dihydropyridazin-1(2*H*)-yl)-*N*-(3,5-difluoro-4-hydroxybenzyl)propenamide **S15** (0.38 mg, 0.76 mmol, 45%) as a yellow solid. **^{1}H NMR** (700 MHz, MeOD) δ 6.83 (d, *J* = 7.7 Hz, 2H), 4.46 (t, *J* = 6.9 Hz, 2H), 4.20 (s, 2H), 3.67 (s, 3H), 2.62 (t, *J* = 6.9 Hz, 2H). **^{13}C NMR** (151 MHz, MeOD) δ 172.0 (C), 154.7 (C), 154.4 (C), 153.8 (dd, *J* = 242.7, 6.9 Hz, 2×C), 136.7 (C), 136.3 (C), 134.37 (t, *J* = 16.1 Hz, C), 130.57 (t, *J* = 7.7 Hz, C), 111.95 (dd, *J* = 16.8, 5.8 Hz, 2×CH), 45.4 (CH_2), 43.3 (CH_2), 35.4 (CH_3), 34.8 (CH_2). **IR** (solid): 3304, 2920, 2852, 1627, 1528 cm^{-1}. **LRMS (ESI)** 576 (50, $[M^{81}Br^{81}Br+H]^+$), 578 (100, $[M^{79}Br^{81}Br+H]^+$), (50, $[M^{79}Br^{79}Br+H]^+$); **HRMS (ESI)** calcd for $C_{20}H_{17}Br_2F_2N_3O_5$ $[M^{79}Br^{81}Br+H]^+$ 577.9561; observed 577.9555.

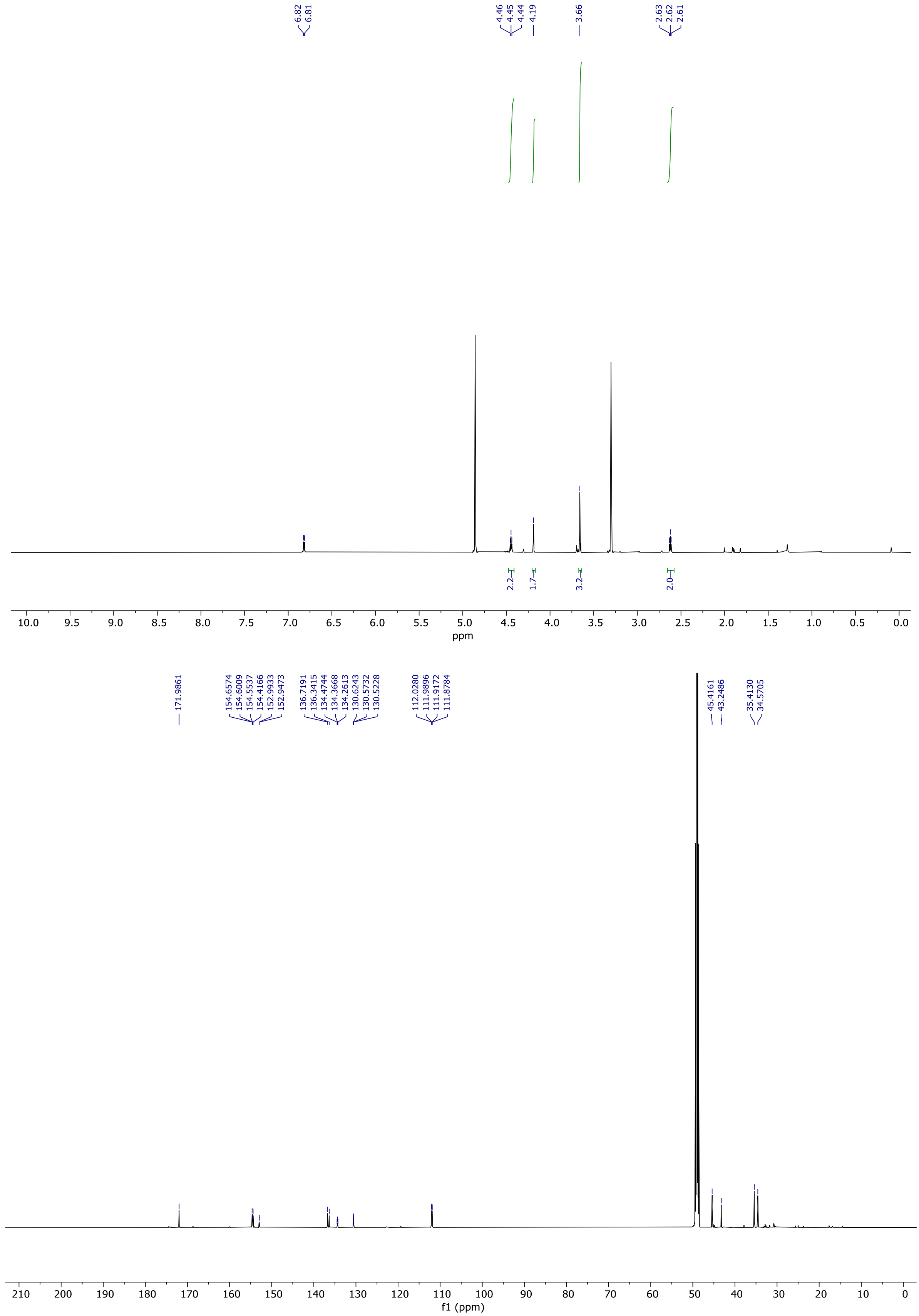

Figure S21: ^{1}H and ^{13}C NMR for reagent **S15**.

4-((3-(4,5-Dibromo-2-methyl-3,6-dioxo-3,6-dihydropyridazin-1(2*H*)-yl)propanamido)methyl)-2,6-difluorophenyl 6-azidohexanoate **6**

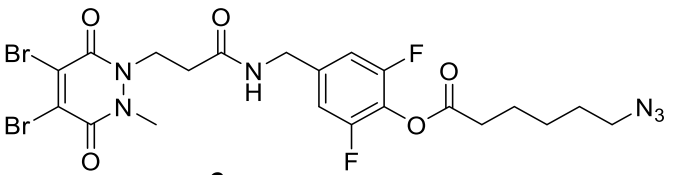

6

To a solution of 6-azidohexanoic acid **S6** (63 mg, 0.40 mmol) in anh. DMF (5 mL) was added EDC·HCl (115 mg, 0.60 mmol) and DMAP (37 mg, 0.30 mmol) under argon. The reaction mixture was stirred at 0 °C for 30 mins. After this time, to this solution, was added dropwise a pre-mixed solution of 3-(4,5-dibromo-2-methyl-3,6-dioxo-3,6-dihydropyridazin-1(2*H*)-yl)-*N*-(3,5-difluoro-4-hydroxybenzyl)propenamide **S15** (100 mg, 0.20 mmol) and NEt_3 (42 µL, 0.30 mmol) in anh. DMF (3 mL). After this, the resulting reaction mixture was stirred at 21 °C for 16 h under argon. The reaction mixture was then concentrated *in vacuo* with toluene co-evaporation (3 × 30 mL, as an azeotrope) and the crude residue purified by flash column chromatography (20% to 100% Cyclohexane/EtOAc) to afford 4-((3-(4,5-dibromo-2-methyl-3,6-dioxo-3,6-dihydropyridazin-1(2*H*)-yl)propanamido)methyl)-2,6-difluorophenyl 6-azidohexanoate **6** (25 mg, 0.039 mmol, 20%) as a yellow oil. **^{1}H NMR** (600 MHz, MeOD) δ 7.01 (d, *J* = 8.4 Hz, 1H), 4.46 (t, *J* = 6.9 Hz, 1H), 4.30 (s, 1H), 3.66 (s, 2H), 3.32 (d, *J* = 6.8 Hz, 1H), 2.74 – 2.59 (m, 2H), 1.77 (p, *J* = 7.3 Hz, 1H), 1.66-1.62 (m, *J* = 9.3, 2H), 1.54 – 1.46 (m, 1H). **^{13}C NMR** (151 MHz, MeOD) δ 172.2 (C), 171.5 (C), 156.31 (dd, *J* = 249.4, 4.6 Hz, 2×C), 154.6 (C), 154.4 (C), 139.90 (t, *J* = 8.0 Hz, C), 136.7 (C), 136.3 (C), 127.2 (t, *J* = 16.4 Hz, C), 112.17 (dd, *J* = 19.0, 4.1 Hz, 2×CH), 52.2 (CH_2), 45.3 (CH_2), 43.2 (CH_2), 35.4 (CH_3), 34.5 (CH_2), 33.9 (CH_2), 29.5 (CH_2), 27.1 (CH_2), 25.5 (CH_2). **IR** (thin film): 3325, 2929, 2859, 2096, 1772, 1632, 1514 cm^{-1}. **LRMS (ESI)** 639 (50, $[M^{81}Br^{81}Br+H]^+$), 637 (100, $[M^{79}Br^{81}Br+H]^+$), 635 (50, $[M^{79}Br^{79}Br+H]^+$); **HRMS (ESI)** calcd for $C_{21}H_{22}Br_2F_2N_6O_5$ $[M^{79}Br^{81}Br+H]^+$ 637.0039; observed 637.0044.

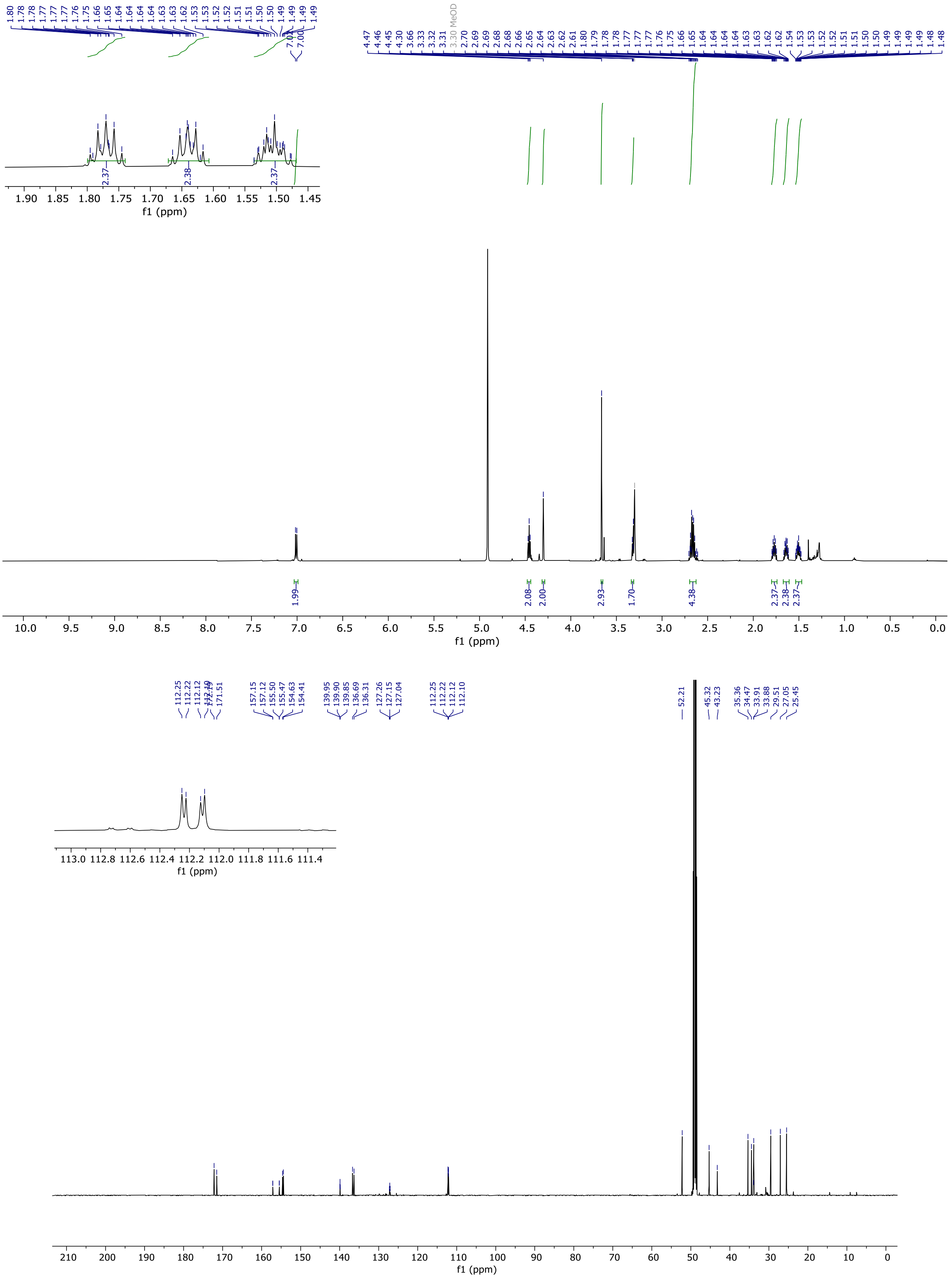

Figure S22: ^{1}H and ^{13}C NMR for reagent **6**.

1.3 Synthesis of reagent **7**

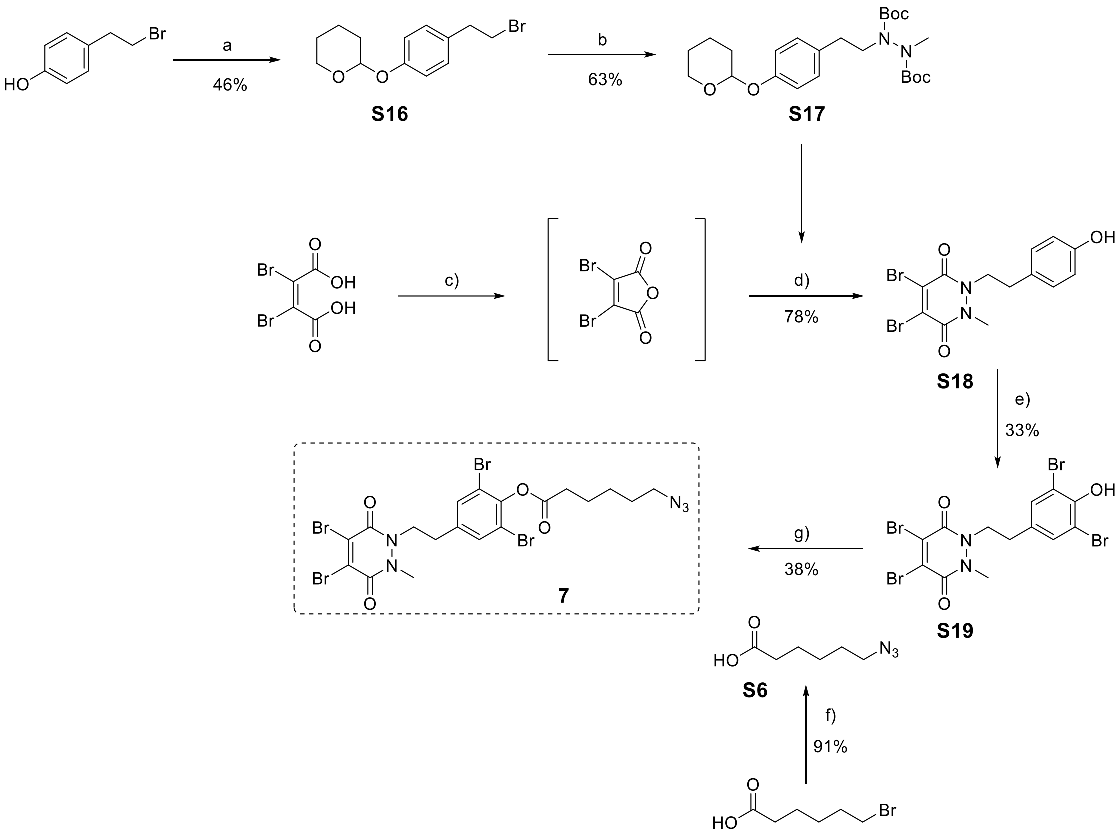

Scheme S3: Synthesis of reagent **7**. Reagents and conditions: a) PPTS, 3,4-dihydro-2*H*-pyran, DCM, 16 h, b) NaH (60% in mineral oil), KI, DMF, 85 °C, 1 h, c) AcOH, reflux, d) Reagent **S14**, AcOH, reflux, 2.5 h, e) NBS, THF, 0°C, 4 h, f) 6-bromohexanoic acid, NaN_3, Acetone:H_2O (50:50), 16 h, g) EDC·HCl,DMAP, NEt_3, THF:DMF (5:1), 16 h.

2-(4-(2-bromoethyl)phenoxy)tetrahydro-2*H*-pyran **S16**[4]

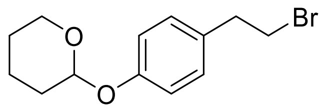

S16

To a solution of 4-(2-bromoethyl)phenol (200 mg, 0.99 mmol) in DCM (2 mL) was added 3,4-Dihydro-2*H*-pyran (125 mg, 1.49 mmol) and pyridinium *p*-toluenesulfonate (25 mg, 0.099 mmol). Solution was stirred at 21 °C for 16 h. The reaction mixture was then extracted with DCM (3 × 10 mL) and brine (10 mL). The combined organic phases were dried ($MgSO_4$), filtered and the solvent removed *in vacuo* to afford (4-(2-bromoethyl)phenoxy)tetrahydro-2*H*-pyran **S16** (130 mg, 0.46 mmol, 46%) as a colourless oil. **^{1}H NMR** (600 MHz, $CDCl_3$) δ 7.16 – 7.08 (m, 2H), 7.04 – 6.94 (m, 2H), 5.40 (t, J = 3.4 Hz, 1H), 3.93 – 3.89 (m, 1H), 3.62 – 3.58 (m, 1H), 3.53 (t, J = 7.8 Hz, 2H), 3.10 (t, J = 7.7 Hz, 2H), 2.04 – 1.97 (m, 1H), 1.90 – 1.80 (m, 2H), 1.72 – 1.64 (m, 2H), 1.63 – 1.55 (m, 1H). **^{13}C NMR** (151 MHz, $CDCl_3$) δ 156.06 (C), 132.0 (C), 129.7 (2×CH), 116.6 (2×CH), 96.4 (CH), 62.1 (CH_2), 38.8 (CH_2), 33.4 (CH_2), 30.4 (CH_2), 25.3 (CH_2), 18.9 (CH_2). **IR** (thin film): 2941, 2871, 1707, 1508, 1233 cm^{-1}.

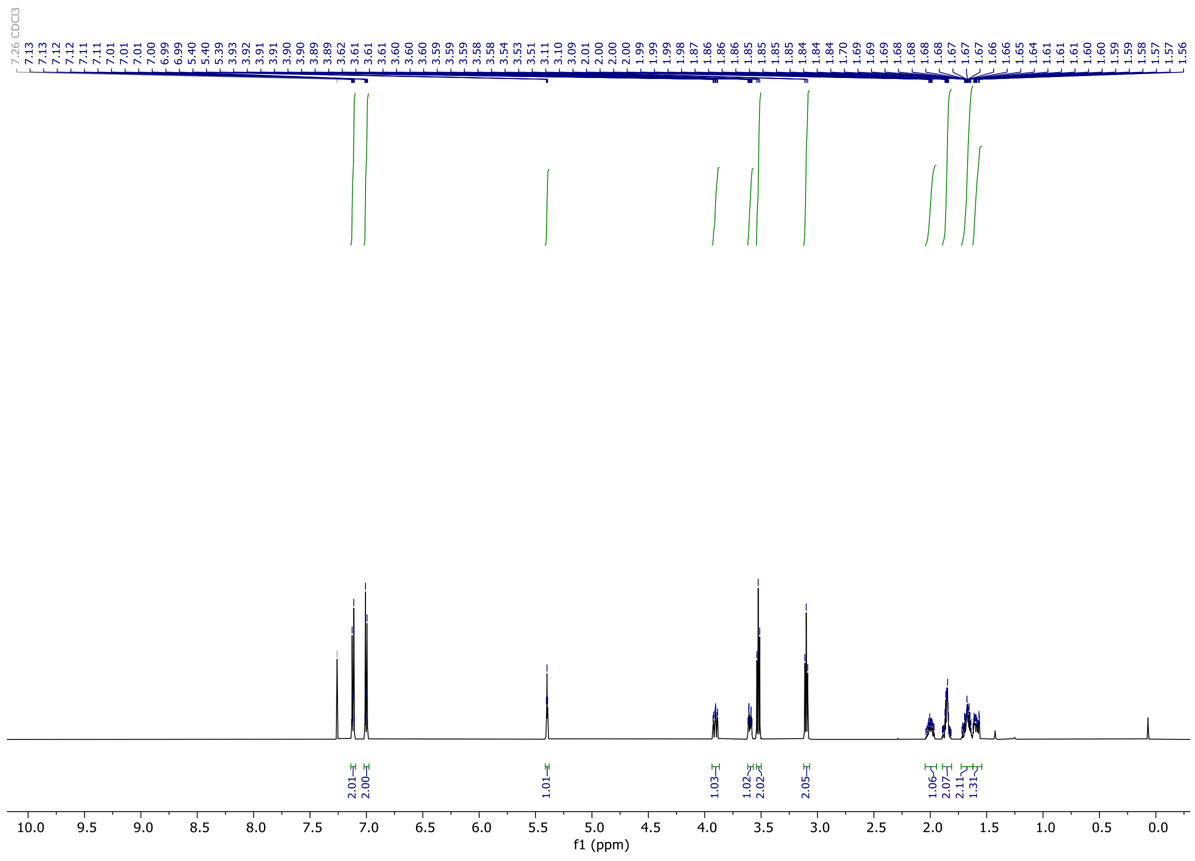

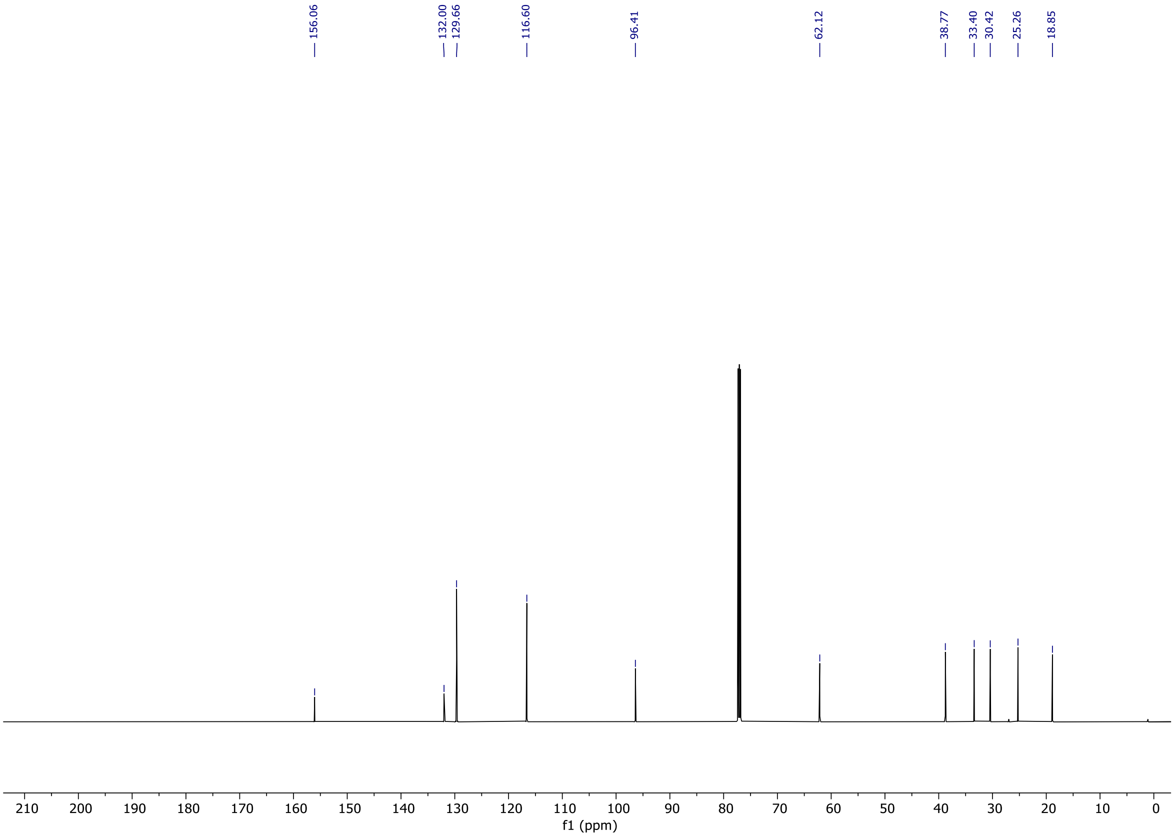

Figure S23: ^{1}H and ^{13}C NMR for reagent **S16**.

Di-*tert*-butyl 1-methyl-2-(4-((tetrahydro-2*H*-pyran-2-yl)oxy)phenethyl)hydrazine-1,2-dicarboxylate **S17**

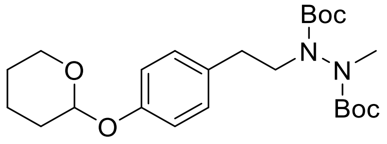

S17

To a solution of di-*tert*-butyl-1-methylhydrazine-1,2-dicarboxylate **S1** (365 mg, 1.48 mmol) in DMF (1.5 mL) was added NaH (60%, 59 mg, 2.47 mmol) and stirred at 21 °C for 10 mins. After this time, 2-(4-(2-bromoethyl)phenoxy)tetrahydro-2*H*-pyran **S16** (210 mg, 0.74 mmol) in DMF (3.5 mL) and KI (12 mg, 0.07 mmol) was added. The reaction was heated at 85 °C for 1 h. The reaction mixture was then concentrated *in vacuo* with toluene co-evaporation (3 × 30 mL, as an azeotrope). The crude residue was then purified by flash column chromatography (0% to 80% Cyclohexane/EtOAc) to afford di-*tert*-butyl 1-methyl-2-(4-((tetrahydro-2*H*-pyran-2-yl)oxy)phenethyl)hydrazine-1,2-dicarboxylate **S17** (130 mg, 0.29 mmol, 63%) as a colourless oil. **^{1}H NMR** (600 MHz, $CDCl_3$, regioisomers) δ 7.13 – 7.09 (m, 2H), 7.01 – 6.90 (m, 2H), 5.38 (t, *J* = 3.5 Hz, 1H), 3.95 – 3.65 (m, 2H), 3.62 – 3.37 (m, 2H), 3.07 – 2.76 (m, 3H), 2.05 – 1.77 (m, 4H), 1.73 – 1.55 (m, 4H), 1.58 – 1.29 (m, 18H). **^{13}C NMR** (151 MHz, $CDCl_3$) δ 156.2 (C), 155.6 (C), 155.6 (C), 132.0 (C), 129.7 (2×CH), 116.6 (2×CH), 96.5 (CH), 80.9 (CH), 62.1 (CH_2), 51.0 (CH_2), 36.6 (CH_3), 33.5 (CH_2), 30.4 (CH_2), 28.4 (3×CH_3), 27.0 (CH_2), 25.3 (CH_2), 18.9 (CH_2). **IR** (thin film): 2974, 2936, 2875, 1707, 1477, 1365, 1147, 1126 cm^{-1}. **LRMS (ESI)** 451 (100, $[M+H]^+$), **HRMS (ESI)** calcd for $C_{24}H_{38}N_2O_6$ $[M+H]^+$ 451.2808; observed 451.2803.

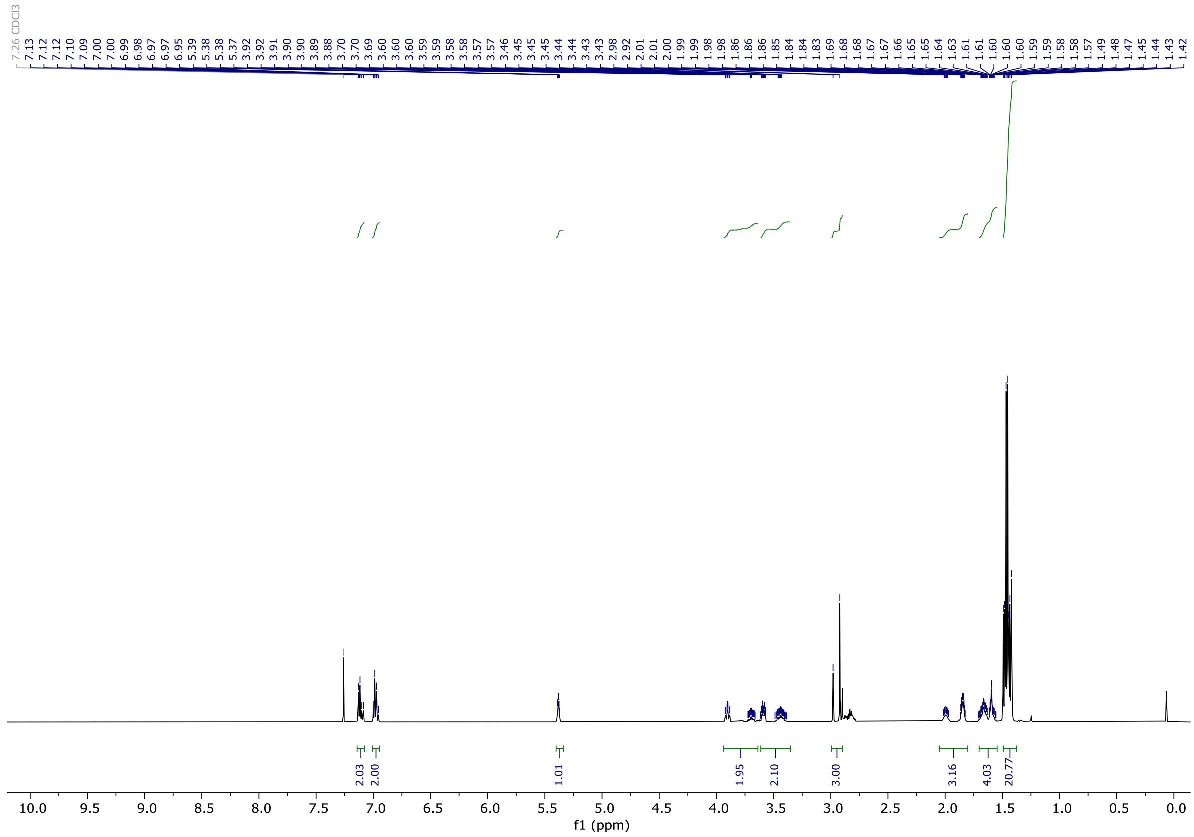

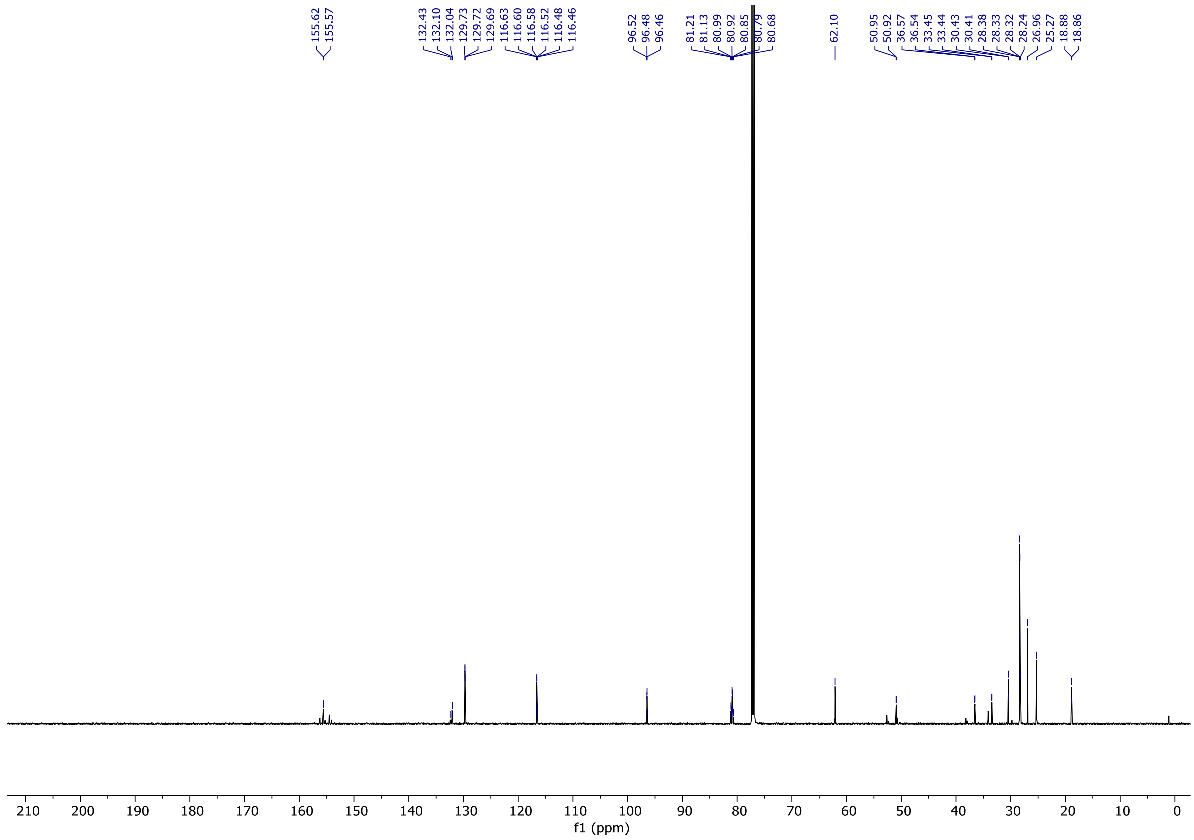

Figure S24: ^{1}H and ^{13}C NMR for reagent **S17**.

4,5-dibromo-1-(4-hydroxyphenethyl)-2-methyl-1,2-dihydropyridazine-3,6-dione **S18**

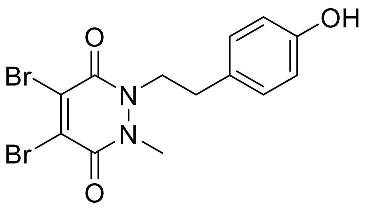

S18

Dibromomaleic acid (63 mg, 0.23 mmol) was dissolved in AcOH (1 mL) and heated under reflux for 30 min. To this solution, was added di-*tert*-butyl 1-methyl-2-(4-((tetrahydro-2*H*-pyran-2-yl)oxy)phenethyl)hydrazine-1,2-dicarboxylate **S17** (120 mg, 0.27 mmol) and the reaction heated under reflux for a further 2.5 h. After this time, the reaction mixture was concentrated *in vacuo* with toluene co-evaporation (3 × 30 mL, as an azeotrope) and the crude residue was purified by flash column chromatography (0% to 60% EtOAc/cyclohexane) to afford 4,5-dibromo-1-(4-hydroxyphenethyl)-2-methyl-1,2-dihydropyridazine-3,6-dione **S18** (72 mg, 0.18 mmol, 78%) as a yellow solid. **^{1}H NMR** (600 MHz, CD_3CN) δ 7.00 (d, *J* = 8.4 Hz, 2H), 6.84 (s, 1H), 6.70 (d, *J* = 8.5 Hz, 2H), 3.50 (s, 3H), 4.26 (t, *J* = 7.3 Hz, 2H) 2.84 (t, *J* = 7.3 Hz, 2H). **^{13}C NMR** (151 MHz, CD_3CN) δ 156.7 , 154.0, 153.8, 136.2 136.2, 131.1 (C), 129.4 (C), 116.1 (CH), 49.7 (CH_2), 35.4 (CH_3) , 33.6 (CH_2). IR (solid): 3339, 1623, 1515 cm^{-1}. **IR** (solid): 3339, 2929, 1622, 2769, 1637, 1515 cm^{-1}. **LRMS (ESI)** 407 (50, [$M^{81}Br^{81}Br+H]^+$), 405 (100, [$M^{79}Br^{81}Br+H]^+$), 403 (50, [$M^{79}Br^{79}Br+H]^+$); **HRMS (ESI)** calcd for $C_{13}H_{12}Br_2N_2O_3$ [$M^{79}Br^{81}Br+H]^+$ 404.9272; observed 404.9267.

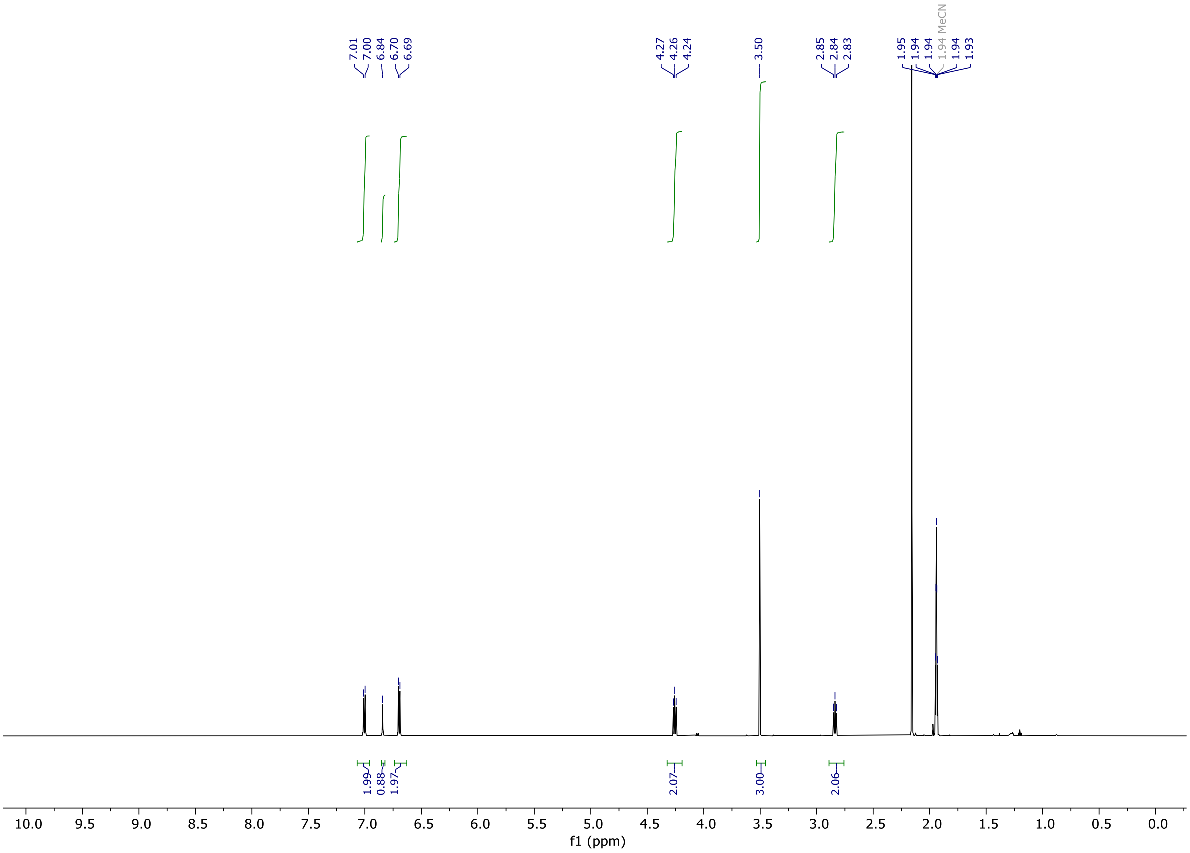

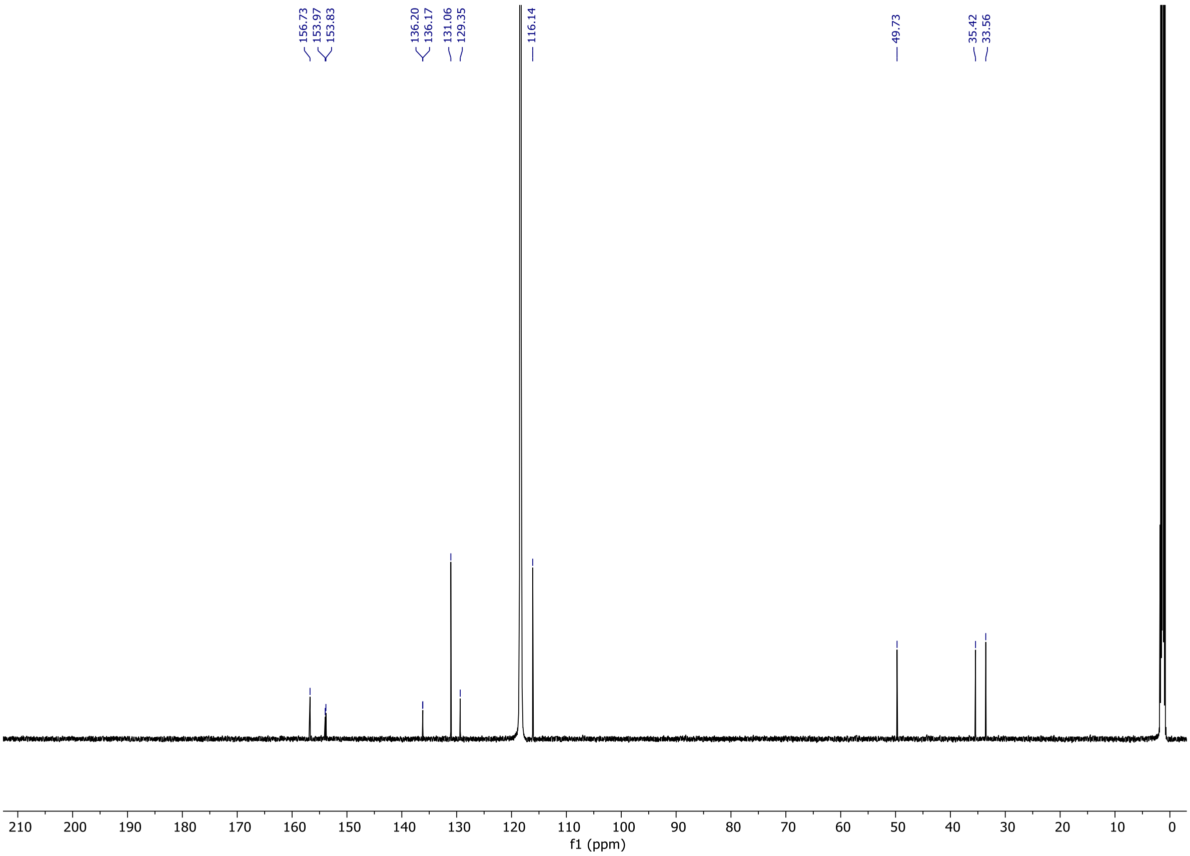

Figure S25: ^{1}H and ^{13}C NMR for reagent **S18**.

4,5-Dibromo-1-(3,5-dibromo-4-hydroxyphenethyl)-2-methyl-1,2-dihydropyridazine-3,6-dione **S19**

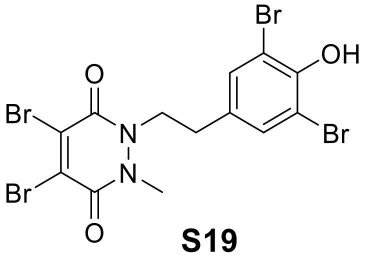

S19

To a solution of 4,5-dibromo-1-(4-hydroxyphenethyl)-2-methyl-1,2-dihydropyridazine-3,6-dione **S18** (69 mg, 0.17 mmol) in THF (6 mL) was added NBS (77 mg, 0.43 mmol) at 0 °C. The reaction was stirred at 0 °C for 4 h. After this time, the reaction mixture was concentrated *in vacuo*. The crude mixture was re-dissolved in DCM (10 mL) and the organic layer was washed with $Na_2O_2S_2$ (3 × 15 mL). The combined organic layers were dried with $MgSO_4$, filtered and the solvent removed *in vacuo*. The crude residue was purified by flash column chromatography (0% to 60% EtOAc/cyclohexane) to afford 4,5-dibromo-1-(3,5-dibromo-4-hydroxyphenethyl)-2-methyl-1,2-dihydropyridazine-3,6-dione **S19** (42 mg, 0.06 mmol, 33%) as a yellow solid. **^{1}H NMR** (500 MHz, CD_3CN) δ 7.30 (s, 2H), 7.00 (s, 1H), 4.28 (t, *J* = 7.0 Hz, 2H), 3.53 (s, 3H), 2.83 (t, *J* = 7.0 Hz, 2H). **^{13}C NMR** (151 MHz, MeOD) δ 154.7, 154.7, 151.4, 136.4, 136.3, 133.8, 132.3, 112.2, 49.7 (CH_3), 35.4 (CH_2), 33.1 (CH_2). **IR** (solid): 3350, 2920, 2851, 1616 cm^{-1}. **LRMS (ESI)** 567 (16, $[M^{81}Br_4+H]^+$), 565 (16, $[M^{79}Br^{81}Br_3+H]^+$), 563 (100, $[M^{79}Br_2{}^{81}Br_2+H]^+$), 561 (69, $[M^{79}Br_3{}^{81}Br+H]^+$); 559 (18, $[M^{79}Br_4+H]^+$), **HRMS (ESI)** calcd for $C_{13}H_{10}Br_4N_2O_3$ $[M^{79}Br_2{}^{81}Br_2+H]^+$ 562.7462; observed 562.7457.

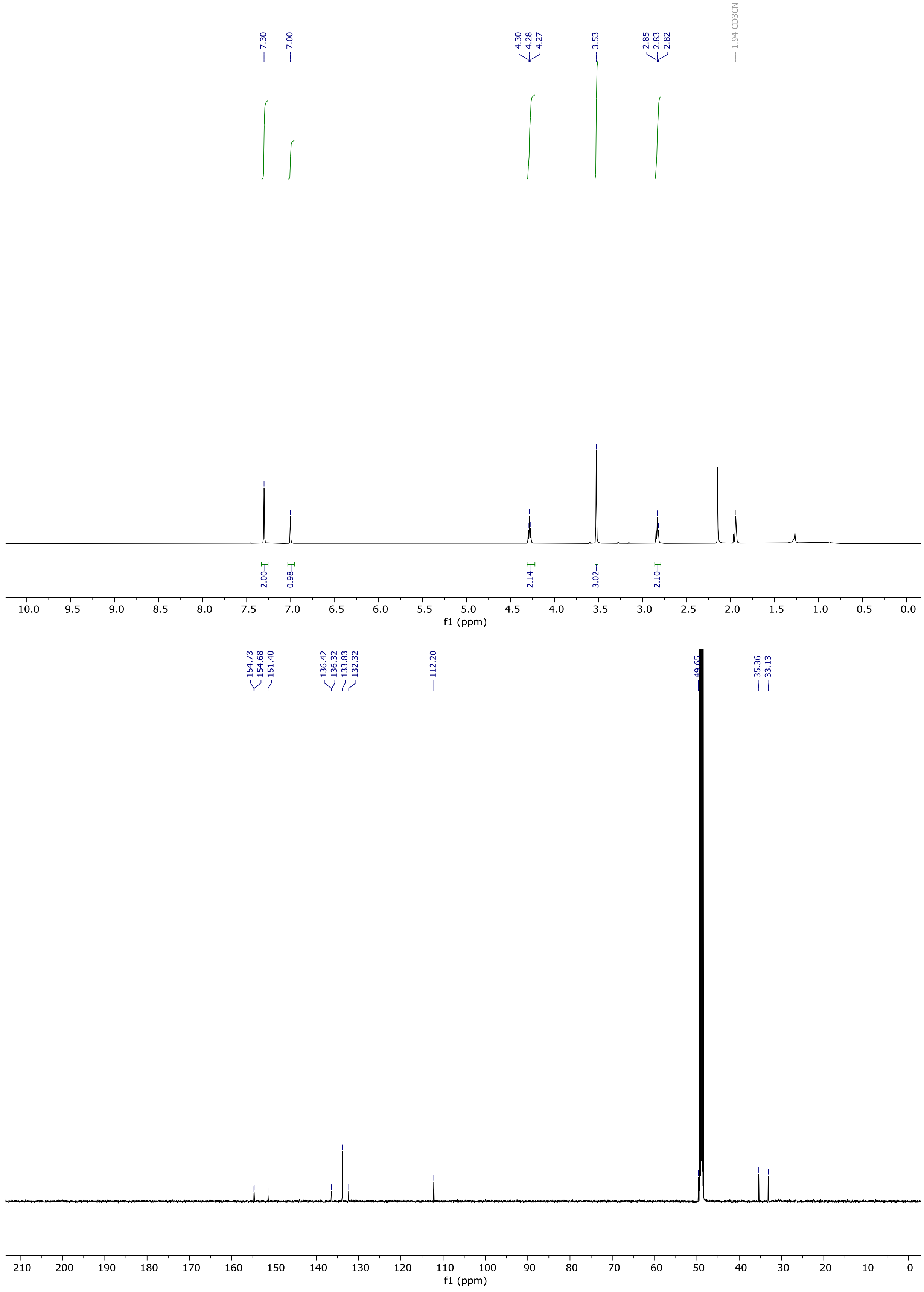

Figure S26: ^{1}H and ^{13}C NMR for reagent **S19**.

2,6-Dibromo-4-(2-(4,5-dibromo-2-methyl-3,6-dioxo-3,6-dihydropyridazin-1(2H)-yl)ethyl)phenyl 6-azidohexanoate **7**

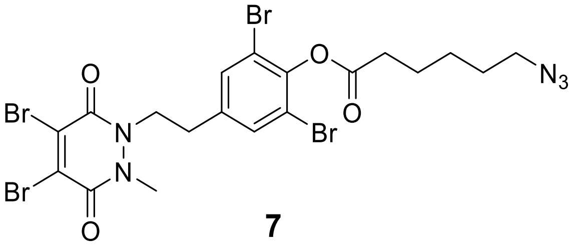

7

To a solution of 6-azidohexanoic acid **S6** (89 mg, 0.16 mmol) in anh. THF (1.5 mL) and DMF (0.5 mL) was added EDC·HCl (35 mg, 0.18 mmol), and the reaction mixture was stirred for 30 mins at 0 °C under argon. After this time, to this solution, was added dropwise a pre-mixed solution of 4,5-dibromo-1-(3,5-dibromo-4-hydroxyphenethyl)-2-methyl-1,2-dihydropyridazine-3,6-dione **S19** (89 mg, 0.16 mmol) and NEt_3 (46 µL, 0.33 mmol) in anh. DMF (5 mL). Upon completion, the reaction mixture was concentrated *in vacuo* with toluene co-evaporation (3 × 30 mL, as an azeotrope). The crude residue was purified by flash column chromatography (0% to 10% DCM/MeOH) to afford 2,6-dibromo-4-((3-(4,5-dibromo-3,6-dioxo-2-phenyl-3,6-dihydropyridazin-1(2*H*)-yl)propanamido)methyl)phenyl 6-azidohexanoate **7** as a yellow oil (40 mg, 0.06 mmol, 38%). **^{1}H NMR** (600 MHz, CD_3CN) δ 7.49 (s, 1H), 4.32 (t, J = 7.2 Hz, 1H), 3.55 (s, 2H), 3.32 (t, J = 6.8 Hz, 1H), 2.92 (t, J = 7.2 Hz, 1H), 2.68 (t, J = 7.3 Hz, 1H), 1.78 (p, J = 7.4 Hz, 1H), 1.64 (m, 1H), 1.56 – 1.47 (m, 1H). **^{13}C NMR** (151 MHz, CD_3CN) δ 171.1 (C), 154.1 (C), 153.9 (C), 145.8 (C), 139.8 (C), 136.4 (C), 136.1 (C), 134.1 (CH), 118.1 (C), 51.9 (CH_2), 48.7 (CH_2), 35.6 (CH_3), 34.1 (CH_2), 33.3 (CH_2), 29.1 (CH_2), 26.8 (CH_2), 25.0 (CH_2). **IR** (thin film): 2927, 2852, 2097, 1769, 1637 cm^{-1}. **LRMS (ESI)** 706 (21, $[M^{81}Br_4+H]^+$), 704 (65, $[M^{79}Br^{81}Br_3+H]^+$) 702 (100, $[M^{79}Br_2{}^{81}Br_2+H]^+$), 700 (69, $[M^{79}Br_3{}^{81}Br+H]^+$) 698 (18, $[M^{79}Br_4+H]^+$); **HRMS (ESI)** calcd for $C_{17}H_{15}Br_4N_6O_5$ $[M^{79}Br_2{}^{81}Br_2+H]^+$ 701.8208; observed 701.8202.

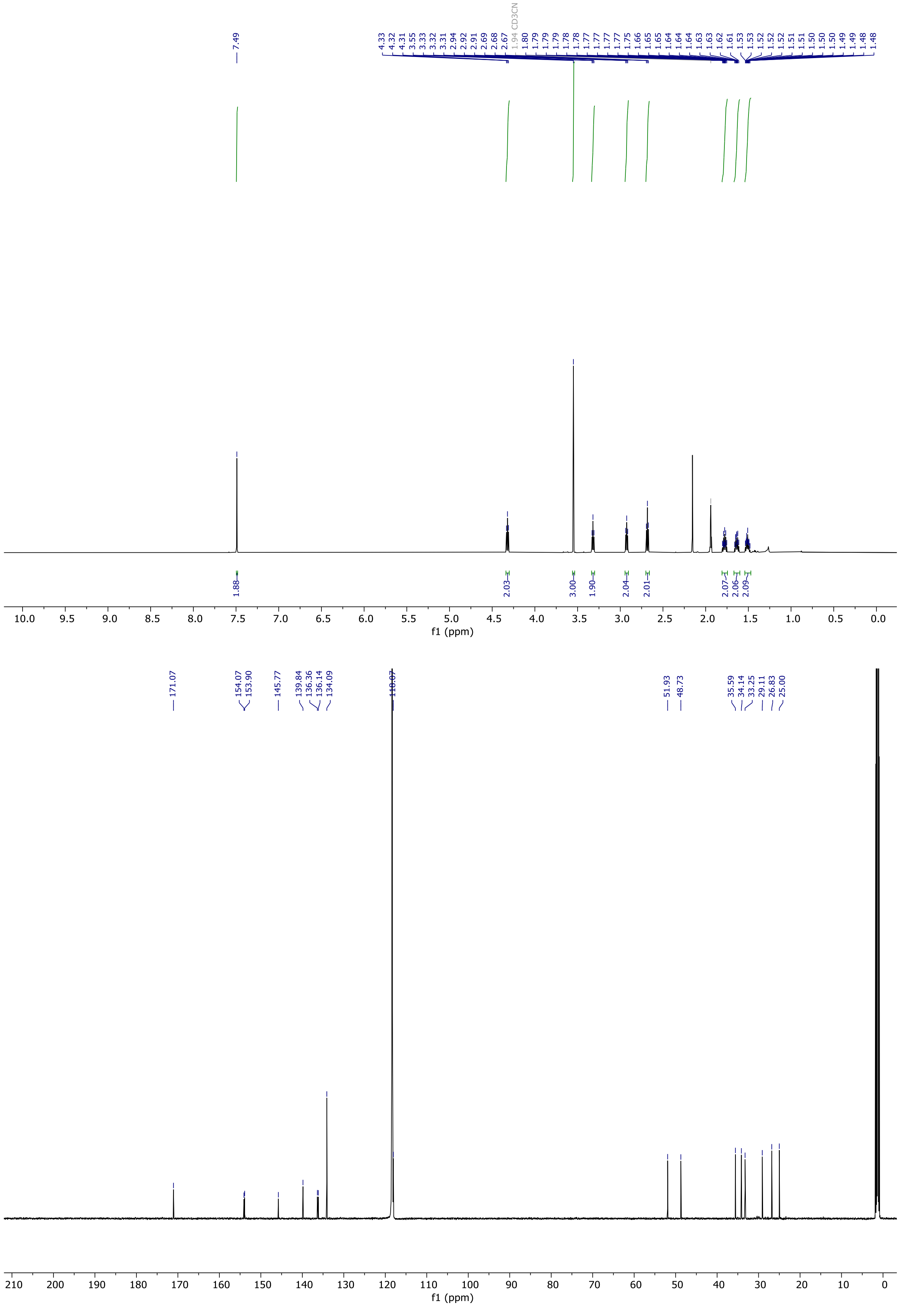

Figure S27: ^{1}H and ^{13}C NMR for reagent **7**.

1.4 Synthesis of reagent **8**

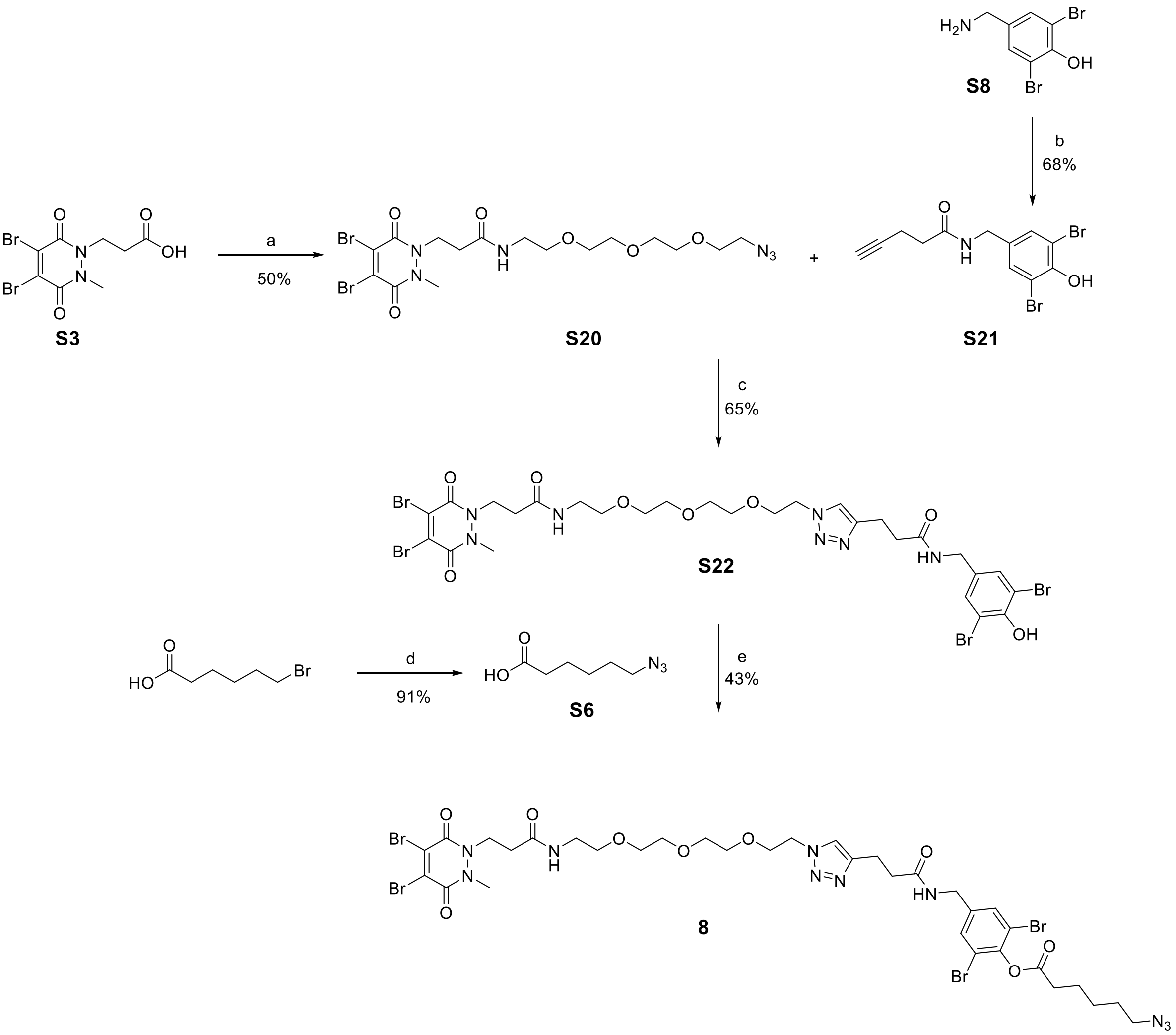

Scheme S4: Synthesis of reagent **8**. Reagents and conditions: a) 2-(2-(2-(2-azidoethoxy)ethoxy)ethoxy)ethan-1-amine, EDC·HCl, NEt_3, anh. THF, 5 h, b) Pent-4-ynoic acid, EDC·HCl, NEt_3, anh. DMF, 16 h c) CuI, DIPEA, THF, 16 h , d) 6-bromohexanoic acid, NaN_3, Acetone:H_2O (50:50), 16 h, e) Reagent **S6**, EDC·HCl, DMAP, NEt_3, anh. DMF, 16 h.

N-(2-(2-(2-(2-azidoethoxy)ethoxy)ethoxy)ethyl)-3-(4,5-dibromo-2-methyl-3,6-dioxo-3,6-dihydropyridazin-1(2H)-yl)propenamide **S20**

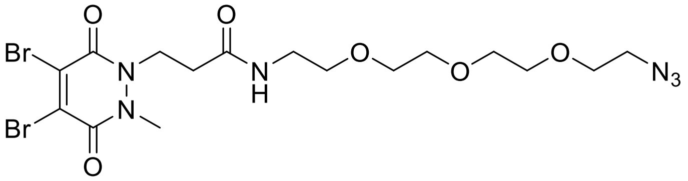

S20

To a solution of 3-(4,5-Dibromo-2-methyl-3,6-dioxo-3,6-dihydropyridazin-1(2H)-yl) propanoic acid **S3** (200 mg, 0.56 mmol) in anh. THF (4 mL) was added EDC·HCl (119 mg, 0.62 mmol). The reaction mixture was then stirred at 0 °C for 30 min under argon. After this time, to this solution, was added dropwise a pre-mixed solution of 2-(2-(2-(2-azidoethoxy)ethoxy)ethoxy)ethan-1-amine (111 µL, 0.56 mmol) in anh. THF (4 mL) and NEt_3 (93 µL, 0.67 mmol). After this, the reaction mixture was stirred for 5 h under argon. After this time, the solvent mixture was concentrated *in vacuo* with toluene co-evaporation (3 × 30 mL toluene, 3 × 30 mL $CHCl_3$ as an azeotrope) and the crude residue was purified by flash column chromatography (0% to 20% DMC/MeOH) to afford *N*-(2-(2-(2-(2-azidoethoxy)ethoxy)ethoxy)ethyl)-3-(4,5-dibromo-2-methyl-3,6-dioxo-3,6-dihydropyridazin-1(2*H*)-yl)propenamide **S20** (211 mg, 0.38 mmol, 68%) as a colourless oil. **^{1}H NMR** (600 MHz, CD_3CN) δ 6.85 – 6.68 (s, 1H, -NH), 4.32 (t, *J* = 7.0 Hz, 2H), 3.61 (t, *J* = 4.9, 2H), 3.59 – 3.54 (m, 9H), 3.53 – 3.51 (m, 2H), 3.42 (t, *J* = 5.6 Hz, 2H), 3.36 (t, *J* = 4.9 Hz, 2H), 3.25 (q, *J* = 5.6 Hz, 2H), 2.49 (t, *J* = 7.0 Hz, 2H). **^{13}C NMR** (151 MHz, CD_3CN) δ 170.5 (C), 154.0 (C), 153.7 (C), 136.6 (C), 136.1 (C), 71.1 (CH_2), 70.9 (CH_2), 70.5 (CH_2), 70.1 (CH_2), 51.5 (CH_2), 45.0 (CH_2), 39.9 (CH_2), 35.6 (CH_3), 34.4 (CH_2). **IR** (thin film): 3337, 2922, 2869, 2105, 1634 cm^{-1}. **LRMS (ESI)** 559 (50, $[M^{81}Br^{81}Br+H]^+$), 557 (100, $[M^{79}Br^{81}Br+H]^+$), 555 (50, $[M^{79}Br^{79}Br+H]^+$); **HRMS (ESI)** calcd for $C_{16}H_{24}Br_2N_6O_6$ $[M^{79}Br^{81}Br+H]^+$ 557.0182; observed 557.0176.

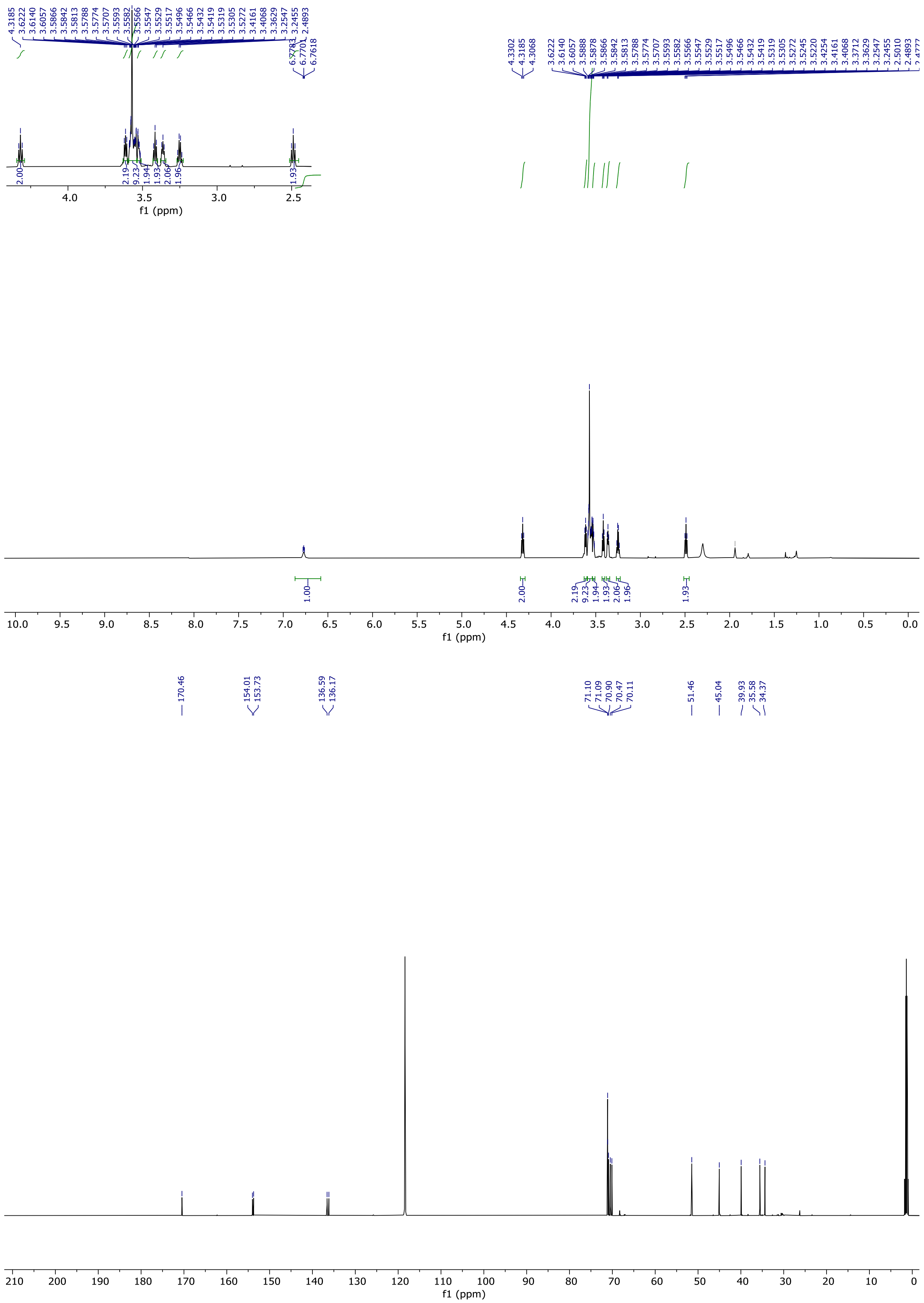

Figure S28: ^{1}H and ^{13}C NMR for reagent **S20**.

N-(3,5-Dibromo-4-hydroxybenzyl)pent-4-ynamide **S21**

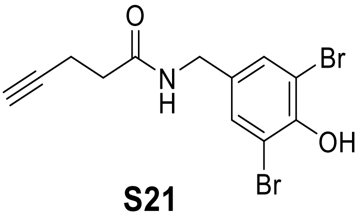

S21

To a solution of 4-pentynoic acid (87 mg, 0.89 mmol) in anh. DMF (16 mL) was added EDC·HCl (257 mg, 1.34 mmol). The reaction mixture was stirred for 30 mins at 0 °C under argon. After this time, to this solution, was added 4-(aminomethyl)-2,6-bromophenol **S8** (500 mg, 1.78 mmol) and the solution was stirred overnight under argon. The reaction mixture was then concentrated *in vacuo* with toluene co-evaporation (3 × 30 mL, as an azeotrope). The crude residue purified was by flash column chromatography (0% to 20% cyclohexane/EtOAc) to afford *N*-(3,5-dibromo-4-hydroxybenzyl)pent-4-ynamide **S21** (190 mg, 0.53 mmol, 59%) as a white solid. **^{1}H NMR** (500 MHz, MeOD) δ 7.42 (s, 2H), 4.25 (s, 2H), 2.50 – 2.47 (m, 2H), 2.43 – 2.40 (m, 2H), 2.25 (t, J = 2.6 Hz, 1H). **^{13}C NMR** (126 MHz, MeOD) δ 174.0 (C), 151.4 (C), 134.2 (CH), 112.1 (C), 83.5 (CH), 70.5 (C), 42.5 (CH_2), 36.0 (CH_2), 15.7 (CH_2). **IR** (solid): 3400, 2914, 2850, 2120, 1640 cm^{-1}. **LRMS (ESI)** 360 (50, [M^{81}Br^{81}Br+H]$^{+}$), 362 (100, [M^{79}Br^{81}Br+H]$^{+}$), 360 (50, [M^{79}Br^{79}Br+H]$^{+}$; **HRMS (ESI)** calcd for $C_{12}H_{11}Br_2NO_2$ [M^{79}Br^{81}Br+H]$^{+}$ 361.9214; observed 361.9209.

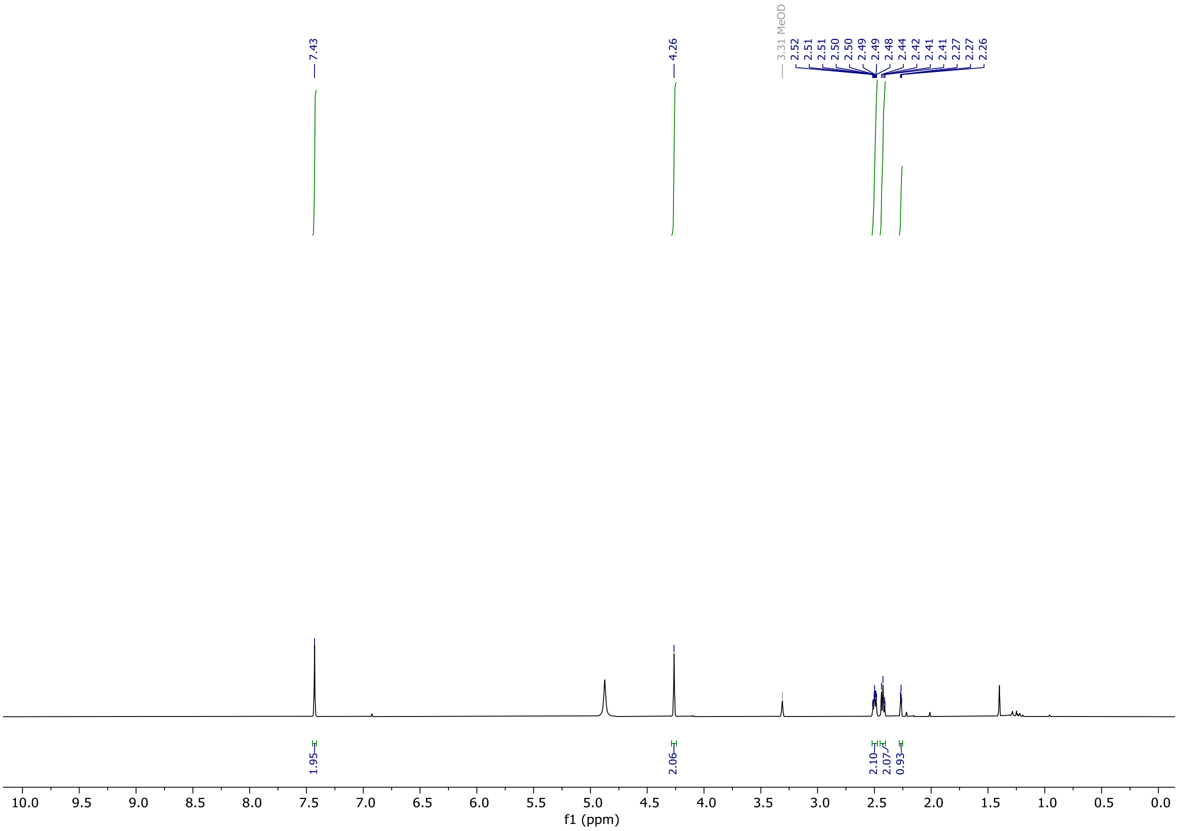

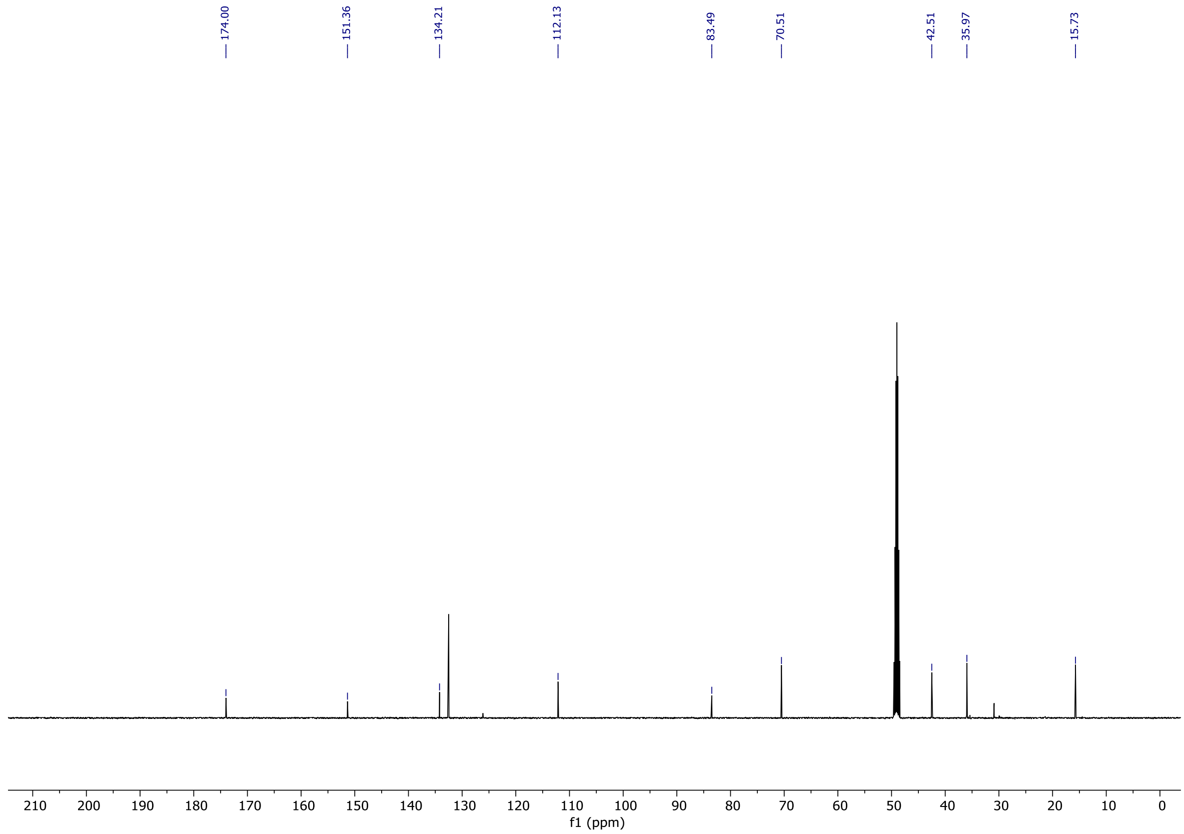

Figure S29: ^{1}H and ^{13}C NMR for reagent **S21**.

3-(4,5-Dibromo-2-methyl-3,6-dioxo-3,6-dihydropyridazin-1(2*H*)-yl)-*N*-(2-(2-(2-(2-(4-(3-((3,5-dibromo-4-hydroxybenzyl)amino)-3-oxopropyl)-1*H*-1,2,3-triazol-1-yl)ethoxy)ethoxy)ethoxy)ethyl)propenamide **S22**

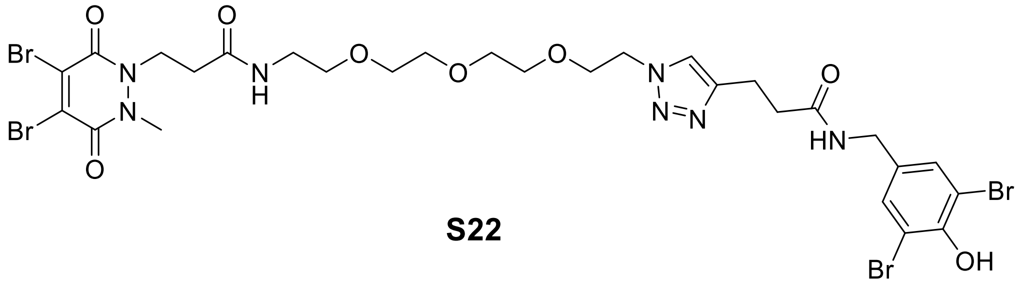

S22

To a solution of *N*-(3,5-dibromo-4-hydroxybenzyl)pent-4-ynamide **S21** (139 mg, 0.25 mmol) in THF (12 mL), was added *N*-(2-(2-(2-(2-azidoethoxy)ethoxy)ethoxy)ethyl)-3-(4,5-dibromo-2-methyl-3,6-dioxo-3,6-dihydropyridazin-1(2*H*)-yl)propenamide **S20** (184 mg, 0.50 mmol), CuI (24 mg, 0.13 mmol), and DIPEA (44 µL, 0.25 mmol). The reaction mixture was stirred at 21 °C for 16 h. The solution was then filtered and the filtrate was concentrated *in vacuo*. The crude residue was purified by flash column chromatography (0% to 20% MeOH/EtOAc AcOH) to afford 3-(4,5-dibromo-2-methyl-3,6-dioxo-3,6-dihydropyridazin-1(2*H*)-yl)-*N*-(2-(2-(2-(2-(4-(3-((3,5-dibromo-4-hydroxybenzyl)amino)-3-oxopropyl)-1*H*-1,2,3-triazol-1-yl)ethoxy)ethoxy)ethoxy)ethyl)propenamide **S22** (148 mg, 0.16 mmol, 65%) as an orange oil. **^{1}H NMR** (600 MHz, CD_3CN) δ 7.59 (s, 1H), 7.37 (s, 2H), 7.13 (s, 1H), 6.99 (s, 1H), 6.86 (s, 1H), 4.44 (t, *J* = 5.1 Hz, 2H), 4.30 (t, *J* = 7.0 Hz, 2H), 4.20 (d, *J* = 6.0 Hz, 2H), 3.79 (t, *J* = 5.1 Hz, 2H), 3.52 – 3.46 (m, 9H), 3.39 (t, *J* = 5.6 Hz, 2H), 3.23 (q, *J* = 5.6 Hz, 2H), 2.93 (t, *J* = 7.2 Hz, 2H), 2.54 (t, *J* = 7.2 Hz, 2H), 2.47 (t, *J* = 7.0 Hz, 2H). **^{13}C NMR** (151 MHz, CD_3CN) δ 170.5 (C), 154.0 (C), 153.8 (C), 150.1 (C), 136.6 (C), 136.2 (C), 135.3 (C), 132.3 (2 × CH), 111.0 (C), 71.0 (CH_2), 71.0 (CH_2), 70.9 (CH_2), 70.9 (CH_2), 70.1 (CH_2), 70.0 (CH_2), 50.8 (CH_2), 45.0 (CH_2), 42.0 (CH_2), 39.9 (CH_2), 36.0 (CH_3), 35.6 (CH_2), 34.4 (CH_2). **IR** (thin film): 3306, 2917, 1633,1552, 1351 cm^{-1}. **LRMS (ESI)** 920 (21, $[M^{81}Br_4+H]^+$), 918 (65, $[M^{79}Br^{81}Br_3+H]^+$), 916 (100, $[M^{79}Br_2{}^{81}Br_2+H]^+$), 914 (69, $[M^{79}Br_3{}^{81}Br+H]^+$), 912 (18, $[M^{79}Br_4+H]^+$); **HRMS (ESI)** calcd $C_{28}H_{35}Br_4N_7O_8$ $[M^{79}Br^{81}Br-H]^-$ 915.9161; observed 915.9170.

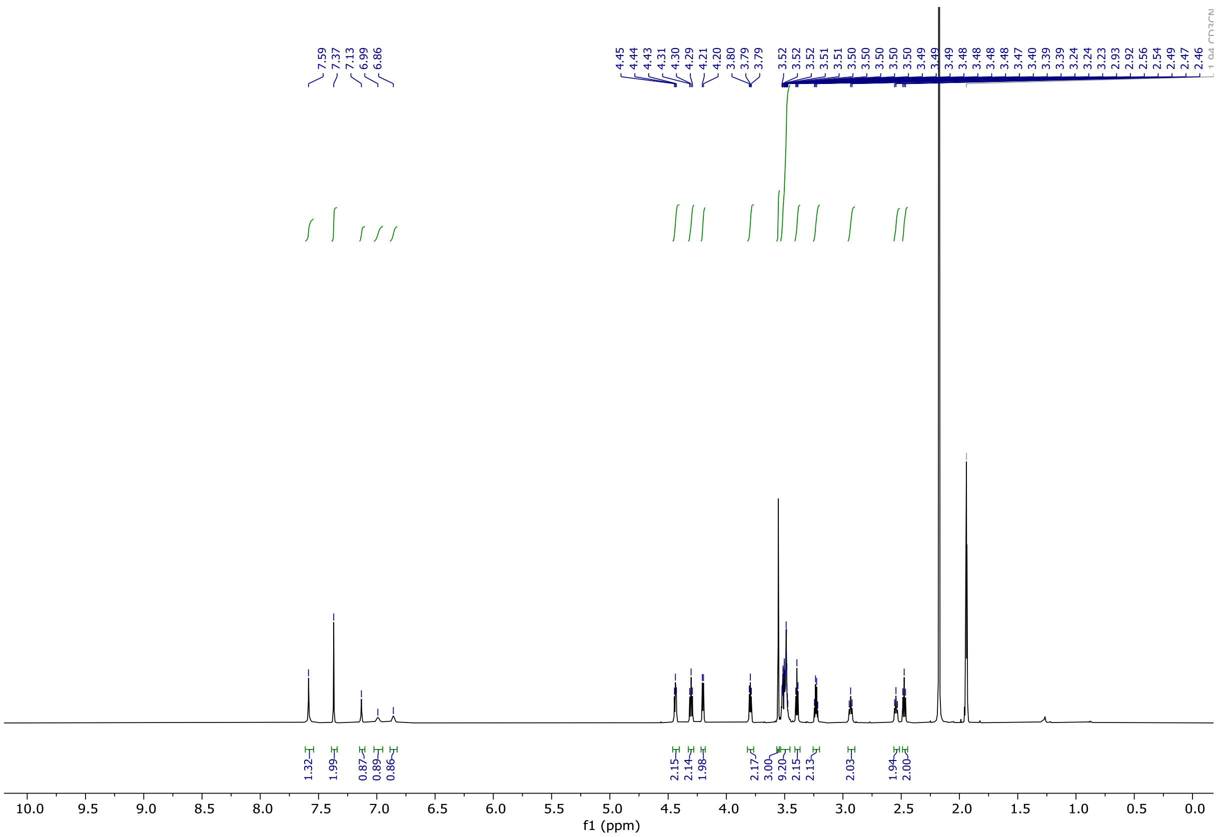

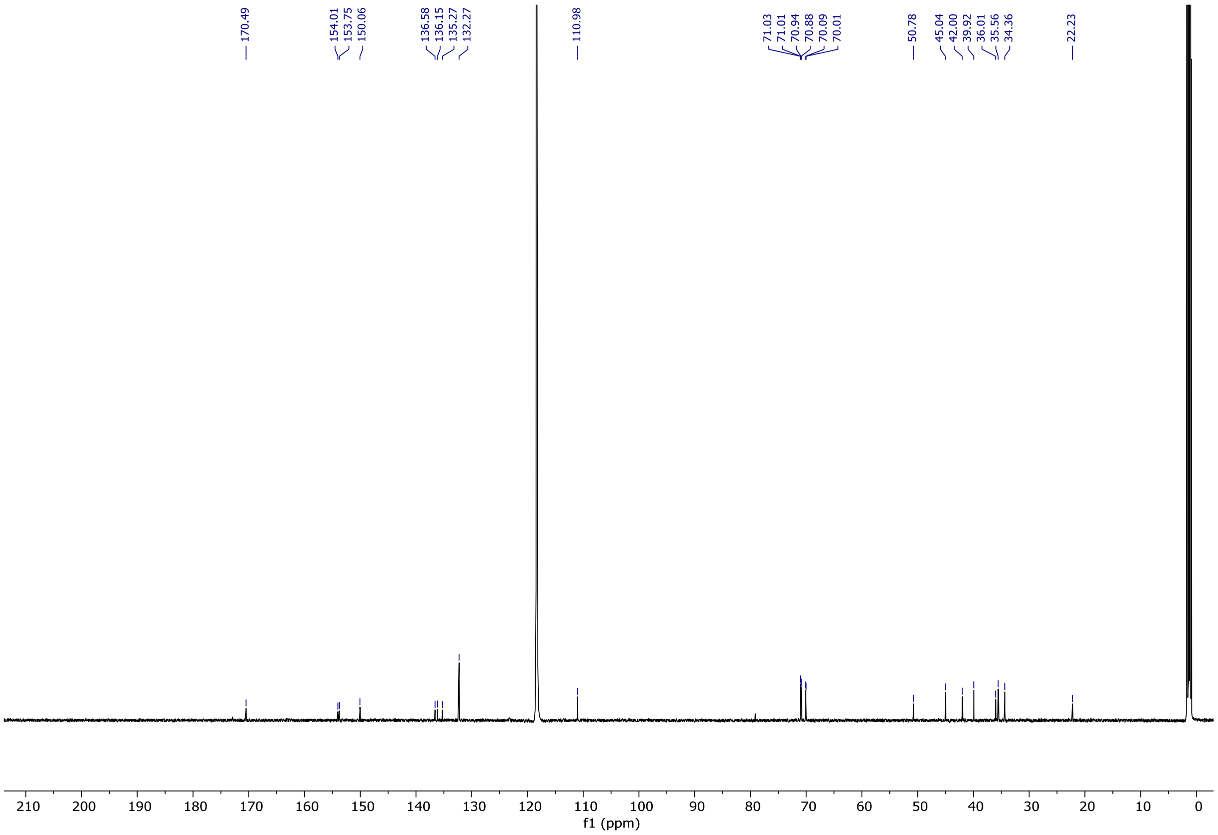

Figure S30: ^{1}H and ^{13}C NMR for reagent **S22**.

2,6-Dibromo-4-((3-(1-(15-(4,5-dibromo-2-methyl-3,6-dioxo-3,6-dihydropyridazin-1(2*H*)-yl)-13-oxo-3,6,9-trioxa-12-azapentadecyl)-1*H*-1,2,3-triazol-4-yl)propanamido)methyl)phenyl 6-azidohexanoate **8**

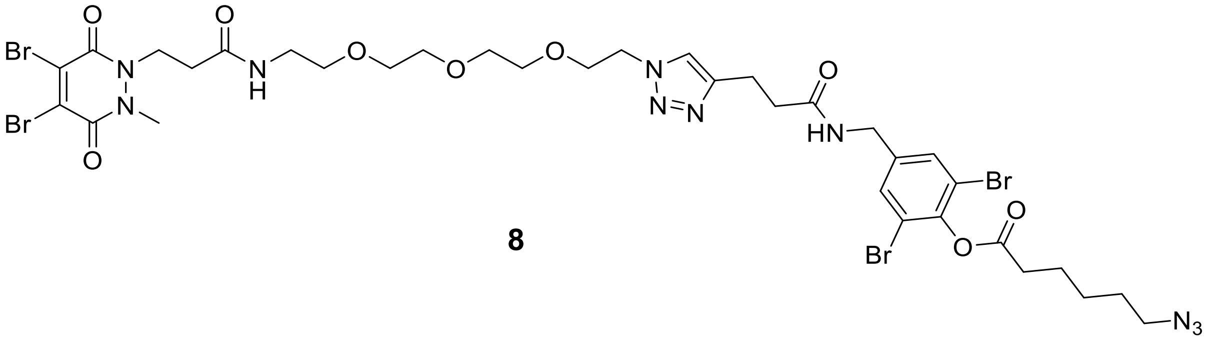

8

To a solution of 6-azidohexanoic acid **S6** (22 mg, 0.14 mmol) in anh. THF (3 mL) was added EDC·HCl (40 mg, 0.21 mmol) and DMAP (17 mg, 0.14 mmol). The reaction mixture was then stirred for 30 min at 0 °C under argon. After this time, to this solution, was added dropwise a pre-mixed solution of 3-(4,5-dibromo-2-methyl-3,6-dioxo-3,6-dihydropyridazin-1(2*H*)-yl)-*N*-(2-(2-(2-(2-(4-(3-((3,5-dibromo-4-hydroxybenzyl)amino)-3-oxopropyl)-1*H*-1,2,3-triazol-1-yl)ethoxy)ethoxy)ethoxy)ethyl)propenamide **S22** (140 mg, 0.15 mmol) and NEt_3 (39 µL, 0.38 mmol) in anh. DMF (5 mL). After this, the reaction mixture was stirred at 21 °C for 16 h under argon. The reaction mixture was then concentrated *in vacuo* with toluene co-evaporation (3 × 30 mL, as an azeotrope). The crude residue was purified by flash column chromatography (0% to 20% MeOH/EtOAc) to afford 2,6-dibromo-4-((3-(1-(15-(4,5-dibromo-2-methyl-3,6-dioxo-3,6-dihydropyridazin-1(2*H*)-yl)-13-oxo-3,6,9-trioxa-12-azapentadecyl)-1*H*-1,2,3-triazol-4-yl)propanamido)methyl)phenyl 6-azidohexanoate **8** (60 mg, 0.06 mmol, 43%) as a brown oil. **^{1}H NMR** (600 MHz, CD_3CN) δ 7.60 (s, 1H), 7.51 (s, 2H), 7.21 (t, *J* = 6.1 Hz, 1H), 6.96 (t, *J* = 5.8 Hz, 1H), 4.45 (t, *J* = 5.1 Hz, 2H), 4.30 (t, *J* = 6.9 Hz, 2H), 3.80 (t, *J* = 5.1 Hz, 2H), 3.55 (s, 3H), 3.53 – 3.45 (m, 8H), 3.40 (t, *J* = 5.6 Hz, 2H), 3.32 (t, *J* = 6.8 Hz, 2H), 3.23 (q, *J* = 5.6 Hz, 2H), 2.95 (t, *J* = 7.5 Hz, 2H), 2.68 (t, *J* = 7.3 Hz, 2H), 2.58 (t, *J* = 7.4 Hz, 2H), 2.48 (t, *J* = 7.0 Hz, 1H), 1.77 (p, *J* = 7.4 Hz, 2H), 1.67 – 1.60 (m, 2H), 1.55 – 1.45 (m, 2H). **^{13}C NMR** (151 MHz, CD_3CN) δ 173.1 (C), 171.2 (C), 170.6 (C), 154.0 (C), 153.7 (C), 147.2 (C), 145.6 (C), 141.9 (C), 136.6 (C), 136.1 (C), 132.2 (2 × CH), 123.2 (CH), 118.0 (2 × C), 71.0 (CH_2), 70.9 (CH_2), 70.9 (CH_2), 70.8 (CH_2), 70.2 (CH_2), 70.0 (CH_2), 51.9 (CH_2), 50.8 (CH_2), 45.0 (CH_2), 42.1 (CH_2), 39.9 (CH_2), 35.9 (CH_2), 35.5 (CH_3), 34.4 (CH_2), 34.1 (CH_2), 29.1(CH_2), 26.8 (CH_2), 25.0 (CH_2), 22.2 (CH_2). IR (thin film): 3306, 2925, 2867, 2095, 1769, 1633, 1117 cm^{-1}.

LRMS (ESI) 1061 (50, $[M^{81}Br_4+H]^+$), 1059 (24, $[M^{79}Br^{81}Br_3+H]^+$), 1057 (100, $[M^{79}Br_2{}^{81}Br_2+H]^+$),

1055 (70, $[M^{79}Br_3{}^{81}Br+H]^+$), 1053 (18, $[M^{79}Br_4+H]^+$); **HRMS (ESI)** calcd for $C_{34}H_{44}Br_4N_{10}O_9$ $[M^{79}Br^{81}Br+H]^+$ 1057.0064; observed 1057.0058.

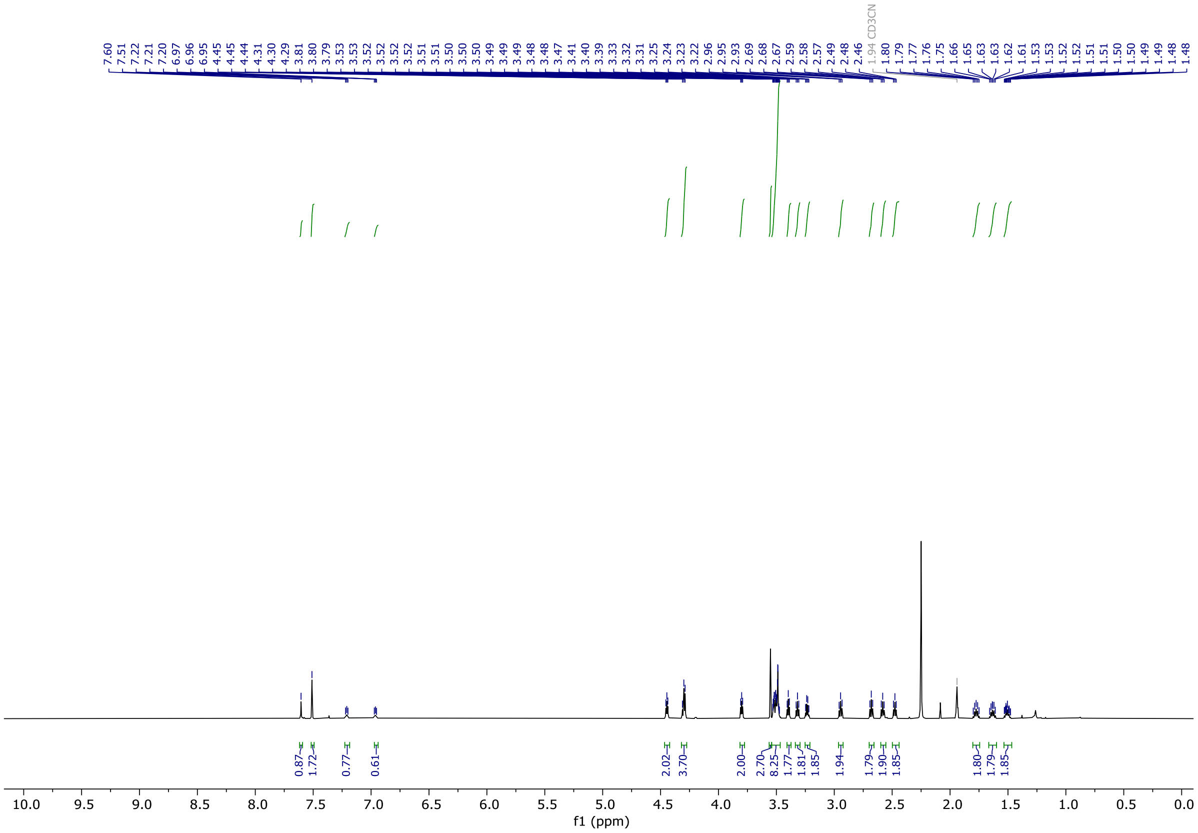

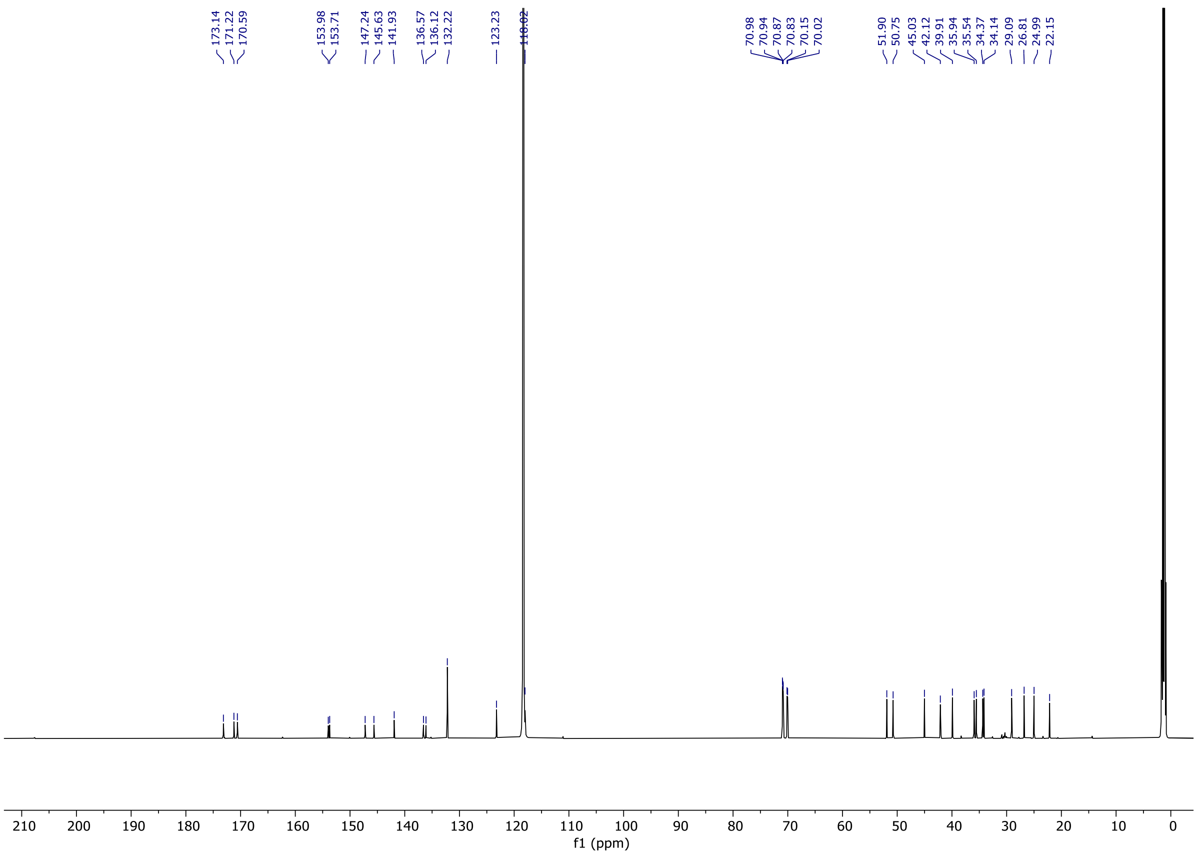

Figure S31: ^{1}H and ^{13}C NMR for reagent **8**.

2.0 Bioconjugation studies

2.1 Ontruzant Fab **1**

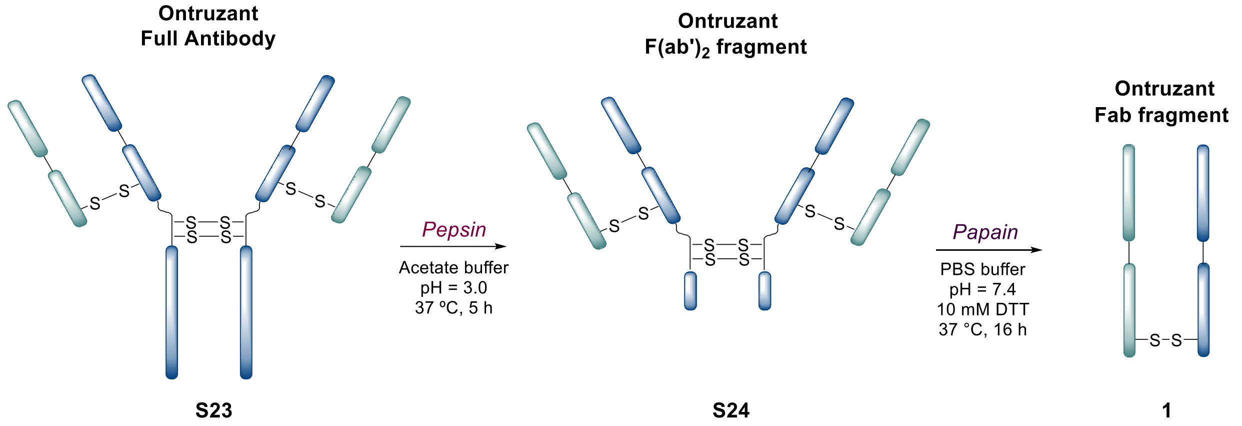

The pH of the sample was lowered by buffer exchange (20 mM sodium acetate, pH 3.1). Immobilized pepsin (470.0 µL) was washed 4 times with the same buffer and Ontruzant (1.0 mL, 68.7 µM) was added. The mixture was incubated for 5 h at 37 °C under constant agitation (1100 rpm). The resin was separated from the digest using a filter column and washed 3 times with digest buffer (50 mM sodium phosphate, 150 mM NaCl, 1 mM EDTA, pH 6.8). The digest was combined with the washes and the volume adjusted to 0.5 mL. Immobilized papain (783 µL, 10 mg/mL) was activated with 10 mM DTT (in digest buffer) with constant agitation (1100 rpm) for 90 min at 37 °C. The resin was washed 4 times with digest buffer (without DTT) and the 0.5 mL of F(ab')$_2$ solution was added. The mixture was incubated for 24 h at 37 °C under constant agitation (1100 rpm). The resin was separated from the digest using a filter column, washed 3 times with PBS (10 mM phosphate, 2.7 mM KCl, 137 mM NaCl, pH 7.4) and the digest combined with the washes. The buffer was exchanged completely for PBS (10 mM phosphate, 2.7 mM KCl, 137 mM NaCl, pH 7.4), and the volume adjusted to 0.5 mL. The purity of Ontruzant Fab **1** (2.3 mg, 75% yield) was confirmed using SDS-PAGE and LCMS. Expected mass: 47639.10 Da. Observed mass: 47638.50 Da.

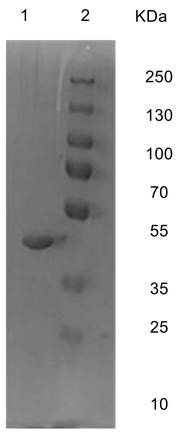

Figure S32: SDS-PAGE gel, 1: Ontruzant Fab **1**, **2:** Ladder.

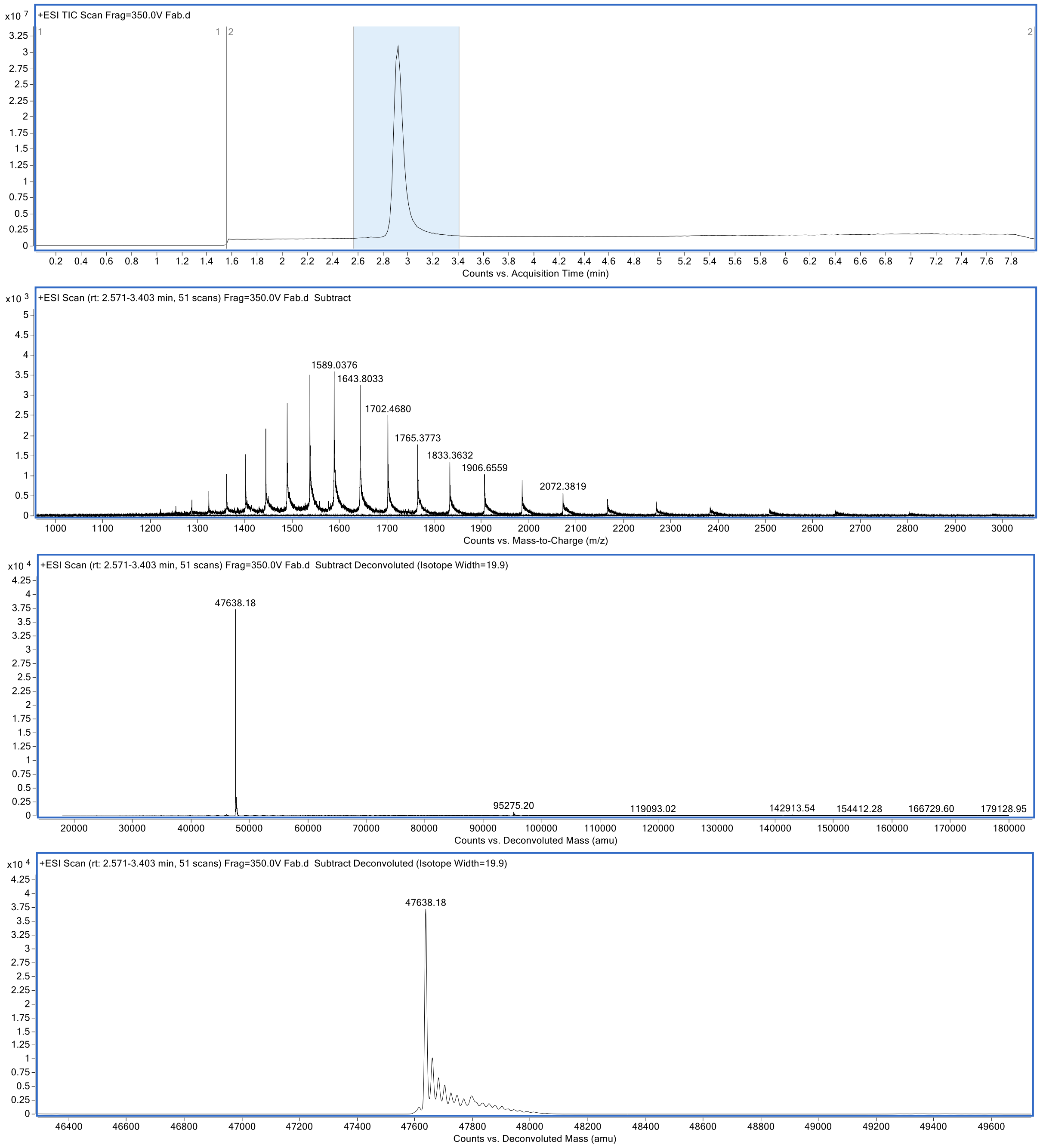

Figure S33: (i) TIC LC-MS trace (top), (ii) non-deconvoluted LC-MS trace (upper middle), (iii) deconvoluted MS data (lower middle, wide range), (iv) bottom (zoom in mass range) for Ontruzant Fab **1**.

2.2 Reduction of Ontruzant Fab 1

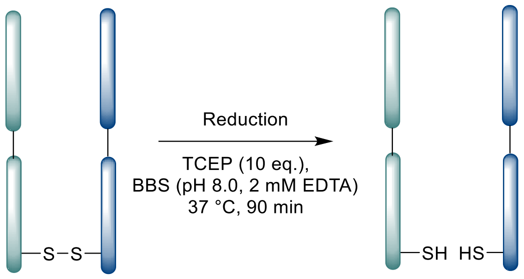

1 **S25**

TCEP·HCl (1.30 µL, 20 mM in deionised water, 15 eq.) was added to a solution of ontruzant Fab **1** (130 µL, 20 µM) in BBS (25 mM sodium borate, 25 mM NaCl, 2 mM EDTA, pH 8.0) and the solution incubated at 37 °C for 90 min. Excess reagents were removed by centrifugation (7 K Zeba Spin desalting column) into BBS (25 mM sodium borate, 25 mM NaCl, 2 mM EDTA, pH 8.0).

2.3 General experimental procedure for using 2-step PD conjugation and lysine transfer protocol

To a solution of Ontruzant Fab **1** (130 µL, 20 µM) in BBS (25 mM sodium borate, 25 mM NaCl, 2 mM EDTA, pH 8.0) was added TCEP·HCl (20 mM in DI H_2O, 10 eq.) The reaction was incubated at 37 °C for 90 min whilst shaking (300 rpm). Upon completion, the conjugate was purified into PBS (pH 7.4, 100 mM, 20 mM NaCl, 2 mM EDTA) by centrifugation (7 K Zeba Spin desalting column), the PD reagent (10 mM in MeCN, 10 eq.) was added and the solution incubated at 37 °C for 4 h whilst shaking at (300 rpm). Upon completion, the conjugate was purified into BBS (25 mM sodium borate, 25 mM NaCl, 2 mM EDTA, pH 8.5). The reaction was incubated at 37 °C for 24 h with shaking (300 rpm). The conjugate was then purified into BBS (25 mM sodium borate, 25 mM NaCl, no EDTA, pH 8.0), DTT (50 mM in DMSO, 175 eq.) was added, and the reaction was incubated at 37 °C for 1 h. After this time, the conjugate was purified into BBS (25 mM sodium borate, 25 mM NaCl, no EDTA, pH 8.0) at 37 °C for 3 h to allow disulfide restoration. Upon completion, the conjugate was purified into DI H_2O. The resulting conjugate was analysed by LCMS analysis.

2.3.1 Reaction of Ontruzant Fab **1** with reagent **2a**

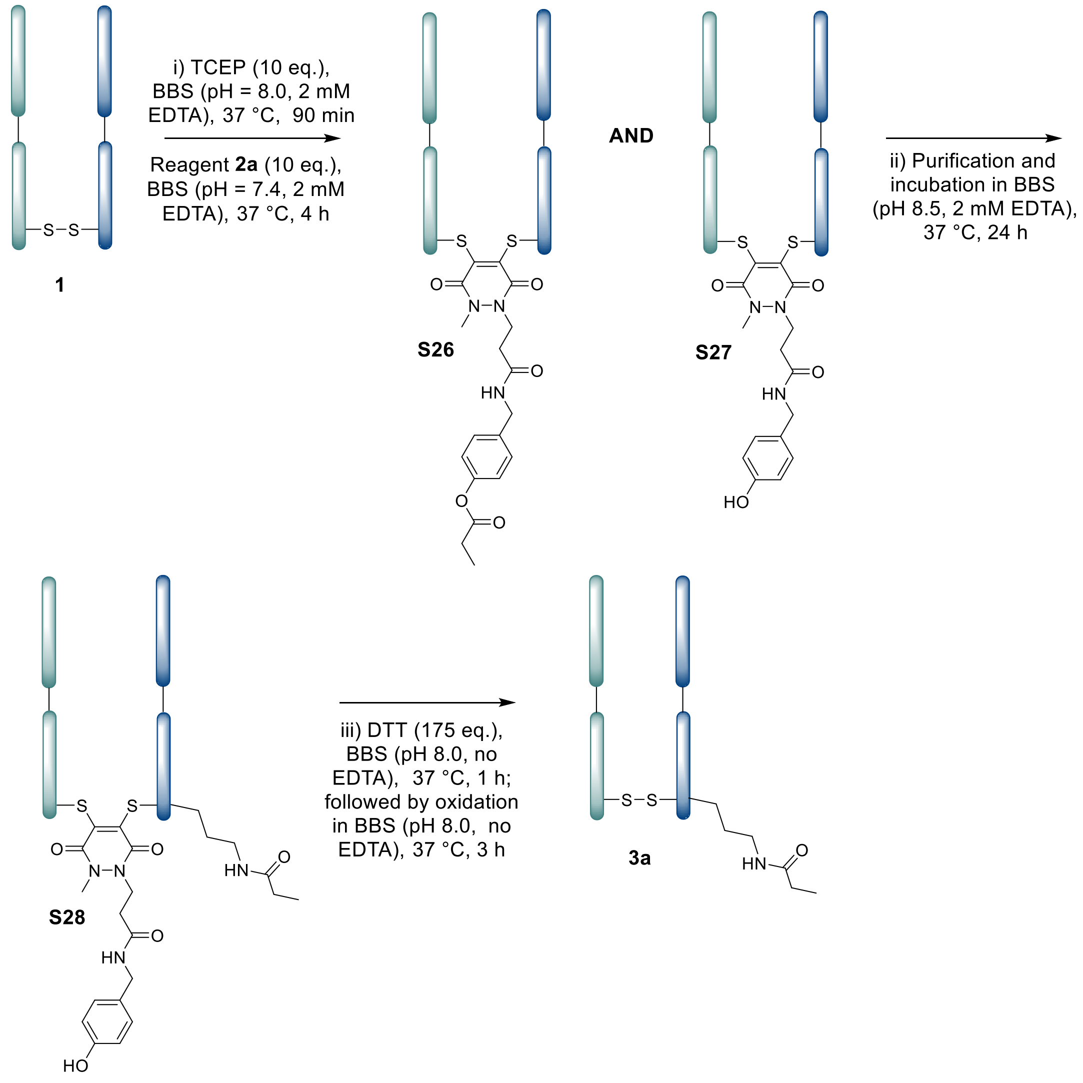

Results were obtained using general procedure 2.3, using reagent **2a**.

a) Conjugation step (i)

Conjugate **S26**: Expected mass: 47996.45 Da, observed mass: 47995.46 Da

Hydrolysed conjugate **S27**: Expected mass: 47940.39 Da, observed mass: 47940.94 Da

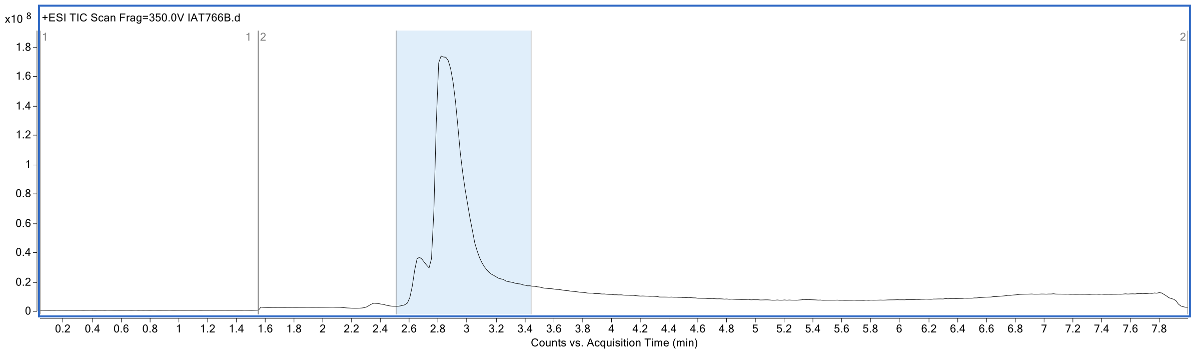

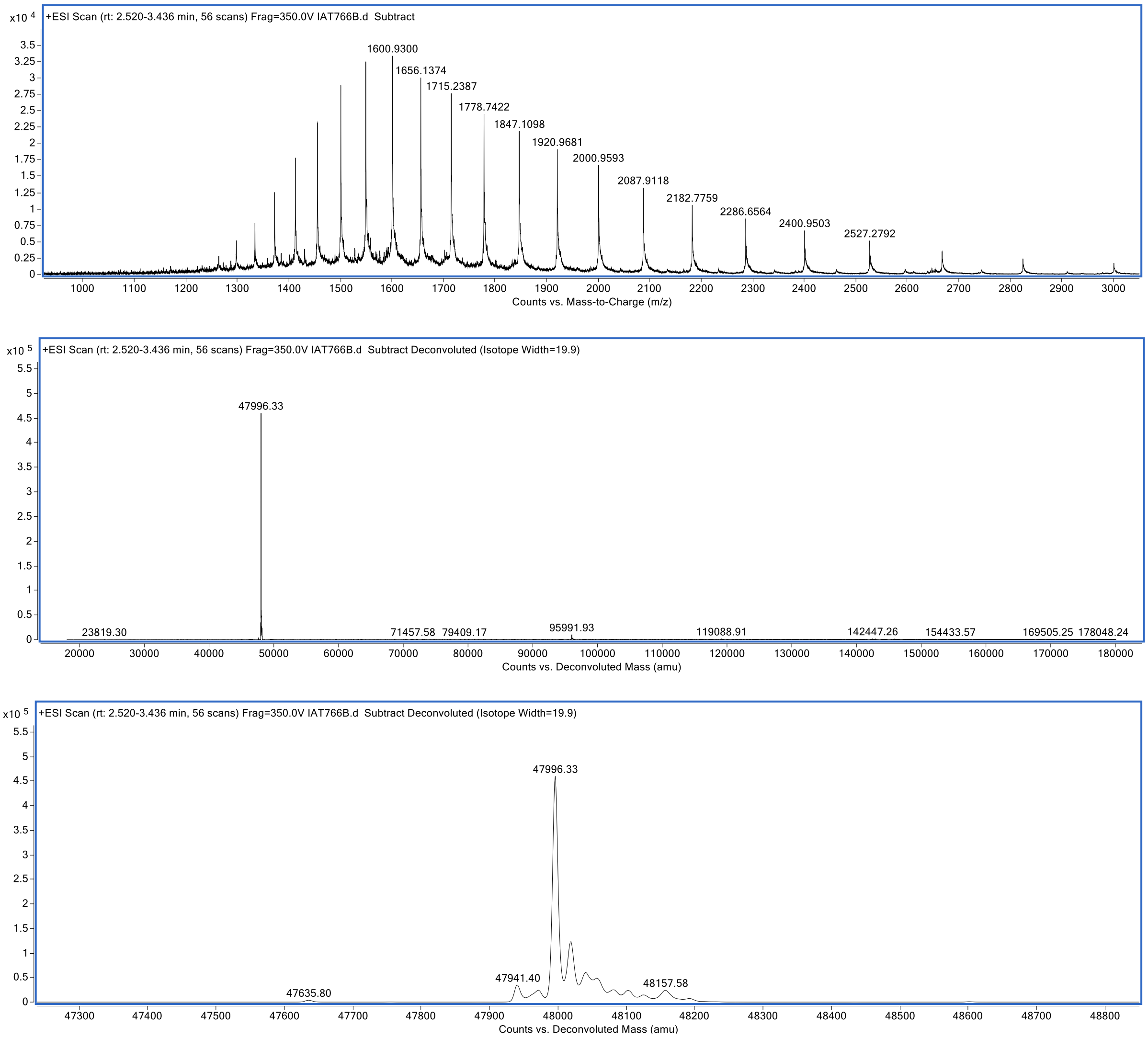

Figure S34: (i) TIC LC-MS trace (top), (ii) non-deconvoluted LC-MS trace (upper middle), (iii) deconvoluted MS data (lower middle, wide range), (iv) zoom in mass range (bottom) for conjugation step.

b) Lysine reaction step (ii)

Conjugate **S28**: Expected mass: 47996.45 Da, observed mass: 47996.35 Da

Hydrolysed conjugate **S27**: Expected mass: 47940.39 Da, observed mass: 47941.05 Da

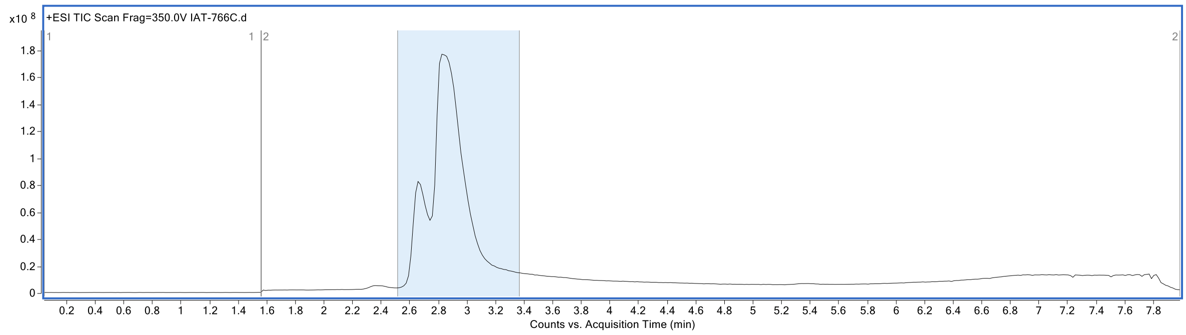

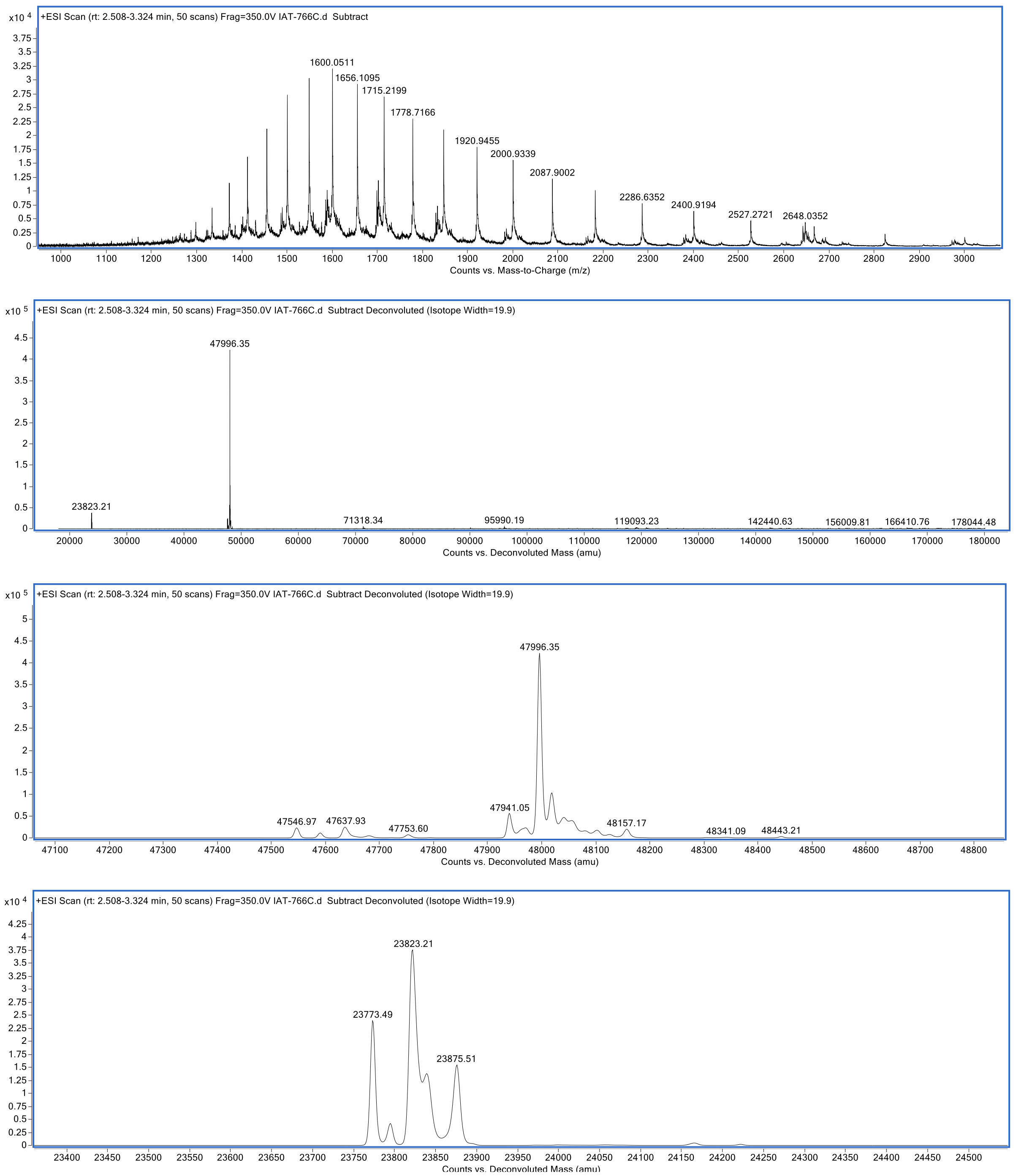

Figure S35: (i) TIC LC-MS trace (top), (ii) non-deconvoluted LC-MS trace (upper middle), (iii) deconvoluted MS data (lower middle, wide range), (iv) zoom in mass range in Fab region (upper bottom), (v) zoom in mass range in LC/HC region (bottom) for lysine reaction step.

c) Restoration step

Mono-labelled conjugate **3a**: Expected mass: 47695.10 Da, observed mass: 47695.62 Da

Ontruzant Fab **1**: Expected mass: 47639.10 Da, observed mass: 47639.26 Da

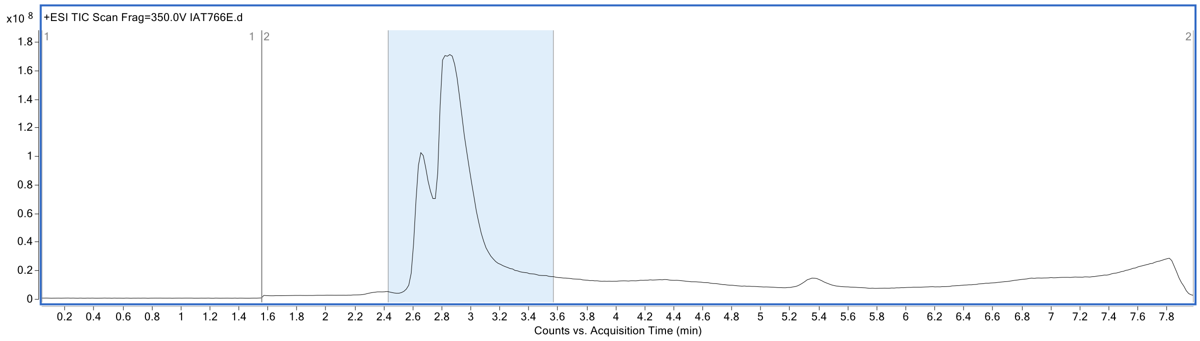

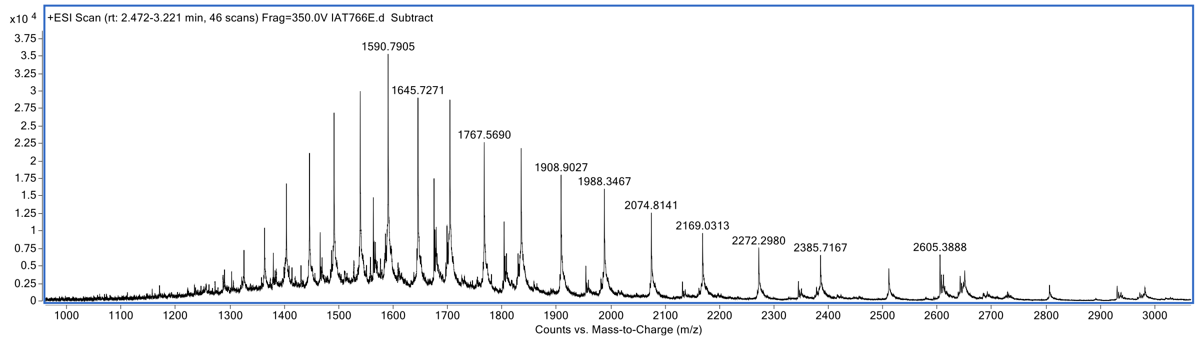

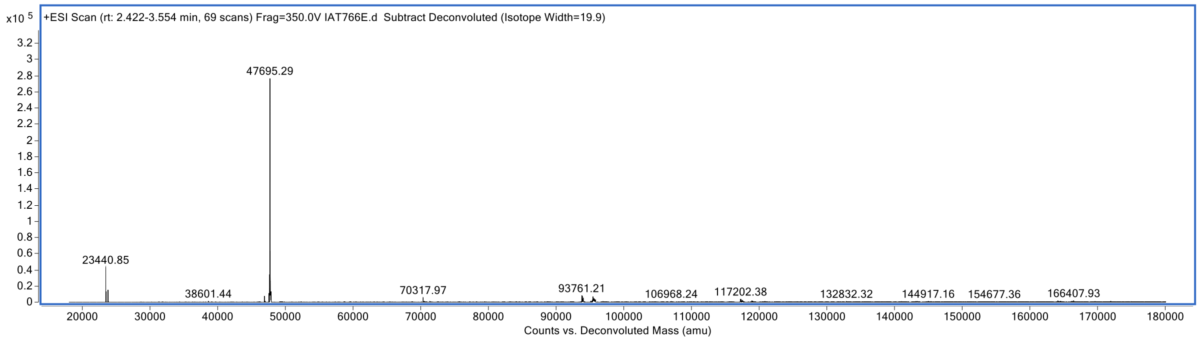

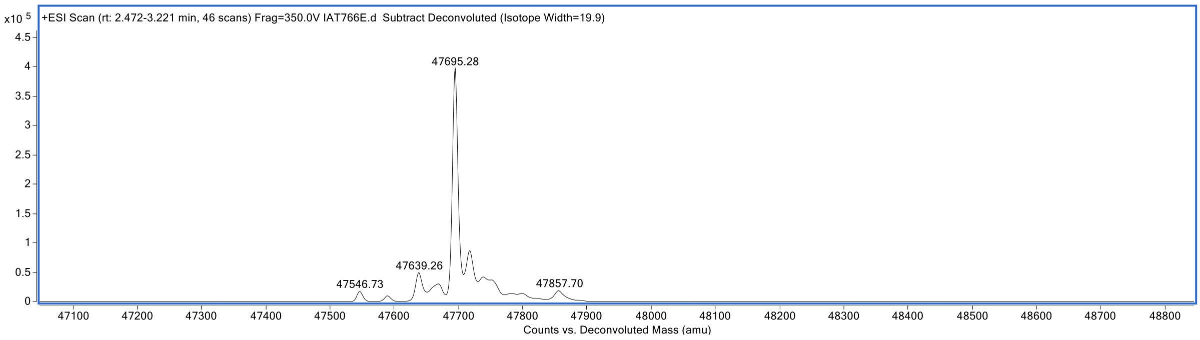

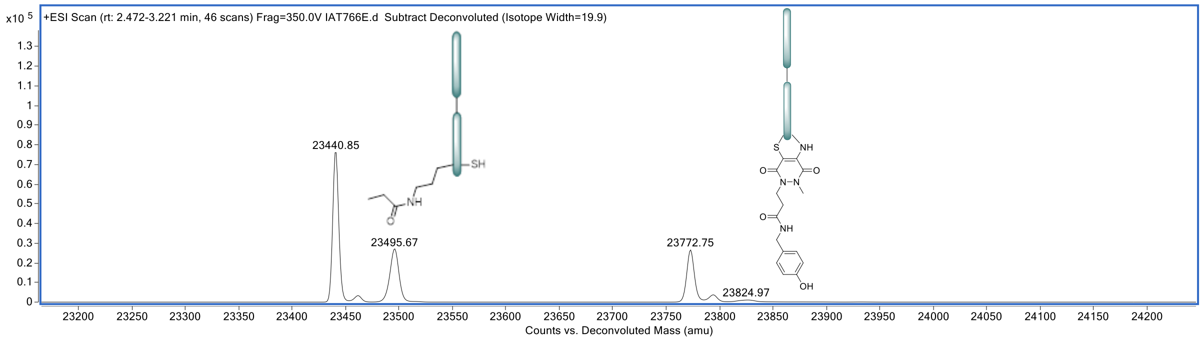

Figure S36: (i) TIC LC-MS trace (top), (ii) non-deconvoluted LC-MS trace (upper middle), (iii) deconvoluted MS data (lower middle, wide range), (iv) zoom in mass range in Fab region (upper bottom), (v) zoom in mass range in LC/HC region (bottom) for restoration step.

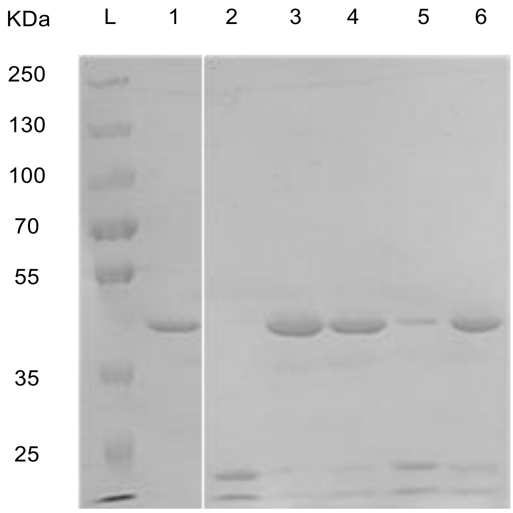

Figure S37: SDS-PAGE gel: L) Ladder, 1) Ontruzant Fab **1**, 2) Reduction step, 3) Conjugation step, 4) Lysine reaction step, 5) Deprotection step, 6) Disulfide restoration step.

2.3.2 Reaction of Ontruzant Fab **1** with reagent **2b**

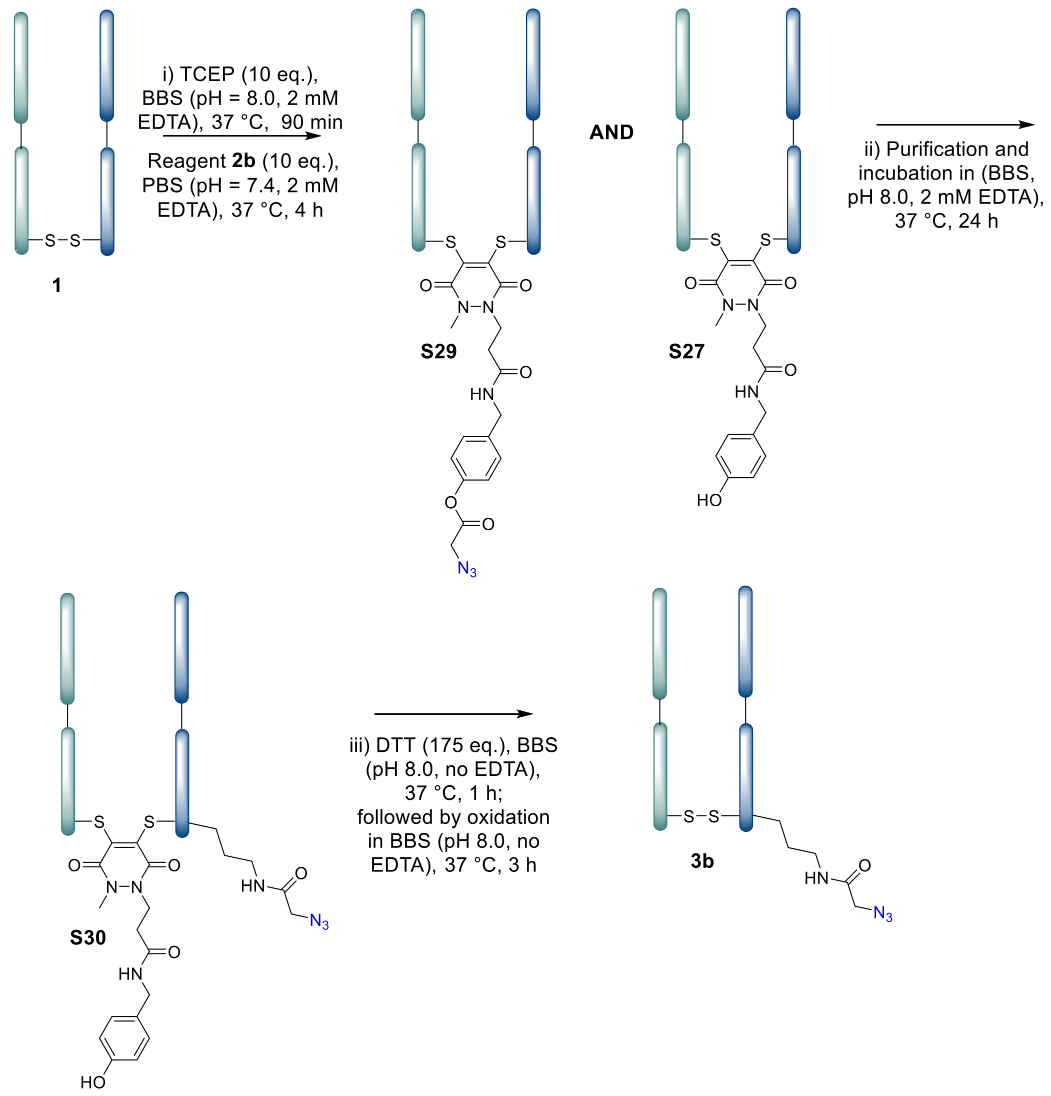

Results were obtained using general procedure 2.3, using reagent **2b**.

a) Conjugation step (i)

Conjugate **S29**: Expected mass: 48022.44, observed mass: 48022.30 Da

Hydrolysed conjugate **S27**: Expected mass: 47939.39 Da, observed mass: 47939.41 Da

Conjugate **S29** + 3 lysine modifications: Expected mass: 48104.44 Da, observed mass: 48104.70 Da

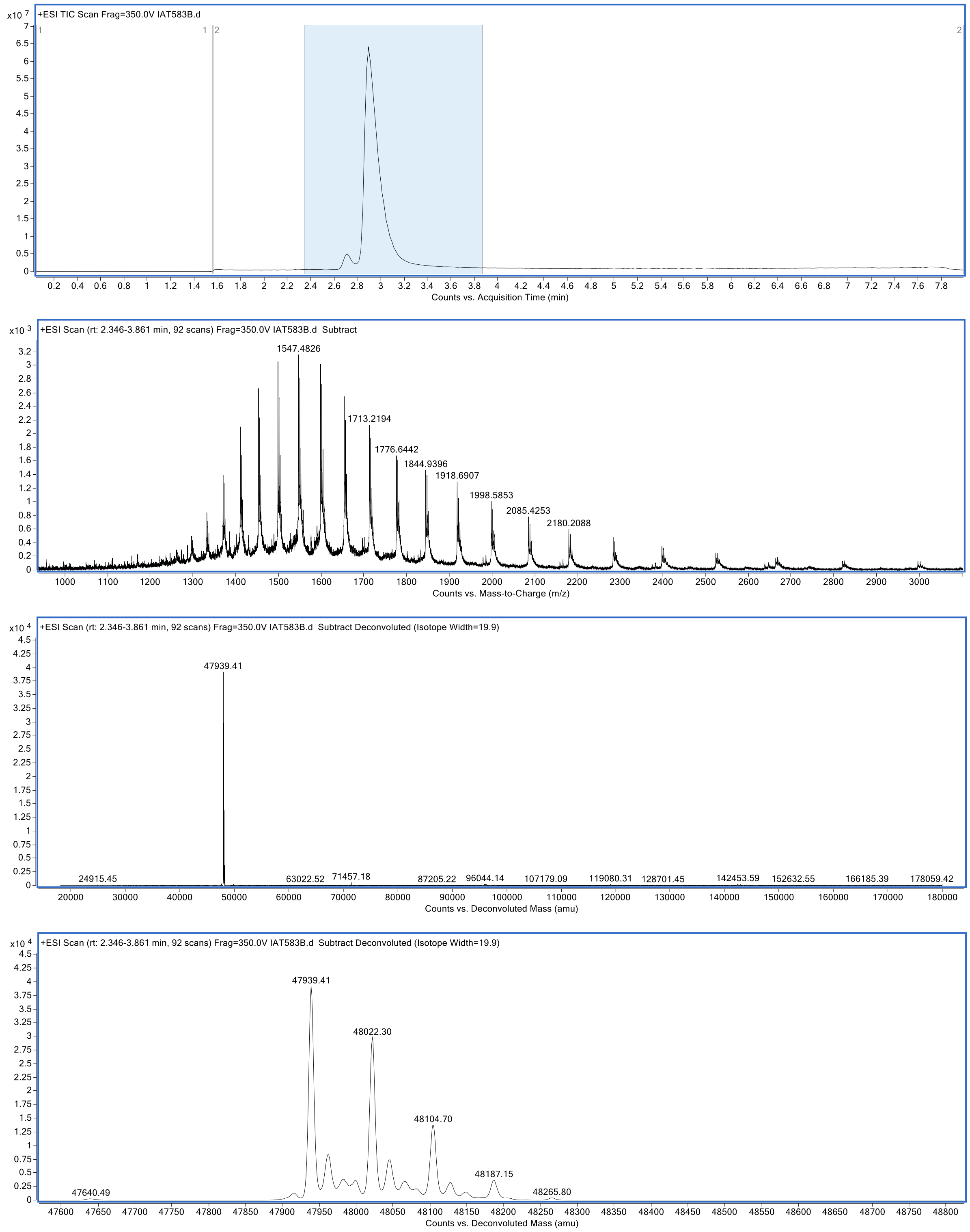

Figure S38: (i) TIC LC-MS trace (top), (ii) non-deconvoluted LC-MS trace (upper middle), (iii) deconvoluted MS data (lower middle, wide range), (iv) zoom in mass range (bottom) for conjugation step.

b) Lysine reaction step (ii)

Conjugate **S30**: Expected mass: 48023.44 Da, observed mass: 48022.30 Da

Hydrolysed conjugate **S27**: Expected mass: 47940.39 Da, observed mass: 47939.50 Da

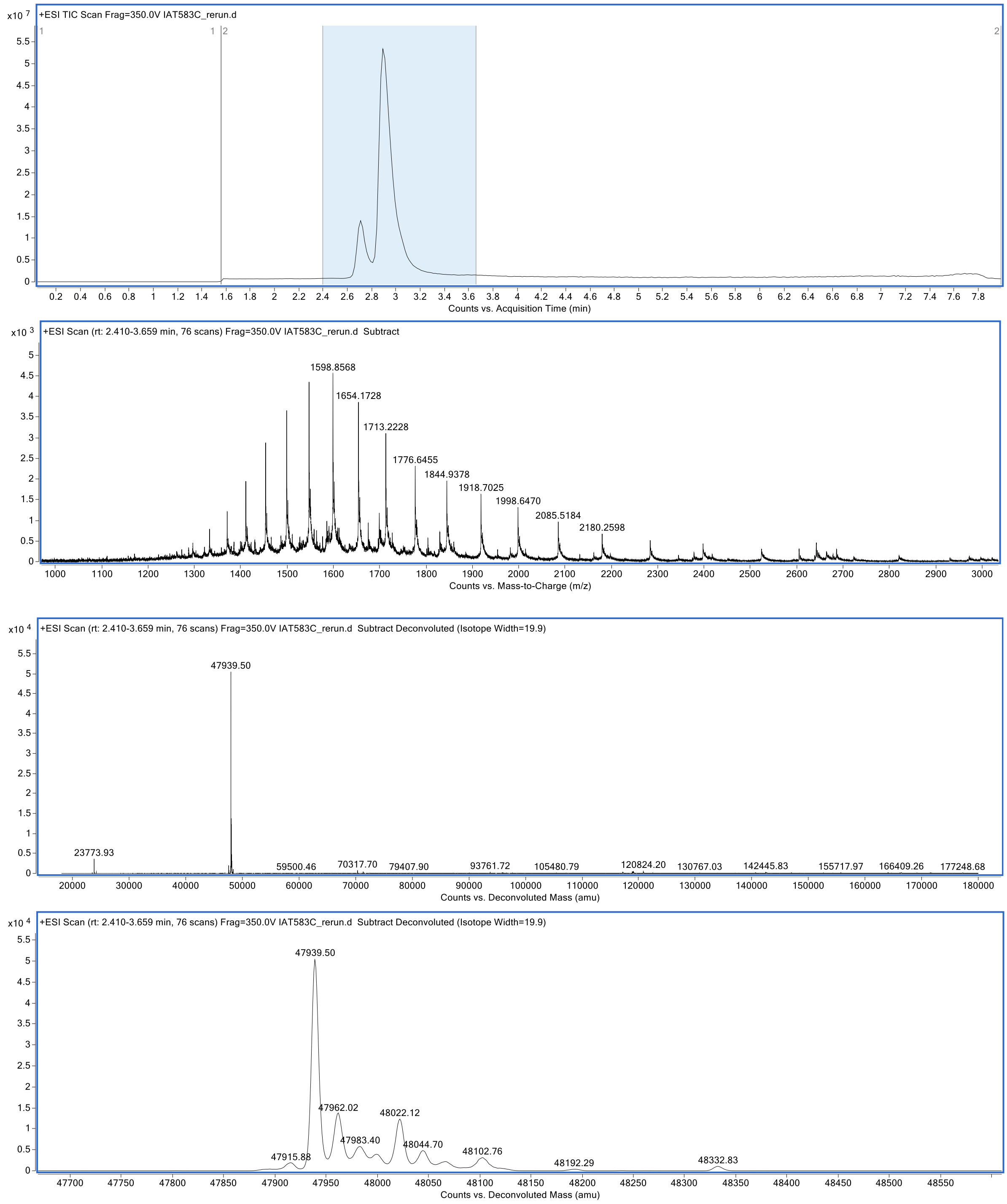

Figure S39: (i) TIC LC-MS trace (top), (ii) non-deconvoluted LC-MS trace (upper middle), (iii) deconvoluted MS data (lower middle, wide range), (iv) zoom in mass range (bottom) for lysine reaction step.

c) Restoration step

Mono-labelled conjugate **3b**: Expected mass: 47724.16, observed mass: 47721.55 Da

Ontruzant Fab **1**: Expected mass: 47639.10 Da, observed mass: 47638.18 Da

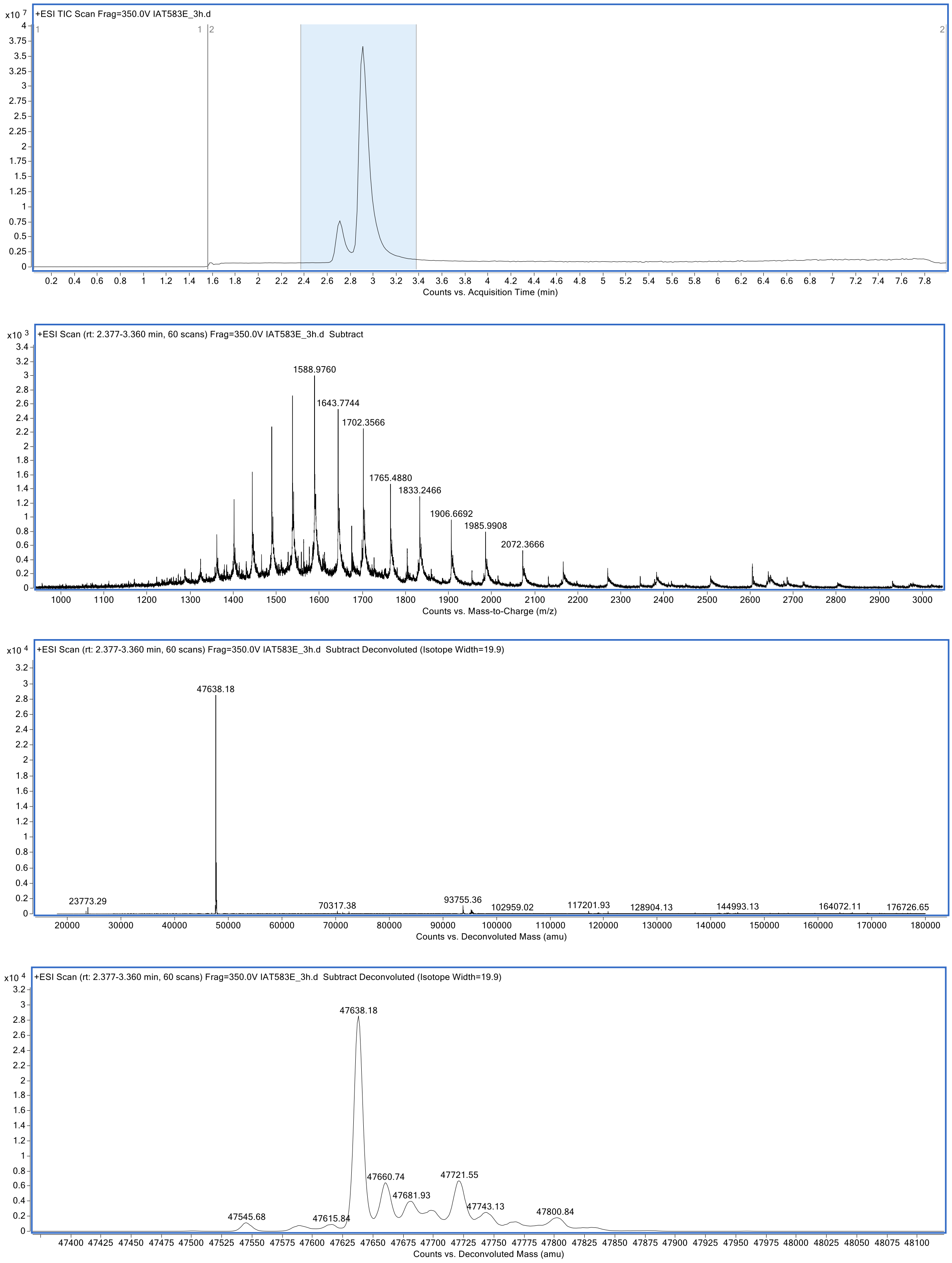

Figure S40: (i) TIC LC-MS trace (top), (ii) non-deconvoluted LC-MS trace (upper middle), (iii) deconvoluted MS data (lower middle, wide range), (iv) zoom in mass range (bottom) for restoration step.

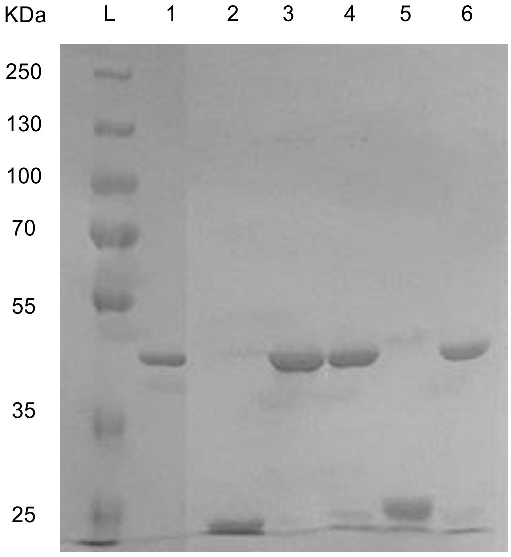

Figure S41: SDS-PAGE gel: L) Ladder, 1) Ontruzant Fab **1**, 2) Reduction step, 3) Conjugation step, 4) Lysine reaction step, 5) Deprotection step, 6) Disulfide restoration step.

2.3.3 Reaction of Ontruzant Fab **1** with reagent **2c**

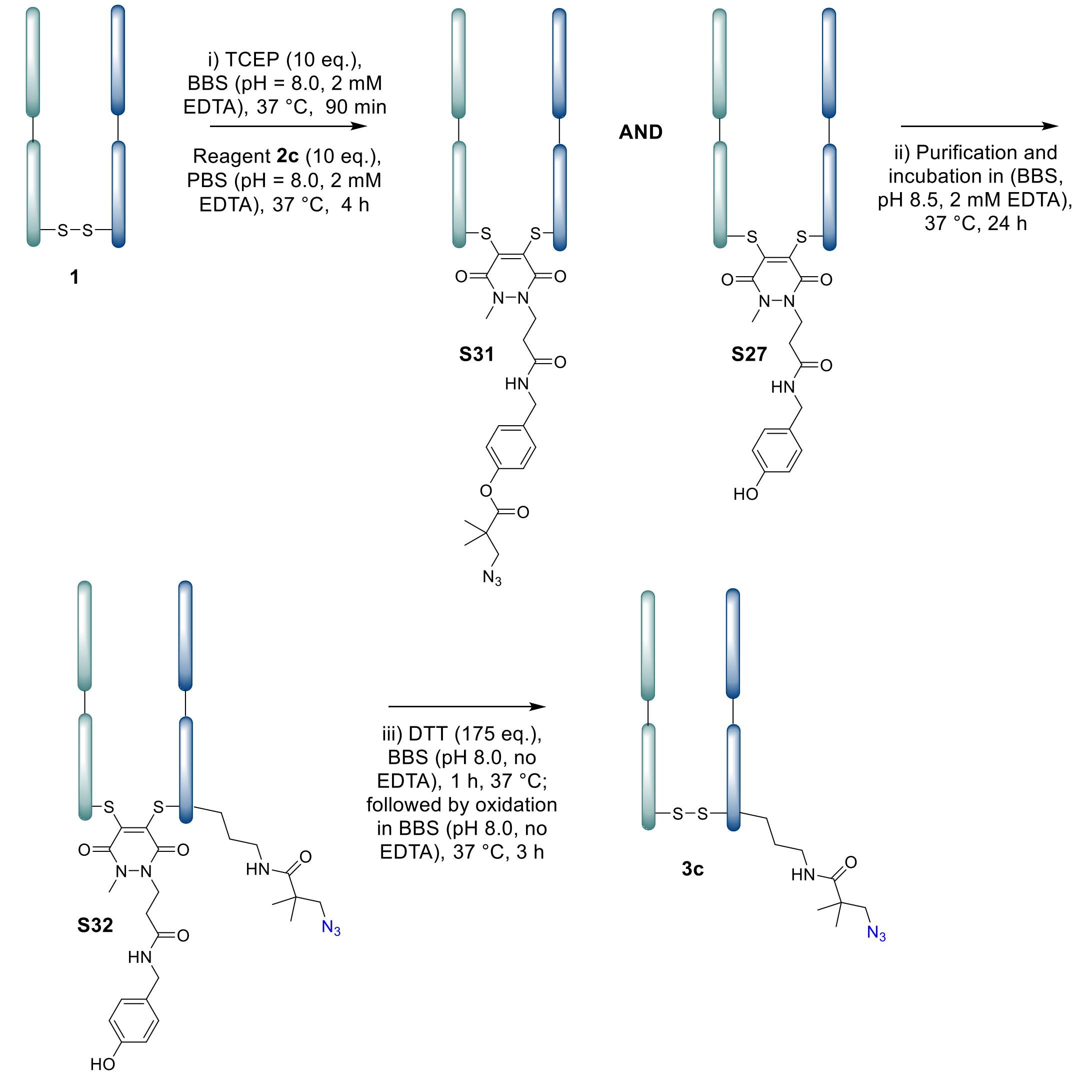

Results were obtained using general procedure 2.3, using reagent **2c**.

a) Conjugation step (i)

Conjugate **S31**: Expected mass: 48065.52 Da, observed mass: 48064.80 Da

Hydrolysed conjugate **S27**: 47939.39 Da, observed mass: 47939.35 Da

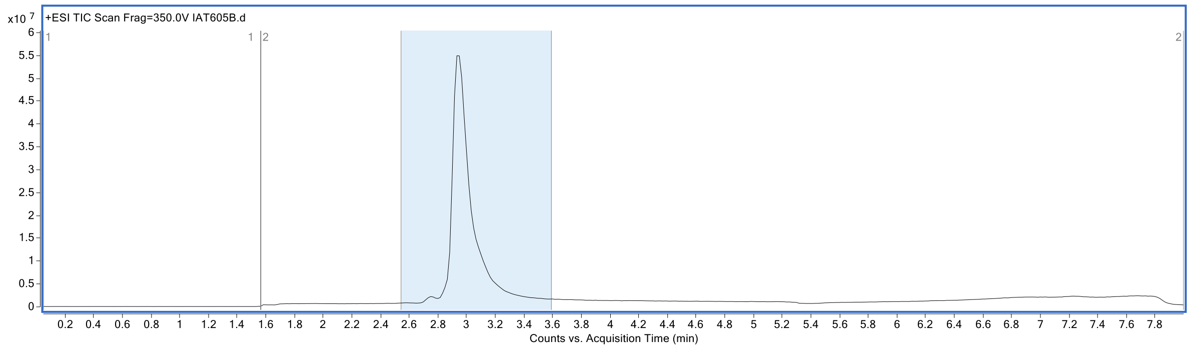

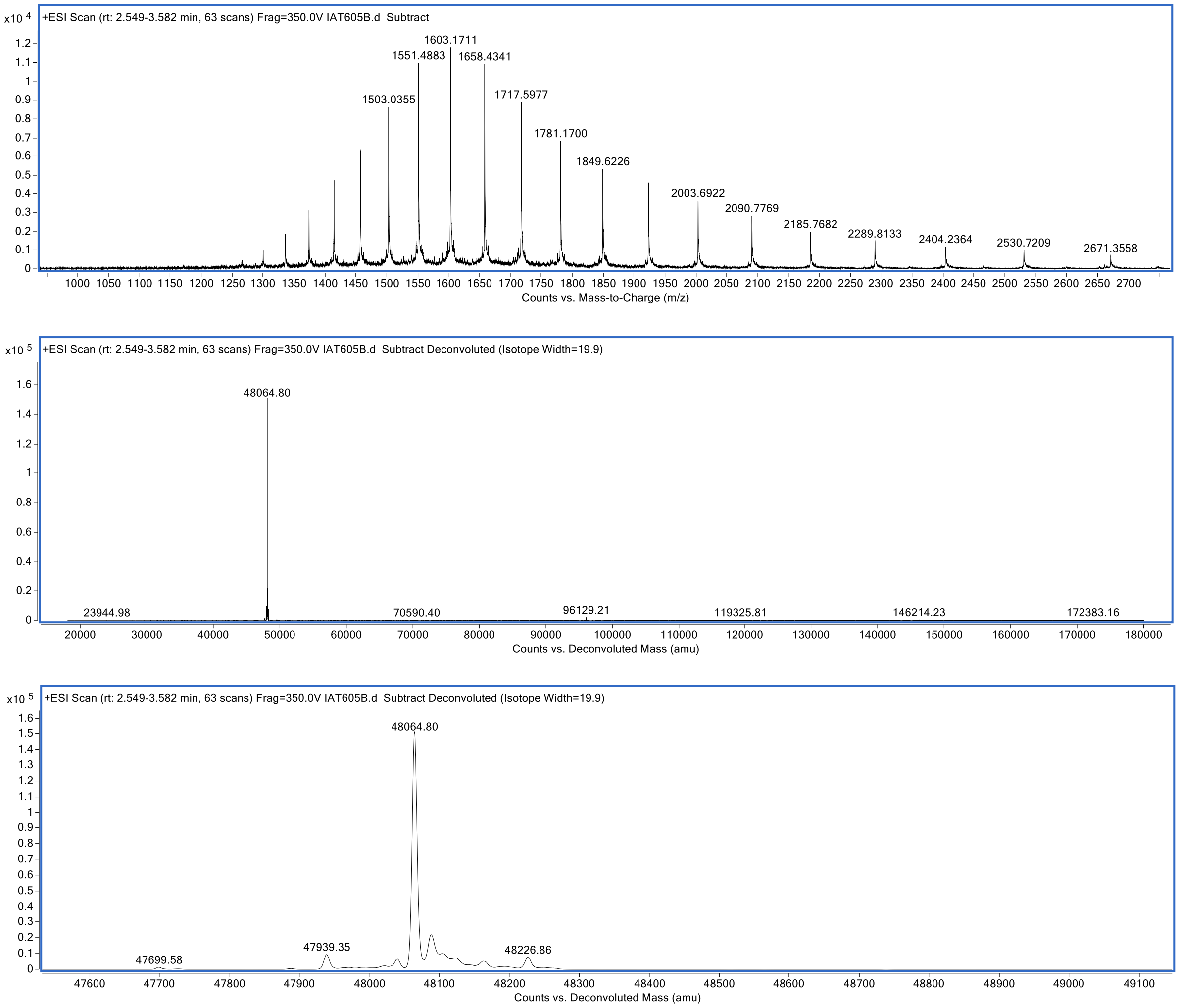

Figure S42: (i) TIC LC-MS trace (top), (ii) non-deconvoluted LC-MS trace (upper middle), (iii) deconvoluted MS data (lower middle, wide range), (iv) zoom in mass range (bottom) for conjugation step.

b) Lysine reaction step (ii)

Conjugate **S32**: Expected mass: 48064.52 Da, observed mass: 48064.80 Da

Hydrolysed conjugate **S27**: Expected mass: 47940.39 Da, observed mass: 47939.42 Da

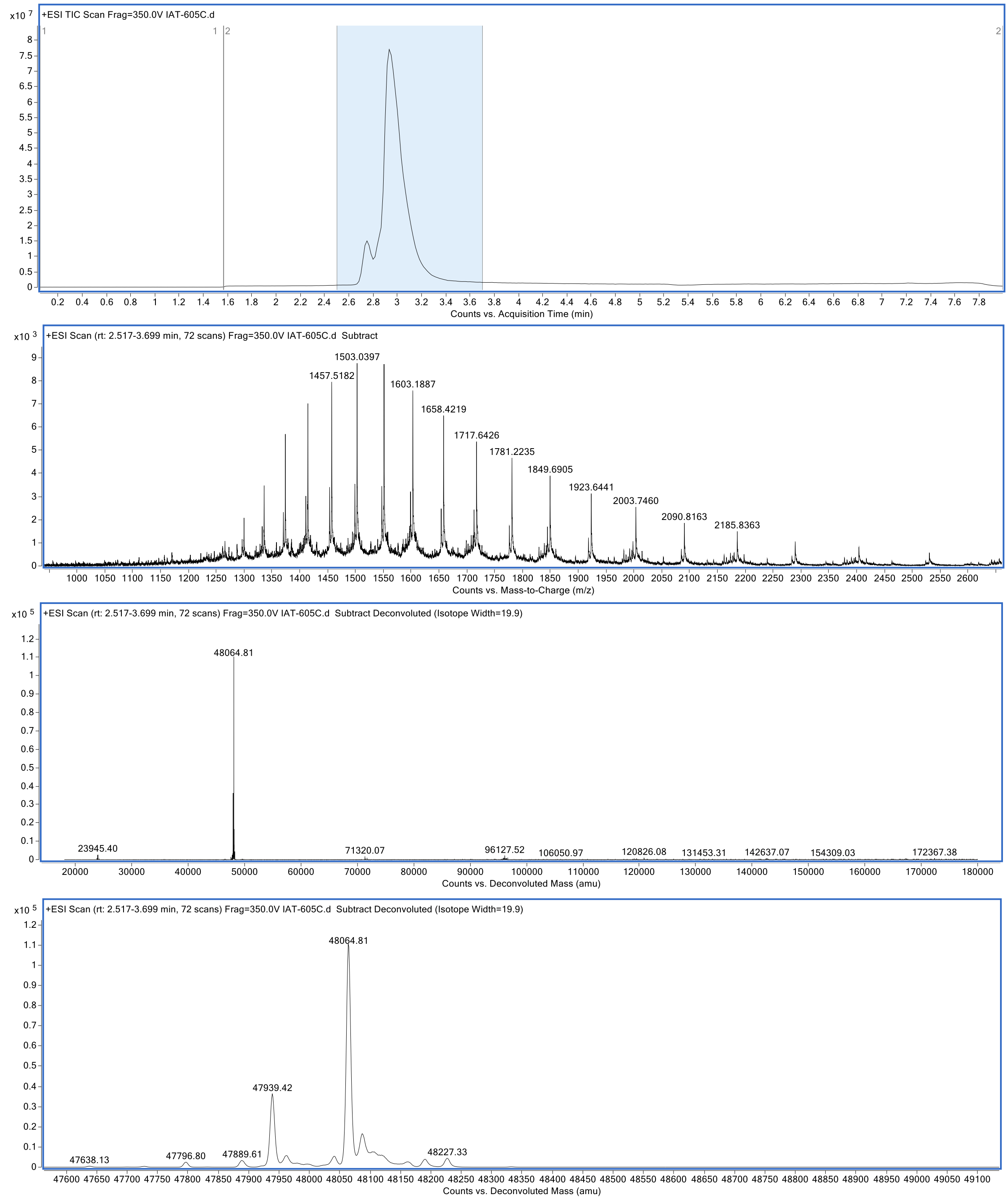

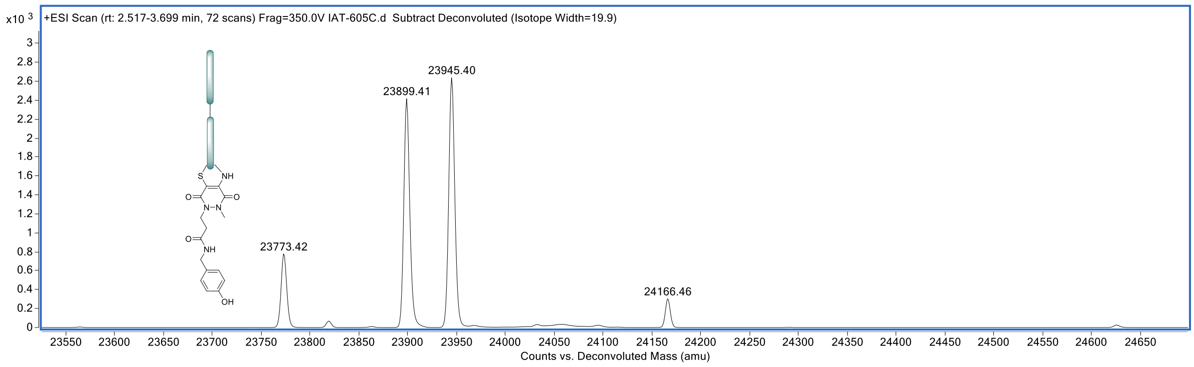

Figure S43: i) TIC LC-MS trace (top), (ii) non-deconvoluted LC-MS trace (upper middle), (iii) deconvoluted MS data (lower middle, wide range), (iv) zoom in mass range in Fab region (upper bottom), (v) zoom in mass range in LC/HC region (bottom) for lysine reaction step.

c) Restoration step

Conjugate **3d:** Expected mass: 47942.55 Da, observed mass: 47940.24 Da

Ontruzant Fab **1**: Expected mass: 47639.10 Da, observed mass: 47638.26 Da

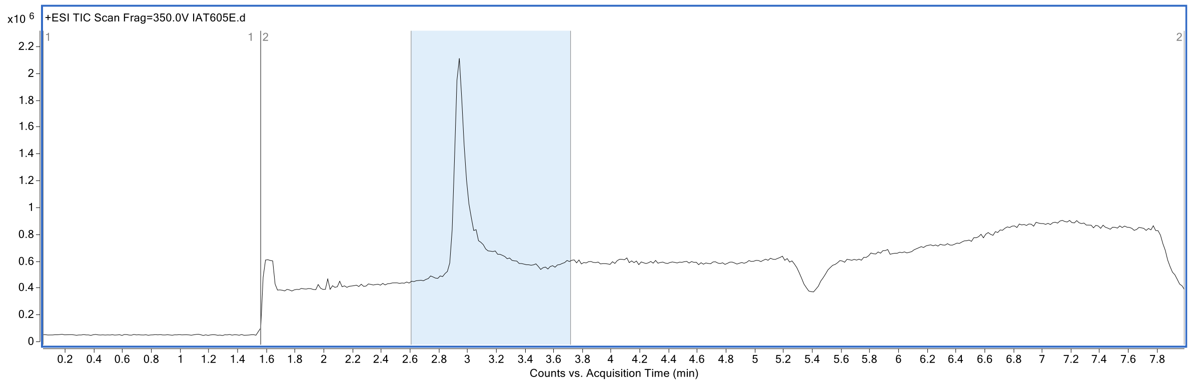

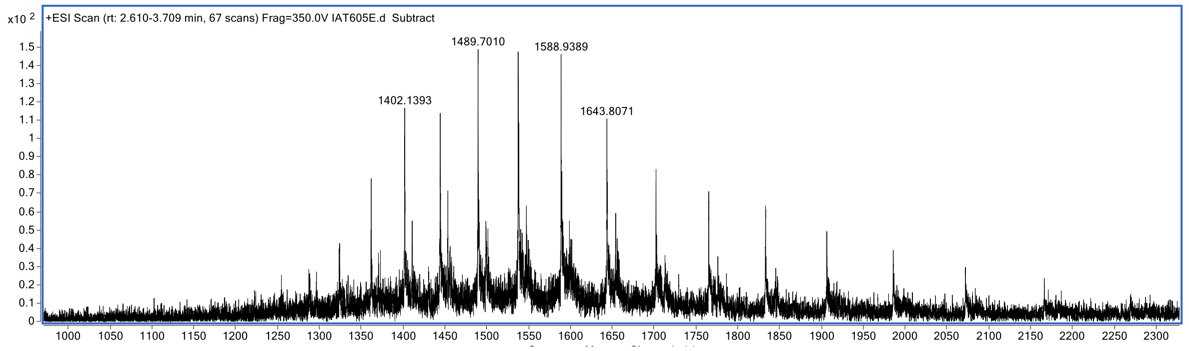

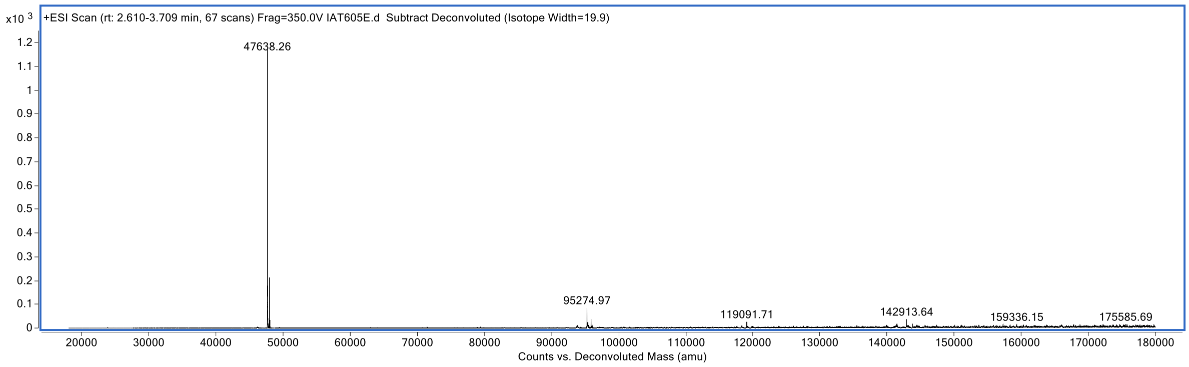

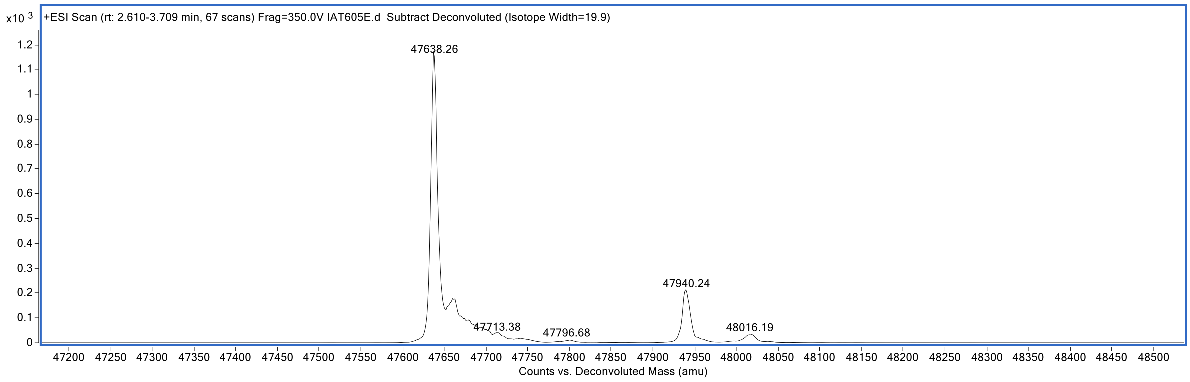

Figure S44: (i) TIC LC-MS trace (top), (ii) non-deconvoluted LC-MS trace (upper middle), (iii) deconvoluted MS data (lower middle, wide range), (iv) zoom in mass range (bottom) for restoration step. Masses in the ca. 95,000 Da region are spurious mass spectrometry artefacts that are the double masses of the conjugates observed in the Fab region; these higher mass species are not observed by SDS-PAGE.

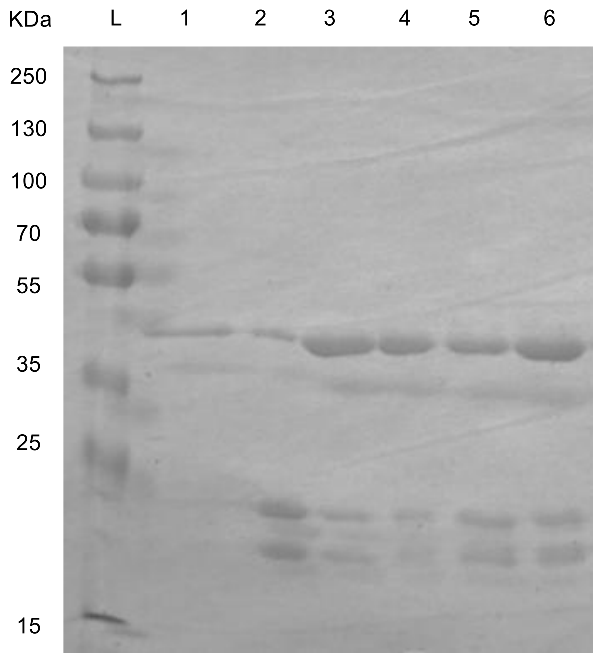

Figure S45: SDS-PAGE gel: L) Ladder, 1) Ontruzant Fab **1**, 2) Reduction step, 3) Conjugation step, 4) Lysine reaction step, 5) Deprotection step, 6) Disulfide restoration step.

2.3.4 Reaction of Ontruzant Fab **1** with reagent **2d**

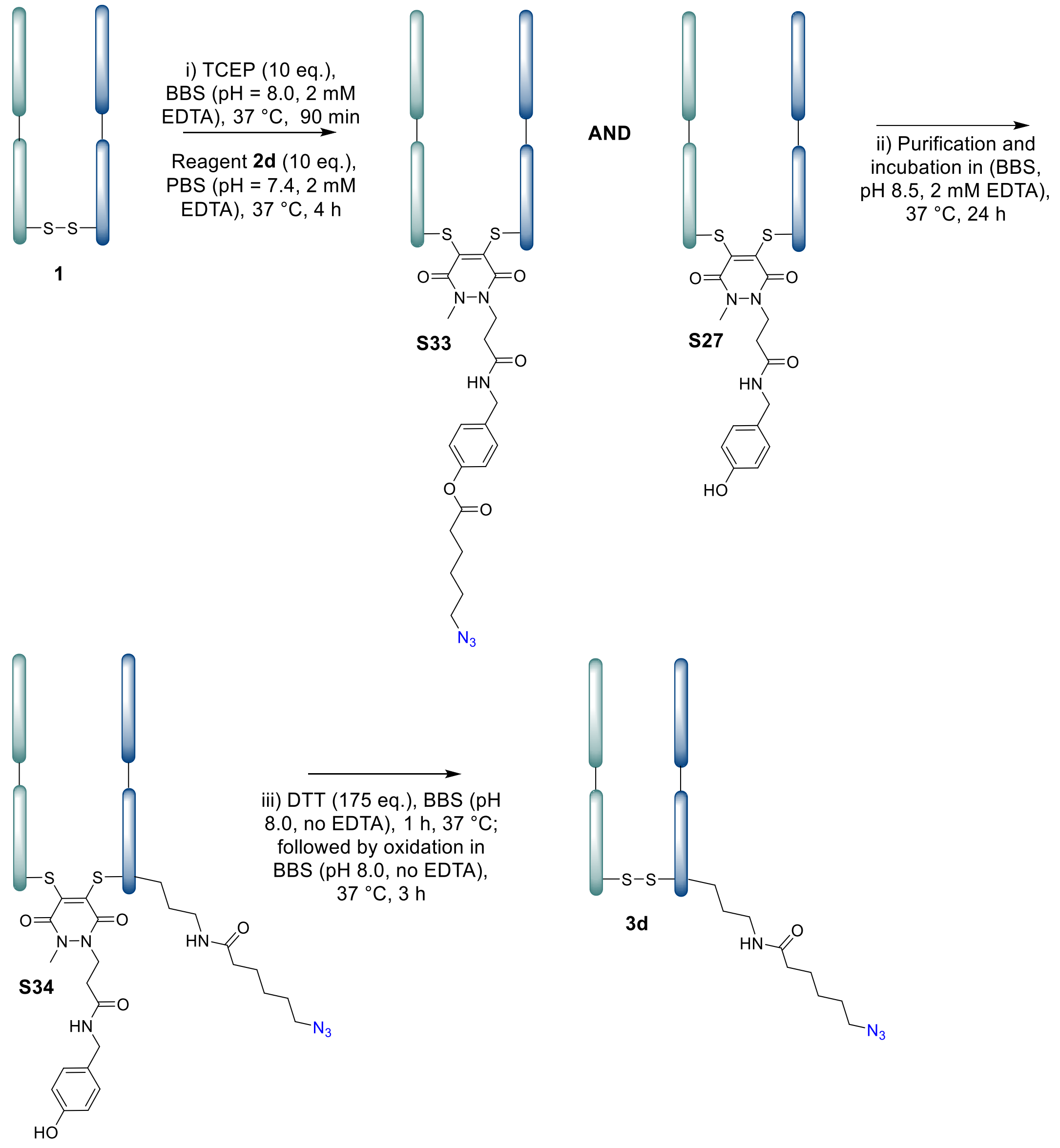

Results were obtained using general procedure 2.3, using reagent **2d**.

a) Conjugation step (i)

Conjugate **S33**: Expected mass: 48078.55 Da, observed mass: 48079.33 Da

Hydrolysed conjugate **S27**: Expected mass: 47939.39 Da, observed mass: 47940.85 Da

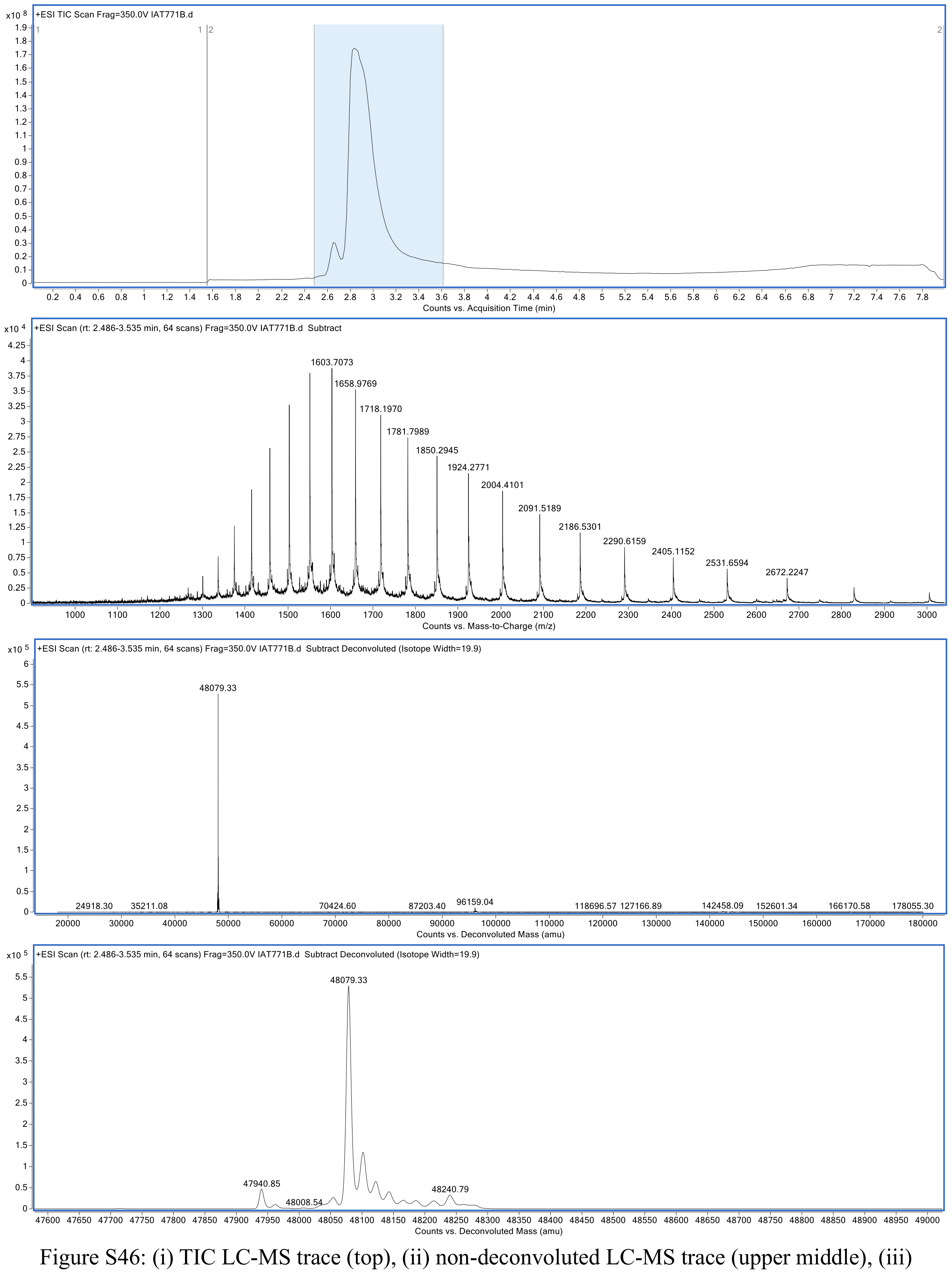

Figure S46: (i) TIC LC-MS trace (top), (ii) non-deconvoluted LC-MS trace (upper middle), (iii) deconvoluted MS data (lower middle, wide range), (iv) zoom in mass range (bottom) for conjugation step.

b) Lysine reaction step (ii)

Conjugate **S33**: Expected mass: 48078.55 Da, observed mass: 48079.30 Da

Hydrolysed conjugate **S27**: Expected mass: 47939.39 Da, observed mass: 47940.74 Da

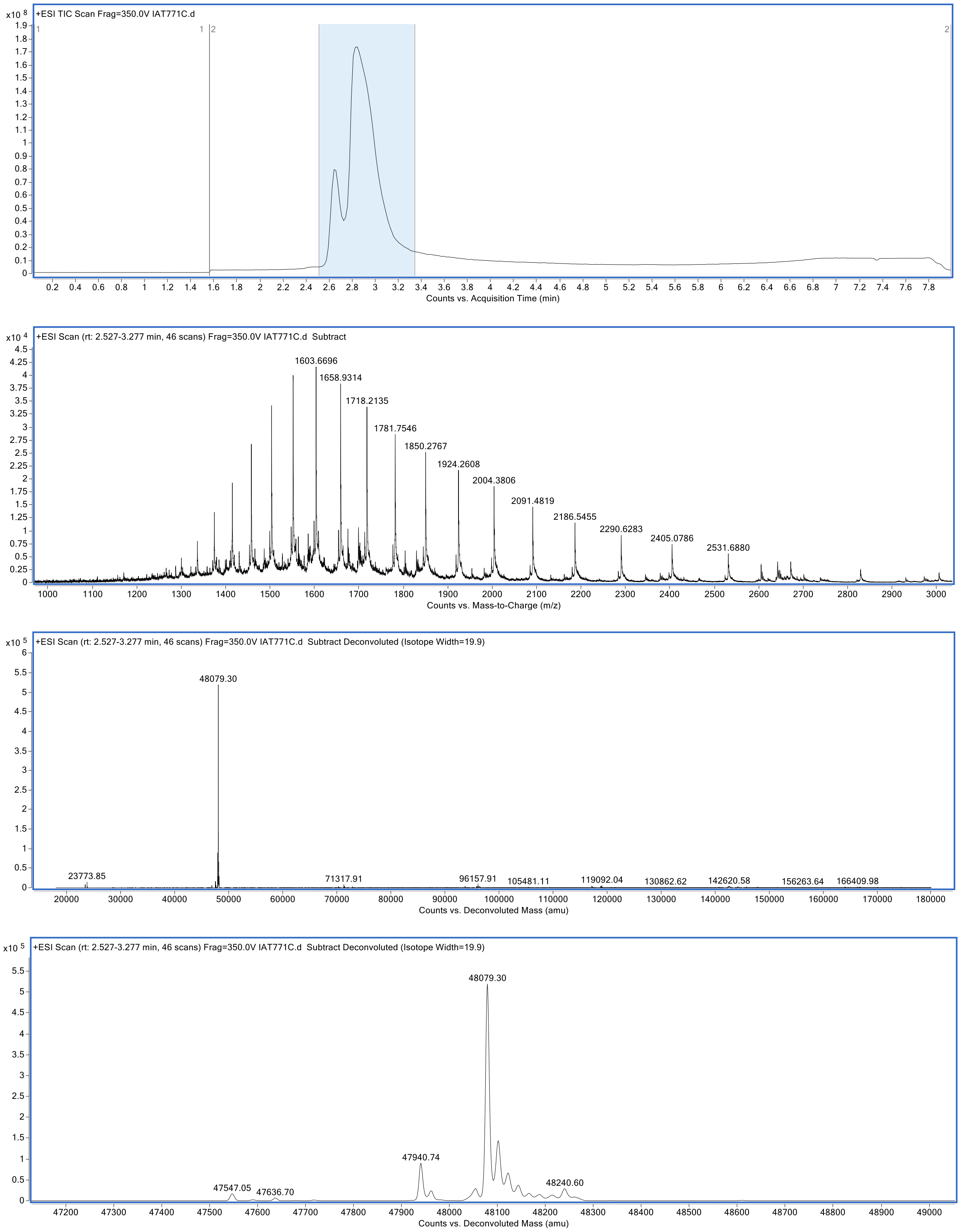

Figure S47: i) TIC LC-MS trace (top), (ii) non-deconvoluted LC-MS trace (upper middle), (iii) deconvoluted MS data (lower middle, wide range), (iv) zoom in mass range in Fab region (bottom) for lysine reaction step.

c) Restoration step

Conjugate **3d:** Expected mass: 47942.55 Da, observed mass: 47940.24 Da

Ontruzant Fab **1**: Expected mass: 47639.10 Da, observed mass: 47638.26 Da

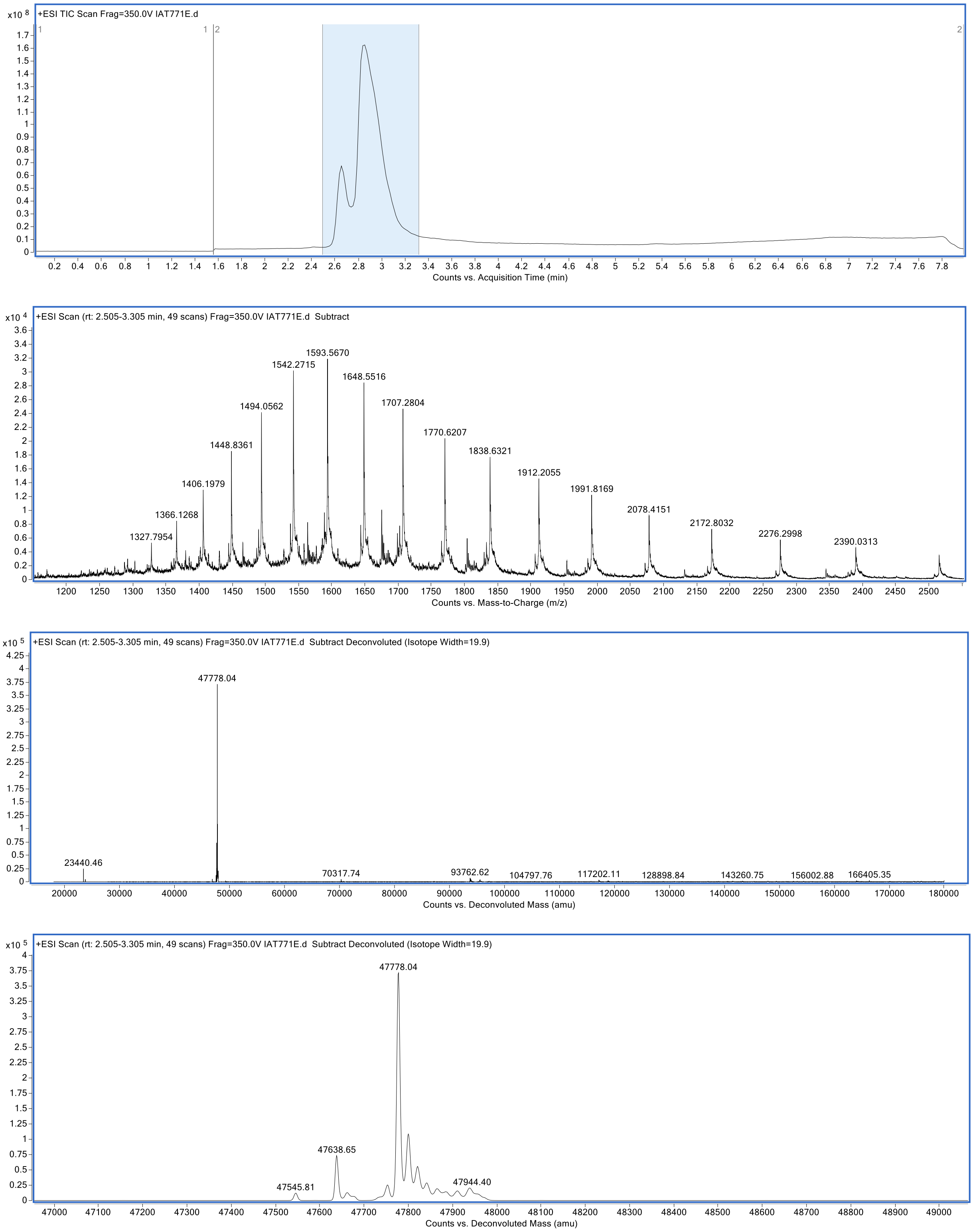

Figure S48: (i) TIC LC-MS trace (top), (ii) non-deconvoluted LC-MS trace (upper middle), (iii) deconvoluted MS data (lower middle, wide range), (iv) zoom in mass range (bottom) for restoration step.

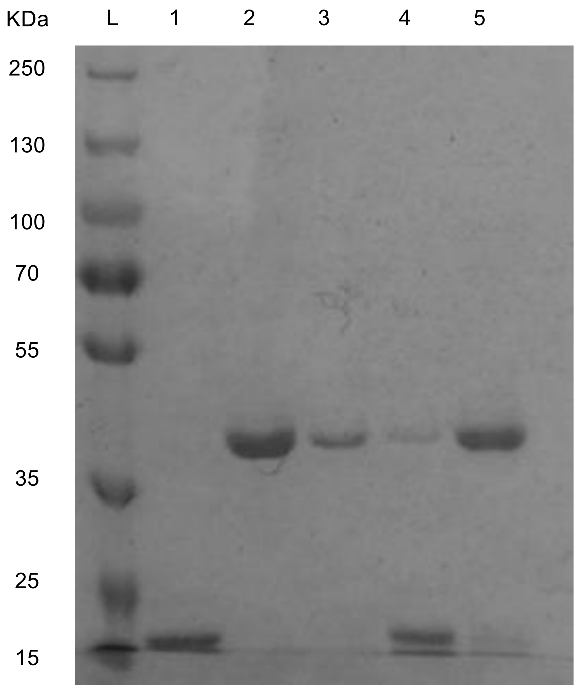

Figure S49: SDS-PAGE gel: L) Ladder, 1) Ontruzant Fab **1**, 2) Reduction step, 3) Conjugation step, 4) Lysine reaction step, 5) Deprotection step, 6) Disulfide restoration step.

2.4 Reaction of Ontruzant Fab **1** with reagent **2d** (10 eq.). Timepoint study for conjugation step (PBS pH 7.4, 37 °C, 4-16 h)

Upon reduction of native Fab, reagent **2d** (10 eq.) was added and the reaction mixture incubated in PBS (pH 7.4, 2 mM EDTA) for 4–16 h. In order to determine the % abundance of the mono-labelled lysine-modified final conjugate, the PD removal step and native disulfide bond restoration upon purification of the final solution in HPLC-grade water were carried out at each time point to evaluate the extent of lysine transfer as a function of time at pH 7.4.

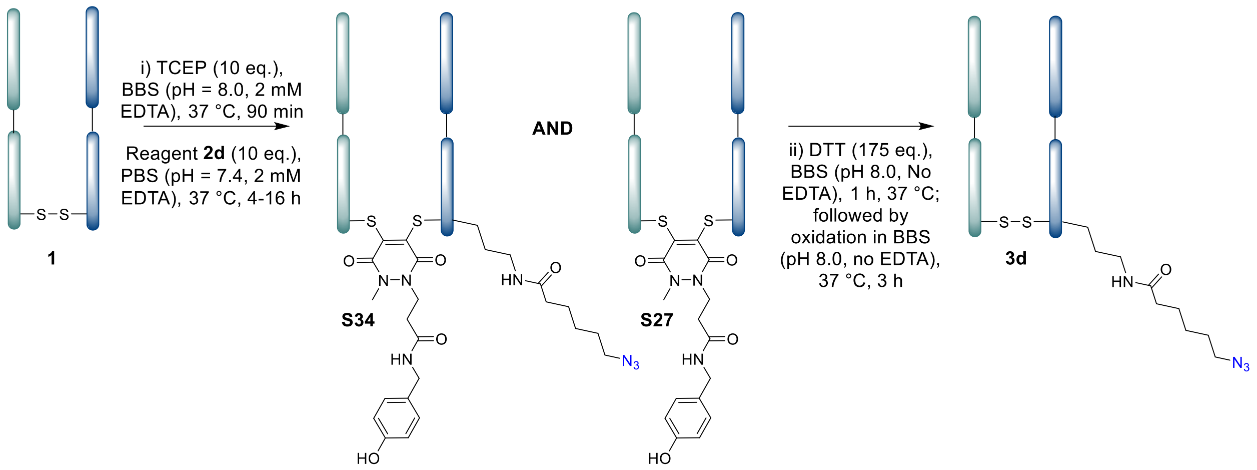

2.4.1 General experimental procedure

To a solution of Ontruzant Fab **1** (130 µL, 20 µM) in BBS (25 mM sodium borate, 25 mM NaCl, 2 mM EDTA, pH 8.0) was added TCEP·HCl (1.30 µL,20 mM in DI H_2O, 10 eq.) The reaction was incubated at 37 °C for 90 min whilst shaking (300 rpm). Upon completion, the conjugate was purified into PBS (50 mM phosphate, 150 mM NaCl, 2 mM EDTA, pH 7.4) by centrifugation (7 K Zeba Spin desalting column), reagent **2d** (10 mM in MeCN, 10 eq.) was added and incubated at 37 °C for 4/6/8/16 h whilst shaking at (300 rpm). Upon completion, the conjugate was purified into BBS (25 mM sodium borate, 25 mM NaCl, no EDTA, pH 8.0), DTT (50 mM in DI H_2O, 175 eq.) was added, and the reaction was incubated at 37 °C for 1 h. Upon completion, the conjugate was purified into BBS (25 mM sodium borate, 25 mM NaCl, no EDTA, pH 8.0) at 37 °C for 3 h to allow disulfide restoration. After this time, the conjugate was purified into DI H_2O. The resulting conjugate was analysed by LCMS analysis.

- 4 h timepoint

Results were obtained using general procedure 2.4.1.

a) Conjugation step (i)

Conjugate **S34**: Expected mass: 48078.55 Da, observed mass: 48079.18 Da

Hydrolysed conjugate **S27**: 47940.39 Da, observed mass: 47940.88 Da

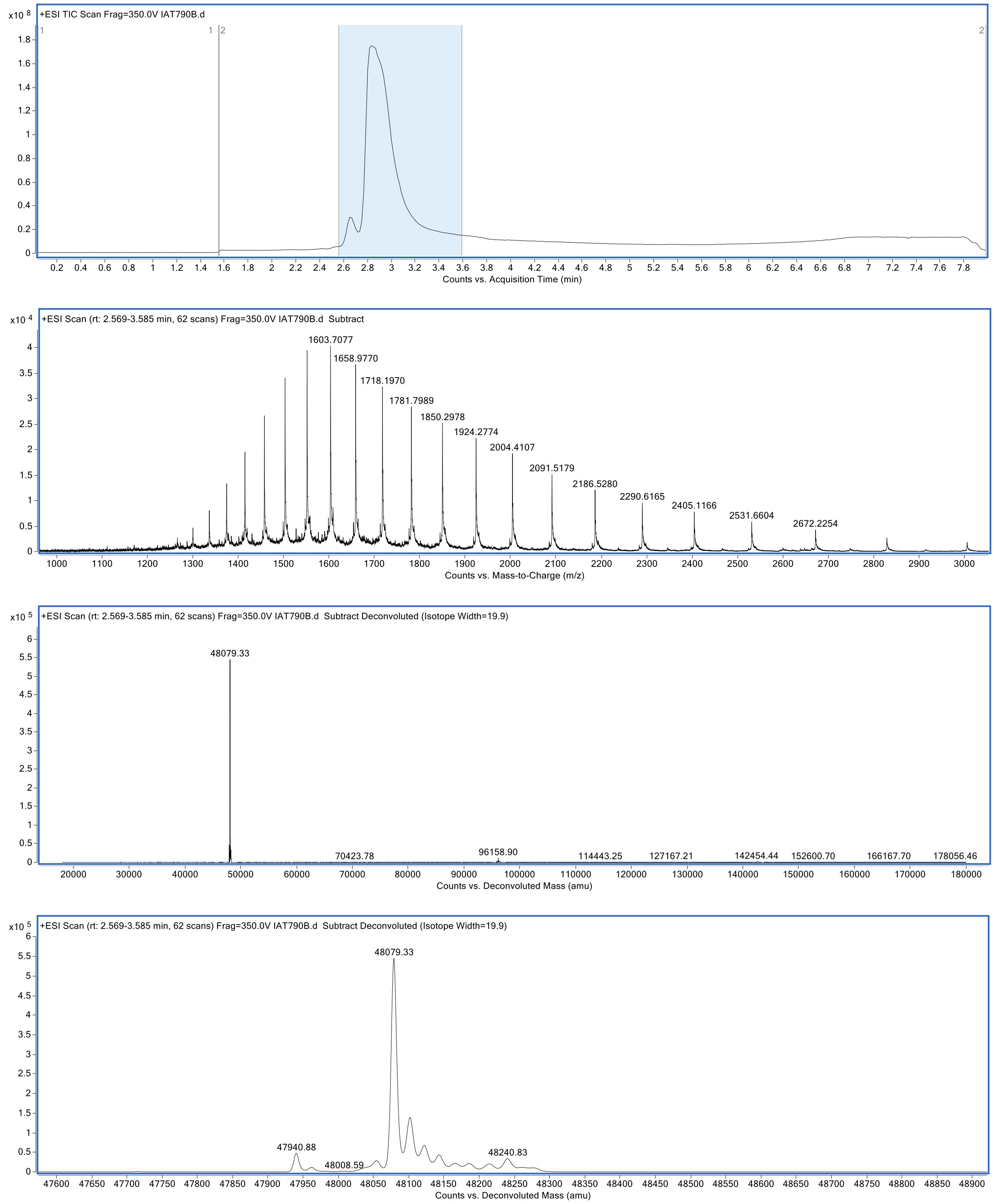

Figure S50: i) TIC LC-MS trace (top), (ii) non-deconvoluted LC-MS trace (upper middle), (iii) deconvoluted MS data (lower middle, wide range), (iv) zoom in mass range (bottom) for conjugation step.

b) Restoration step

Mono-labelled conjugate **3d**: Expected mass: 47779.27 Da, observed mass: 47777.91 Da **(%Abundance: 76%)**

Ontruzant Fab **1**: Expected mass: 47639.10 Da, observed mass: 47638.97 Da **(%Abundance in MS: 24%)**

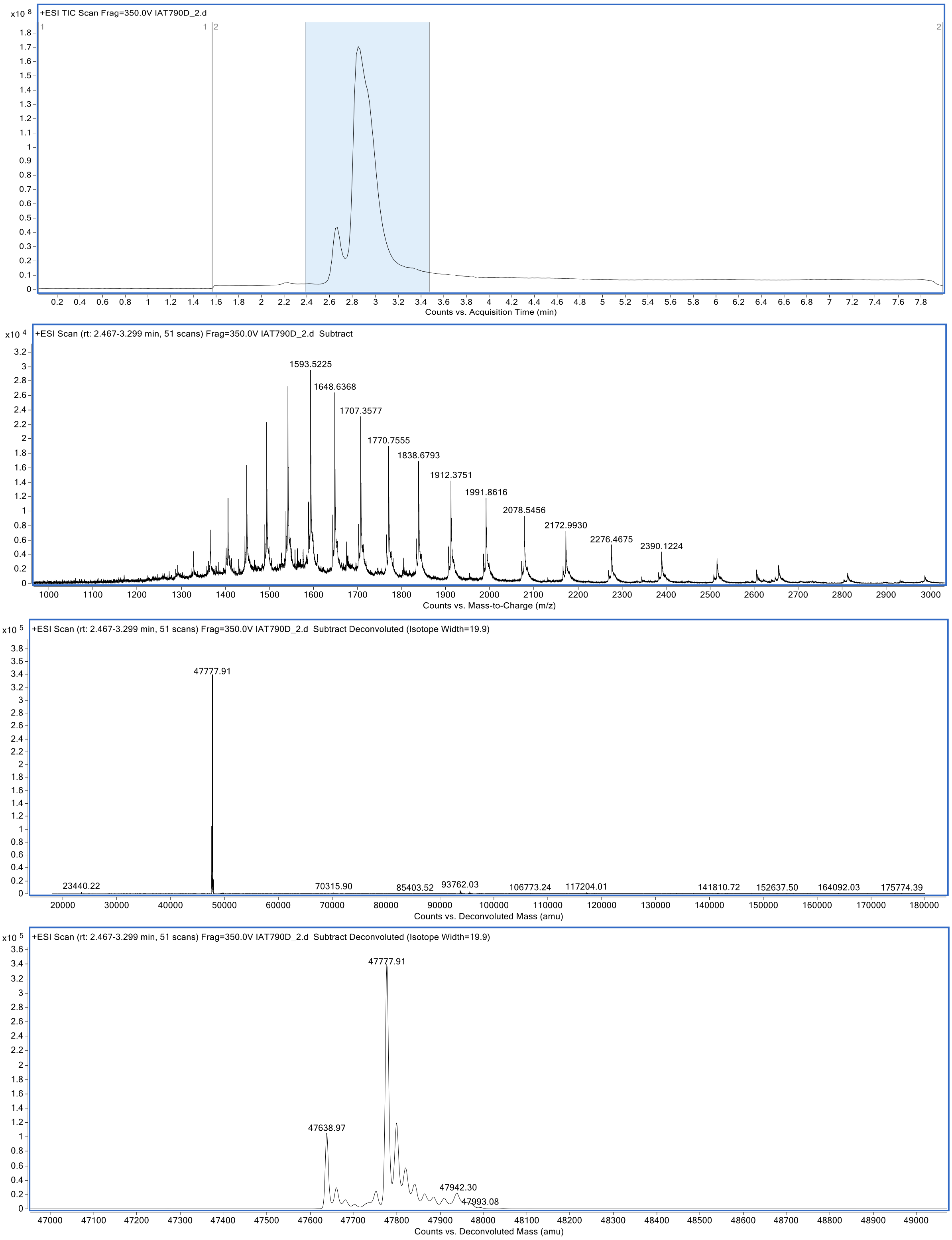

Figure S51: (i) TIC LC-MS trace (top), (ii) non-deconvoluted LC-MS trace (upper middle), (iii) deconvoluted MS data (lower middle, wide range), (iv) zoom in mass range (bottom) for restoration step.

- 6 h timepoint

Results were obtained using general procedure 2.4.1**.**

a) Conjugation step (i)

Expected mass **S34**: 48079.55 Da, observed mass: 48079.18 Da

Hydrolysed conjugate **S27**: 47940.39 Da, observed mass: 47940.88 Da

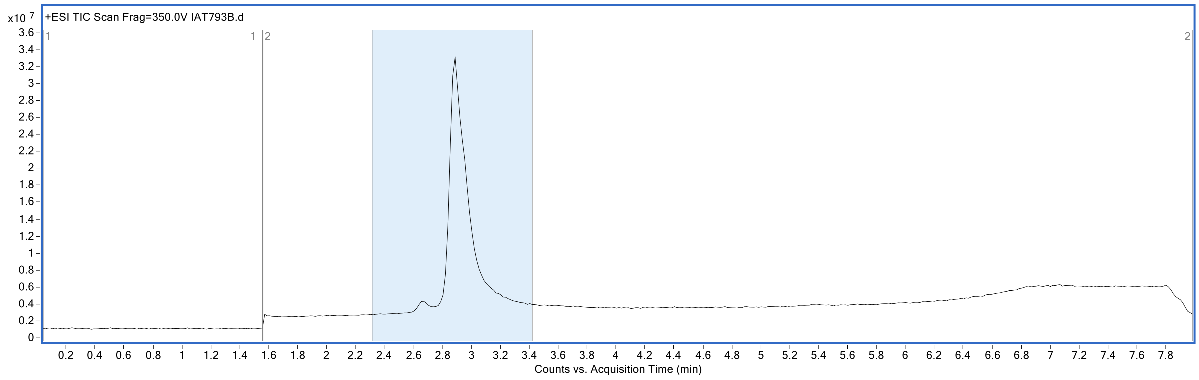

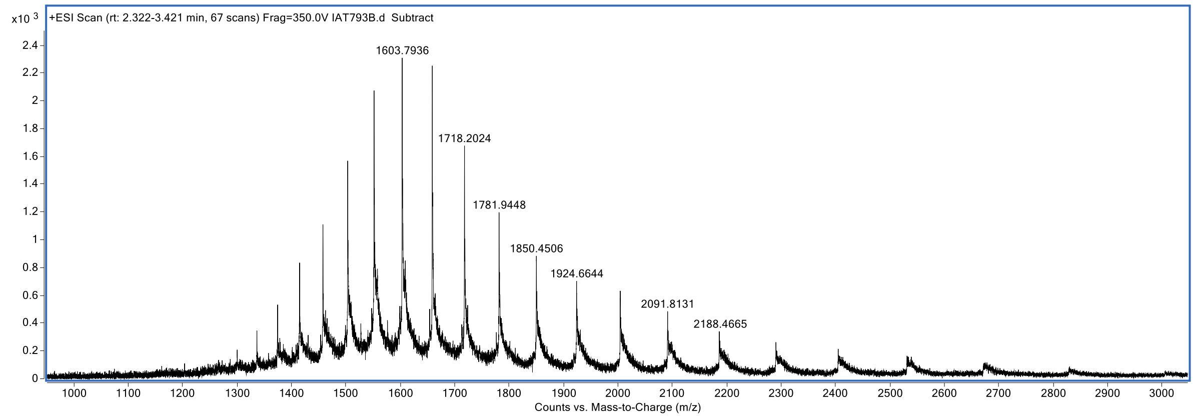

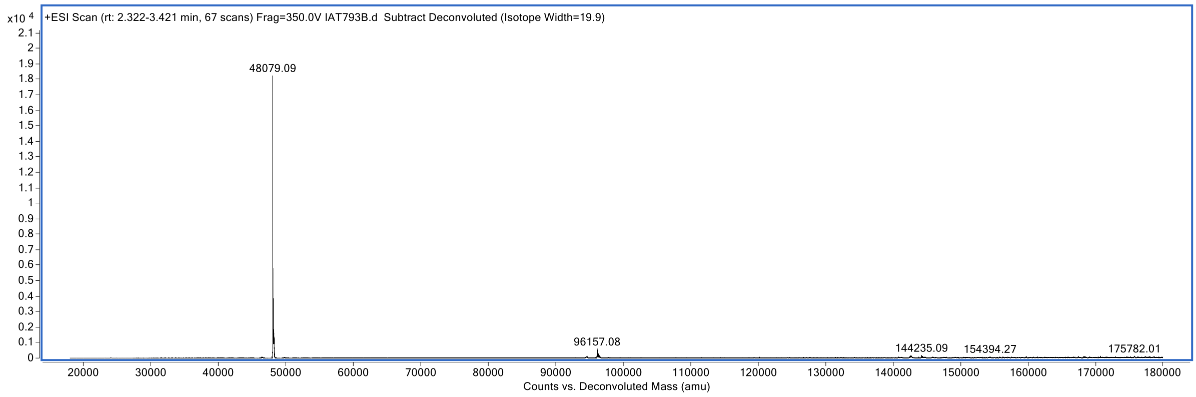

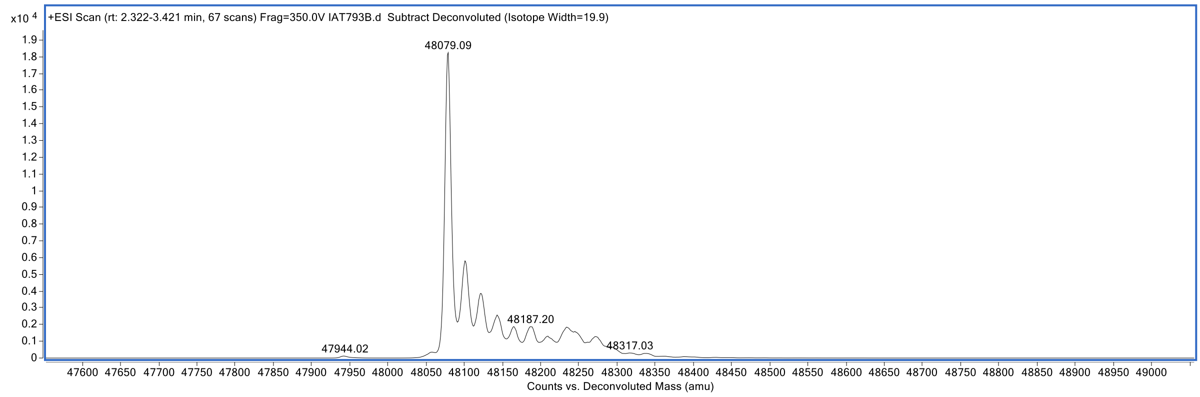

Figure S52: (i) TIC LC-MS trace (top), (ii) non-deconvoluted LC-MS trace (upper middle), (iii) deconvoluted MS data (lower middle, wide range), (iv) zoom in mass range (bottom) for conjugation step.

b) Restoration step

Mono-labelled conjugate **3d**: Expected mass: 47779.27 Da, observed mass: 47778.03 Da **(%Abundance: 87%)**

Ontruzant Fab **1**: Expected mass: 47639.10 Da, observed mass: 47640.13 Da **(%Abundance: 13%)**

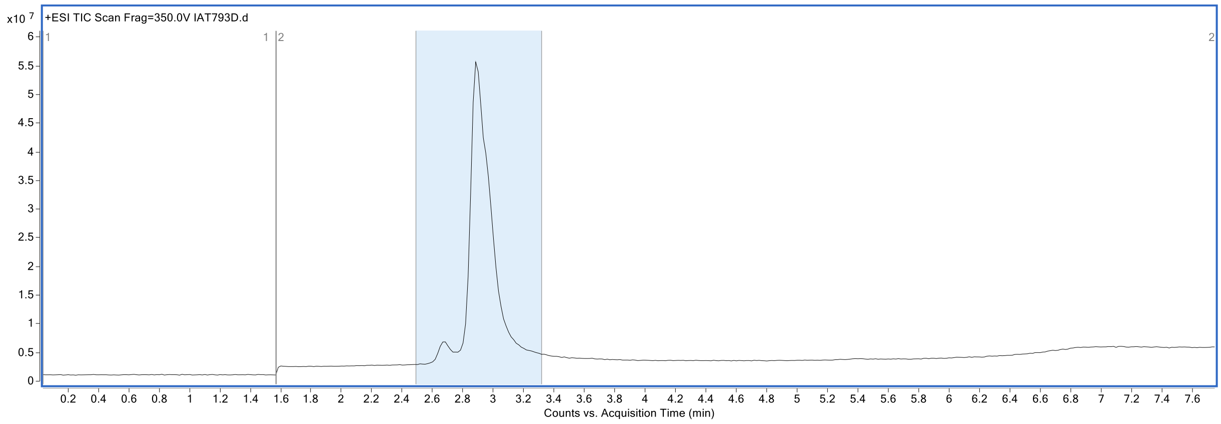

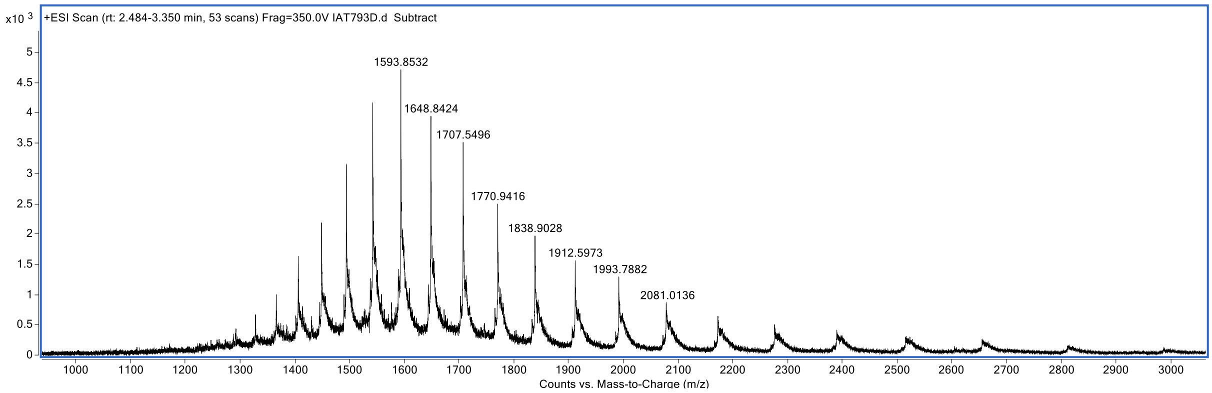

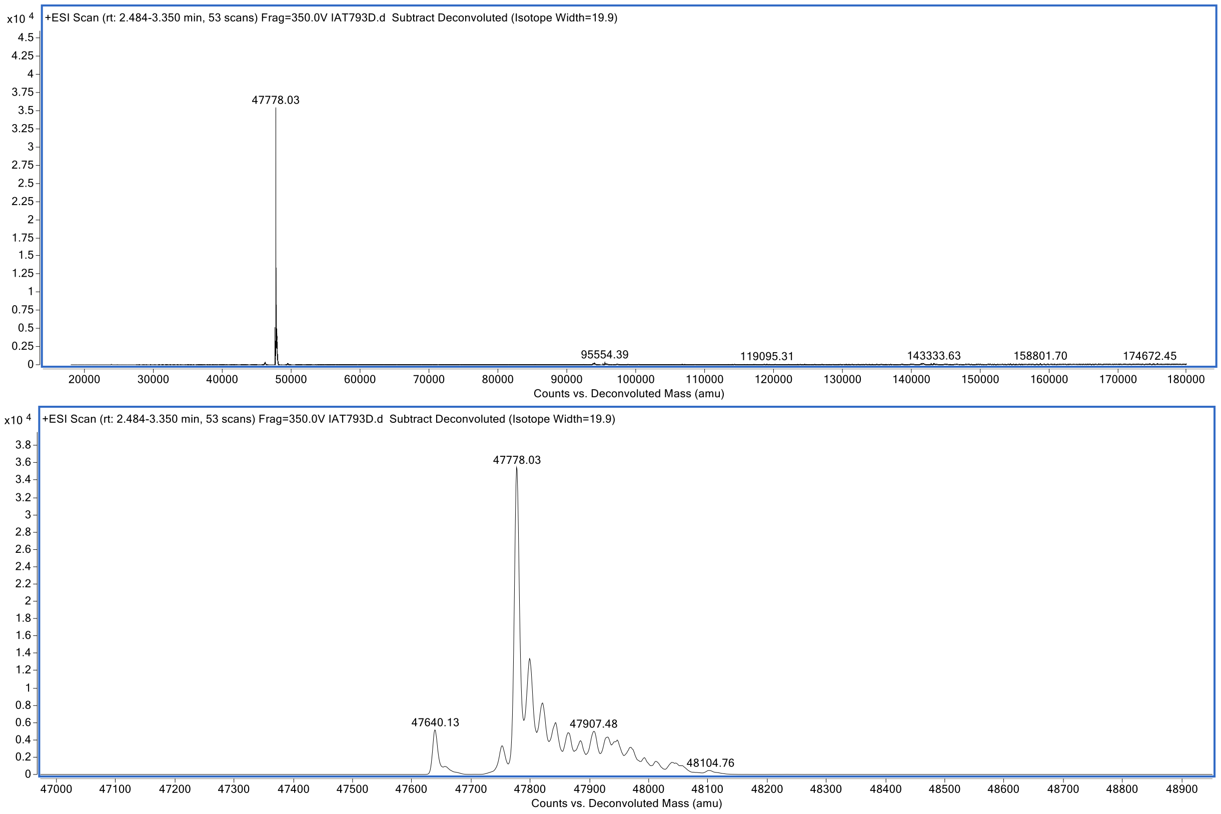

Figure S53: (i) TIC LC-MS trace (top), (ii) non-deconvoluted LC-MS trace (upper middle), (iii) deconvoluted MS data (lower middle, wide range), (iv) zoom in mass range (bottom) for restoration step.

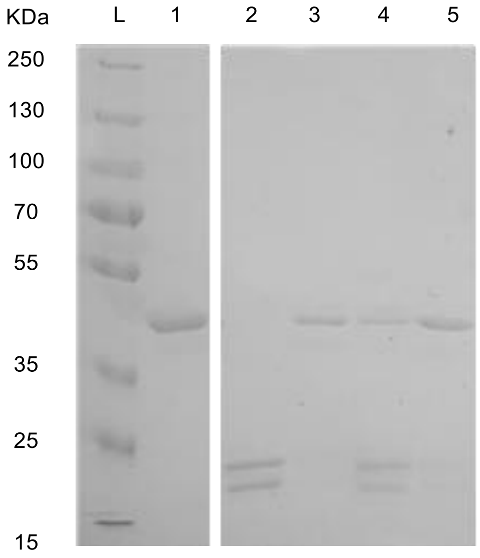

Figure S54: SDS-PAGE gel: Ladder, 1) Ontruzant Fab **1**, 2) Reduction step, 3) Conjugation step, 4) Deprotection step, 5) Disulfide restoration step.

- 8 h timepoint

Results were obtained using general procedure 2.4.1**.**

a) Conjugation step (i)

Conjugate **S34**: Expected mass: 48079.55 Da, observed mass: 48079.34 Da

Hydrolysed conjugate **S27**: 47940.39 Da, observed mass: 47940.88 Da

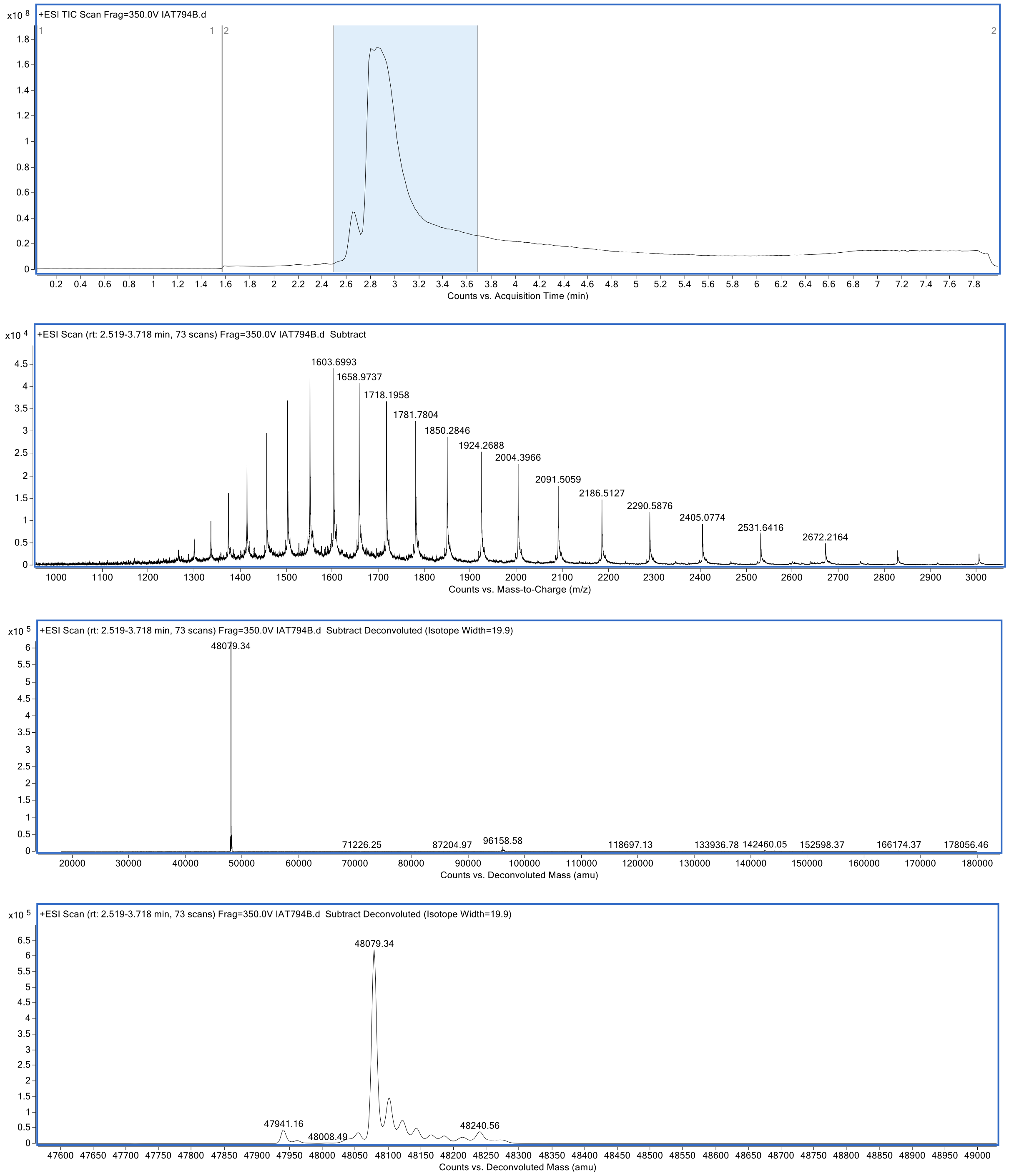

Figure S55: (i) TIC LC-MS trace (top), (ii) non-deconvoluted LC-MS trace (upper middle), (iii) deconvoluted MS data (lower middle, wide range), (iv) zoom in mass range (bottom) for conjugation step.

b) Restoration step

Mono-labelled conjugate **3d**: Expected mass: 47779.27 Da, observed mass: 47778.06 Da **(%Abundance in MS: 88%)**

Ontruzant Fab **1**: Expected mass: 47639.10 Da, observed mass: 47640.13 Da **(%Abundance in MS: 12%)**

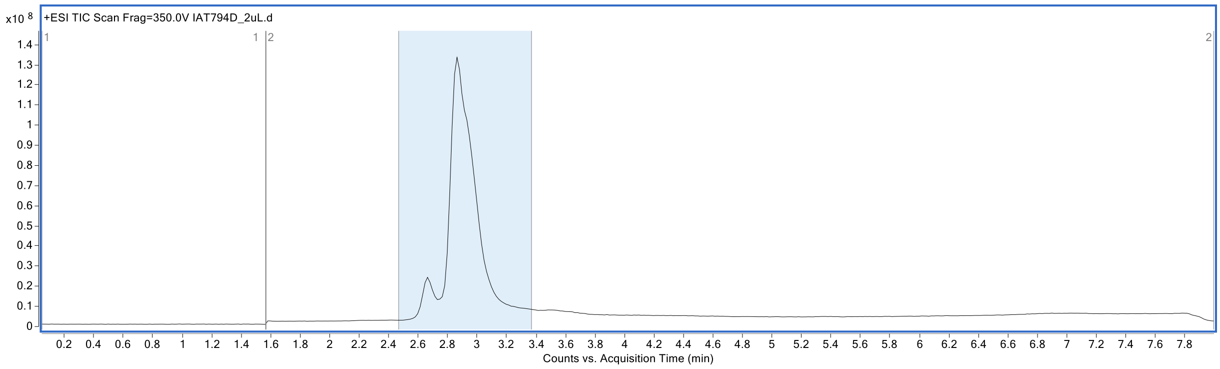

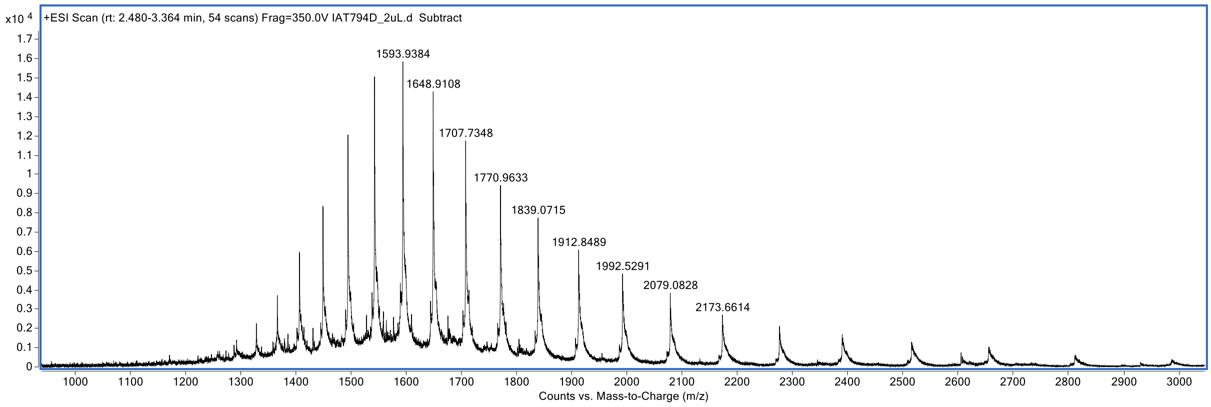

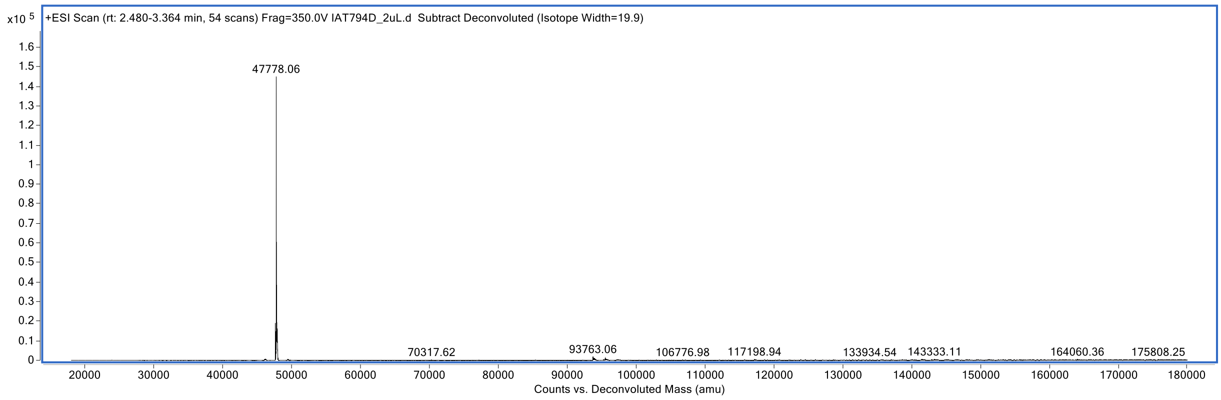

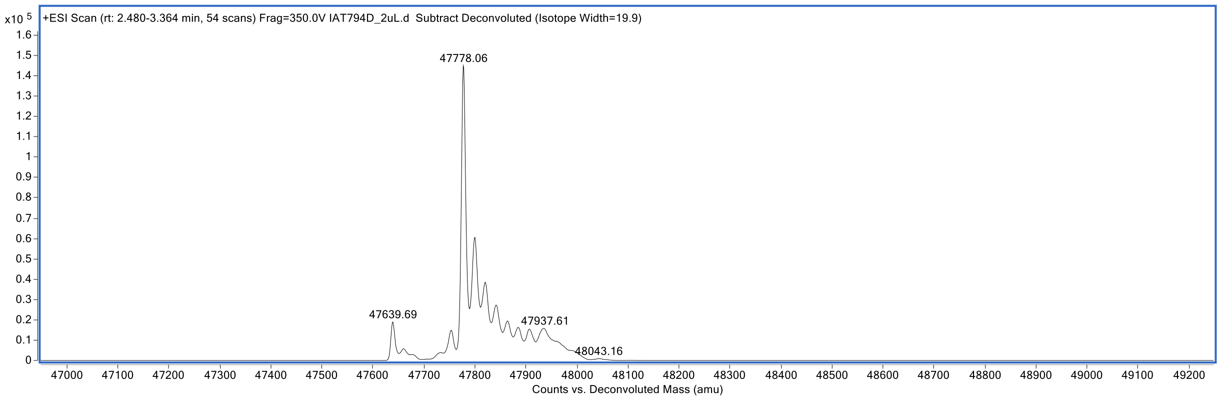

Figure S56: (i) TIC LC-MS trace (top), (ii) non-deconvoluted LC-MS trace (upper middle), (iii) deconvoluted MS data (lower middle, wide range), (iv) zoom in mass range (bottom) for restoration step.

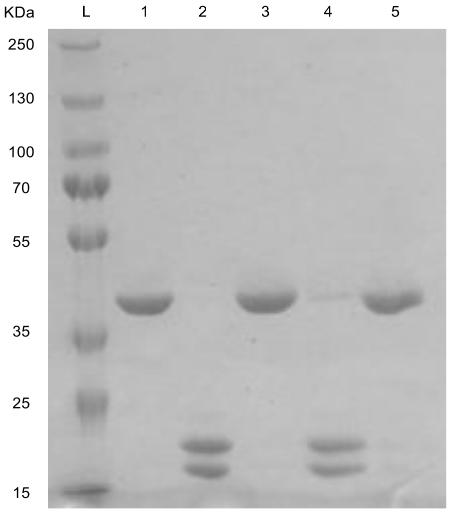

Figure S57: SDS-PAGE gel: L) Ladder, 1) Ontruzant Fab **1**, 2) Reduction step, 3) Conjugation step, 4) Deprotection step, 5) Disulfide restoration step.

- 16 h timepoint

Results were obtained using general procedure 2.4.1.

a) Conjugation step (i)

Conjugate **S34**: Expected mass: 48079.55 Da, observed mass: 48079.26 Da

Hydrolysed conjugate **S27**: 47940.39 Da, observed mass: 47941.05 Da

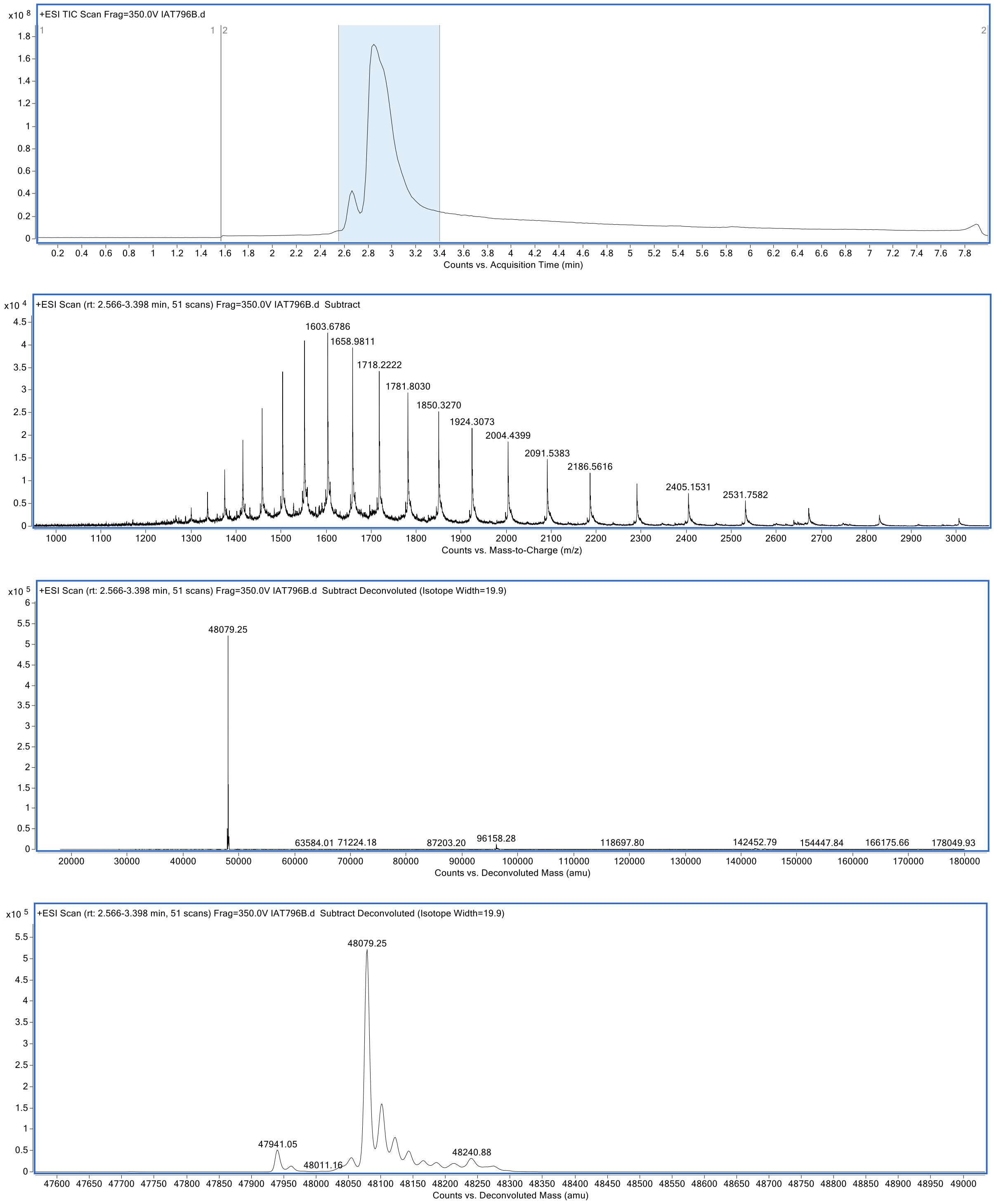

Figure S58: (i) TIC LC-MS trace (top), (ii) non-deconvoluted LC-MS trace (upper middle), (iii) deconvoluted MS data (lower middle, wide range), (iv) zoom in mass range (bottom) for conjugation step.

b) Restoration step

Mono-labelled conjugate **3d**: Expected mass: 47779.274Da, observed mass: 47778.06 Da **(%Abundance in MS: 90%)**

Ontruzant Fab **1**: Expected mass: 47639.10 Da, observed mass: 47640.13 Da **(%Abundance in MS: 10%)**

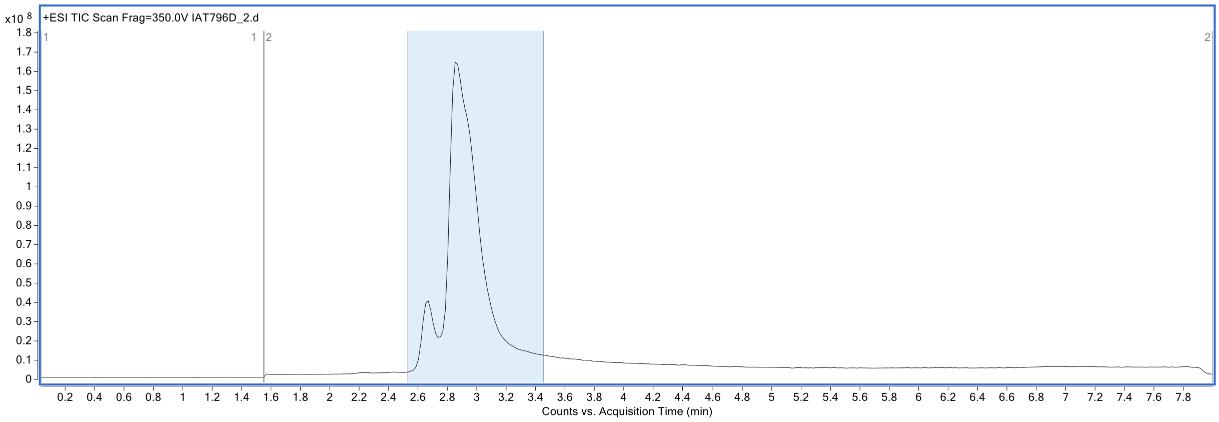

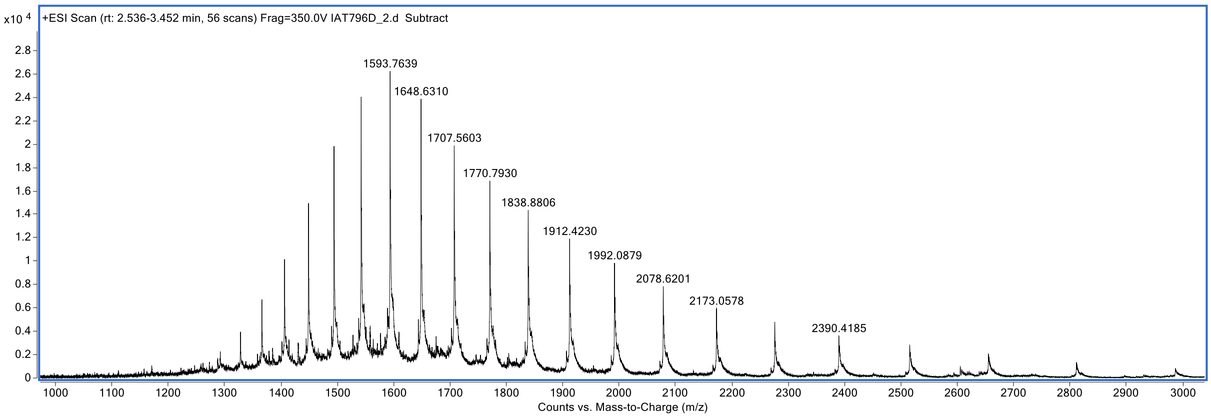

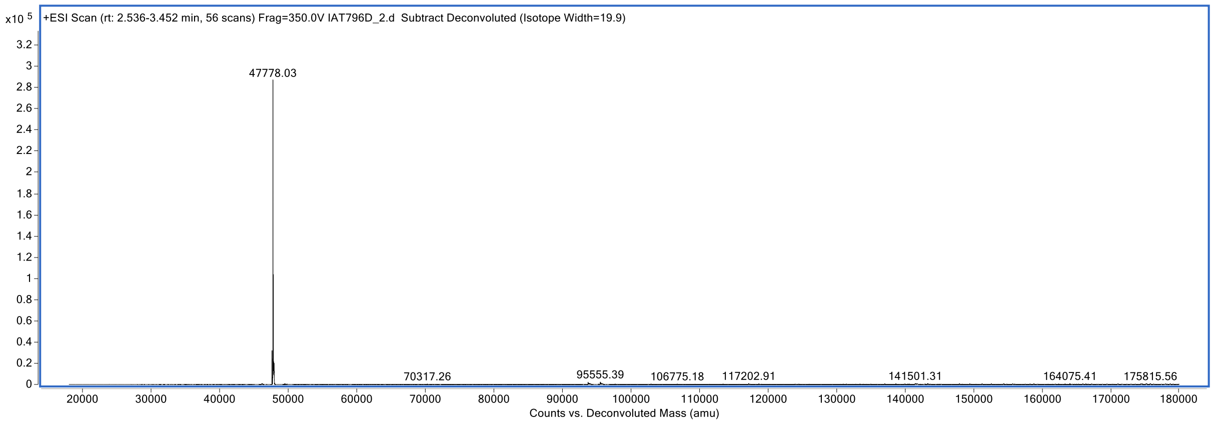

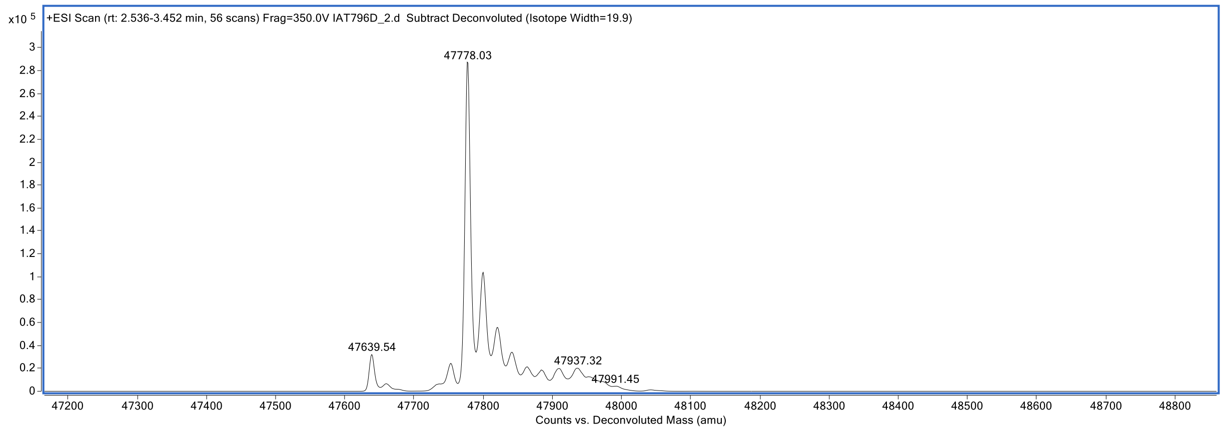

Figure S59: (i) TIC LC-MS trace (top), (ii) non-deconvoluted LC-MS trace (upper middle), (iii) deconvoluted MS data (lower middle, wide range), (iv) zoom in mass range (bottom) for restoration step.

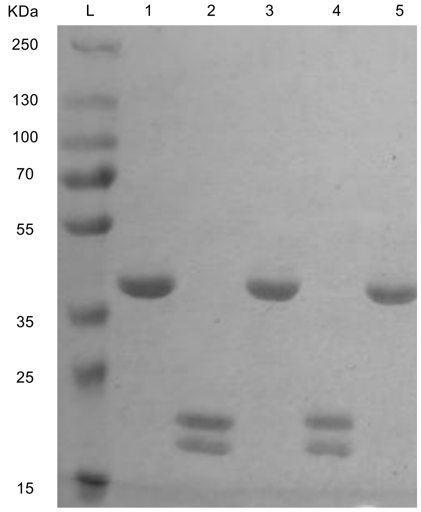

Figure S60: SDS-PAGE gel: L) Ladder, 1) Ontruzant Fab **1**, 2) Reduction step, 3) Conjugation step, 4) Deprotection step, 5) Disulfide restoration step.

2.5 Reaction of Ontruzant Fab 1 with reagent 2d (2.5 eq.)

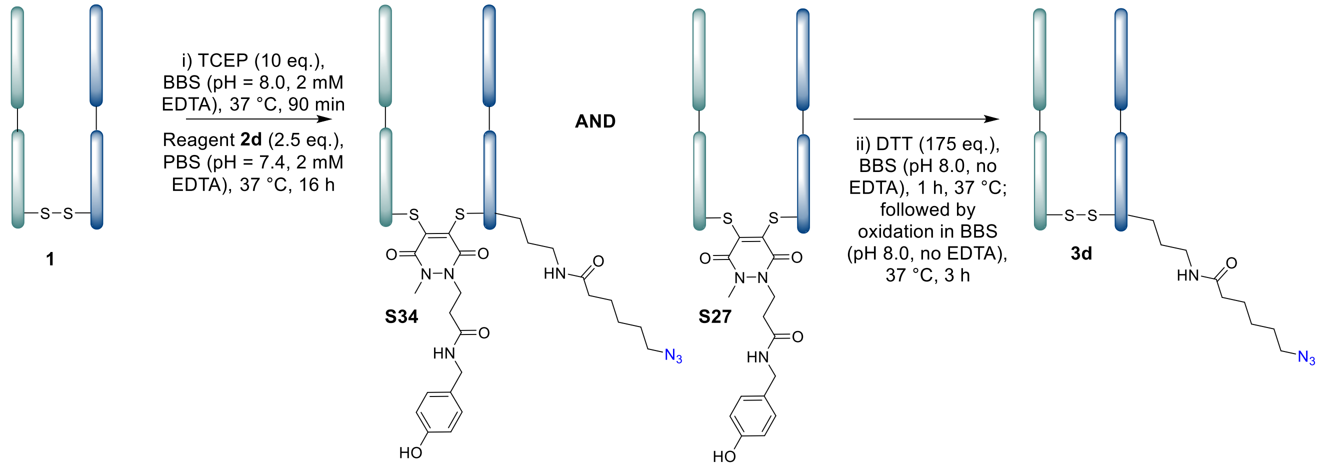

To a solution of Ontruzant Fab **1** (130 µL, 20 µM) in BBS (25 mM sodium borate, 25 mM NaCl, 2 mM EDTA, pH 8.0) was added TCEP·HCl (20 mM in DI H_2O, 2.5 eq.) The reaction was incubated at 37 °C for 90 min whilst shaking (300 rpm). Upon completion, the conjugate was purified into PBS (50 mM phosphate, 150 mM NaCl, 2 mM EDTA, pH 7.4) by centrifugation (7 K Zeba Spin desalting column), reagent **2d** (10 mM in MeCN, 10 eq.) was added and incubated at 37 °C for 16 h whilst shaking at (300 rpm). Upon completion, the conjugate was purified into BBS (25 mM sodium borate, 25 mM NaCl, no EDTA, pH 8.0), DTT (50 mM in DI H_2O, 175 eq.) was added, and the reaction was incubated at 37 °C for 1 h. Upon completion, the conjugate was purified into DI H_2O. The resulting conjugate was analysed by LCMS analysis.

a) Conjugation step (i)

Conjugate **S34**: Expected mass: 48079.55 Da, observed mass: 48079.06 Da

Hydrolysed conjugate **S27**: 47940.39 Da, observed mass: 47940.45 Da

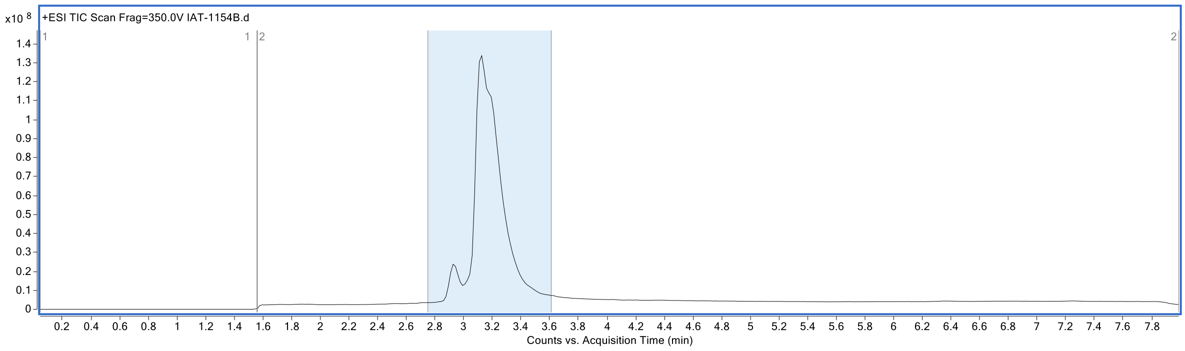

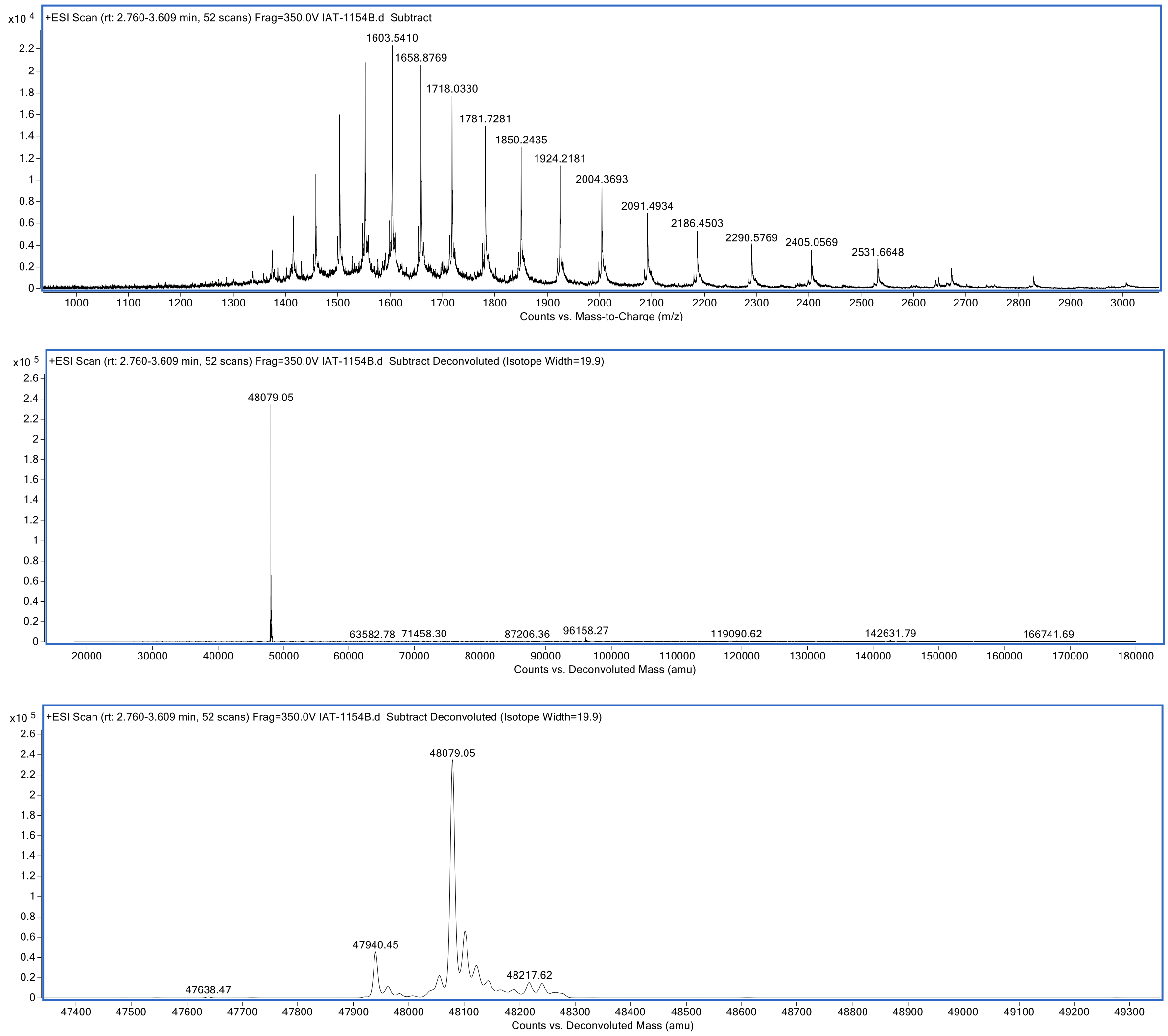

Figure S61: (i) TIC LC-MS trace (top), (ii) non-deconvoluted LC-MS trace (upper middle), (iii) deconvoluted MS data (lower middle, wide range), (iv) zoom in mass range (bottom) for conjugation step.

b) Restoration step

Mono-labelled conjugate **3d**: Expected mass: 47779.27 Da, observed mass: 47777.94 Da **(%Abundance: 90%)**

Ontruzant Fab **1**: Expected mass: 47639.10 Da, observed mass: 47639.01 Da **(%Abundance: 10%)**

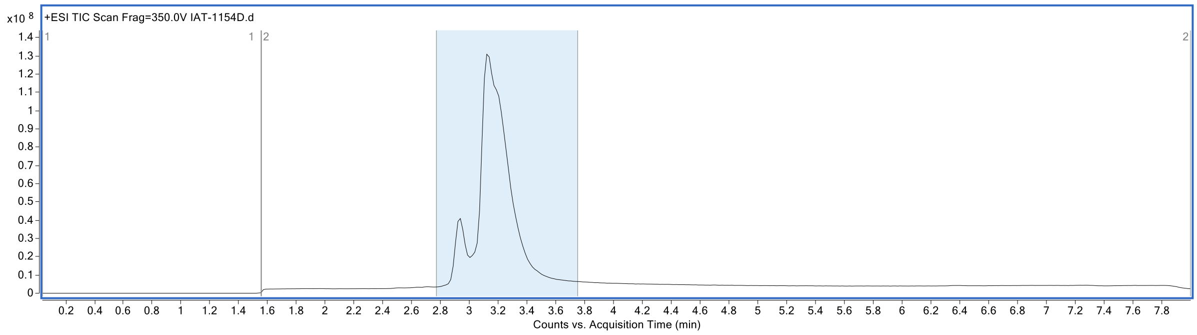

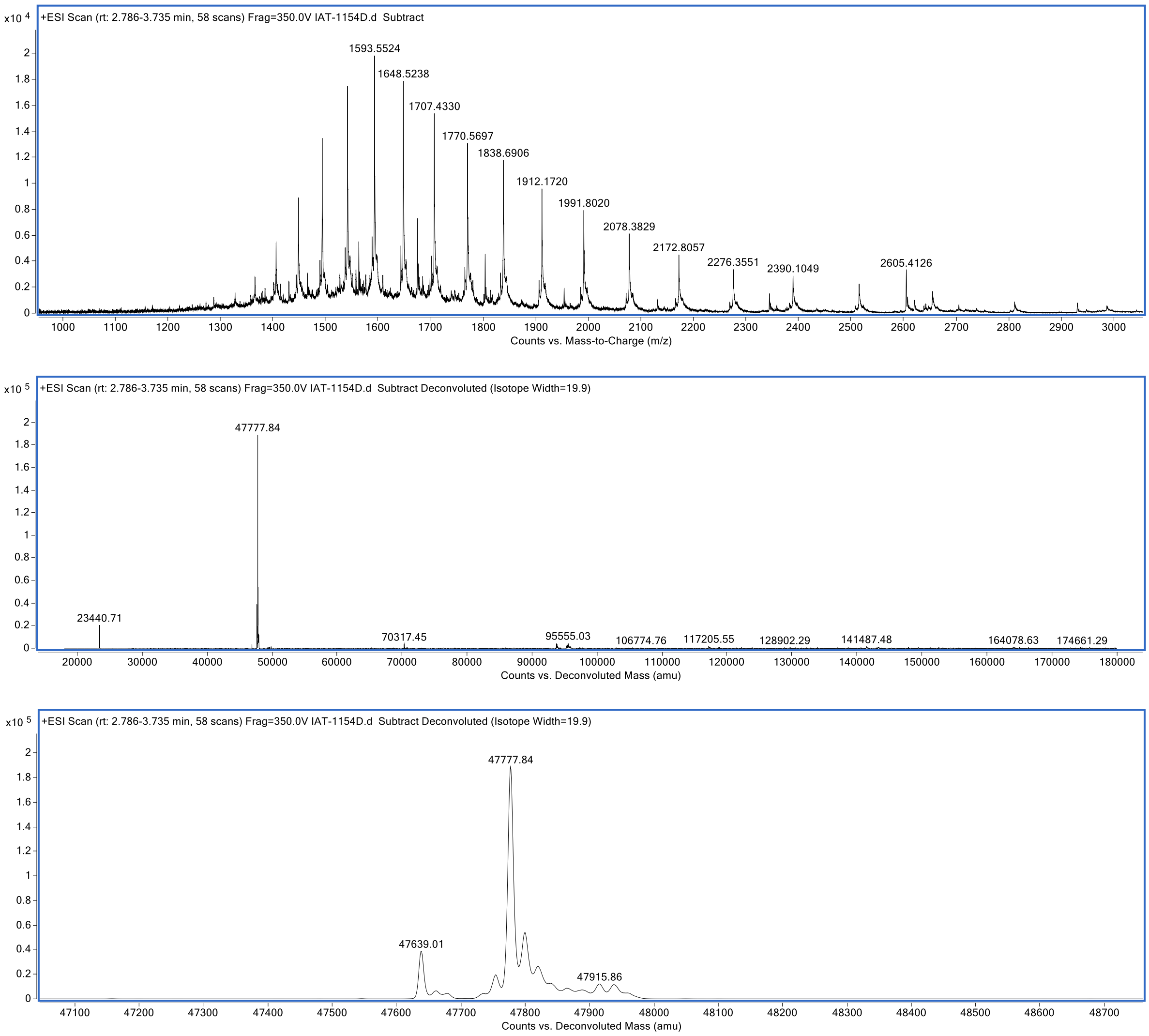

Figure S62: (i) TIC LC-MS trace (top), (ii) non-deconvoluted LC-MS trace (upper middle), (iii) deconvoluted MS data (lower middle, wide range), (iv) zoom in mass range (bottom) for restoration step.

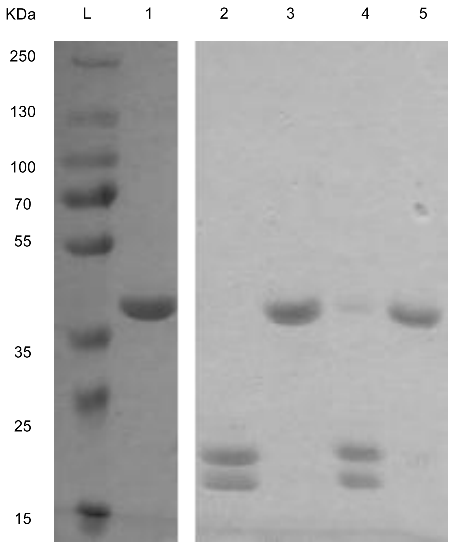

Figure S63: SDS-PAGE gel: L) Ladder, 1) Ontruzant Fab **1**, 2) Reduction step, 3) Conjugation step, 4) Deprotection step, 5) Disulfide restoration step.

2.6 Appraisal of reagents 4, 5 and 6 on Ontruzant Fab 1

2.6.1. General experimental procedure

To a solution of Ontruzant Fab **1** (130 µL, 20 µM) in BBS (25 mM sodium borate, 25 mM NaCl, 2 mM EDTA, pH 8.0) was added TCEP·HCl (20 mM in DI H_2O, 10 eq.) The reaction was incubated at 37 °C for 90 min whilst shaking (300 rpm). Upon completion, the conjugate was purified into PBS (50 mM phosphate, 150 mM NaCl, 2 mM EDTA, pH 7.4) by centrifugation (7k Zeba Spin desalting column) and reagent **4/5/6** (10 mM in MeCN, 2.5 eq.) was added, and incubated at 37 °C for 6 h whilst shaking (300 rpm). After this time, the conjugate was purified into BBS (25 mM sodium borate, 25 mM NaCl, no EDTA, pH 8.0), DTT (50 mM in DI H_2O, 175 eq.) was added, and the reaction was incubated at 37 °C for 1 h. Upon completion, the conjugate was purified into BBS (25 mM sodium borate, 25 mM NaCl, no EDTA, pH 8.0) at 37 °C for 3 h to allow disulfide restoration. After this time, the conjugate was purified into DI H_2O. The resulting conjugate was analysed by LCMS.

2.6.2 Reaction of Ontruzant Fab **1** with reagent **4**

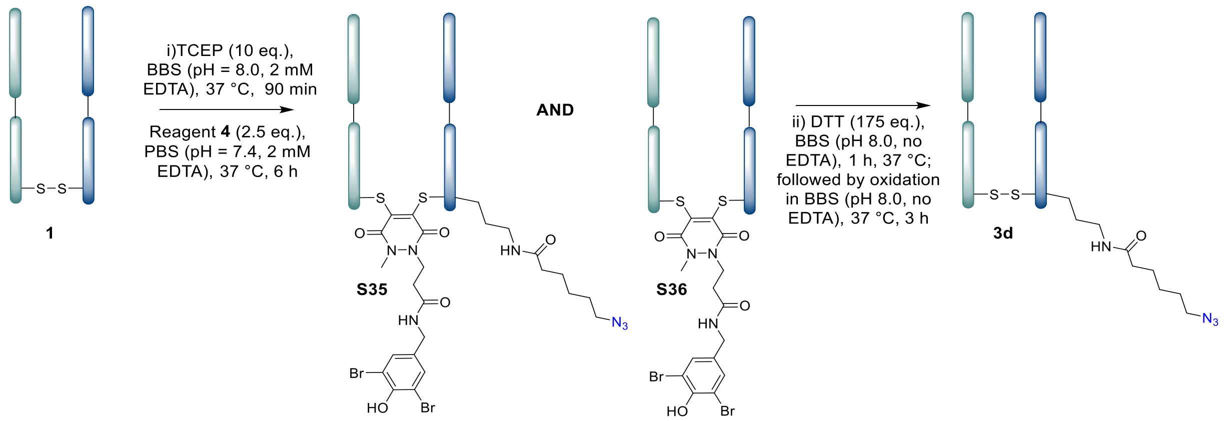

Results were obtained using general procedure 2.6.1, using PD reagent **4**.

a) Conjugation step (i)

Conjugate **S35**: Expected mass: 48236.34 Da, observed mass: 48237.12 Da

Hydrolysed conjugate **S36**: Expected mass: 48098.18 Da, observed mass: 48099.01 Da

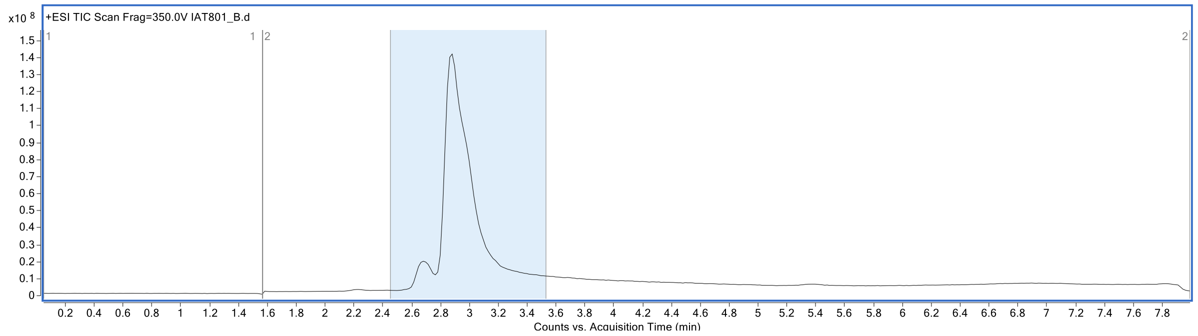

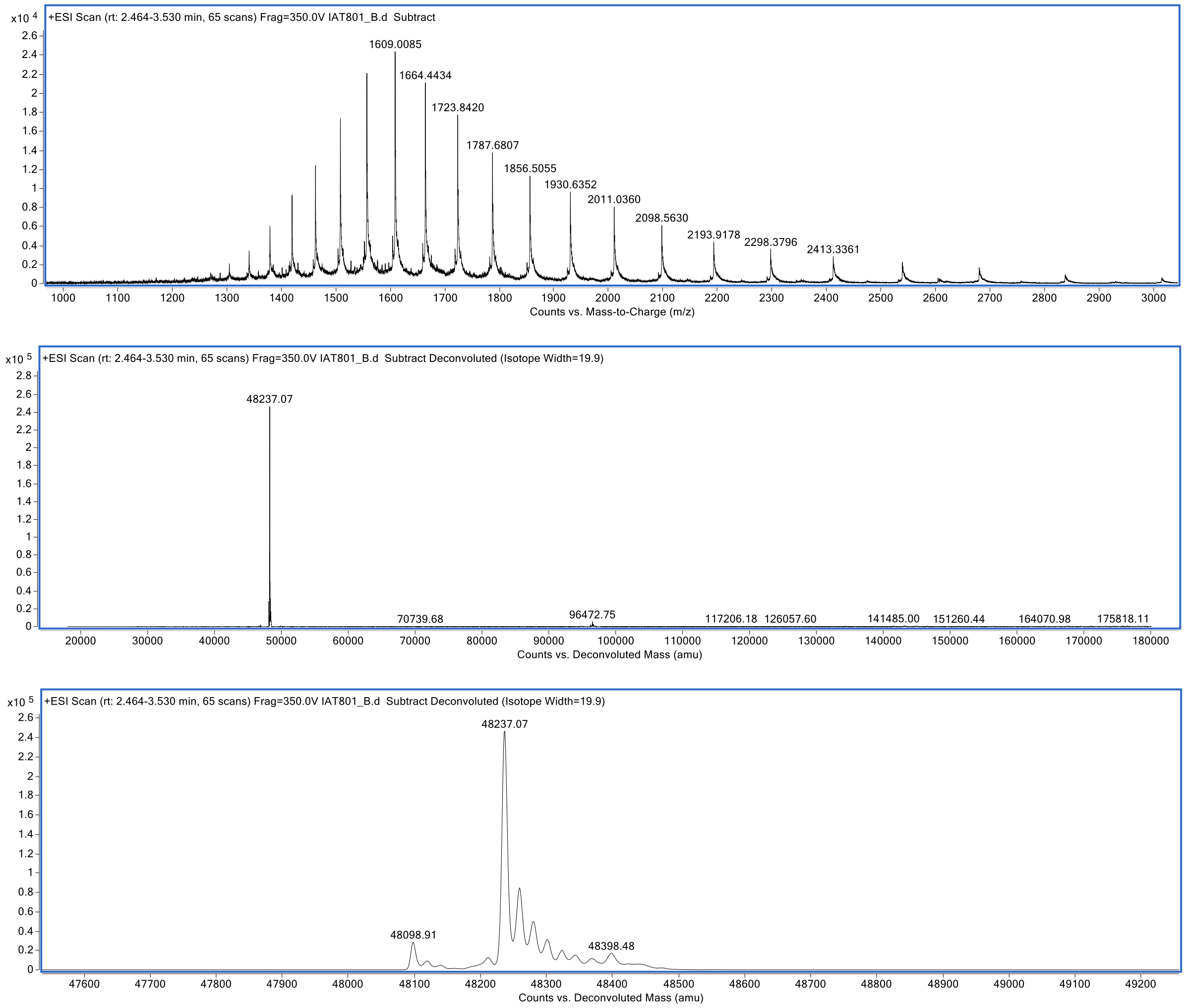

Figure S64: (i) TIC LC-MS trace (top), (ii) non-deconvoluted LC-MS trace (upper middle), (iii) deconvoluted MS data (lower middle, wide range), (iv) bottom (zoom in mass range) for conjugation step.

b) Restoration step

Mono-labelled conjugate **3d**: Expected mass: 47779.27 Da, observed mass: 47778.09 Da **(%Abundance: 93%)**

Ontruzant Fab **1**: Expected mass: 47639.10 Da, observed mass: 47639.50 Da **(%Abundance: 7%)**

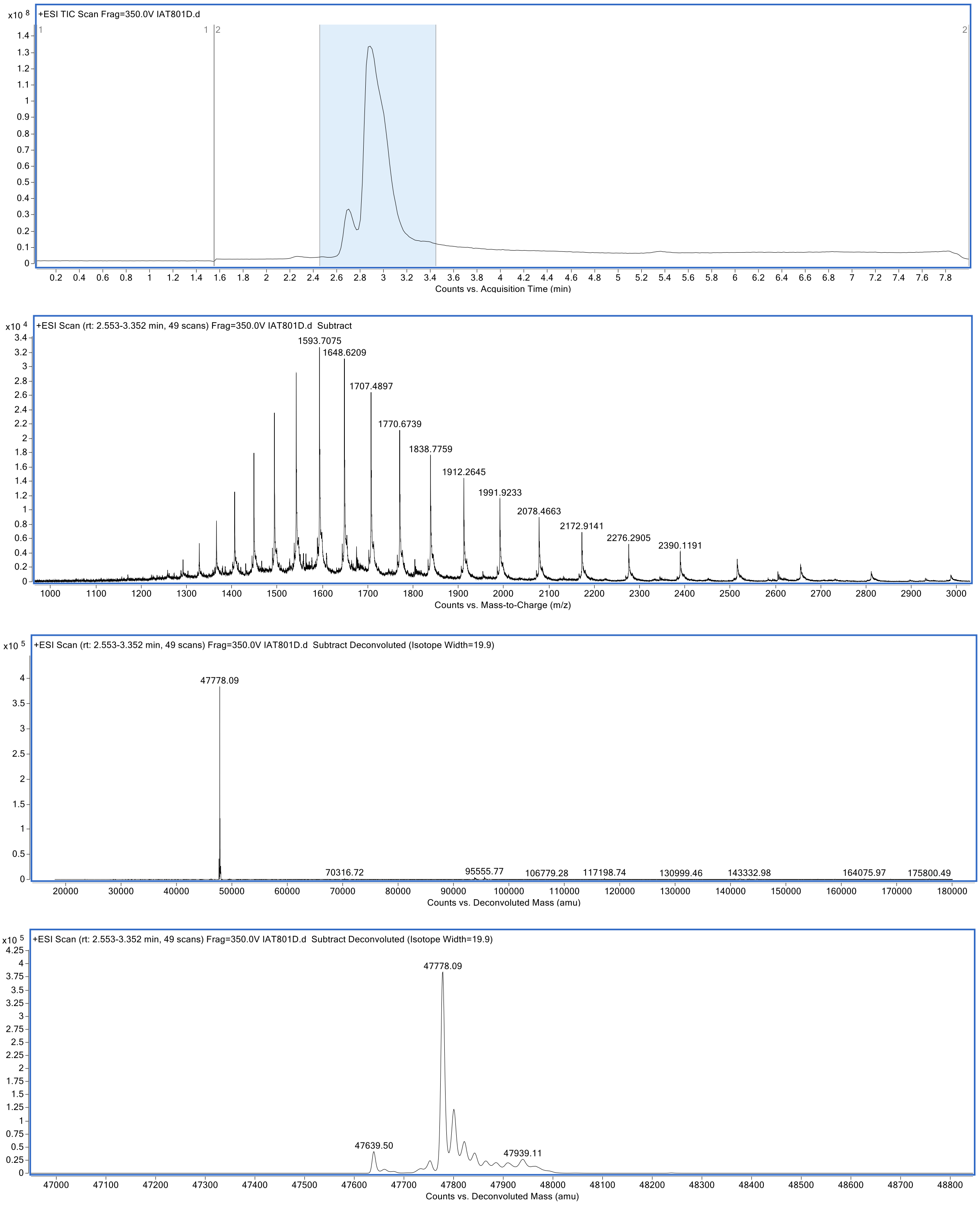

Figure S65: (i) TIC LC-MS trace (top), (ii) non-deconvoluted LC-MS trace (upper middle), (iii) deconvoluted MS data (lower middle, wide range), (iv) bottom (zoom in mass range) for restoration step.

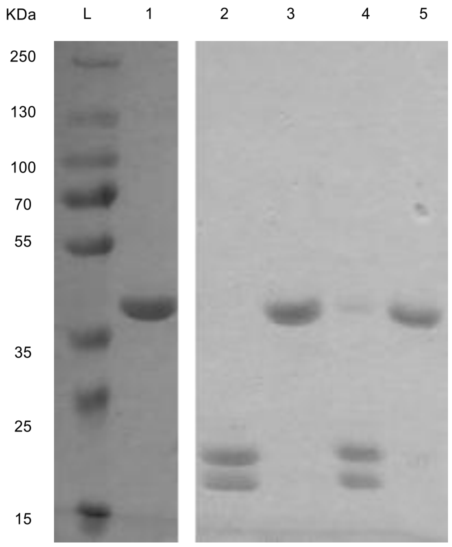

Figure S66: SDS-PAGE gel: L) Ladder, 1) Ontruzant Fab **1**, 2) Reduction step, 3) Conjugation/Lysine reaction step, 4) Deprotection step, 5) Disulfide restoration step.

2.6.2.1 Control reaction of Ontruzant Fab **1** (non-reduced) with reagent **4**

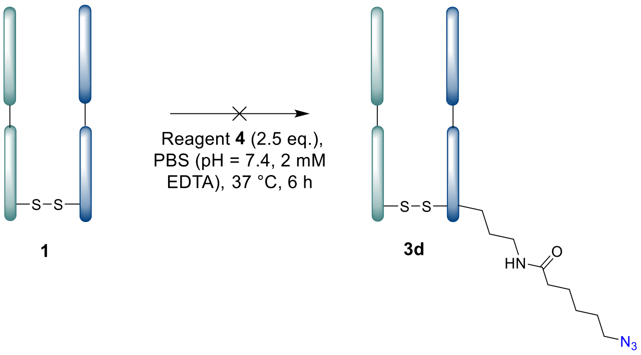

To a solution of Ontruzant Fab **1** (130 µL, 20 µM) in PBS (25 mM sodium borate, 25 mM NaCl, 2 mM EDTA, pH 7.4) was added reagent **4** (10 mM in MeCN, 2.5 equiv.) was added, and incubated at 37 °C for 6 h whilst shaking (300 rpm). After this time, the conjugate was purified into DI H_2O. The resulting conjugate was analysed by LC-MS.

Ontruzant Fab **1**: Expected mass: 47639.10 Da, observed mass: 47639.02 Da

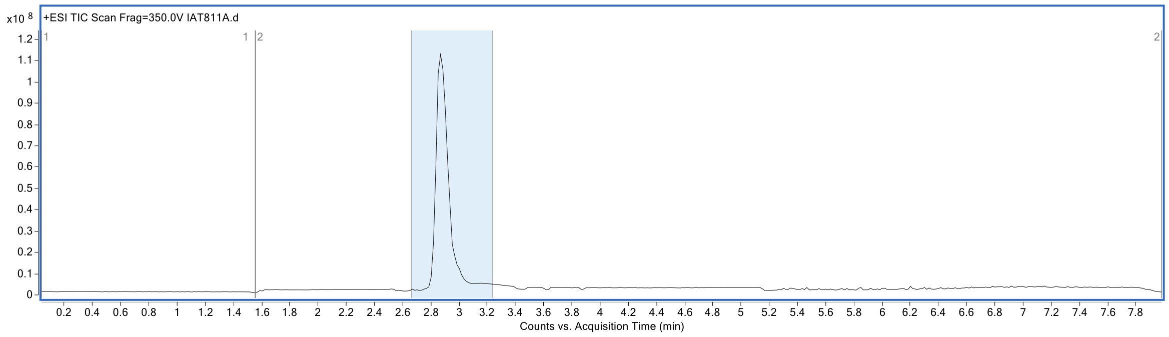

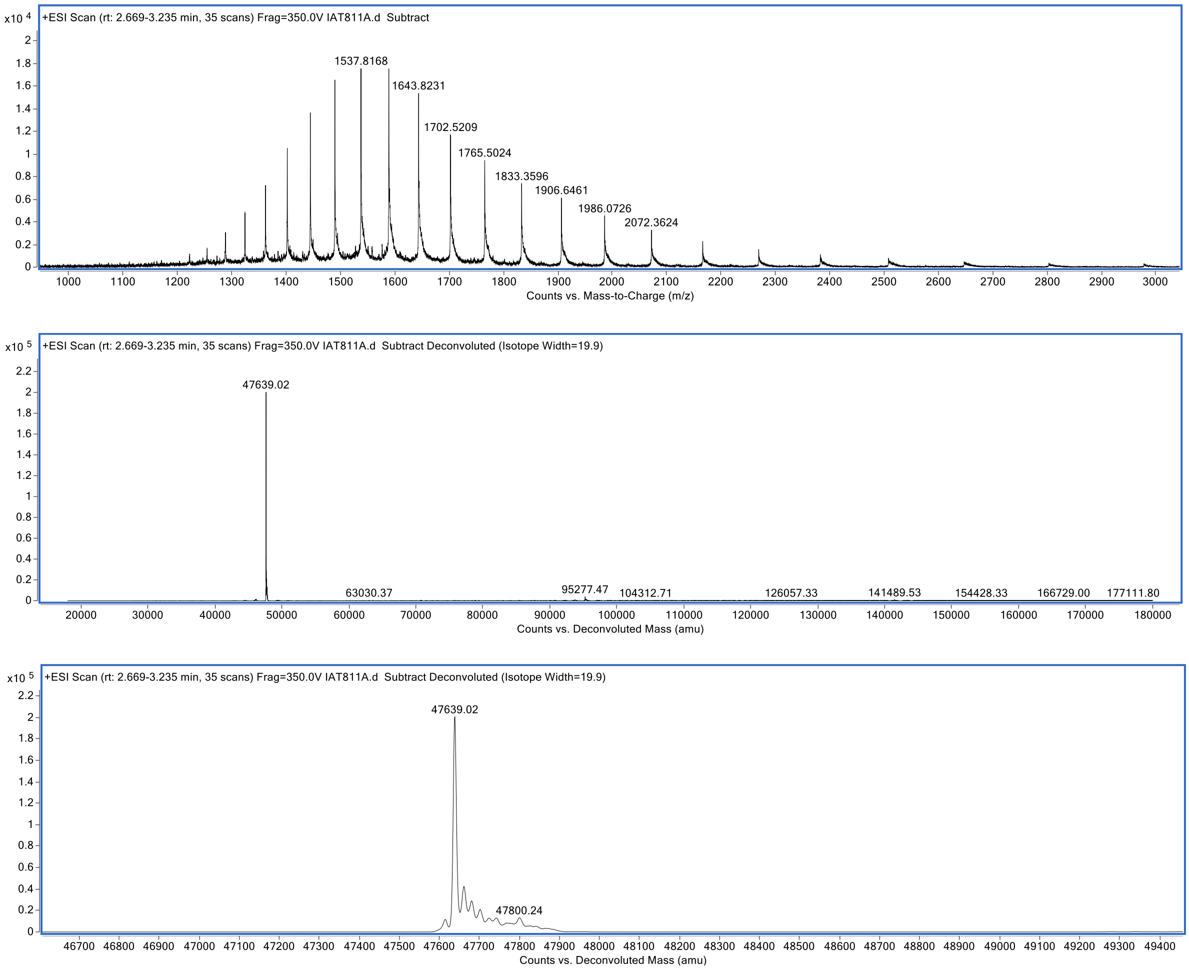

Figure S65a: (i) TIC LC-MS trace (top), (ii) non-deconvoluted LC-MS trace (upper middle), (iii) deconvoluted MS data (lower middle, wide range), (iv) bottom (zoom in mass range) for restoration step.

2.6.3 Reaction of Ontruzant Fab **1** with reagent **5**

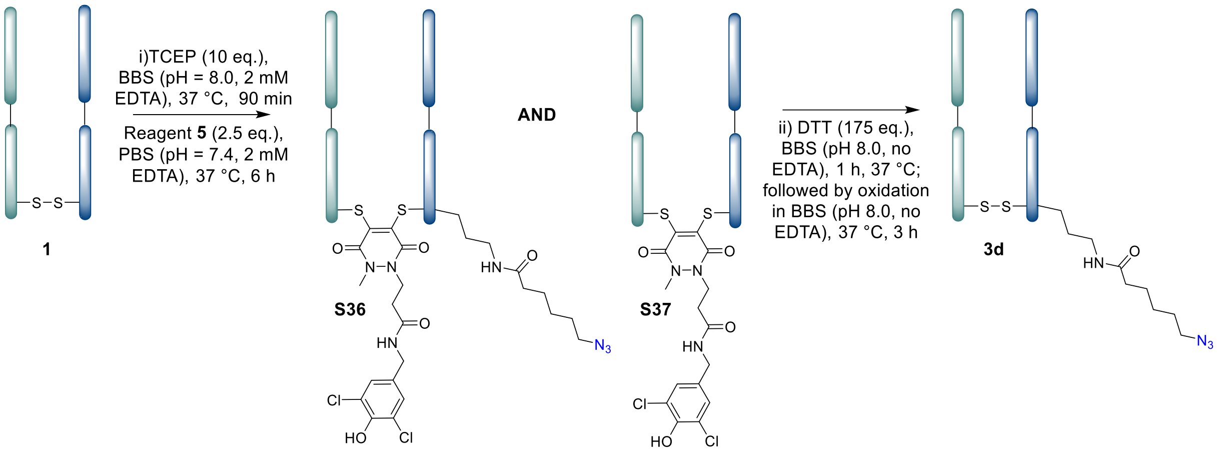

Results were obtained using general procedure 2.6.1., using PD reagent **5**.

a) Conjugation step (i)

Conjugate **S36**: Expected mass: 48146.43 Da, observed mass: 48147.85 Da

Hydrolysed conjugate **S37**: Expected mass: 48008.27 Da, observed mass: 48009.31 Da

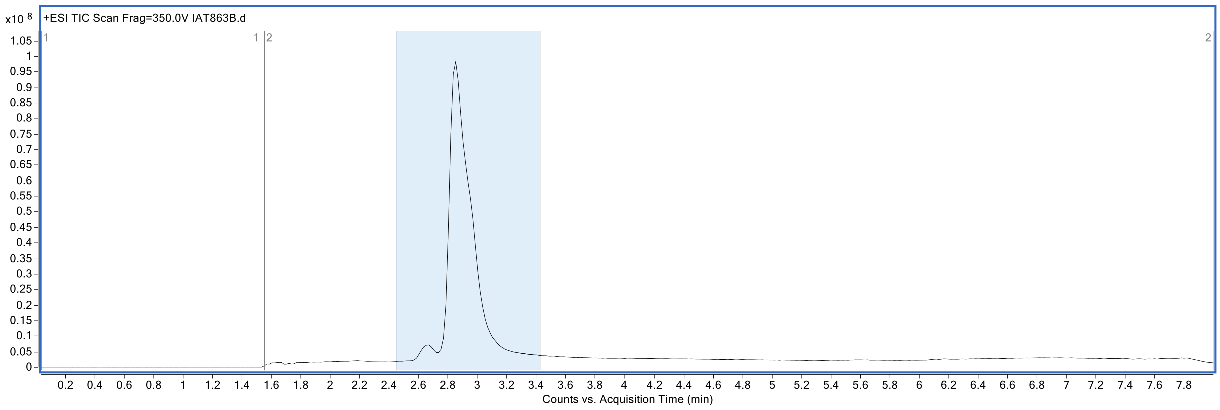

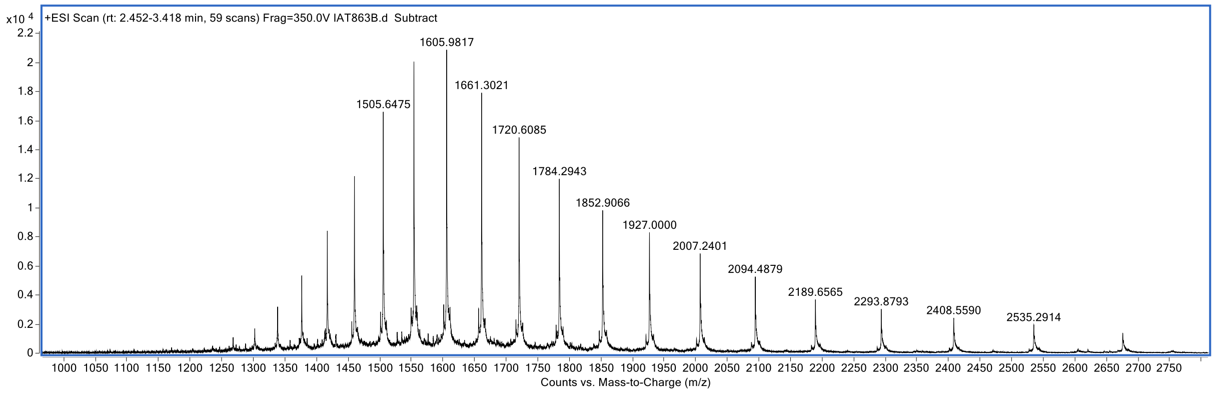

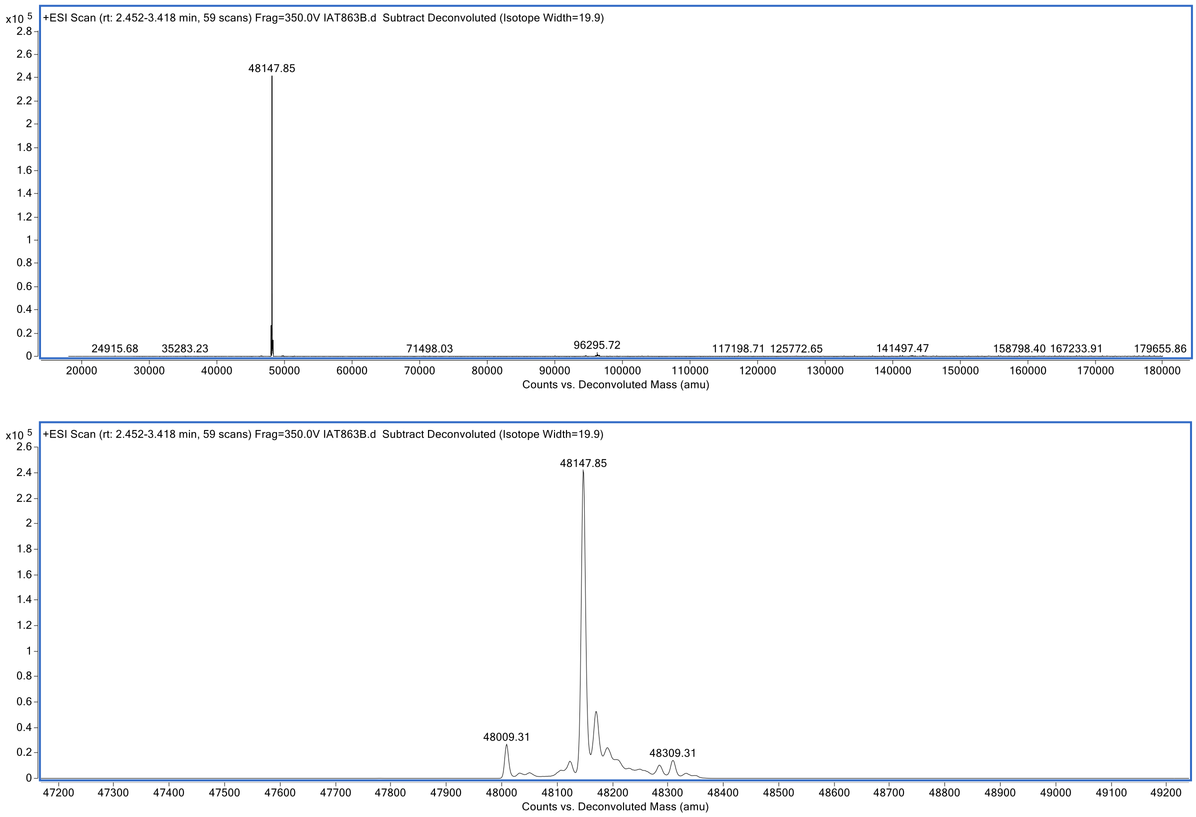

Figure S67: (i) TIC LC-MS trace (top), (ii) non-deconvoluted LC-MS trace (upper middle), (iii) deconvoluted MS data (lower middle, wide range), (iv) bottom (zoom in mass range) for conjugation step.

b) Restoration step

Mono-labelled conjugate **3d**: Expected mass: 47779.27 Da, observed mass: 47777.84 Da **(%Abundance in MS: 90%)**

Ontruzant Fab **1**: Expected mass: 47639.10 Da, observed mass: 47638.79 Da **(%Abundance in MS: 10%)**

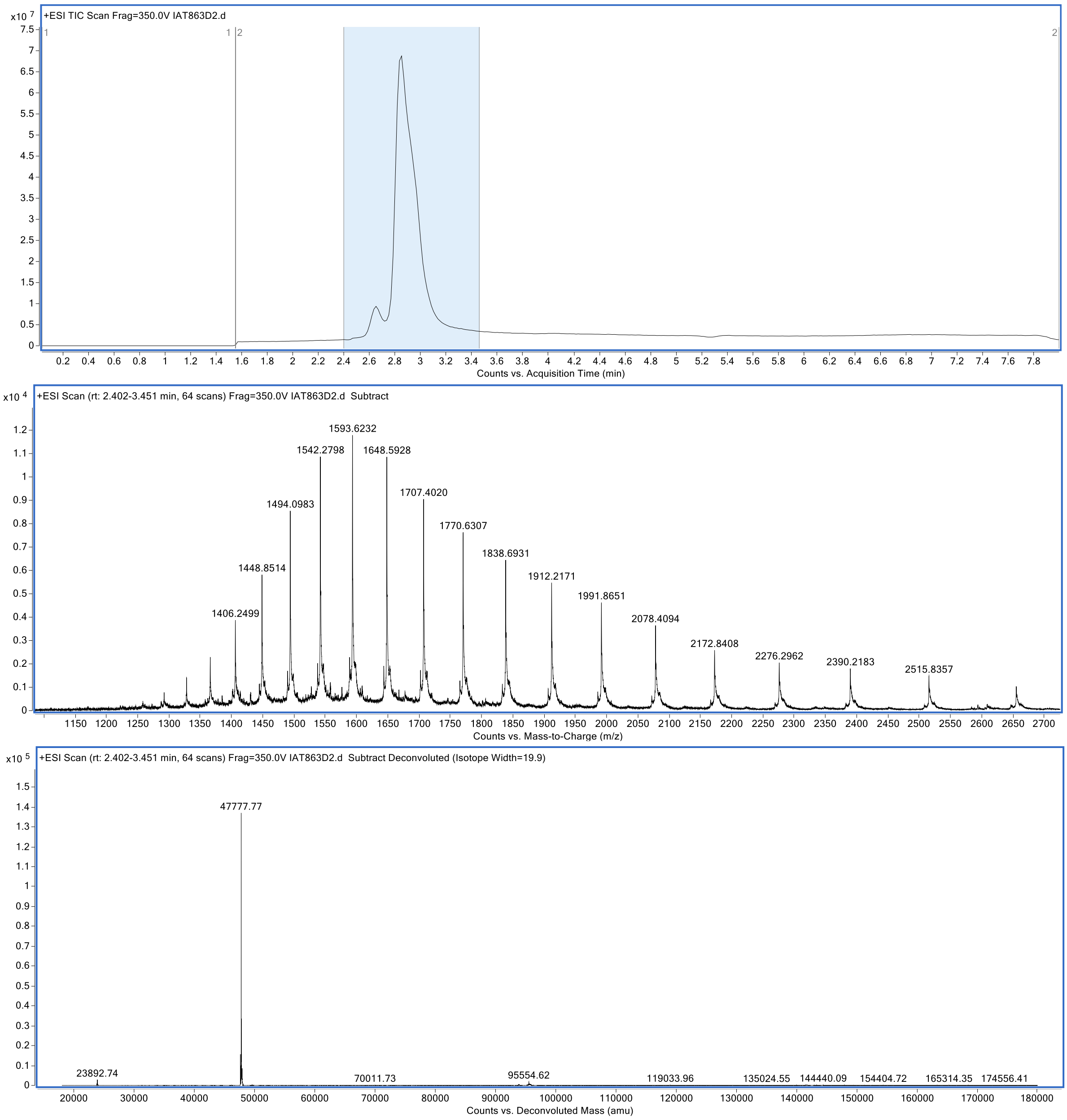

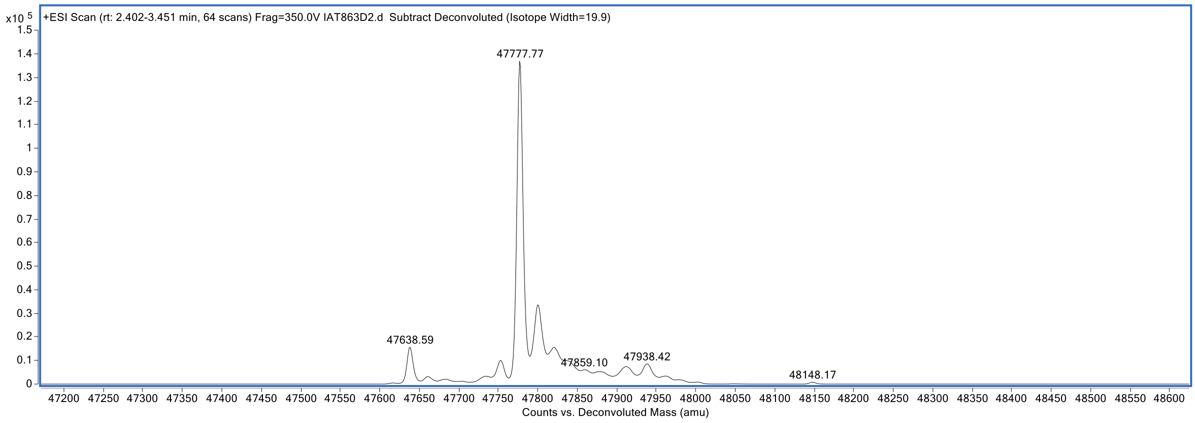

Figure S68: (i) TIC LC-MS trace (top), (ii) non-deconvoluted LC-MS trace (upper middle), (iii) deconvoluted MS data (lower middle, wide range), (iv) bottom (zoom in mass range) for restoration step.

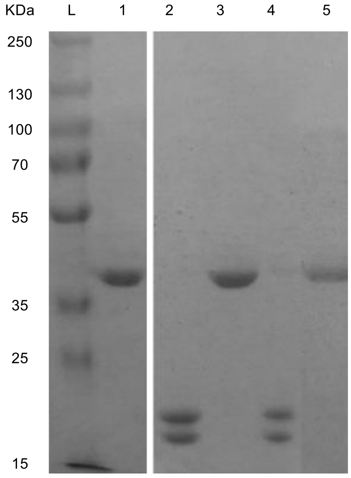

Figure S69: SDS-PAGE gel: L) Ladder, 1) Ontruzant Fab **1**, 2) Reduction step, 3) Conjugation/Lysine reaction step, 4) Deprotection step, 5) Disulfide restoration step.

2.6.3.1 Control reaction of Ontruzant Fab **1** (non-reduced) with reagent **5**

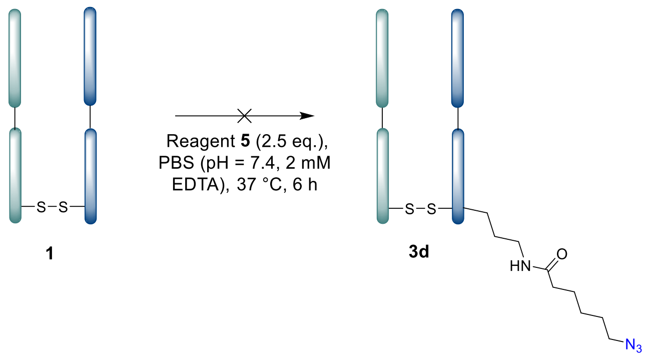

To a solution of Ontruzant Fab **1** (130 µL, 20 µM) in PBS (25 mM sodium borate, 25 mM NaCl, 2 mM EDTA, pH 7.4) was added reagent **5** (10 mM in MeCN, 2.5 equiv.) was added, and incubated at 37 °C for 6 h whilst shaking (300 rpm). After this time, the conjugate was purified into DI H_2O. The resulting conjugate was analysed by LC-MS.

Ontruzant Fab **1**: Expected mass: 47639.10 Da, observed mass: 47639.02 Da

Mono-labelled conjugate **3d**: Expected mass: 47779.27 Da, observed mass: 47778.03 Da

Double-labelled Fab conjugate: Expected mass: 47918.44 Da, observed mass: 47916.73 Da

Triple-labelled Fab conjugate: Expected mass: 48057.62 Da, observed mass: 48055.14 Da

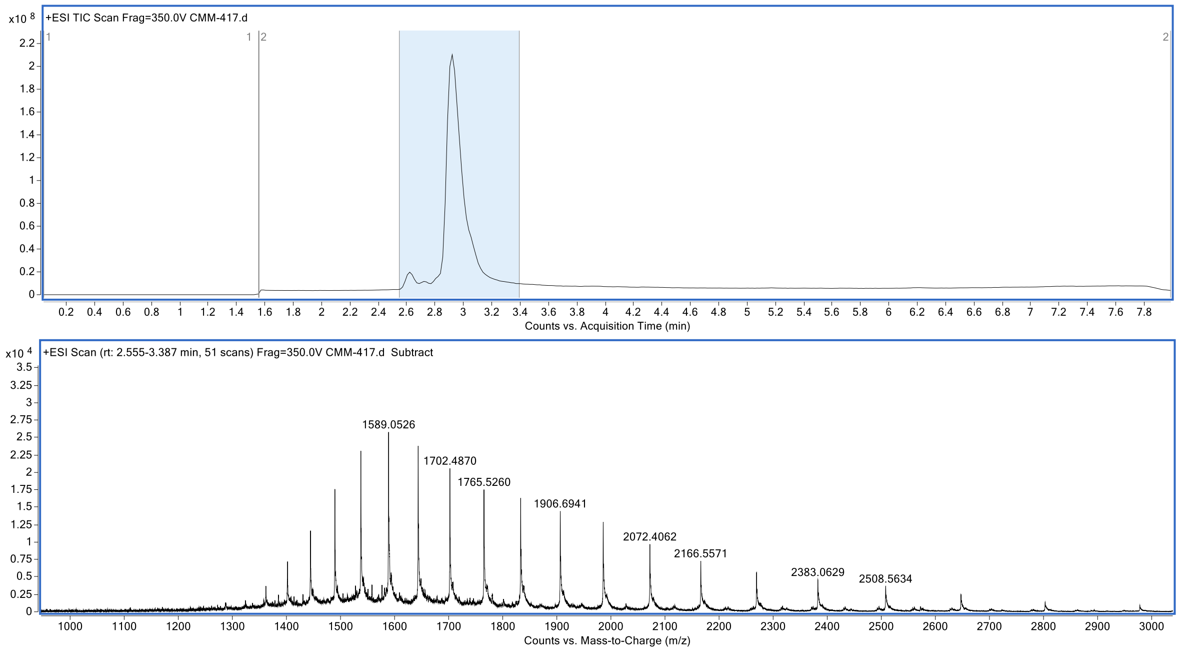

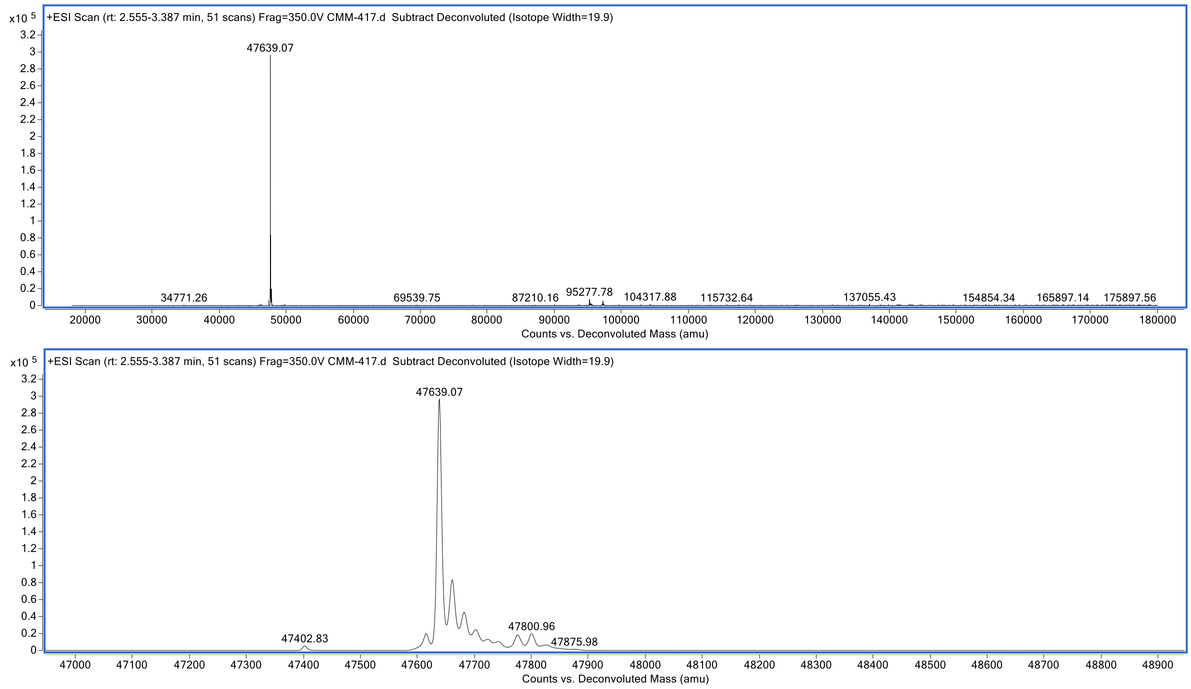

Figure S68a: (i) TIC LC-MS trace (top), (ii) non-deconvoluted LC-MS trace (upper middle), (iii) deconvoluted MS data (lower middle, wide range), (iv) bottom (zoom in mass range) for restoration step.

2.6.4 Reaction of Ontruzant Fab **1** with reagent **6**

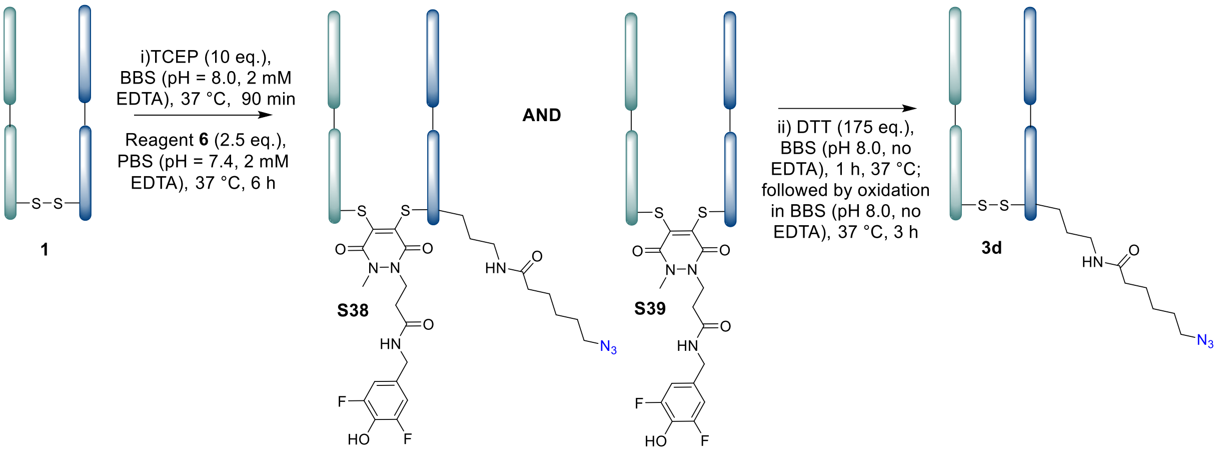

Results were obtained using general procedure 2.6.1., using PD reagent **6**.

a) Conjugation step (i)

Conjugate **S38** Expected mass: 48115.53 Da, observed mass: 48115.18 Da

Hydrolysed conjugate **S39**: Expected mass: 47976.37 Da, Observed mass: 47976.74 Da

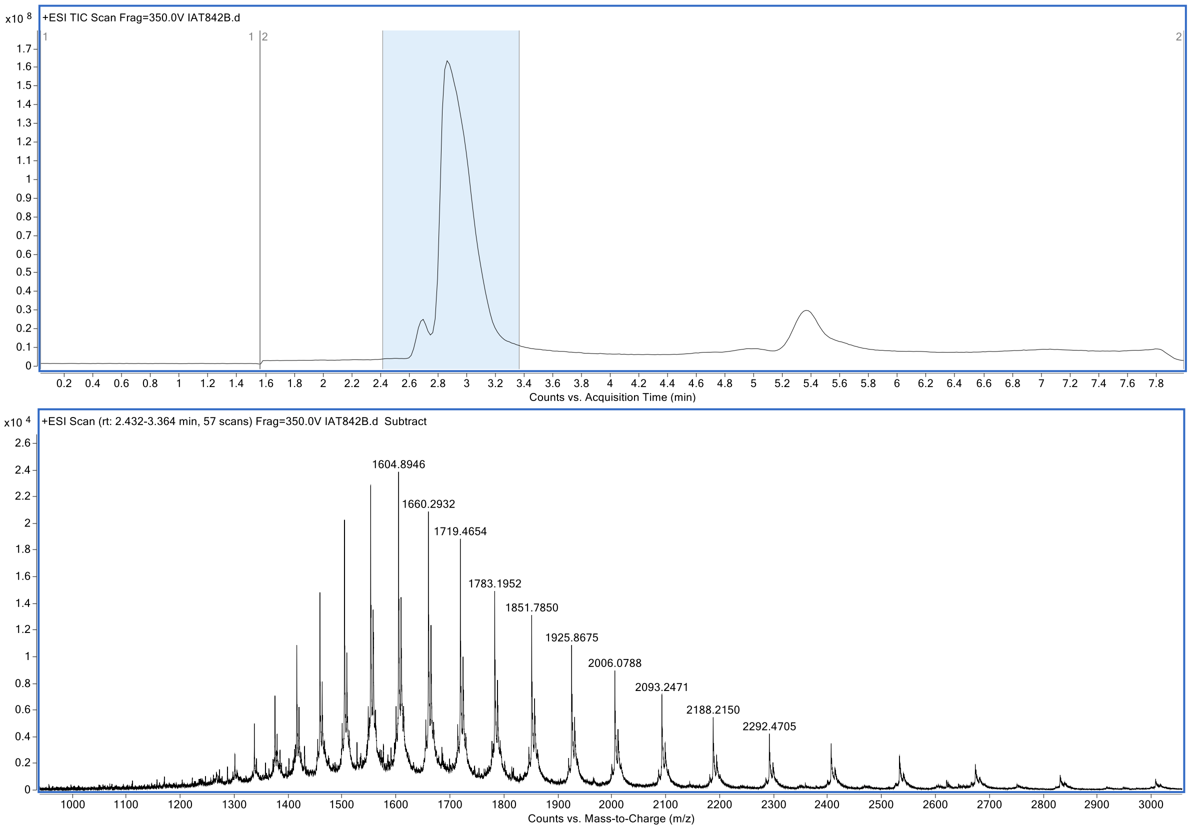

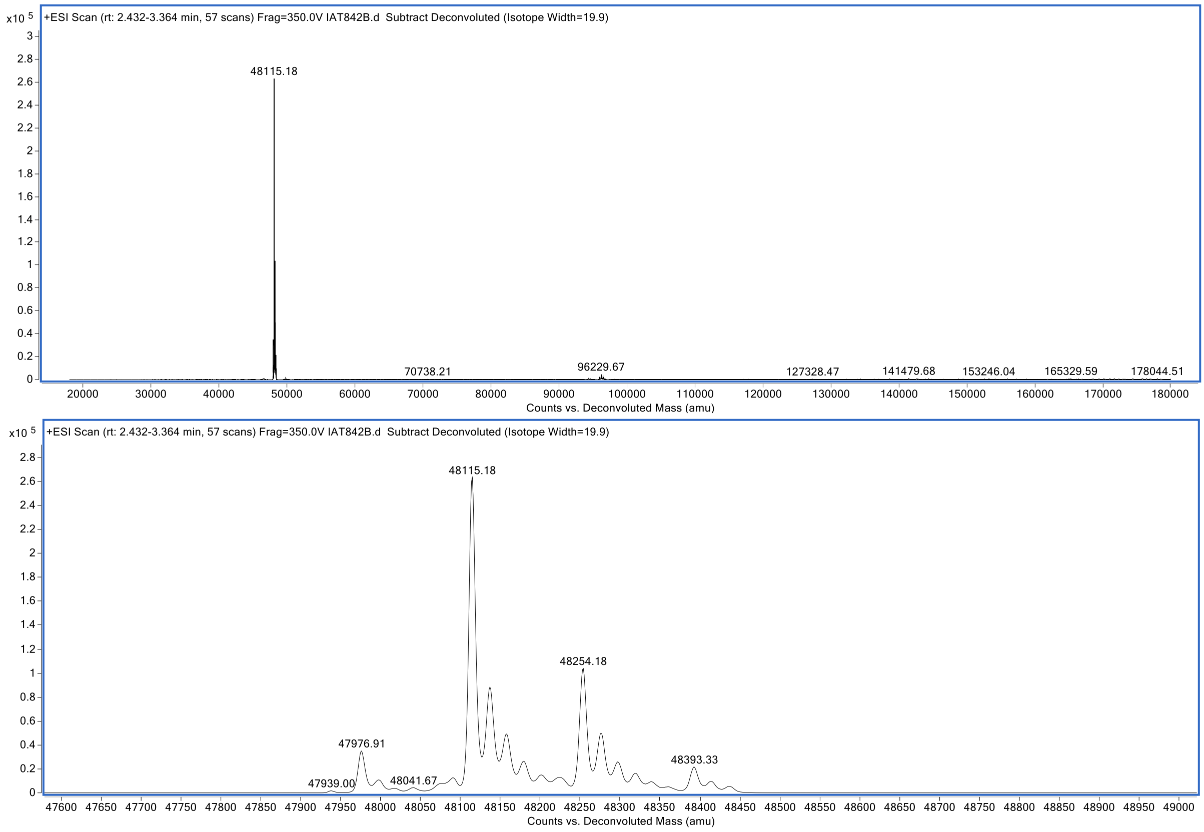

Figure S70: (i) TIC LC-MS trace (top), (ii) non-deconvoluted LC-MS trace (upper middle), (iii) deconvoluted MS data (lower middle, wide range), (iv) zoom in mass range (bottom) for conjugation step.

b) Restoration step

Mono-labelled conjugate **3d**: Expected mass: 47779.27 Da, observed mass: 47778.01 Da **(%Abundance: 60%)**

Ontruzant Fab **1**: Expected mass: 47639.10 Da, observed mass: 47639.33 Da **(%Abundance: 11%)**

Double-labelled Fab conjugate: Expected mass: 47918.44 Da, observed mass: 47916.40 Da

Triple-labelled Fab conjugate: Expected mass: 48057.62 Da, observed mass: 48054.53 Da

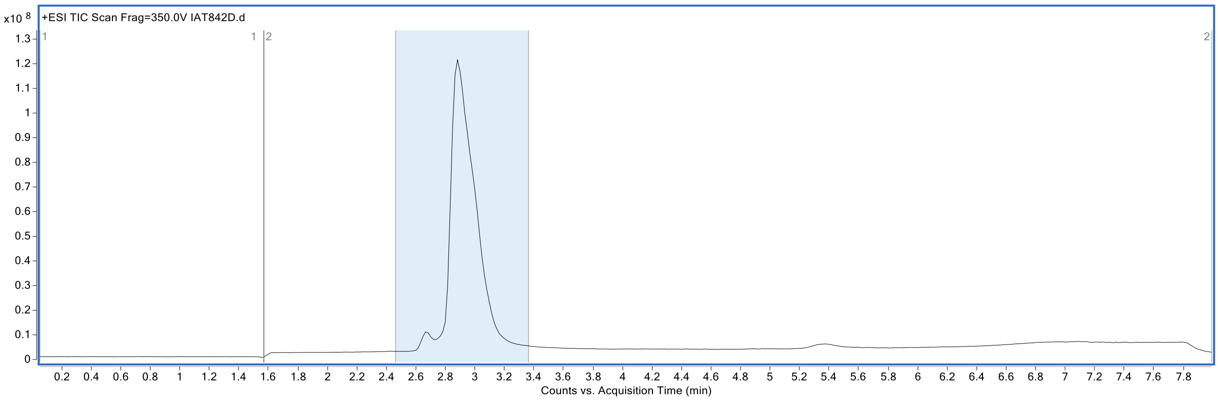

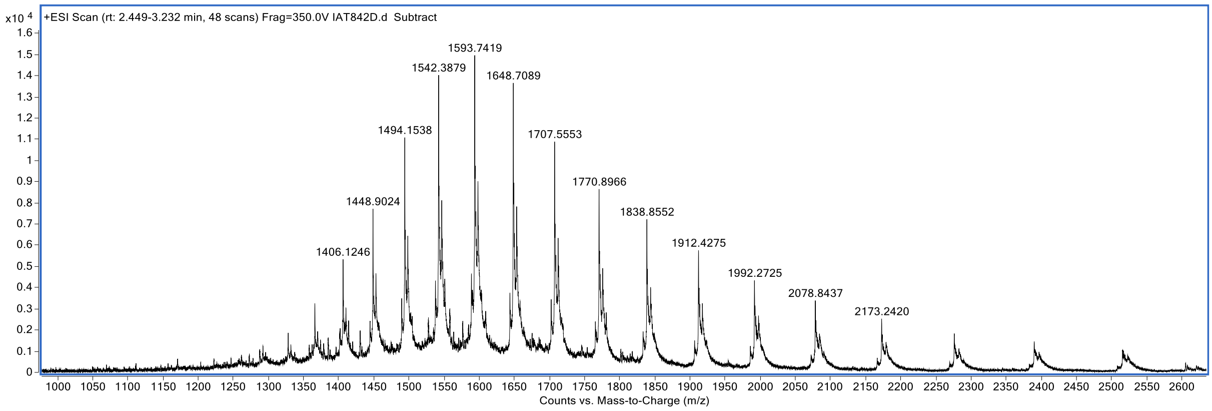

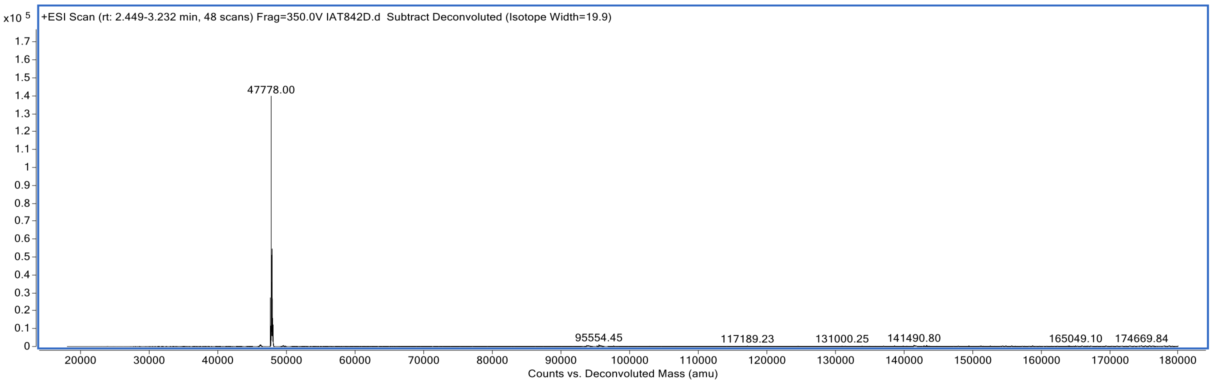

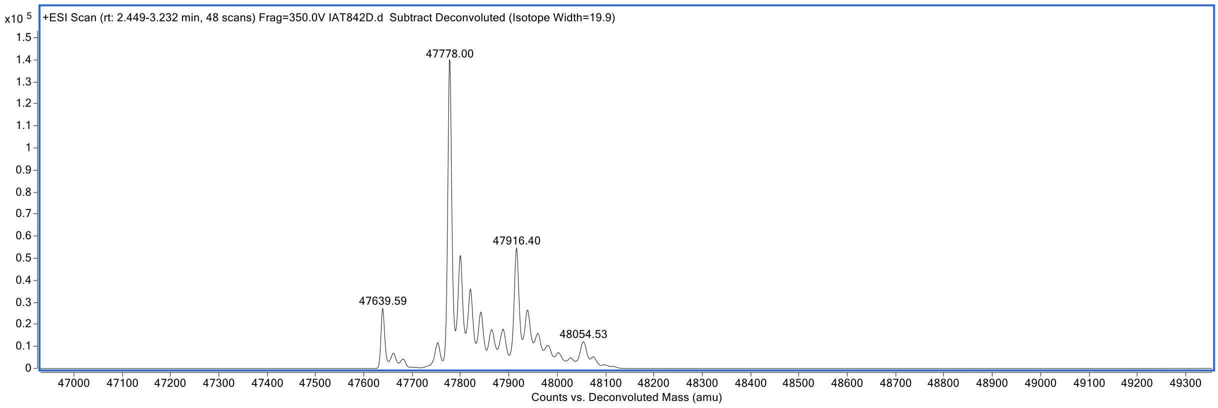

Figure S71: (i) TIC LC-MS trace (top), (ii) non-deconvoluted LC-MS trace (upper middle), (iii) deconvoluted MS data (lower middle, wide range), (iv) zoom in mass range (bottom) for restoration step.

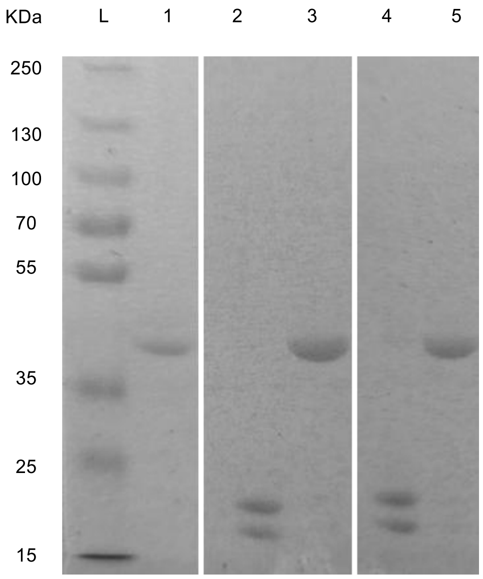

Figure S72: SDS-PAGE gel: L) Ladder, 1) Ontruzant Fab **1**, 2) Reduction step, 3) Conjugation/Lysine reaction step, 4) Deprotection step, 5) Disulfide restoration step.

2.6.4.1 Control reaction of Ontruzant Fab **1** (non-reduced) with reagent **6**

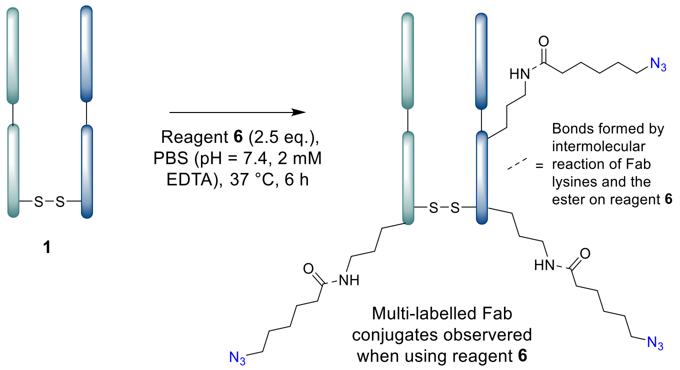

To a solution of Ontruzant Fab **1** (130 µL, 20 µM) in PBS (25 mM sodium borate, 25 mM NaCl, 2 mM EDTA, pH 7.4) was added reagent **6** (10 mM in MeCN, 2.5 equiv.) was added, and incubated at

37 °C for 6 h whilst shaking (300 rpm). After this time, the conjugate was purified into DI H_2O. The resulting conjugate was analysed by LC-MS.

Ontruzant Fab **1**: Expected mass: 47639.10 Da, observed mass: 47639.02 Da

Mono-labelled conjugate **3d**: Expected mass: 47779.27 Da, observed mass: 47778.03 Da

Double-labelled Fab conjugate: Expected mass: 47918.44 Da, observed mass: 47916.73 Da

Triple-labelled Fab conjugate: Expected mass: 48057.62 Da, observed mass: 48055.14 Da

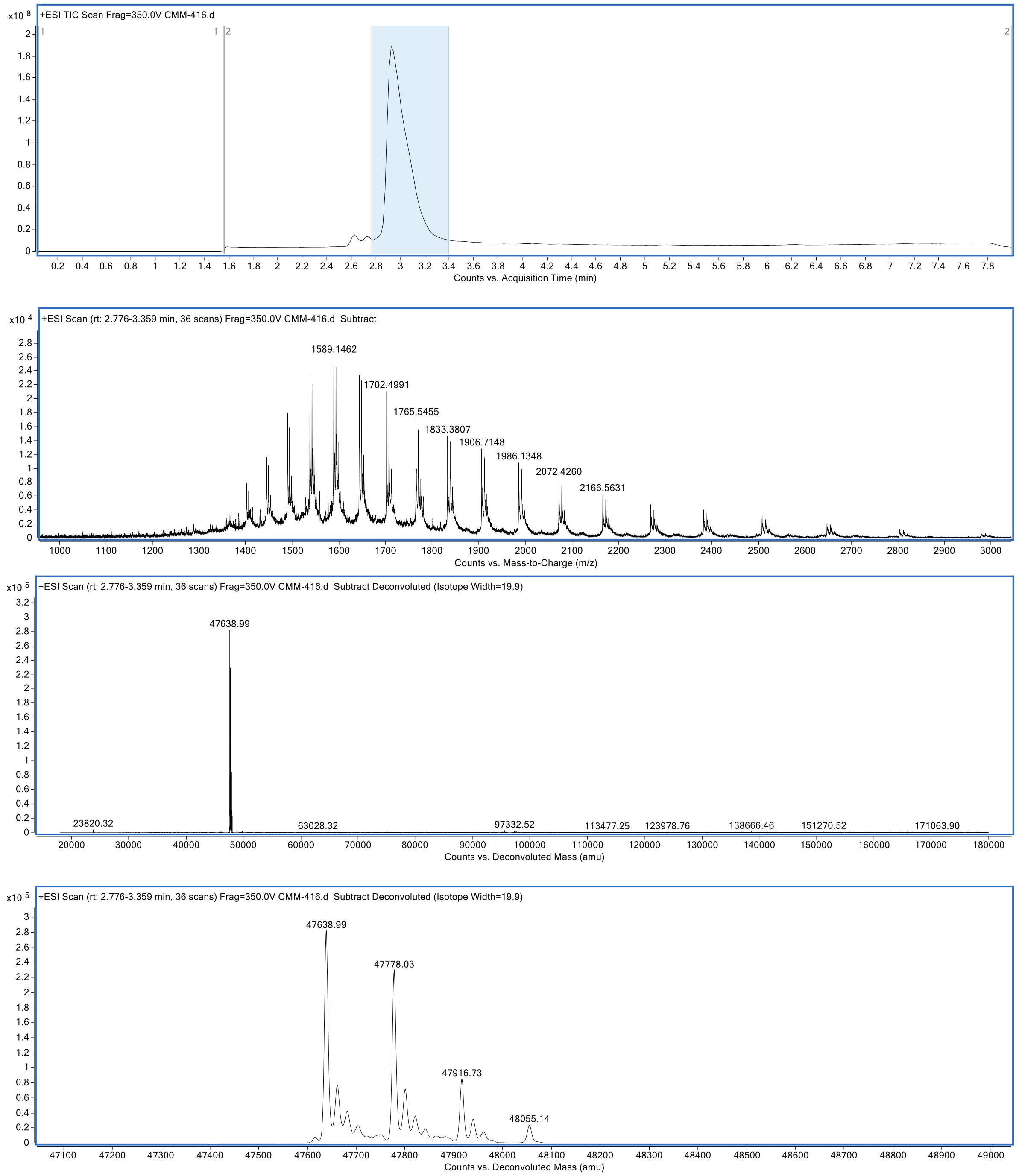

Figure S71a: (i) TIC LC-MS trace (top), (ii) non-deconvoluted LC-MS trace (upper middle), (iii) deconvoluted MS data (lower middle, wide range), (iv) bottom (zoom in mass range) for restoration step.

2.7 Bioconjugation studies of reagents on Ontruzant Fab **1** and subsequent functionilisation

2.7.1. General experimental procedure

To a solution of Ontruzant Fab **1** (130 µL, 20 µM) in BBS (25 mM sodium borate, 25 mM NaCl, 2 mM EDTA, pH 8.0) was added TCEP·HCl (20 mM in DI H_2O, 10 eq.). The reaction was incubated at 37 °C for 90 min whilst shaking (300 rpm). After this time, the conjugate was purified into PBS (50 mM phosphate, 150 mM NaCl, 2 mM EDTA, pH 7.4) by centrifugation (7 K Zeba Spin desalting column), a PD reagent (10 mM in MeCN, 2.5 eq.) was added, and the solution was incubated at 37 °C for 6 h with shaking (300 rpm). After this time, the conjugate was purified into BBS (25 mM sodium borate, 25 mM NaCl, no EDTA, pH 8.0), BCN-PEG2-Amine **9** (10 mM in DMSO, 10 eq.) was added, and reaction incubated at 37 °C for 16 h in the dark. After this time, the conjugate was purified into BBS (25 mM sodium borate, 25 mM NaCl, no EDTA, pH 8.0) by centrifugation (7 K Zeba Spin desalting column). DTT (50 mM in DI H_2O, 175 eq.) was added, and reaction was incubated at 37 °C for 1 h. Upon completion, the conjugate was purified into BBS (25 mM sodium borate, 25 mM NaCl, no EDTA, pH 8.0) at 37 °C for 3 h to allow disulfide restoration. After this time, the conjugate was purified into DI H_2O. The resulting conjugate was analysed by LC-MS.

2.7.2 Reaction of Ontruzant Fab **1** with reagent **4** and BCN-PEG2-amine **9**

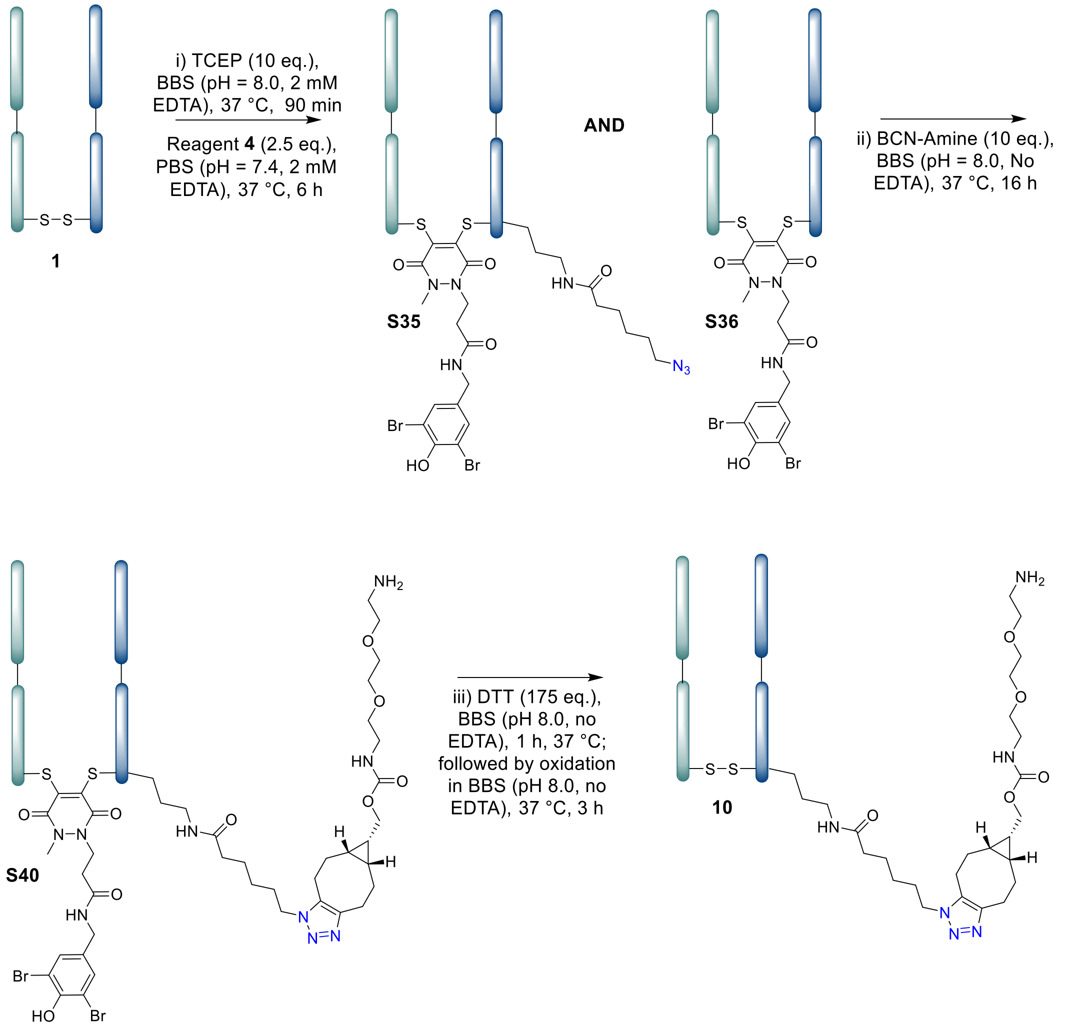

Results were obtained using general procedure 2.7.1, using PD reagent **4**.

a) Conjugation step (i)

Conjugate **S35**: 48237.34 Da, observed mass: 48237.12 Da

Hydrolysed conjugate **S36**: Expected mass: 48098.18 Da, Observed mass: 48099.01 Da

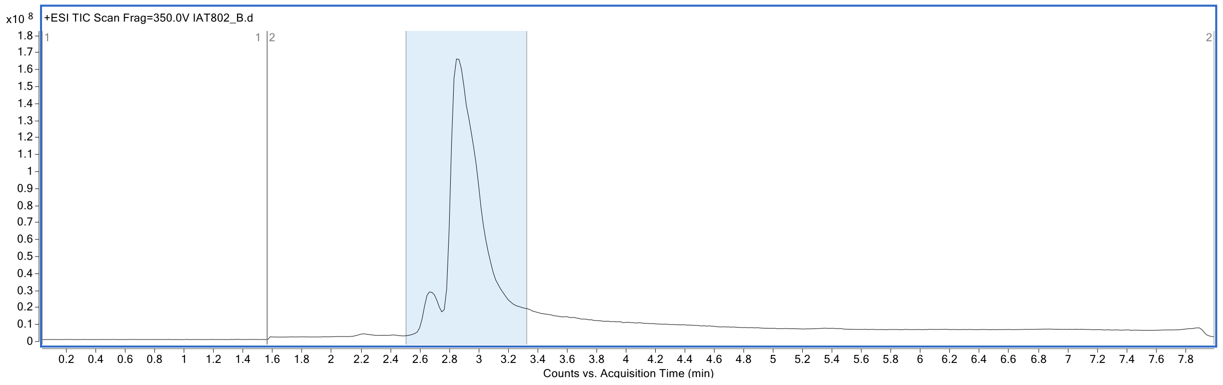

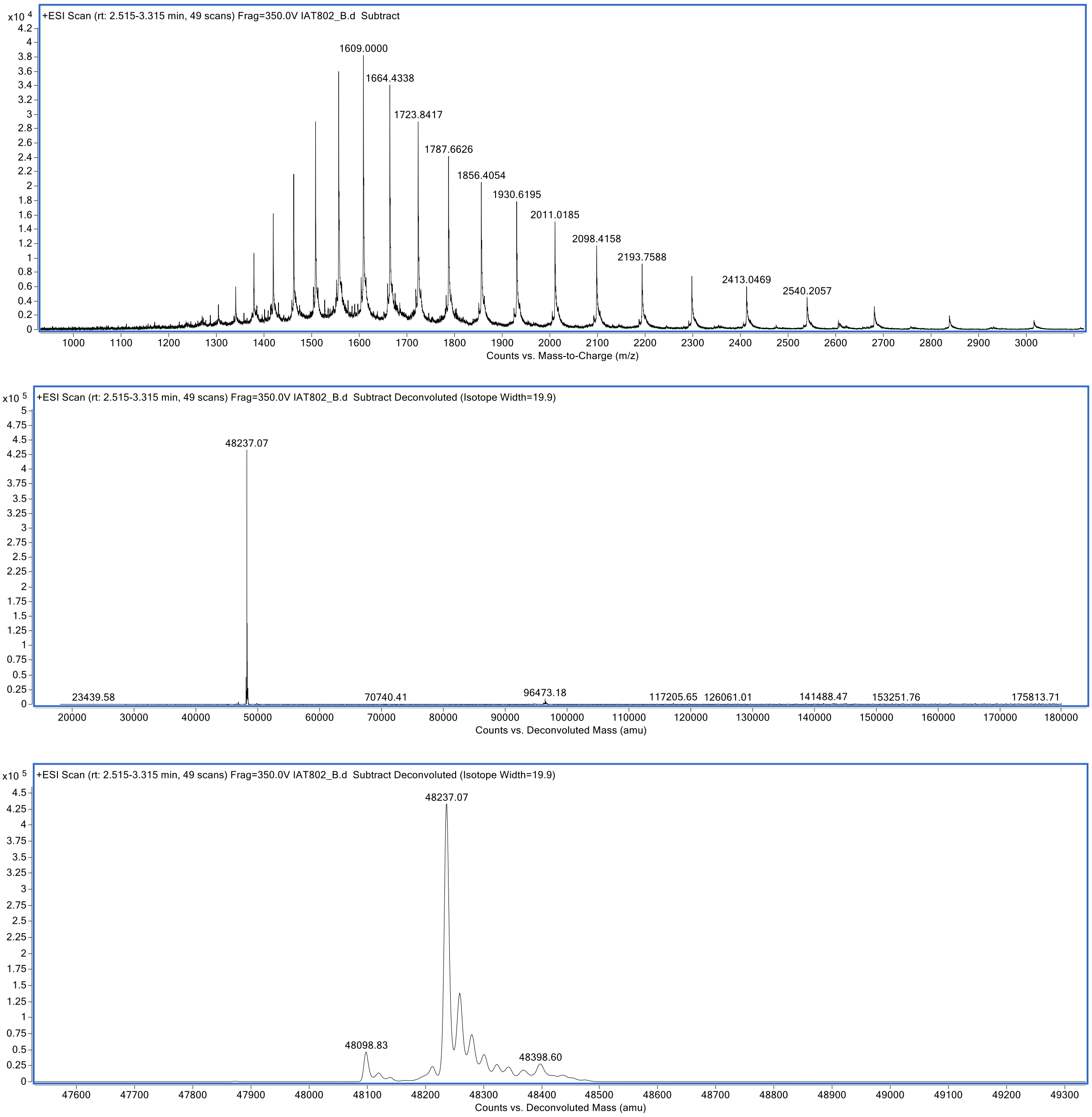

Figure S73: (i) TIC LC-MS trace (top), (ii) non-deconvoluted LC-MS trace (upper middle), (iii) deconvoluted MS data (lower middle, wide range), (iv) bottom (zoom in mass range) for conjugation step.

b) Click step (ii)

Conjugate **S40**: Expected mass: 48561.76 Da, observed mass: 48561.66 Da

Hydrolysed conjugate **S36**: Expected mass: 48098.18 Da, Observed mass: 48099.76 Da

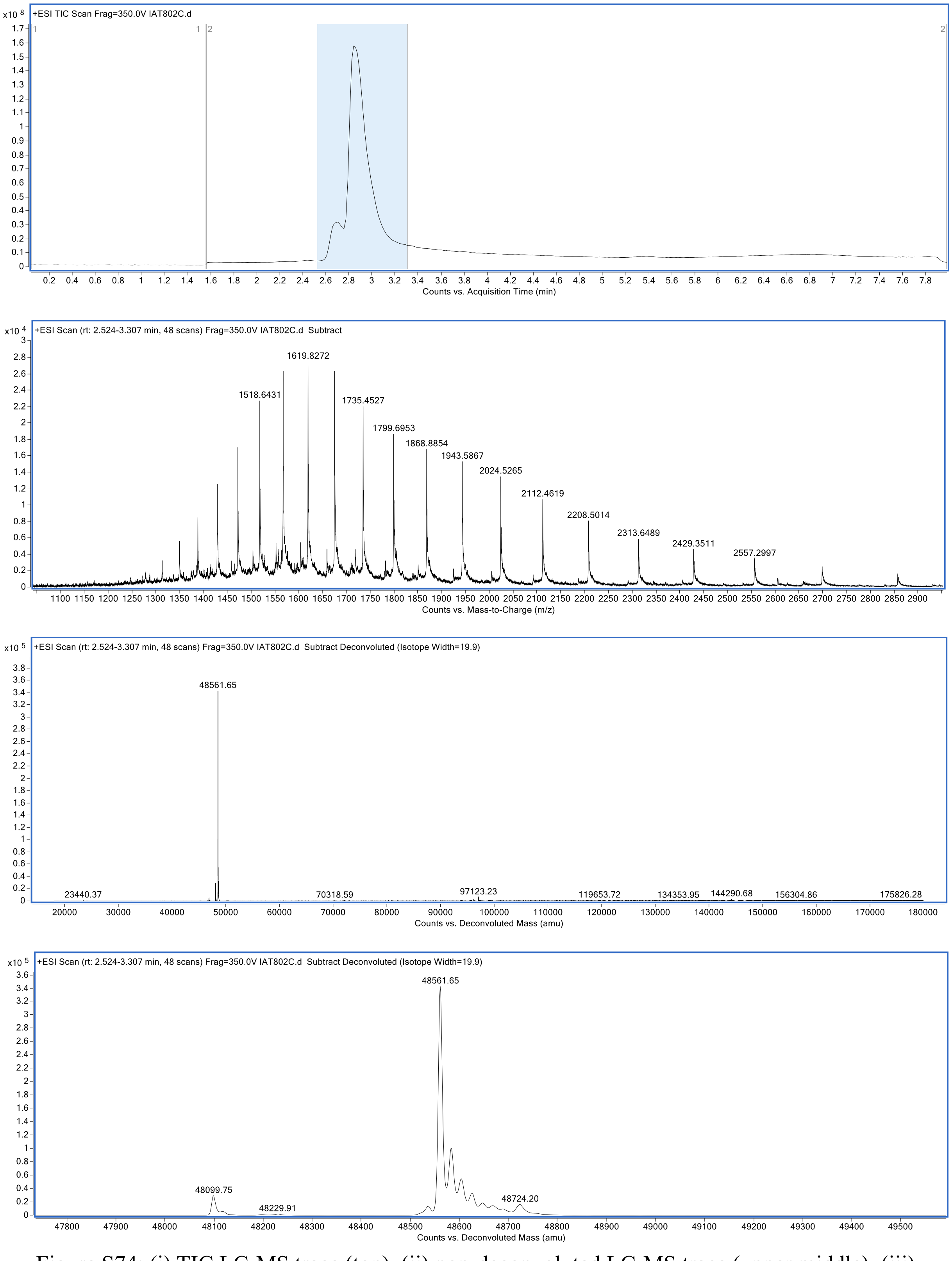

Figure S74: (i) TIC LC-MS trace (top), (ii) non-deconvoluted LC-MS trace (upper middle), (iii) deconvoluted MS data (lower middle, wide range), (iv) bottom (zoom in mass range) for click step.

c) Restoration step

Mono-labelled conjugate **10**: Expected mass: 48102.06 Da, observed mass: 48102.68 Da **(%Abundance: 93%)**

Ontruzant Fab **1**: Expected mass: 47639.10 Da, observed mass: 47639.50 Da **(%Abundance: 7%)**

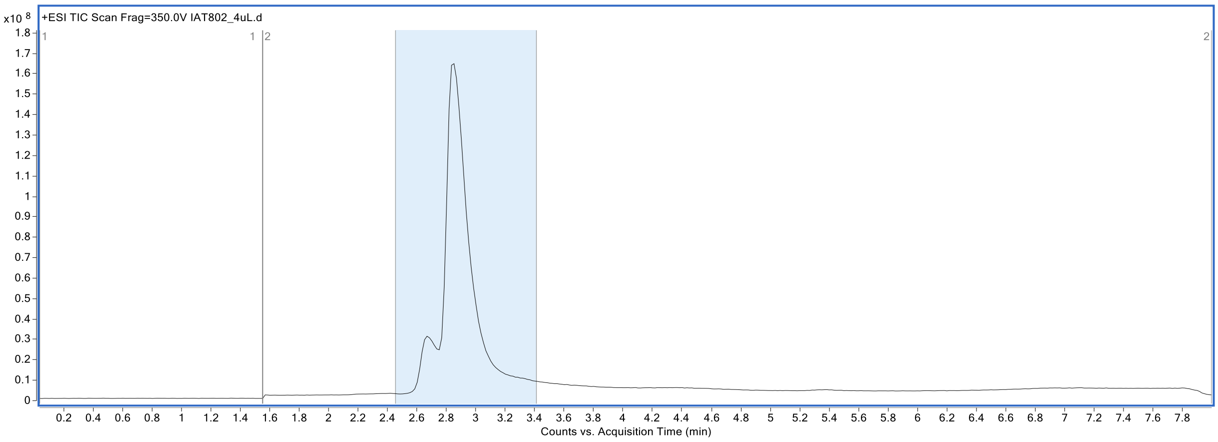

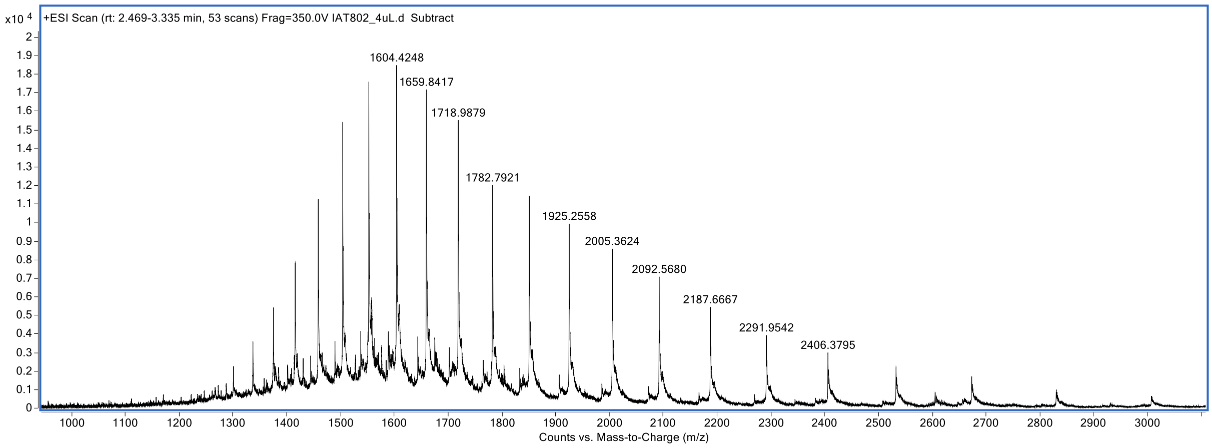

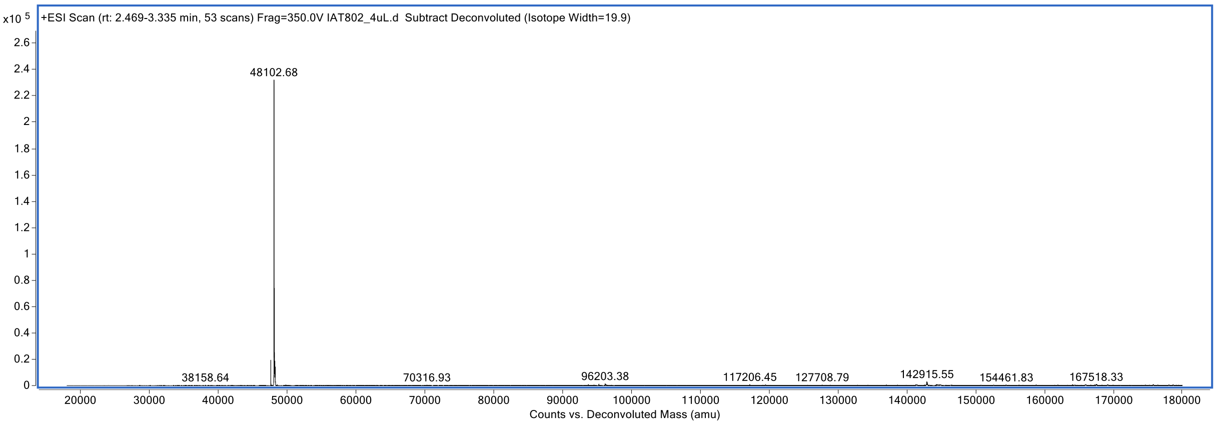

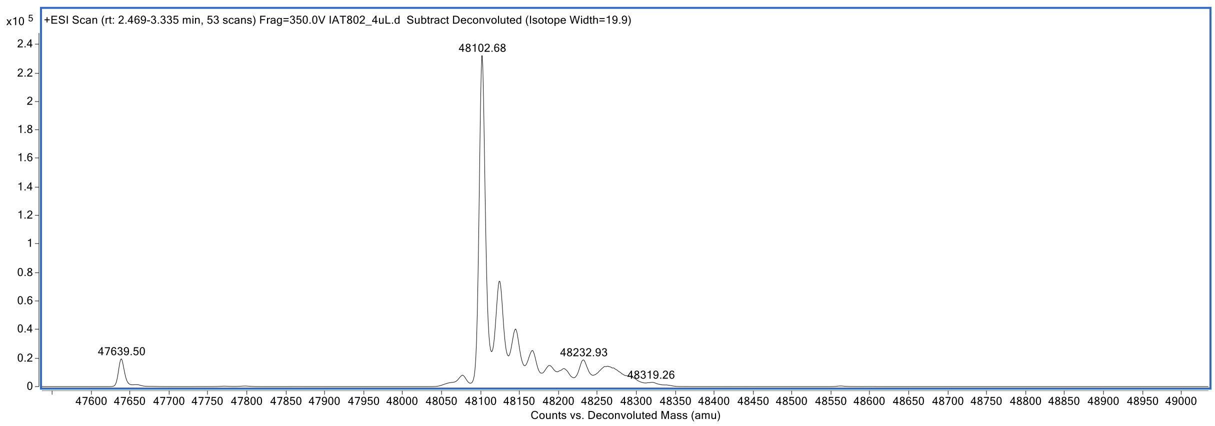

Figure S75: (i) TIC LC-MS trace (top), (ii) non-deconvoluted LC-MS trace (upper middle), (iii) deconvoluted MS data (lower middle, wide range), (iv) bottom (zoom in mass range) for restoration step.

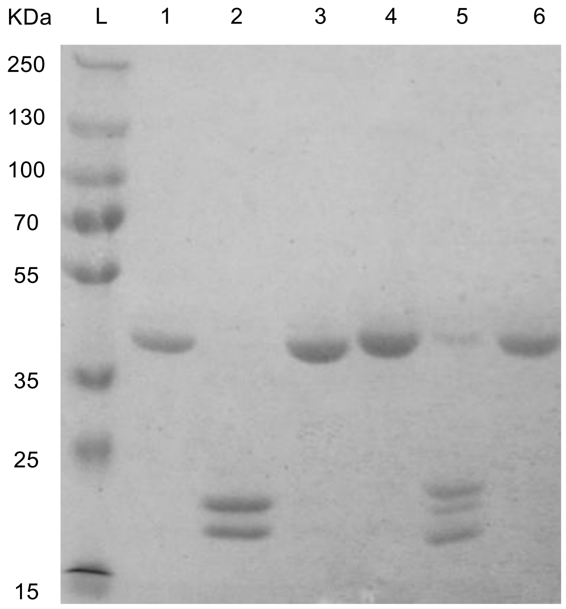

Figure S76: SDS-PAGE gel: L) Ladder, 1) Ontruzant Fab **1**, 2) Reduction step, 3) Conjugation/Lysine reaction step, 4) Click reaction step, 5) Deprotection step, 6) Disulfide restoration step.

2.7.3 Reaction of Ontruzant Fab **1** with reagent **7** and BCN-PEG2-amine **9**

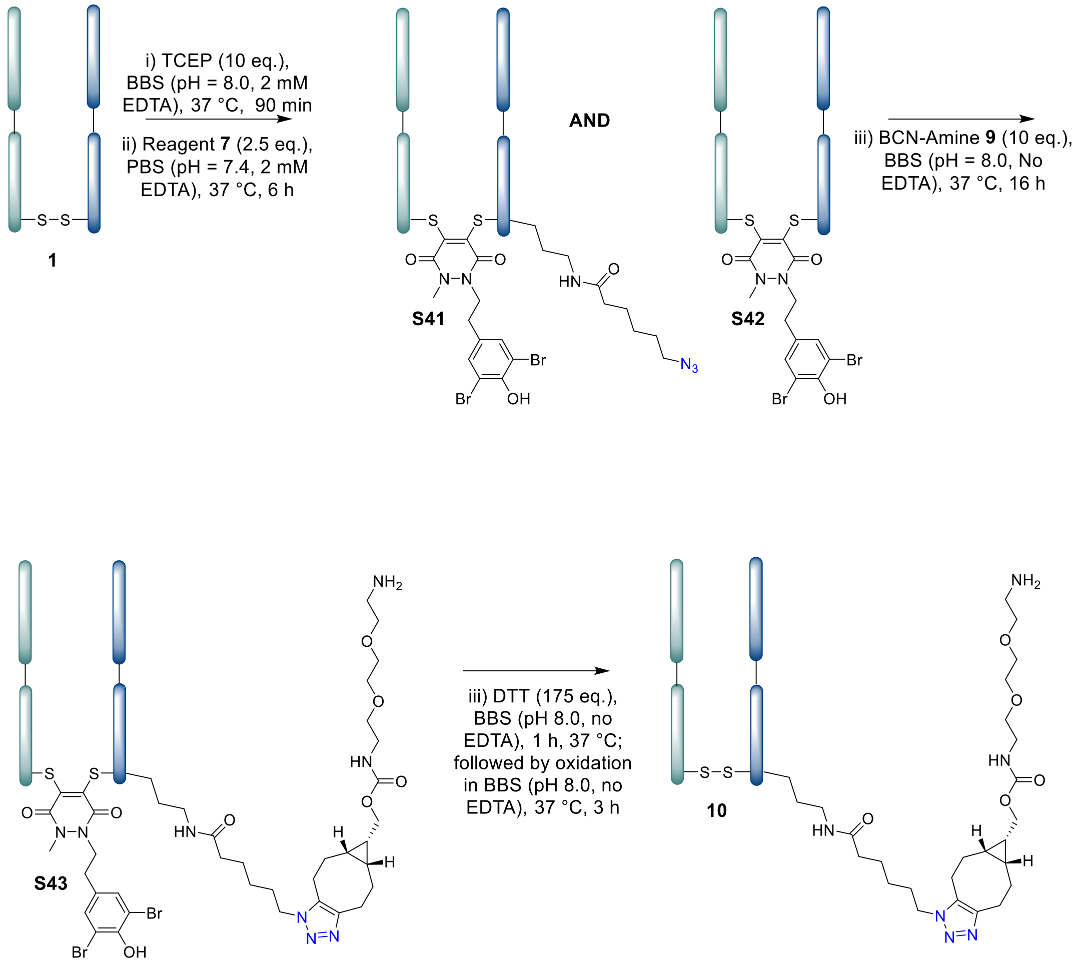

Results were obtained using general procedure 2.7.1, using PD reagent **7**.

a) Conjugation step (ii)

Conjugate **S41**: Expected mass: 48178.29 Da, observed mass: 48179.87 Da

Hydrolysed conjugate **S42**: Expected mass: 48041.13 Da, Observed mass: 48041.41 Da

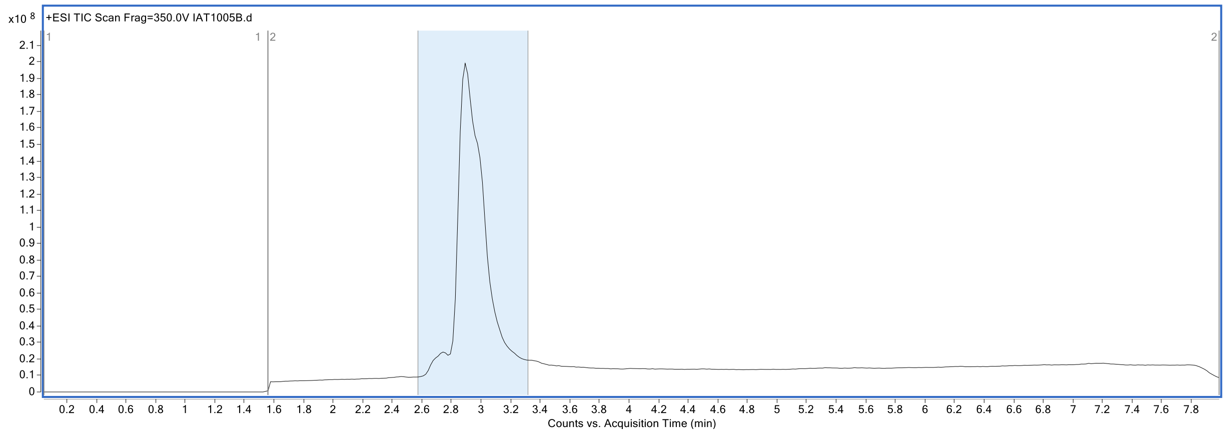

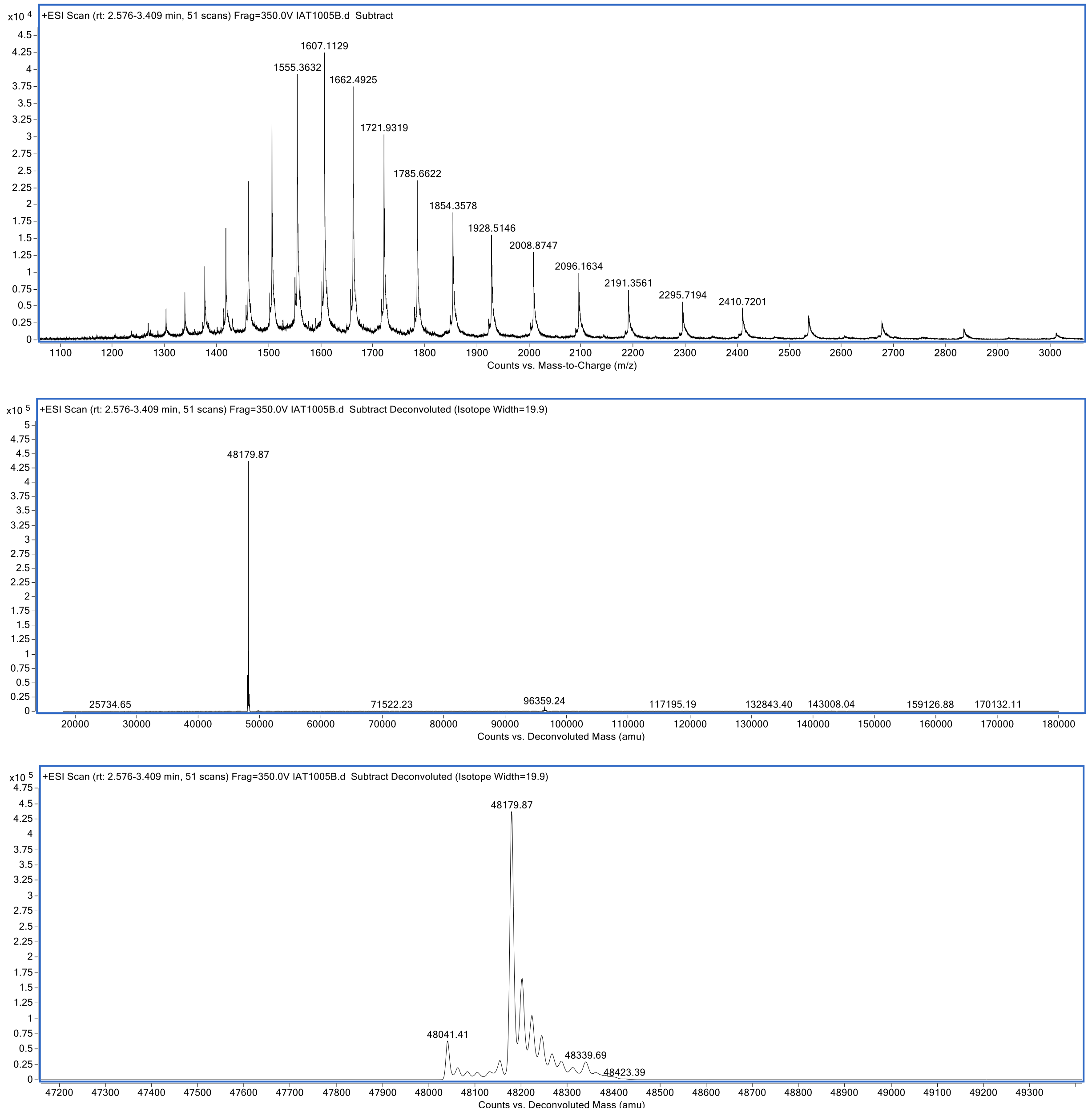

Figure S77: (i) TIC LC-MS trace (top), (ii) non-deconvoluted LC-MS trace (upper middle), (iii) deconvoluted MS data (lower middle, wide range), (iv) bottom (zoom in mass range) for conjugation step.

b) Click step (iii)

Conjugate **S43**: Expected mass: 48503.71 Da, observed mass: 48504.22 Da

Hydrolysed conjugate **S42**: Expected mass: 48041.13 Da, Observed mass: 48042.03 Da

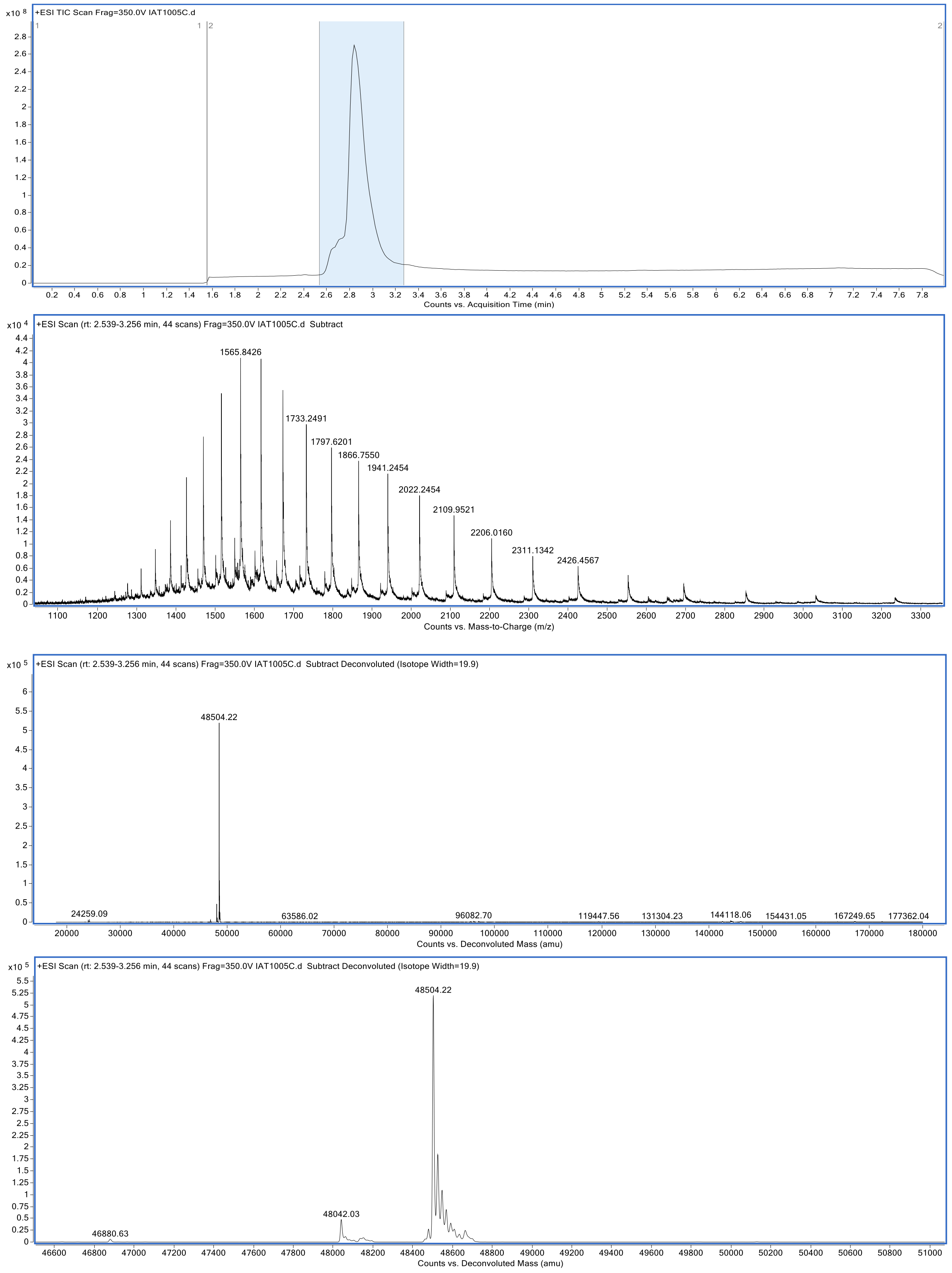

Figure S78: (i) TIC LC-MS trace (top), (ii) non-deconvoluted LC-MS trace (upper middle), (iii) deconvoluted MS data (lower middle, wide range), (iv) bottom (zoom in mass range) for click step.

c) Restoration step

Mono-labelled conjugate **10**: Expected mass: 48102.06 Da, observed mass: 48102.20 Da **(%Abundance: 90%)**

Ontruzant Fab **1**: Expected mass: 47639.10 Da, observed mass: 47639.33 Da **(%Abundance: 10%)**

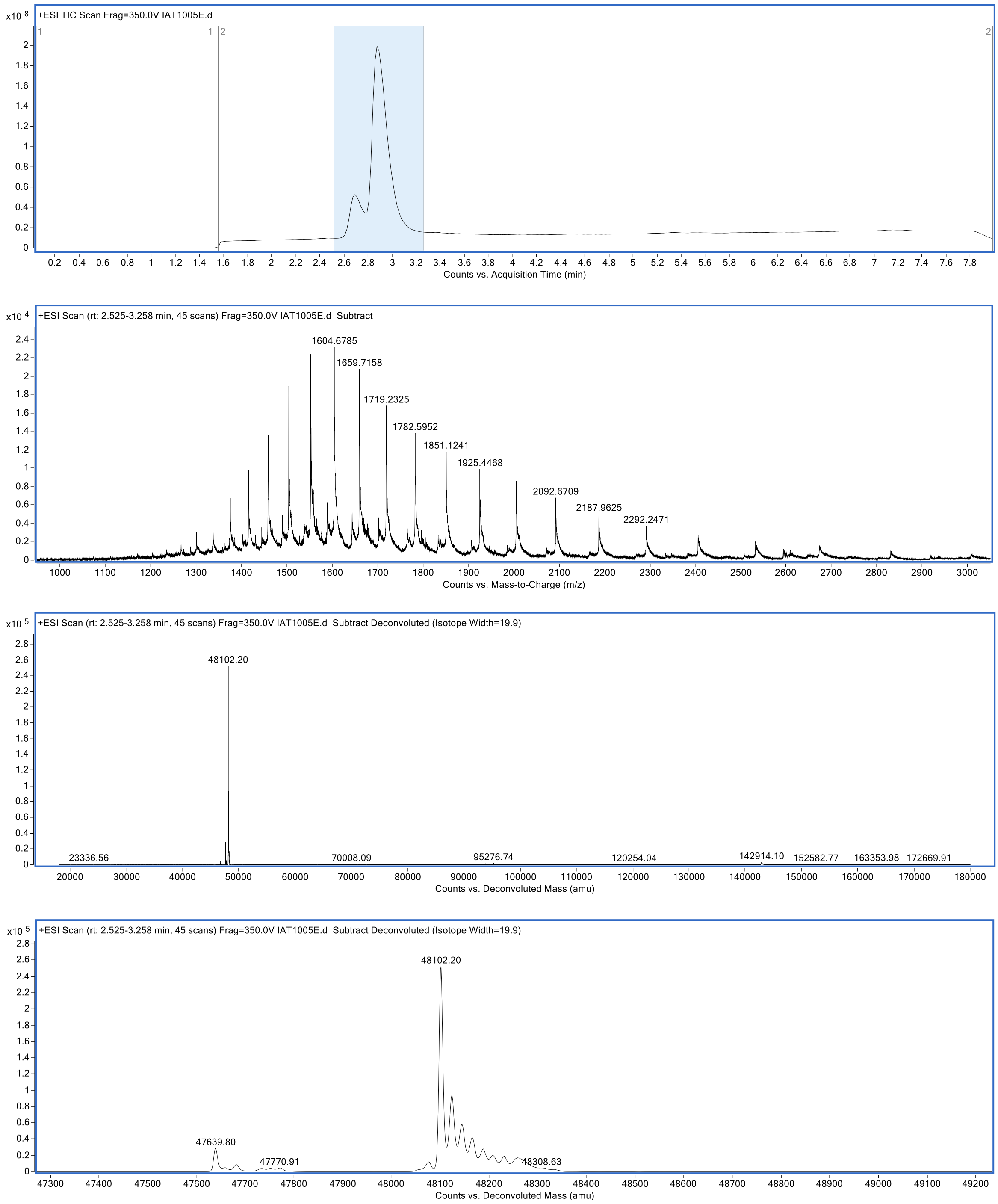

Figure S79: (i) TIC LC-MS trace (top), (ii) non-deconvoluted LC-MS trace (upper middle), (iii) deconvoluted MS data (lower middle, wide range), (iv) bottom (zoom in mass range) for restoration step.

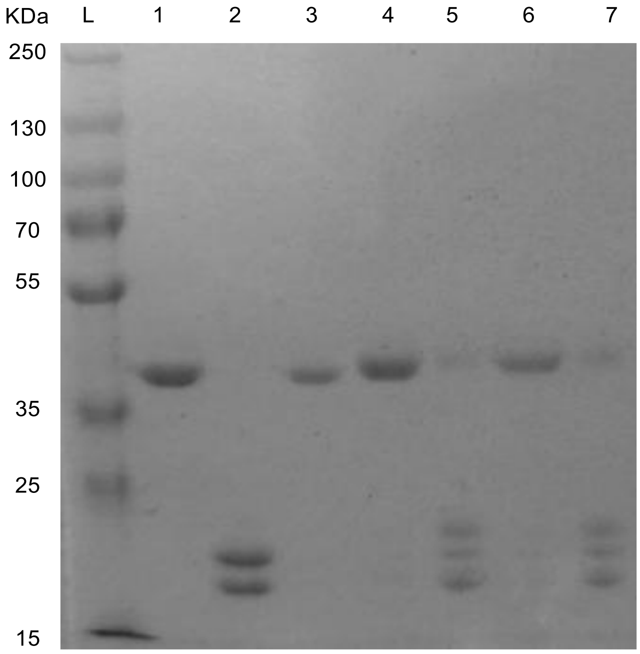

Figure S80: SDS-PAGE gel: L) Ladder, 1) Ontruzant Fab **1**, 2) Reduction step, 3) Conjugation/Lysine reaction step, 4) Click reaction step, 5) Deprotection step, 6) Disulfide restoration step.

2.7.4 Reaction of Ontruzant Fab **1** with reagent **8** and BCN-PEG2-amine **9**

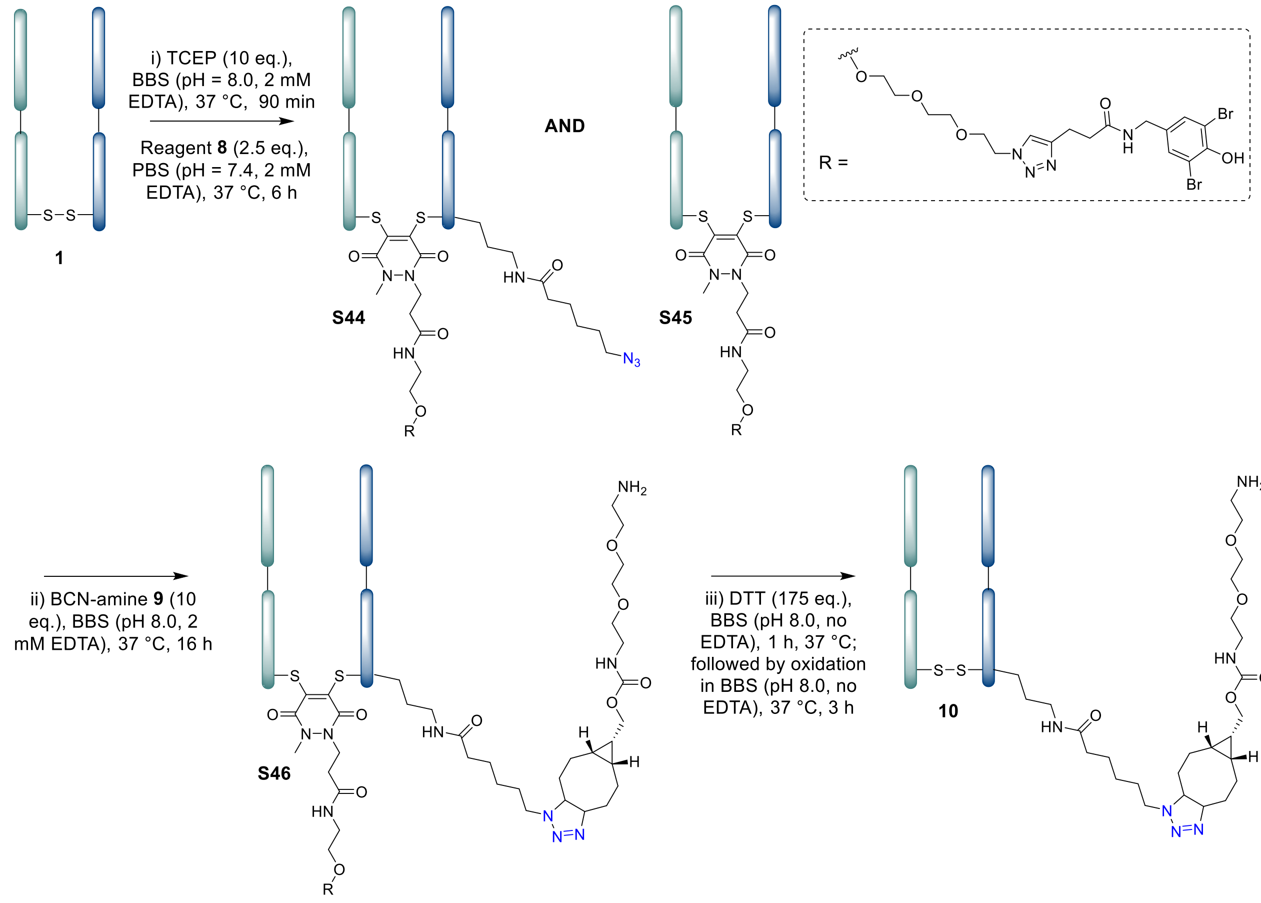

Results were obtained using general procedure 2.7.1., using PD reagent **8**.

a) Conjugation step (i)

Conjugate **S44**: Expected mass: 48535.68 Da, observed mass: 48535.07 Da

Hydrolysed conjugate **S45**: Expected mass: 48396.53 Da, Observed mass: 48396.42 Da

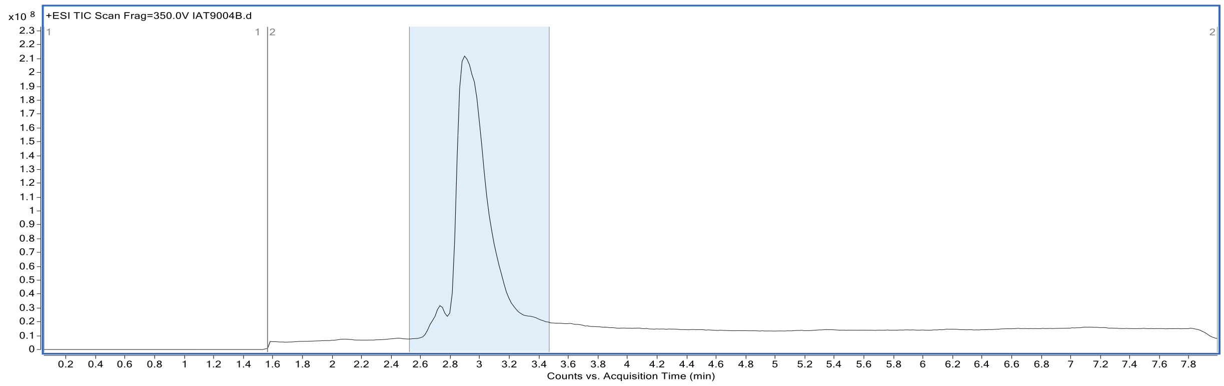

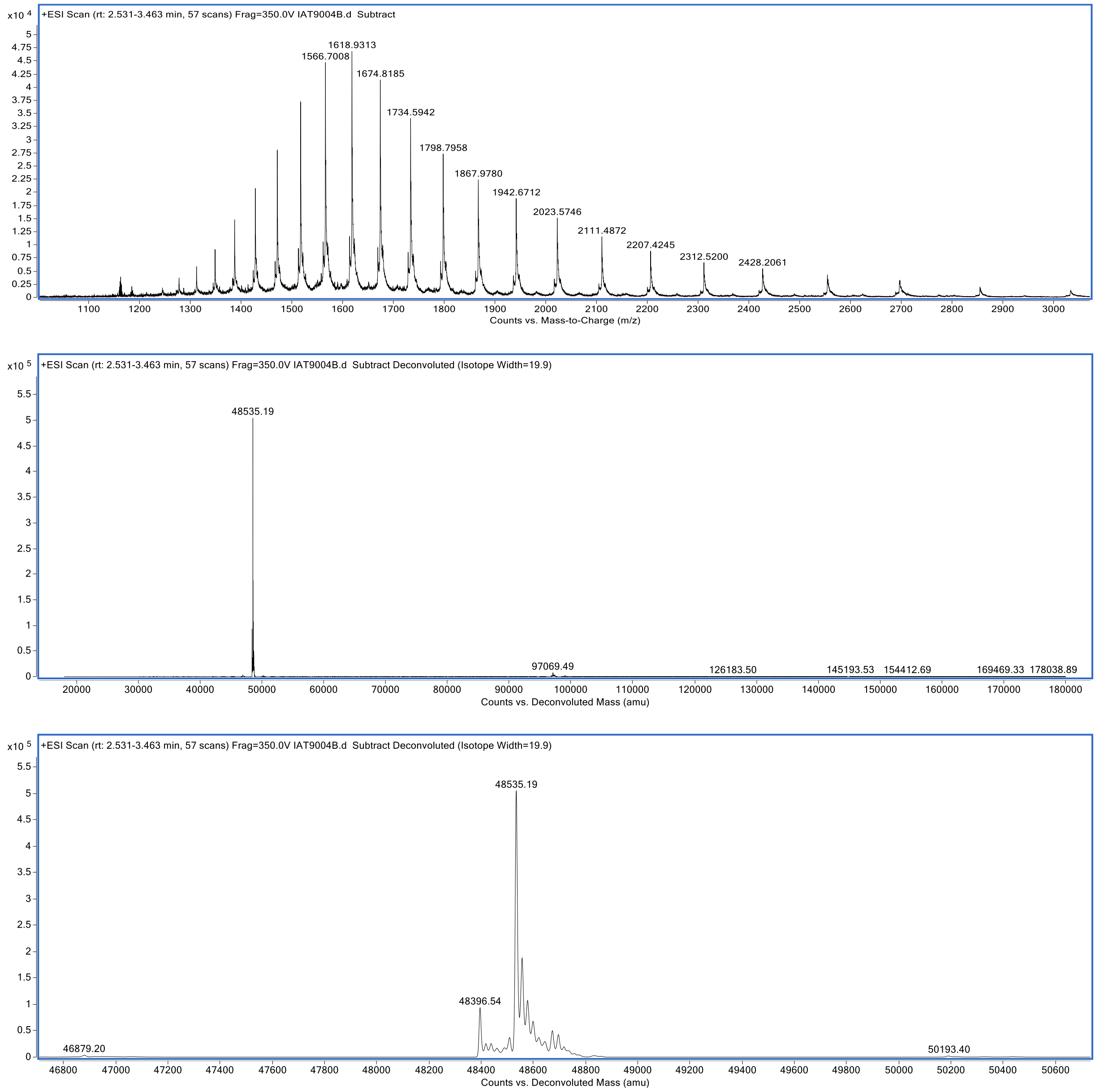

Figure S81: (i) TIC LC-MS trace (top), (ii) non-deconvoluted LC-MS trace (upper middle), (iii) deconvoluted MS data (lower middle, wide range), (iv) bottom (zoom in mass range) for conjugation step.

b) Click step (ii)

Conjugate **S46**: Expected mass: 48860.1Da, observed mass: 48859.86 Da

Hydrolysed conjugate **S45**: Expected mass: 48396.53 Da, Observed mass: 48396.42 Da

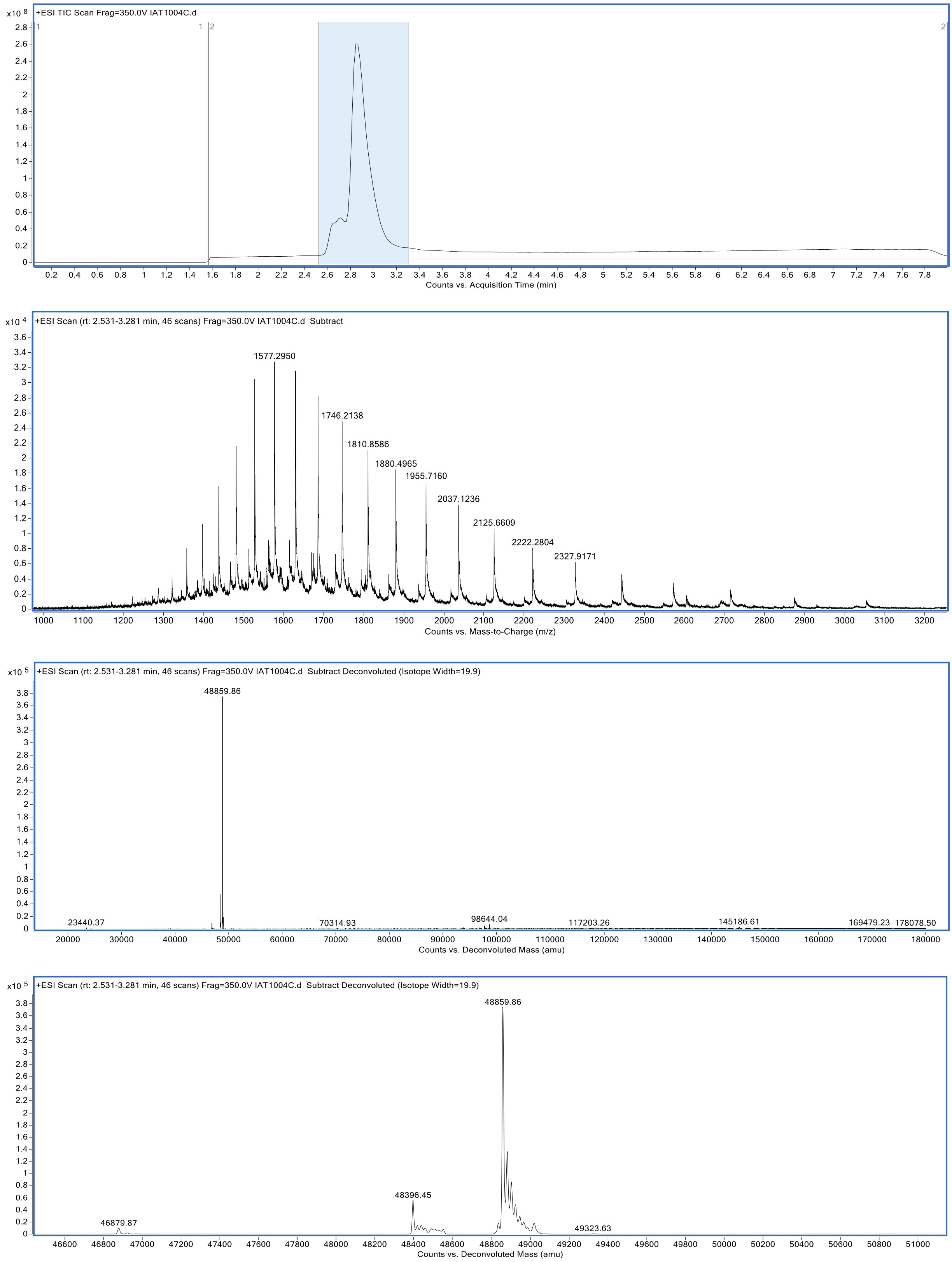

Figure S82: (i) TIC LC-MS trace (top), (ii) non-deconvoluted LC-MS trace (upper middle), (iii) deconvoluted MS data (lower middle, wide range), (iv) bottom (zoom in mass range) for click step.

c) Restoration step

Mono-labelled conjugate **10**: Expected mass: 48103.69 Da, observed mass: 48102.24 Da **(%Abundance: 90%)**

Ontruzant Fab **1**: Expected mass: 47639.10 Da, observed mass: 47639.33 Da **(%Abundance: 10%)**

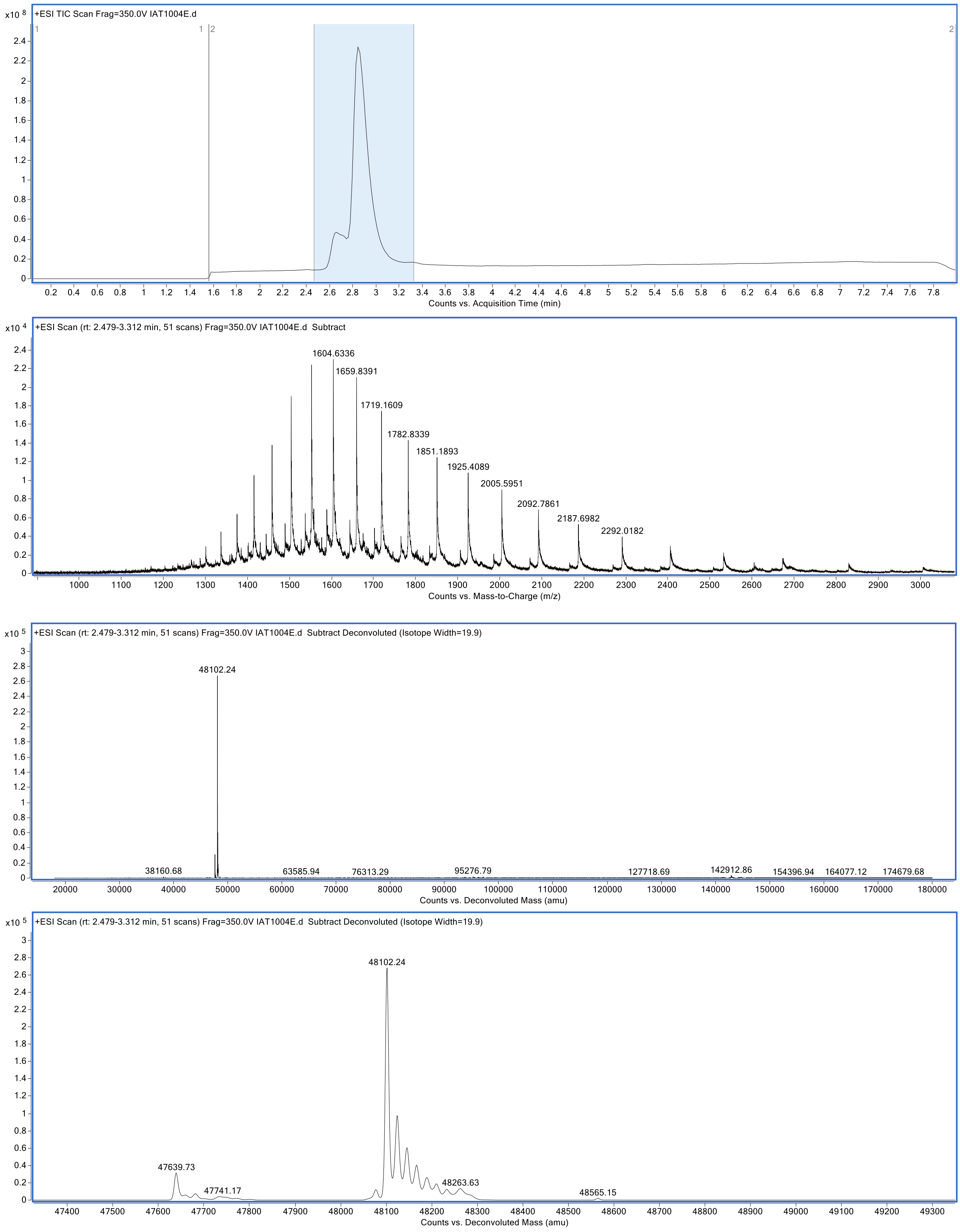

Figure S83: (i) TIC LC-MS trace (top), (ii) non-deconvoluted LC-MS trace (upper middle), (iii) deconvoluted MS data (lower middle, wide range), (iv) bottom (zoom in mass range) for disulfide restoration step.

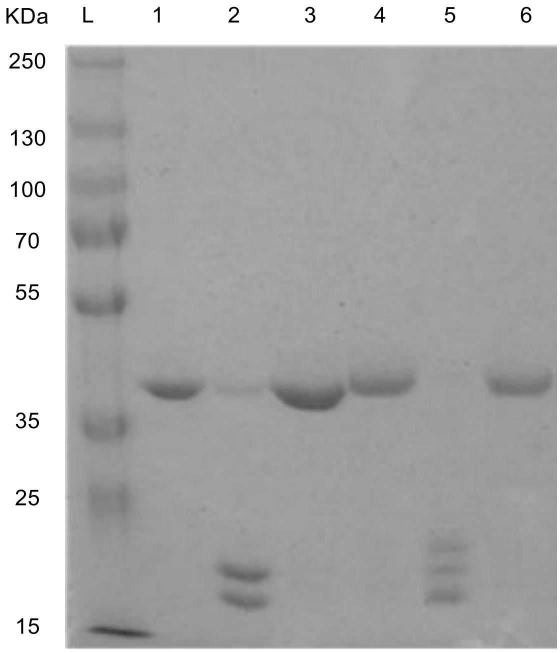

Figure S84: SDS-PAGE gel: L) Ladder, 1) Ontruzant Fab **1**, 2) Reduction step, 3) Conjugation/Lysine reaction step, 4) Click reaction step, 5) Deprotection step, 6) Disulfide restoration step.

2.8 Appraisal of the proposed strategy on other antibody Fab fragments of therapeutic relevance such as Fab_{CD20} and Fab_{CD3}

2.8.1 Reaction of Fab_{CD20} **S47** with reagent **4** and BCN-PEG2-amine **9**

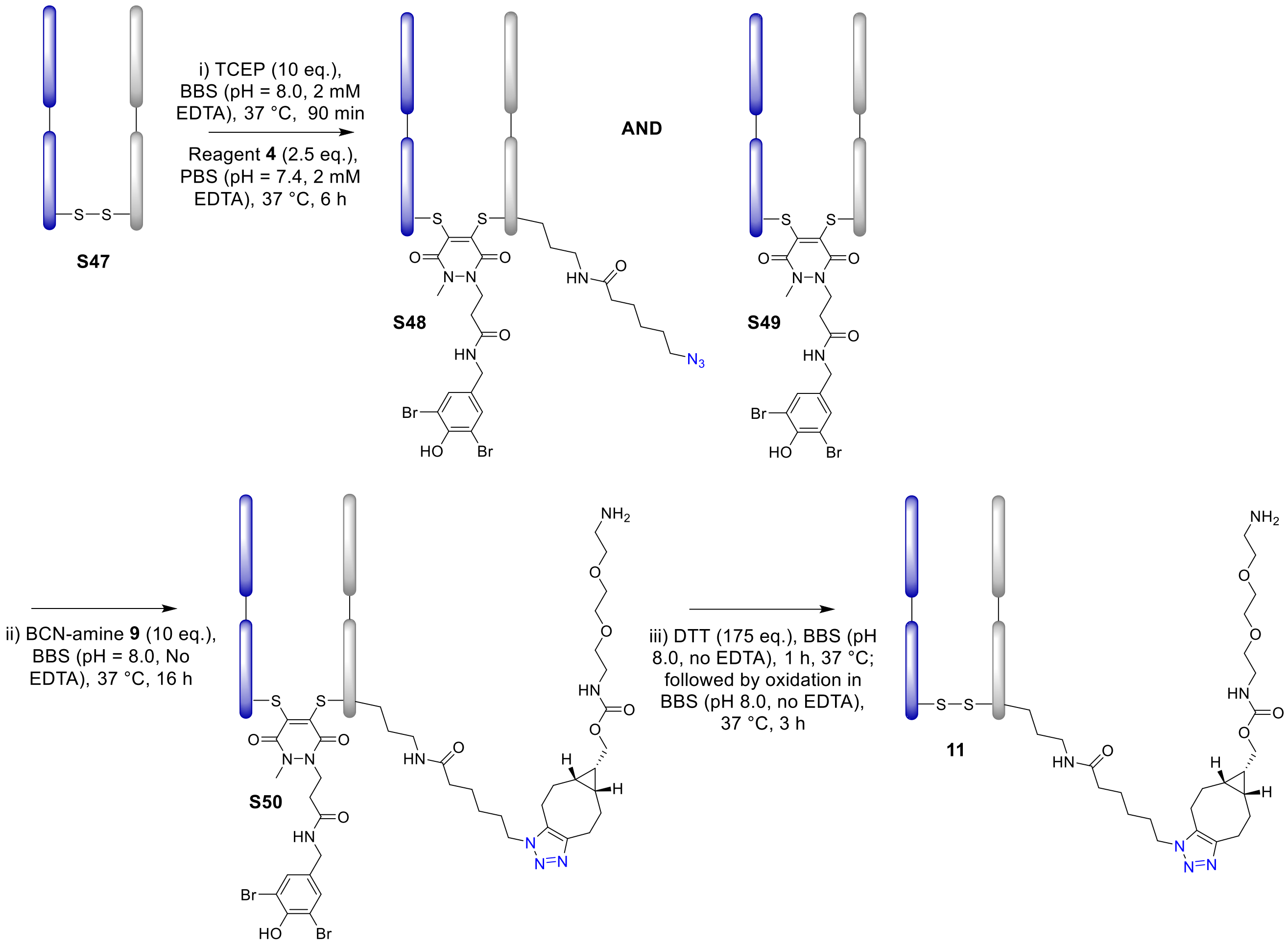

Results were obtained using general procedure 2.7.1**.**, using PD reagent **4**.

a) Conjugation step (i)

Conjugate **S48**: Expected mass: 47778.64 Da, observed mass: 47778.31 Da

Hydrolysed conjugate **S49**: Expected mass: 47639.48 Da, Observed mass: 47639.89 Da

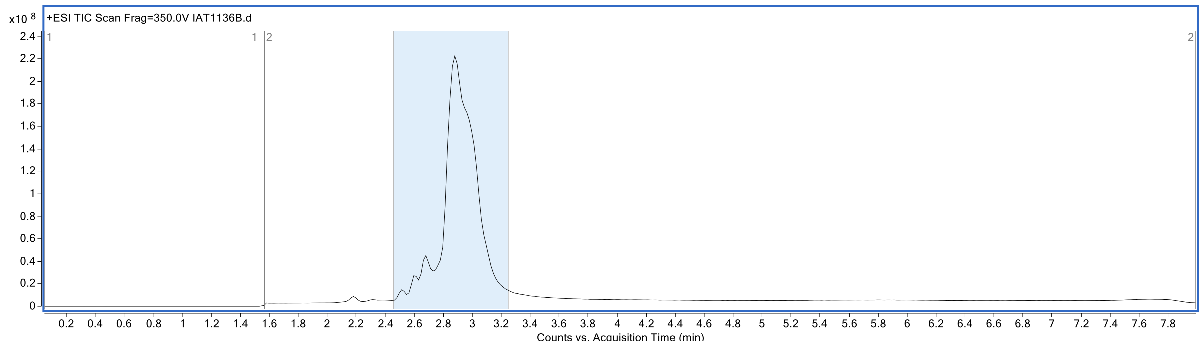

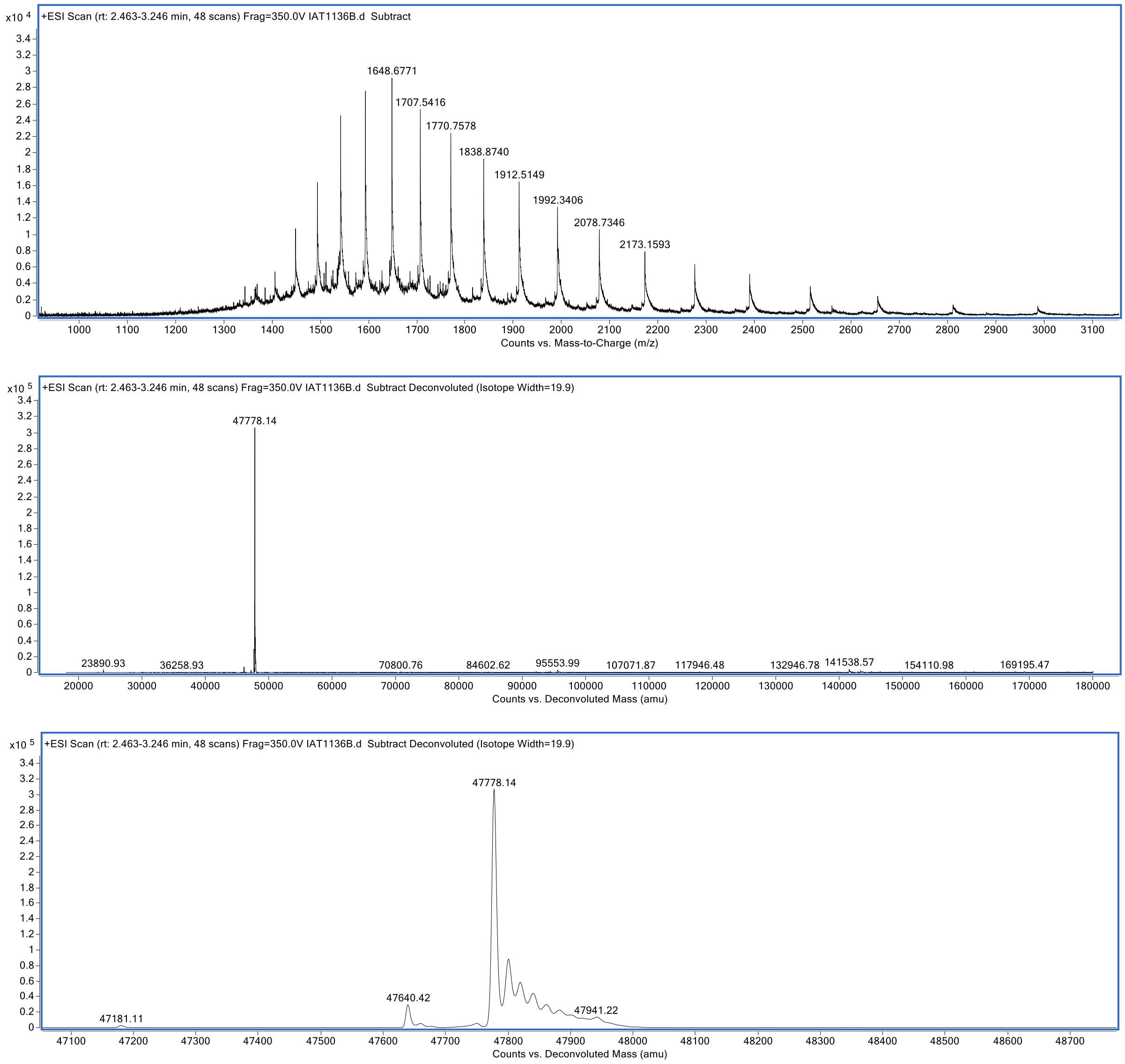

Figure S85: (i) TIC LC-MS trace (top), (ii) non-deconvoluted LC-MS trace (upper middle), (iii) deconvoluted MS data (lower middle, wide range), (iv) bottom (zoom in mass range) for conjugation step.

b) Click step (ii)

Conjugate **S50**: Expected mass: 48102.06 Da, observed mass: 48102.76 Da

Hydrolysed conjugate **S49**: Expected mass: 47639.48 Da, Observed mass: 47640.11 Da

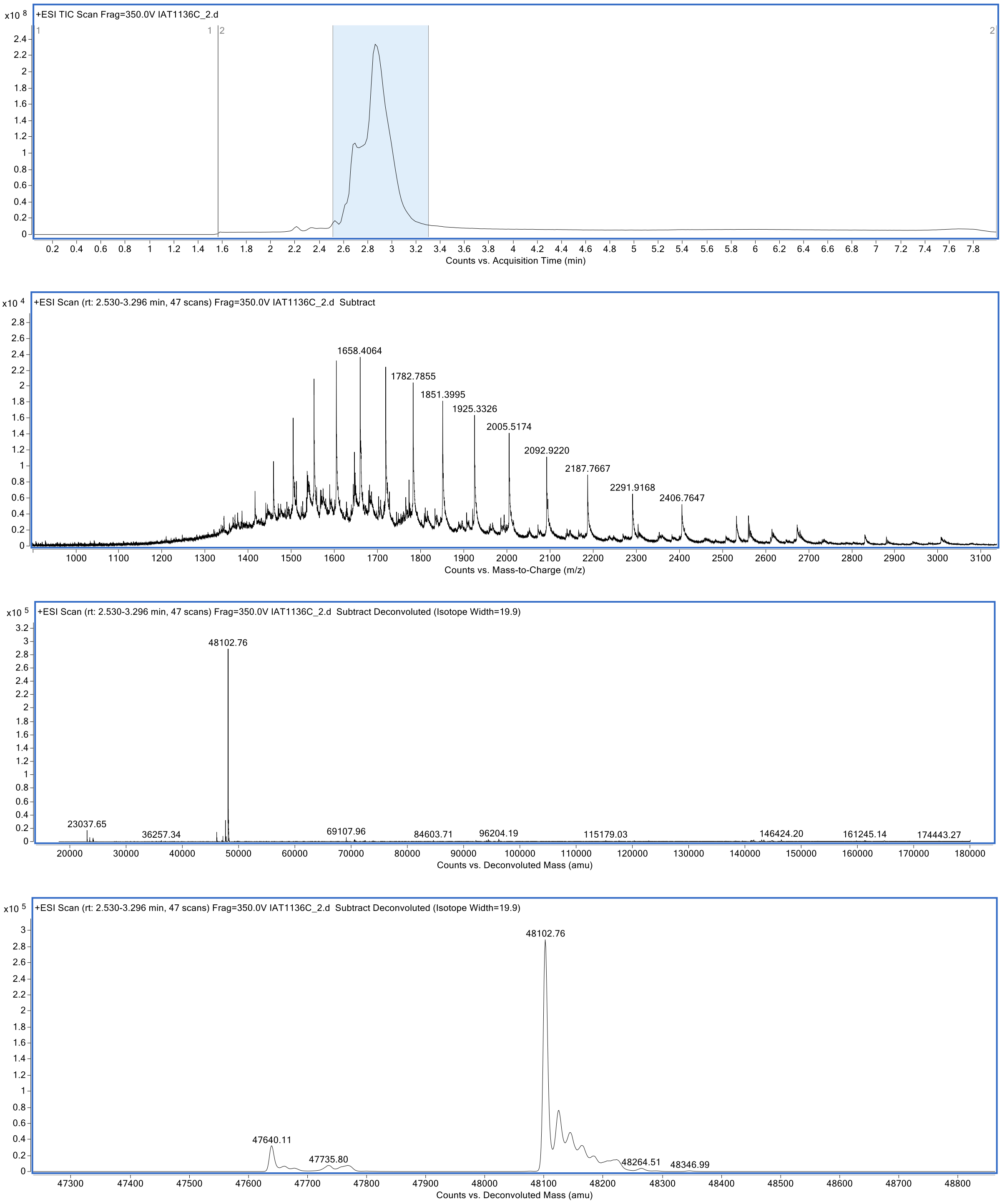

Figure S86: (i) TIC LC-MS trace (top), (ii) non-deconvoluted LC-MS trace (upper middle), (iii) deconvoluted MS data (lower middle, wide range), (iv) bottom (zoom in mass range) for click step.

c) Restoration step

Mono-labelled conjugate **11**: Expected mass: 47644.99 Da, observed mass: 47643.79 Da **(%Abundance: 90%)**

Fab$_{CD20}$ **S47**: Expected mass: 47639.10 Da, observed mass: 47639.33 Da **(%Abundance: 10%)**

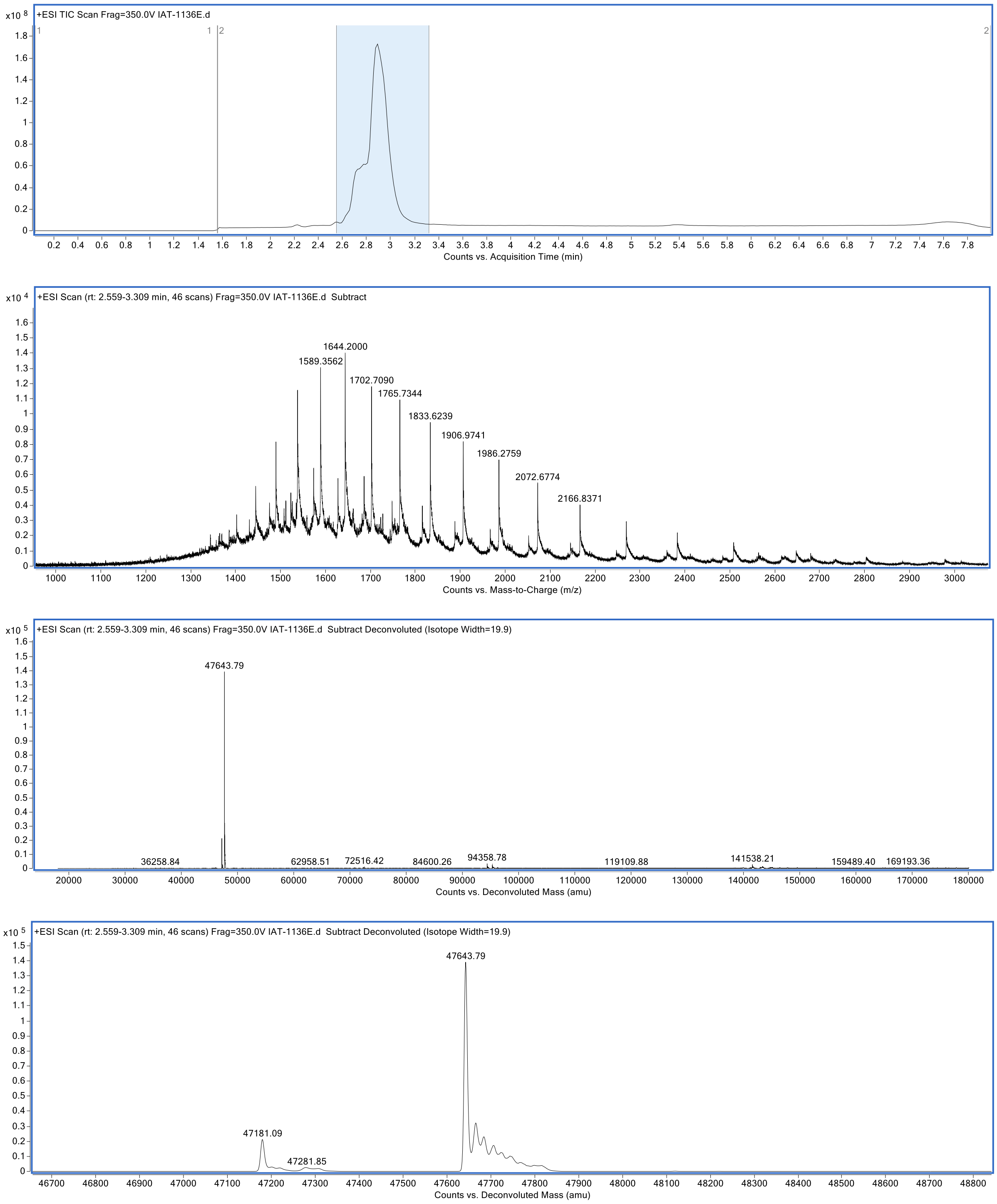

Figure S87: (i) TIC LC-MS trace (top), (ii) non-deconvoluted LC-MS trace (upper middle), (iii) deconvoluted MS data (lower middle, wide range), (iv) bottom (zoom in mass range) for disulfide restoration step.

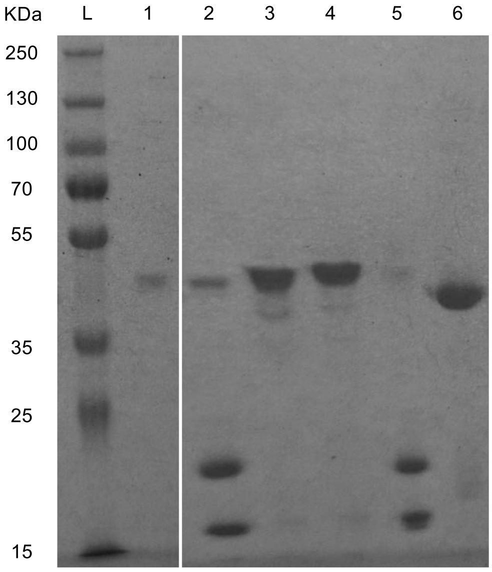

Figure S88: SDS-PAGE gel: L) Ladder, 1) Native Fab$_{CD20}$ **S47**, 2) Reduction step, 3) Conjugation/Lysine transfer step, 4) Deprotection step, 5) Disulfide restoration step.

2.8.2 Reaction of Fab$_{CD3}$ with reagent **4** and BCN-PEG2-amine **9**

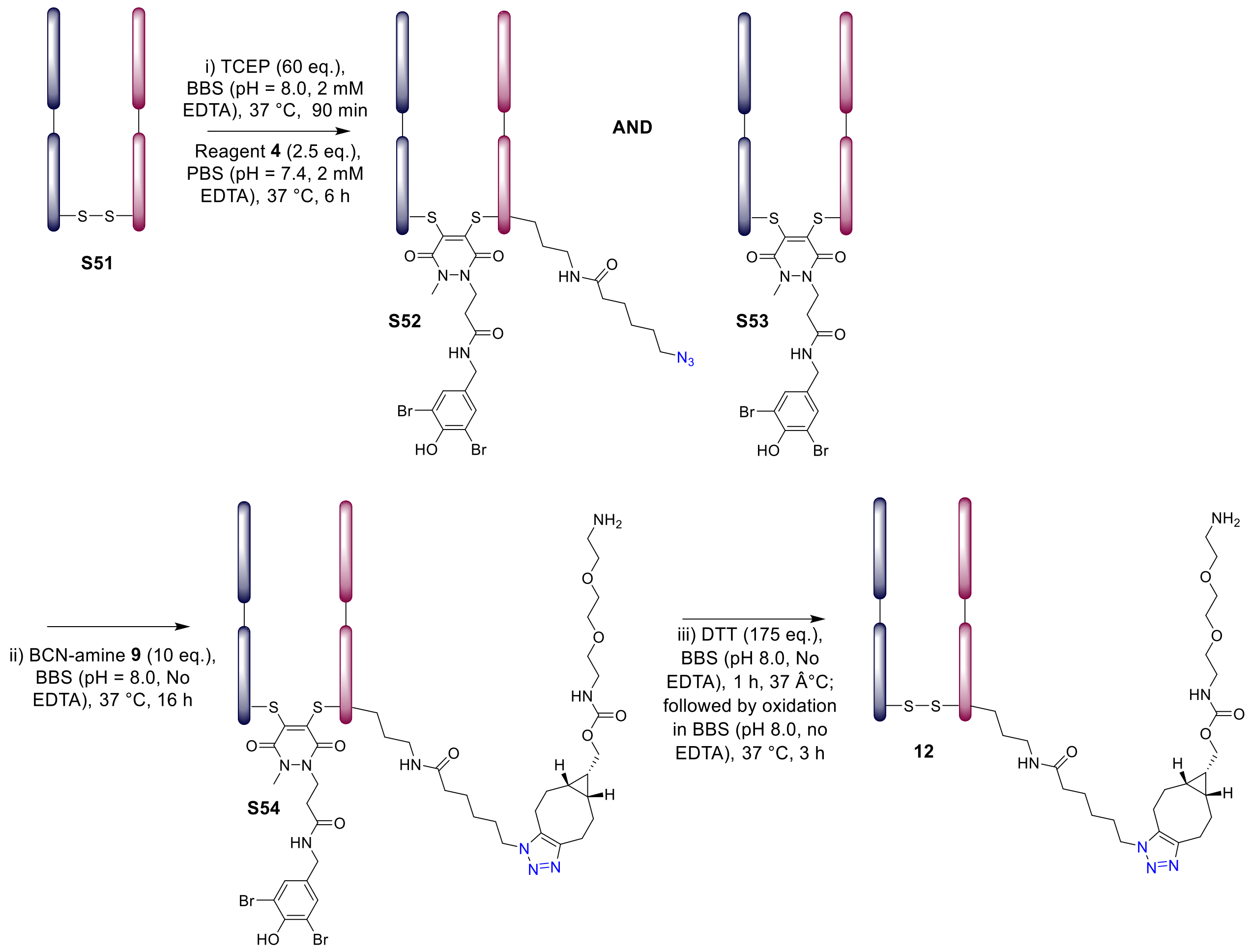

Results were obtained using general procedure 2.7.1.*, using PD reagent **4**. *60 eq. of TCEP·HCl were used for Fab$_{CD3}$ reduction.

a) Conjugation step (i)

Conjugate **S52**: Expected mass: 48045.24 Da, observed mass: 48046.03 Da

Hydrolysed conjugate **S53**: Expected mass: 47906.08 Da, Observed mass: 47907.82 Da

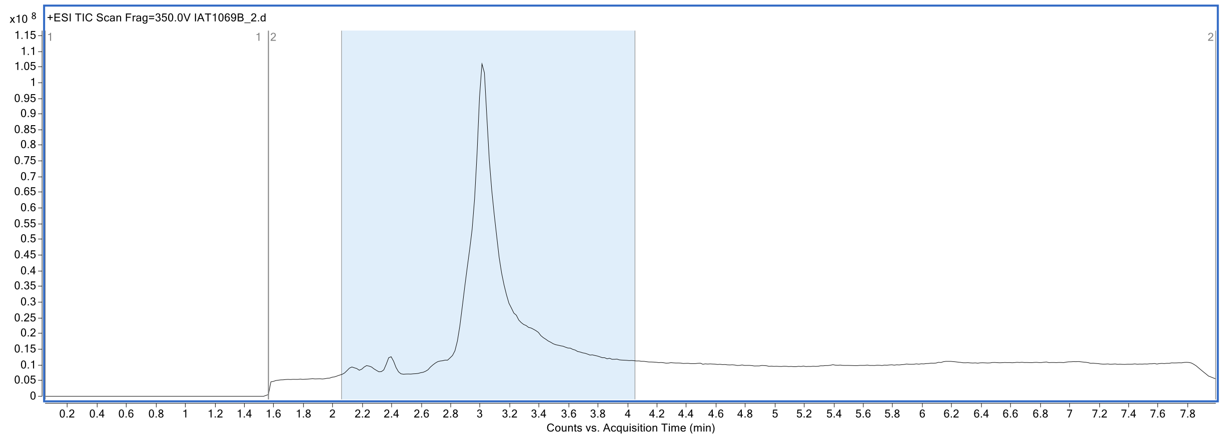

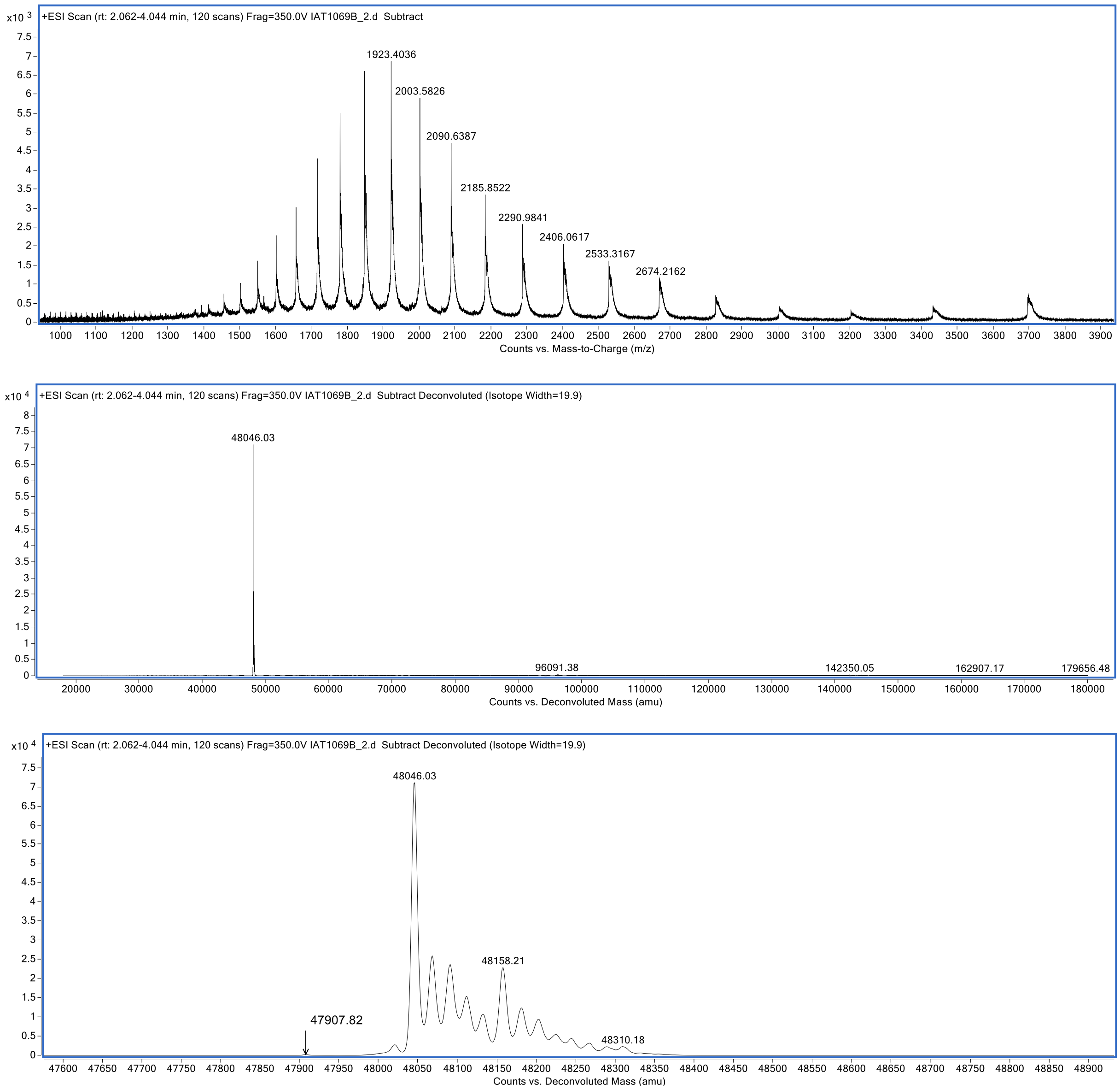

Figure S89: (i) TIC LC-MS trace (top), (ii) non-deconvoluted LC-MS trace (upper middle) (iii) deconvoluted MS data (lower middle, wide range), (iv) bottom (zoom in mass range) for conjugation step.

b) Click step (ii)

Conjugate **S54**: Expected mass: 48369.66 Da, observed mass: 48370.64 Da

Hydrolysed conjugate **S53**: Expected mass: 47906.08 Da, Observed mass: 47908.41 Da

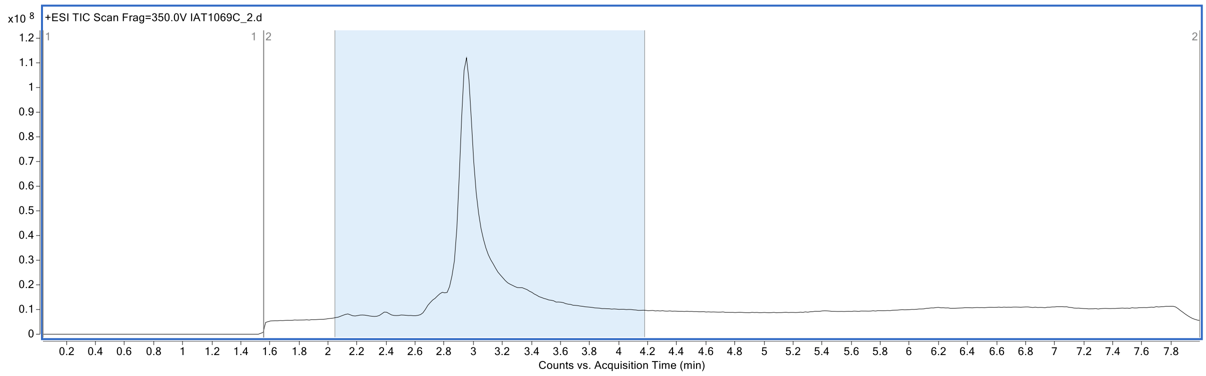

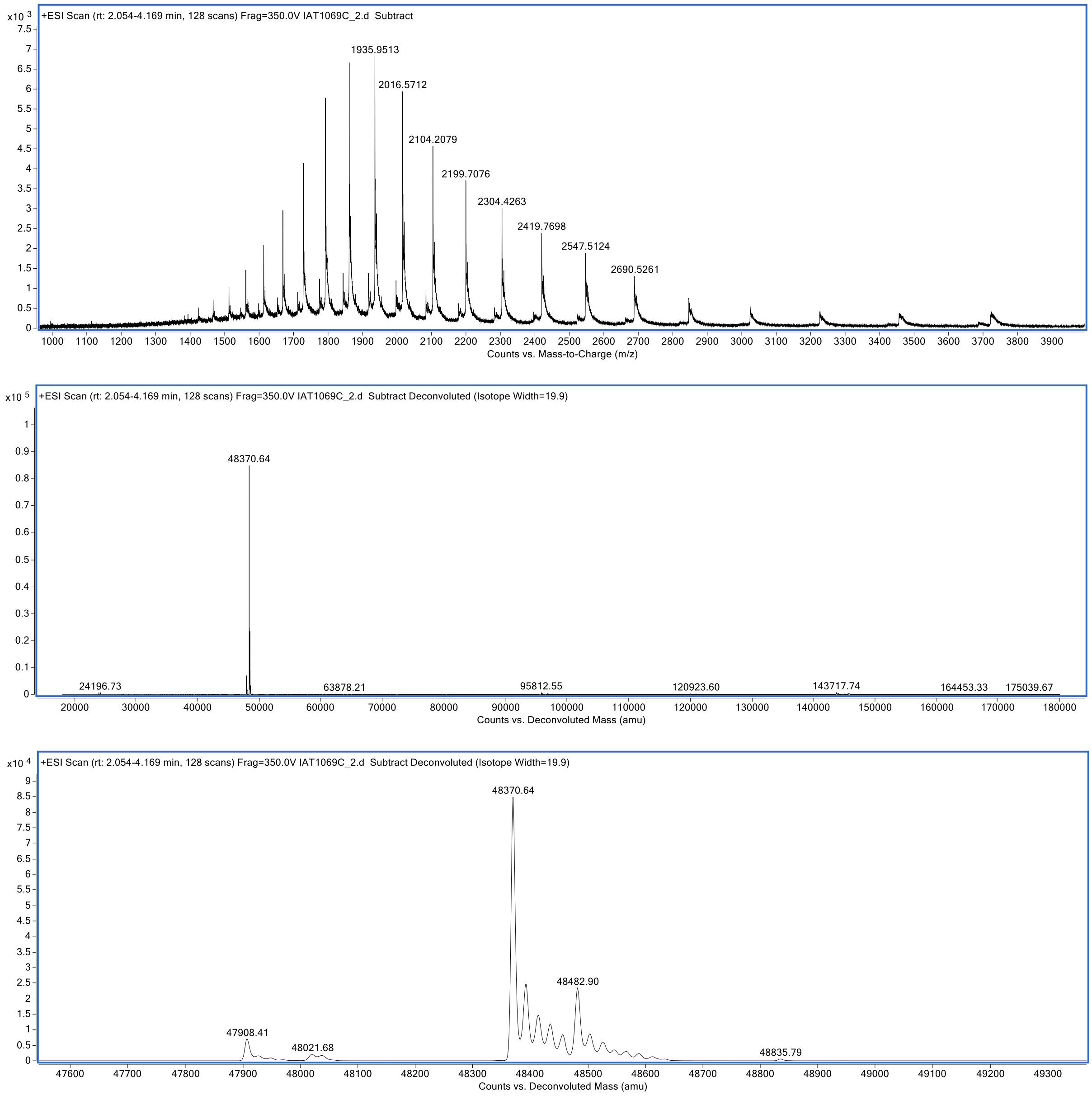

Figure S90: (i) TIC LC-MS trace (top), (ii) non-deconvoluted LC-MS trace (upper middle), (iii) deconvoluted MS data (lower middle, wide range), (iv) bottom (zoom in mass range) for click step.

c) Restoration step

Mono-labelled conjugate **12**: Expected mass: 47911.59 Da, observed mass: 47911.14 Da **(%Abundance: 90%)**

Fab$_{CD3}$ **S49**: Expected mass: 47447.00 Da, observed mass: 47911.14 Da

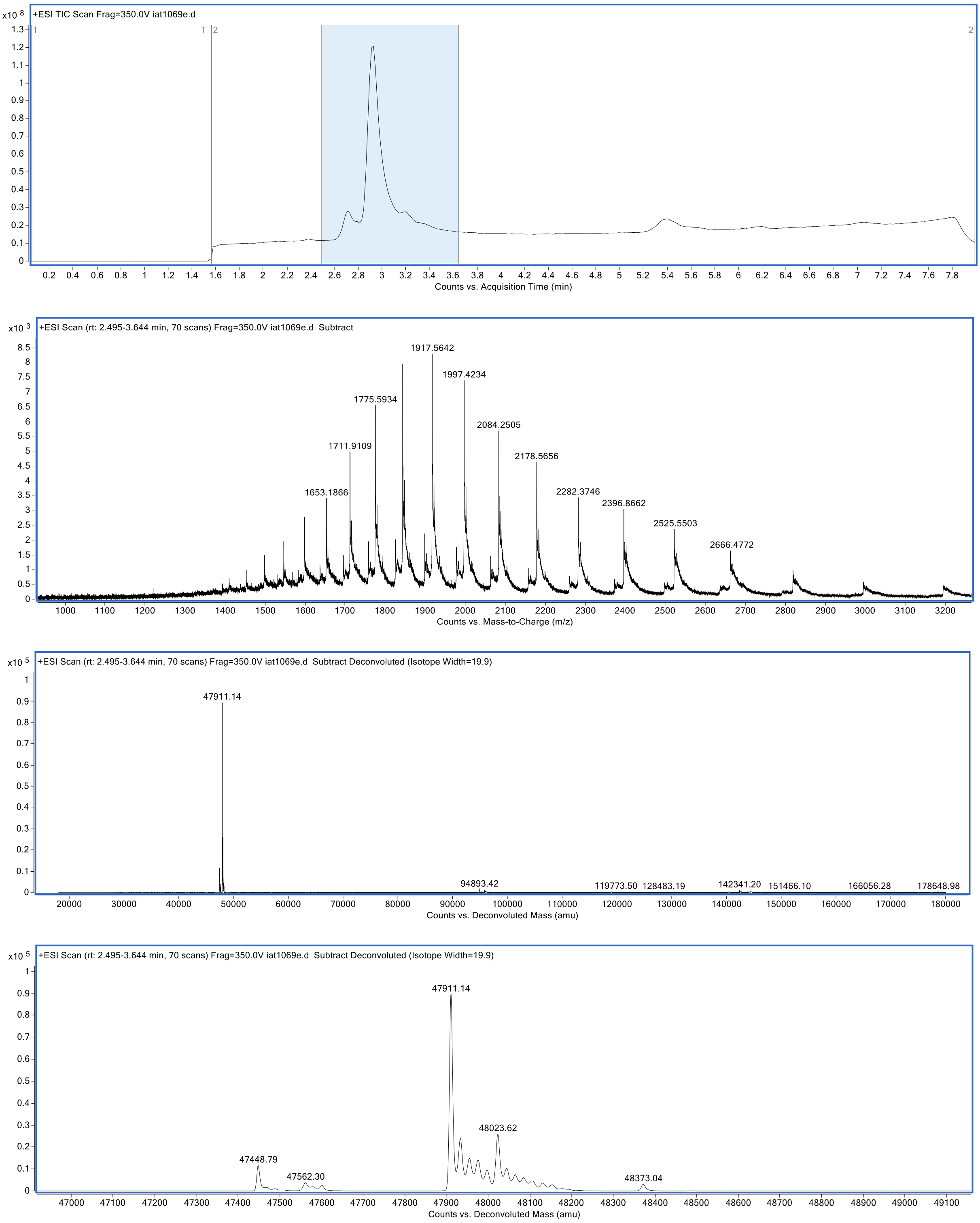

Figure S91: (i) TIC LC-MS trace (top), (ii) non-deconvoluted LC-MS trace (upper middle), (iii) deconvoluted MS data (lower middle, wide range), (iv) bottom (zoom in mass range) for disulfide restoration step.

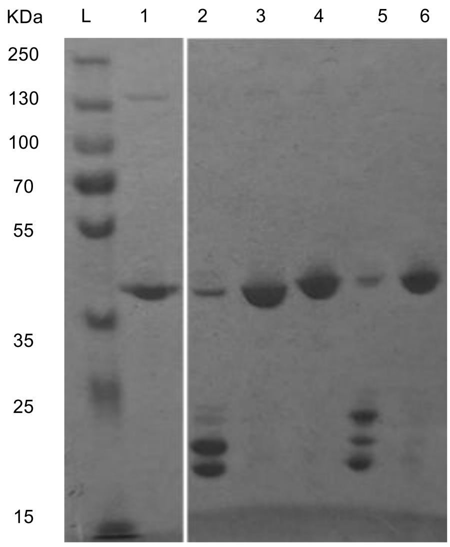

Figure S92: SDS-PAGE gel: L) Ladder, 1) Native Fab$_{CD3}$ **S51**, 2) Reduction step, 3) Conjugation/Lysine reaction step, 4) Click reaction step, 5) Deprotection step, 6) Disulfide restoration step.

3.0 Reaction of Ontruzant Fab **1** with reagent **4** and 5-FAM-PEG3-BCN (exo) **13** to make conjugate **14**

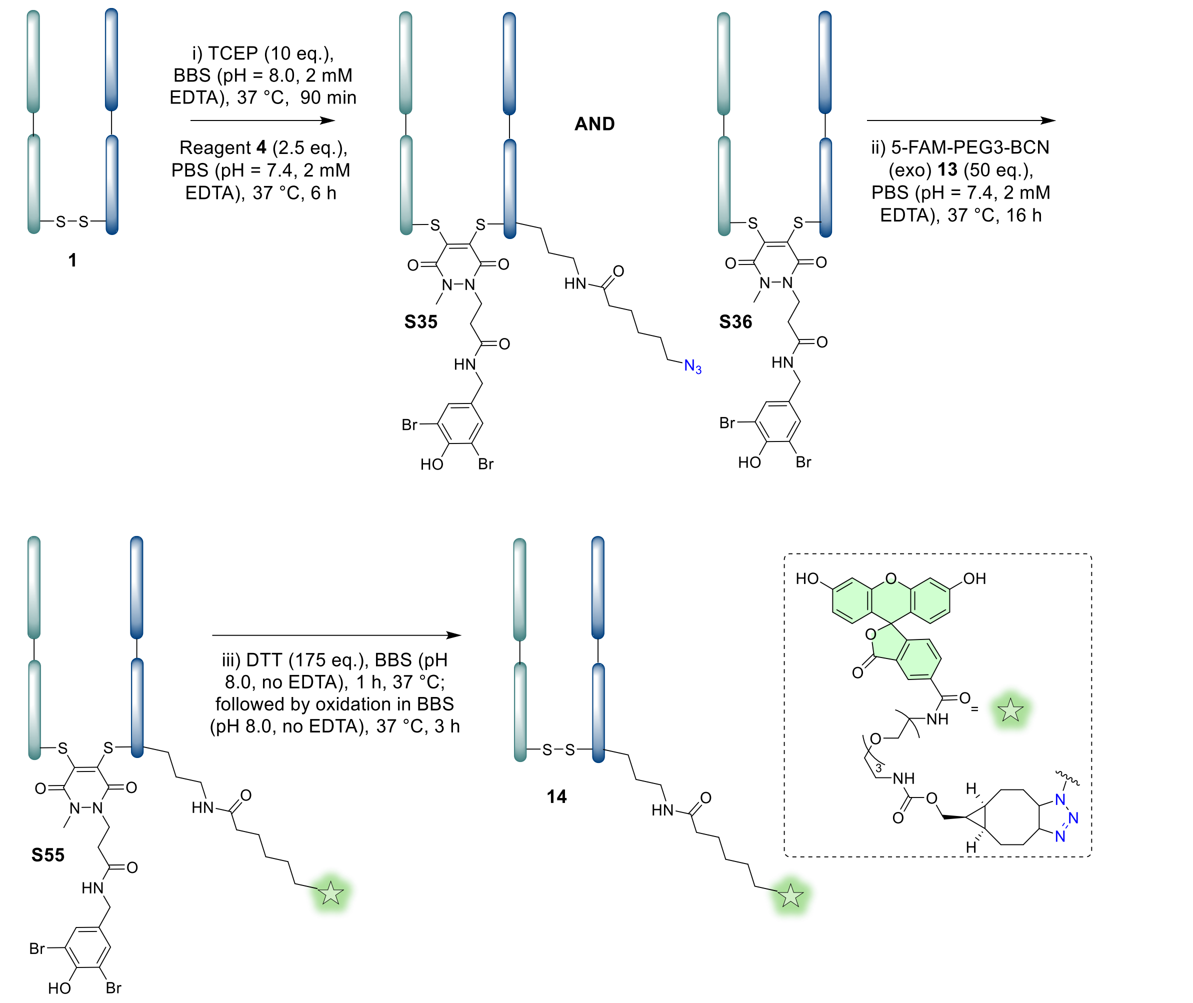

Results were obtained using general procedure 2.7.1.* using PD reagent **4**. *5-FAM-PEG3-BCN (exo) **13** (10 mM in DMSO, 50 eq.) was used in place of BCN-PEG2-amine **9** in the click reaction step.

a) Conjugation step (i)

Conjugate **S35**: Expected mass: 48236.34 Da, observed mass: 48236.71 Da

Hydrolysed conjugate **S36**: Expected mass: 48098.18 Da, Observed mass: 48098.92 Da

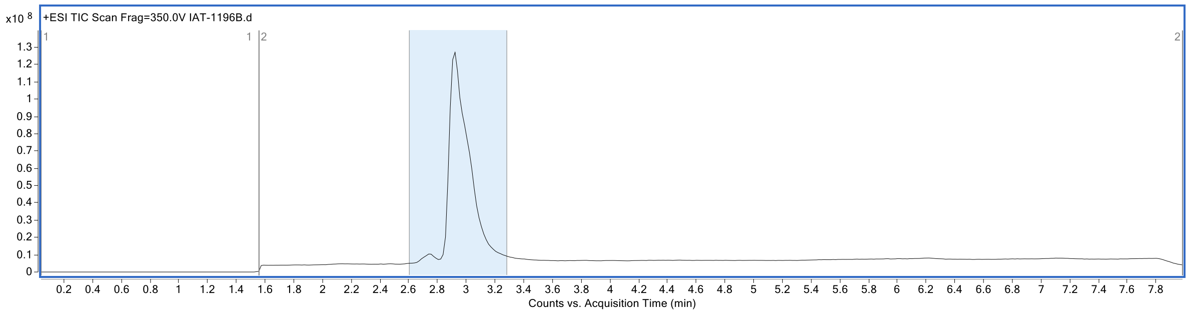

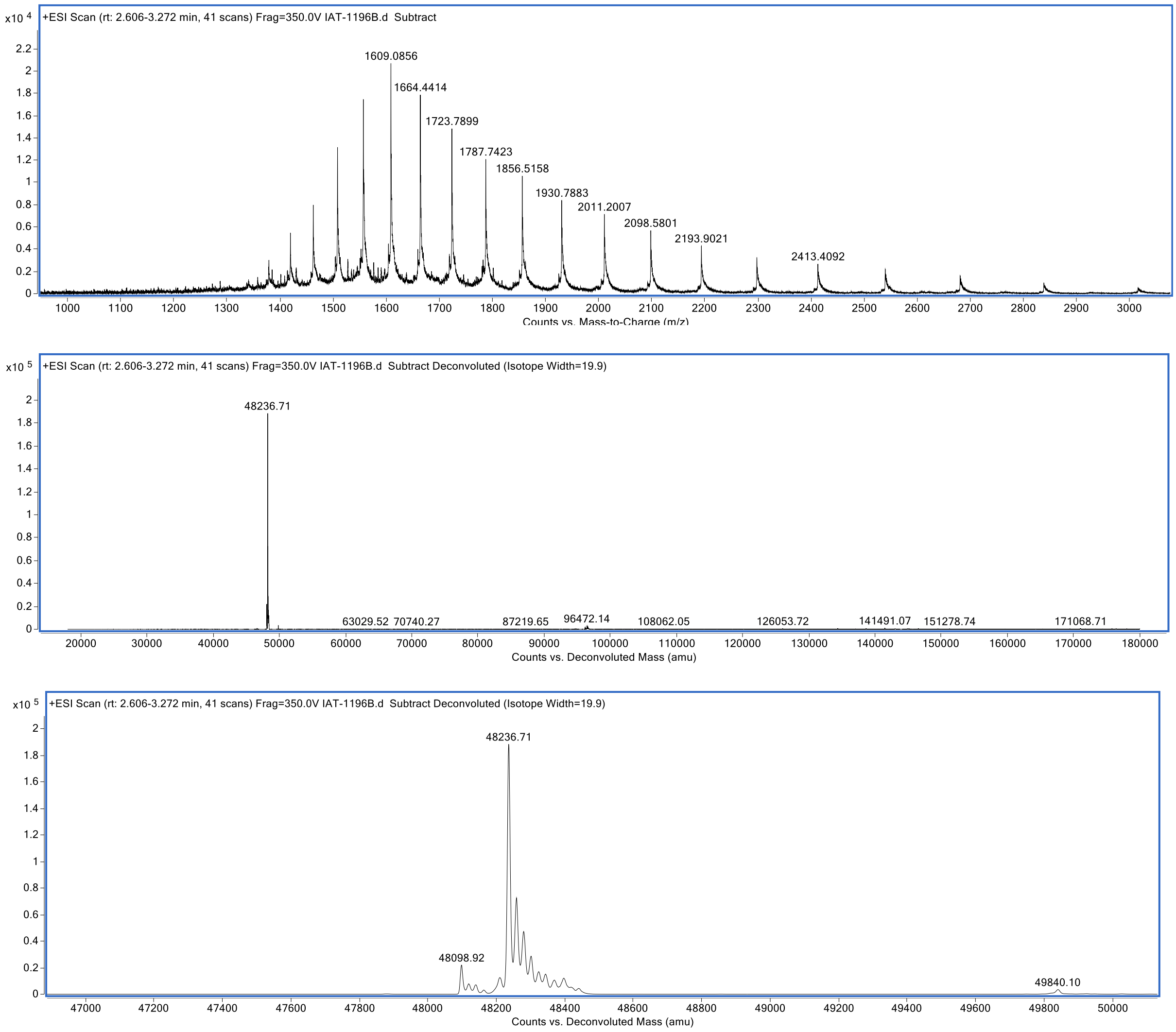

Figure S93: (i) TIC LC-MS trace (top), (ii) non-deconvoluted LC-MS trace (upper middle), (iii) deconvoluted MS data (lower middle, wide range), (iv) bottom (zoom in mass range) for conjugation/lysine reaction step.

b) Click step (ii)

Conjugate **S55**: Expected mass: 48963.11 Da, observed mass: 48964.14 Da

Hydrolysed conjugate **S36**: Expected mass: 48098.18 Da, Observed mass: 48103.70 Da

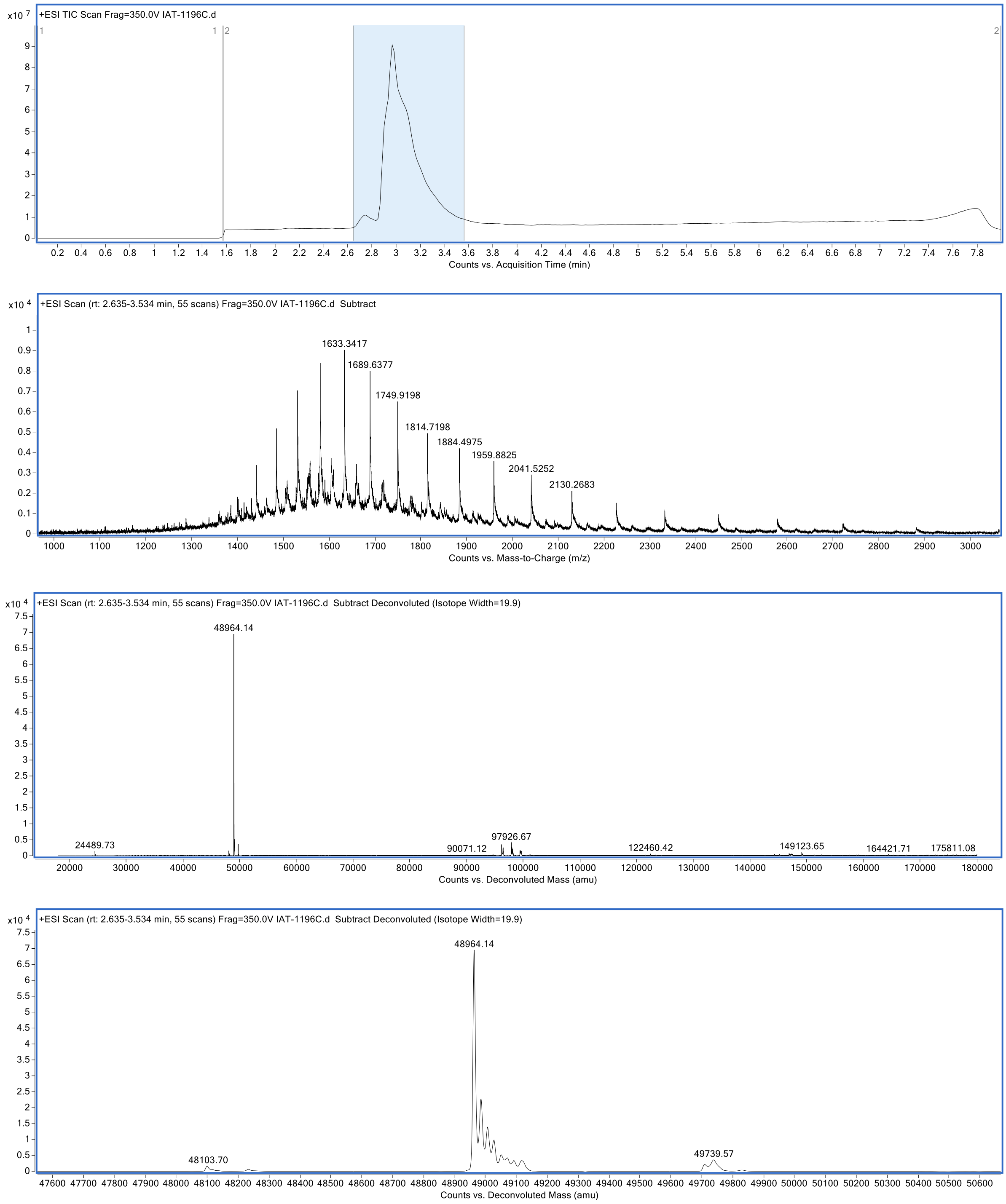

Figure S94: (i) TIC LC-MS trace (top), (ii) non-deconvoluted LC-MS trace (upper middle), (iii) deconvoluted MS data (lower middle, wide range), (iv) bottom (zoom in mass range) for click step.

c) Restoration step

Mono-labelled conjugate **14**: Expected mass: 48506.44 Da, observed mass: 48505.75 Da

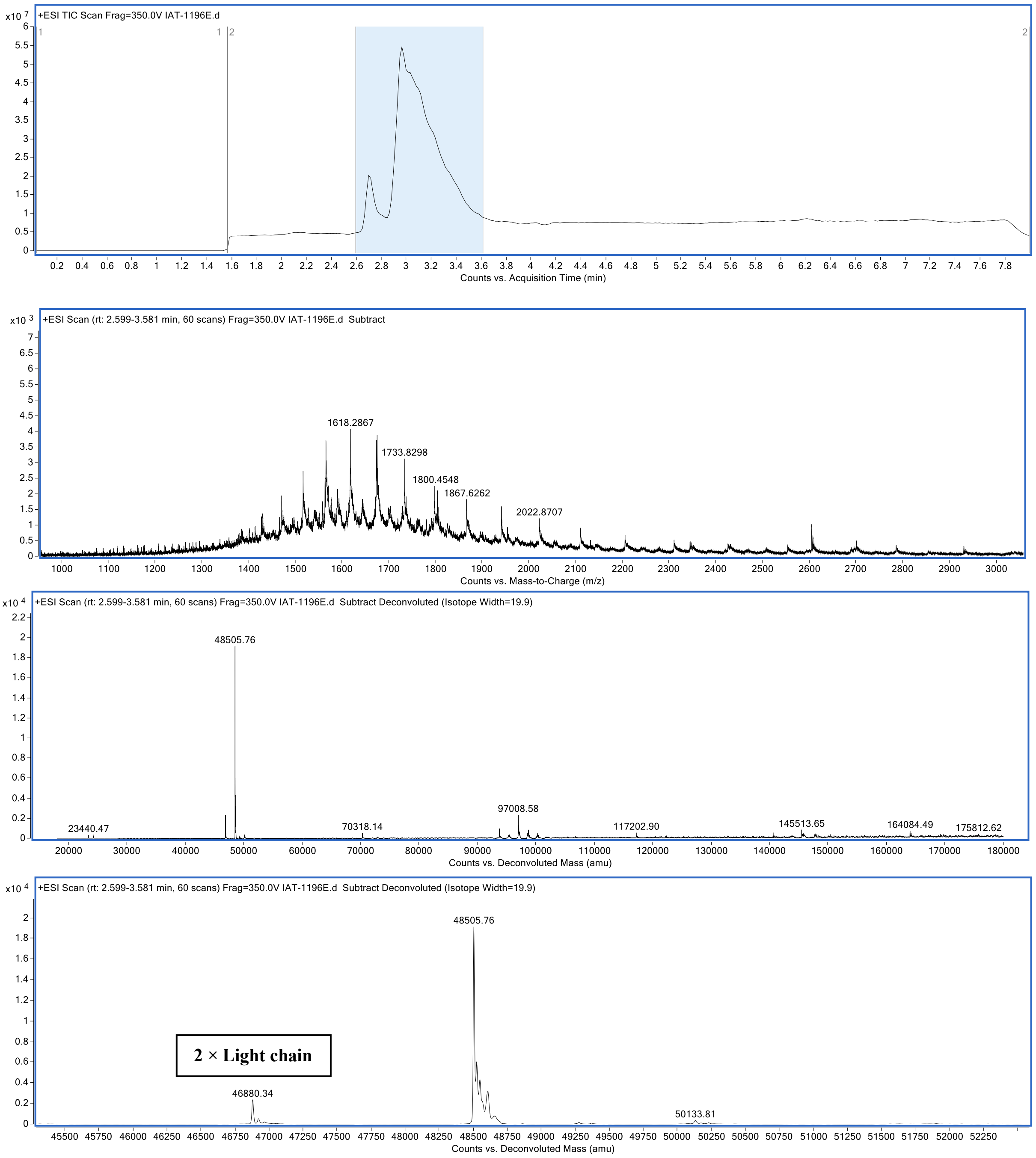

Figure S95: (i) TIC LC-MS trace (top), (ii) non-deconvoluted LC-MS trace (upper middle), (iii) deconvoluted MS data (lower middle, wide range), (iv) bottom (zoom in mass range) for disulfide restoration step. Masses in the ca. 97,000 Da region are spurious mass spectrometry artefacts that are the double masses of the conjugates observed in the Fab region; these higher mass species are not observed by SDS-PAGE. The final % abundance of conjugate **14** was determined by analysis of Figure S93 as the disulfide bond of the native Fab did not appear to be fully reformed in Figure S95 when carrying out the mass specturm analysis (this is somewhat evidenced by the “2 × Light chain” peak).

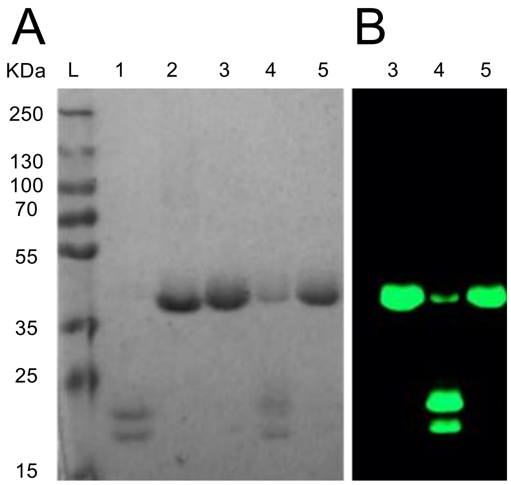

Figure S96: A) SDS-PAGE gel: L) Ladder, 1) Native Fab, 2) Reduction step, 3) Conjugation /Lysine reaction step, 4) Deprotection step, 5) Disulfide restoration step. B) Gel visualisation of lanes 3), 4) and 5) using a light source of 472 nm (Blue LED) and filter**:** 595 nm (Orange EM).

Fluorophore: 5-FAM-PEG3-BCN (exo), using FITC as a reference on ThermoFischer Scientific Fluorescence SpectraViewer

Excitation max: FITC: 499 nm, 5-FAM-PEG3-BCN (exo): 493 nm

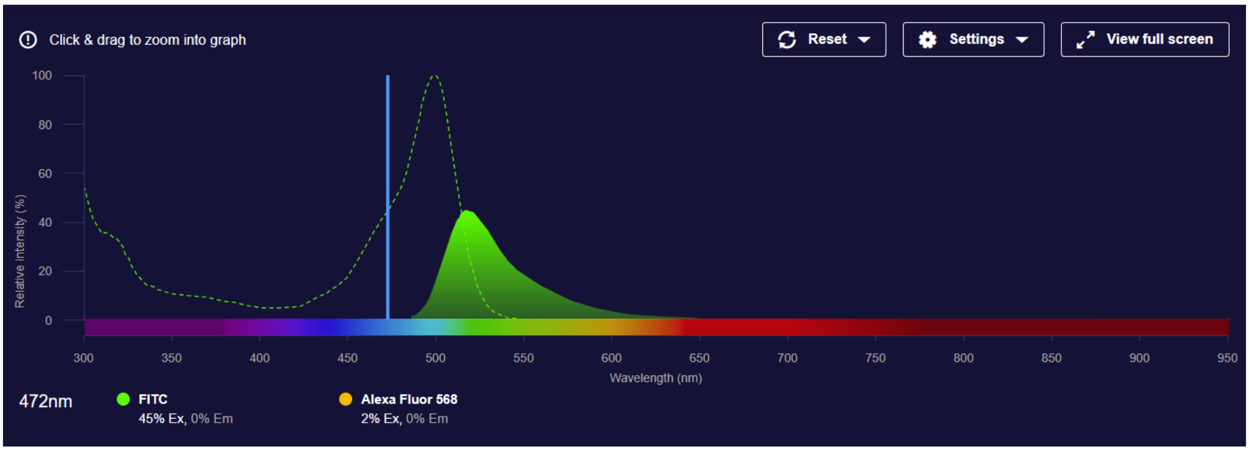

Figure S97: ThermoFischer Scientific Fluorescence SpectraViewer showing the excitation (dashed green) and emission profile of FITC as a reference, light source of 472 nm.

3.1 Formation of dually modified Ontruzant Fab conjugate **16**

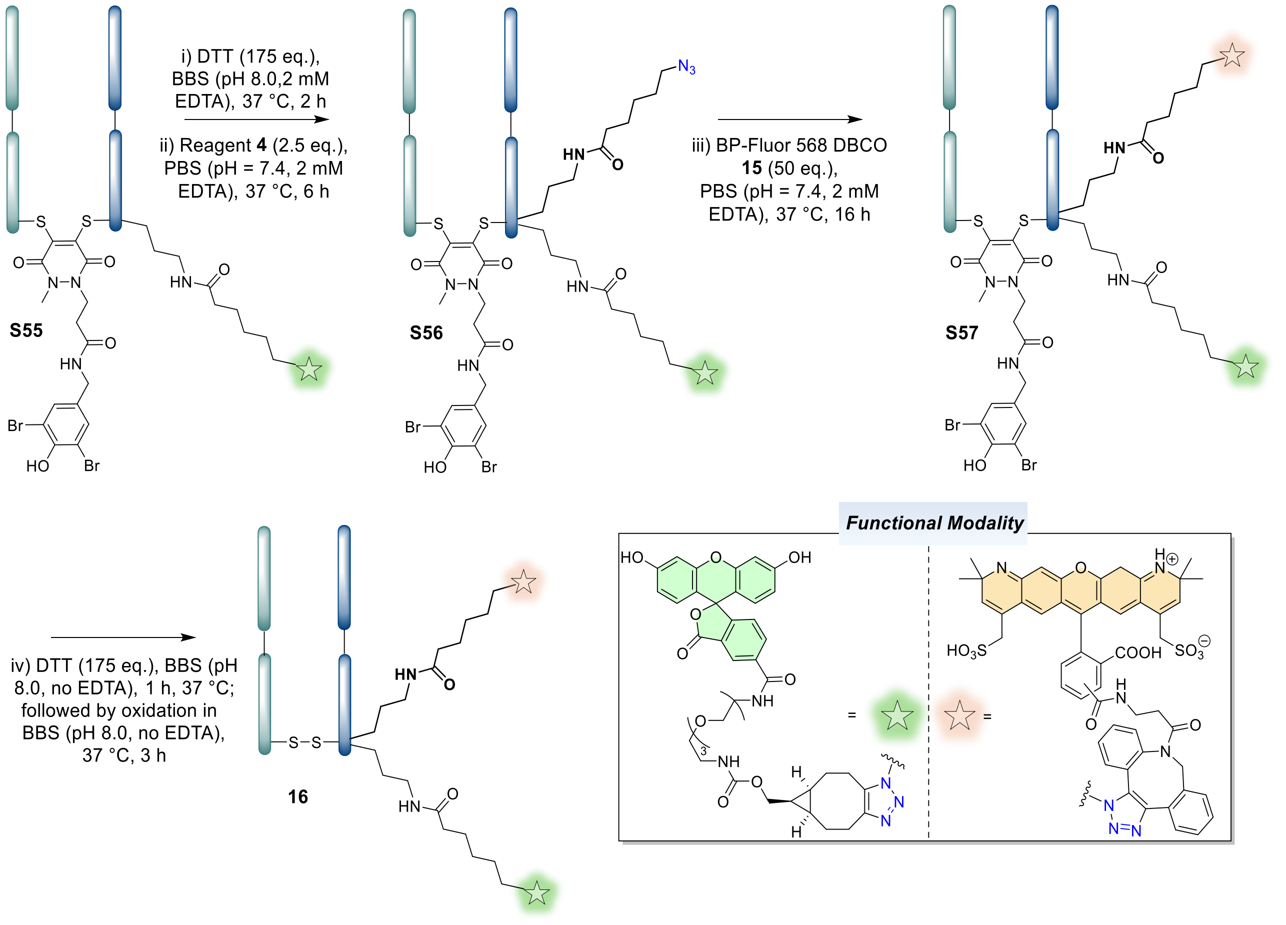

Results were obtained using general procedure 2.7.1.* using reagent **4**. *Application of the optimised protocol for mono-labelling and clicking of a Fab (ESI 3.0) on Ontruzant Fab **1** in two cycles using 5-FAM-PEG3-BCN (exo) **13** (10 mM in DMSO, 50 eq.) (in cycle 1) and BP-Fluor568-DBCO **15** (20 mM in DMSO, 50 eq.) (in cycle 2) as the strained alkyne components and bypassing the disulfide restoration step at the end of cycle 1 to afford dually-labelled Fab conjugate **16**.

a) Conjugation step (ii)

Conjugate **S56**: Expected mass: 49103.28 Da, observed mass: 49103.33 Da

Conjugate **S55**: Expected mass: 48963.11 Da, observed mass: 48965.52 Da

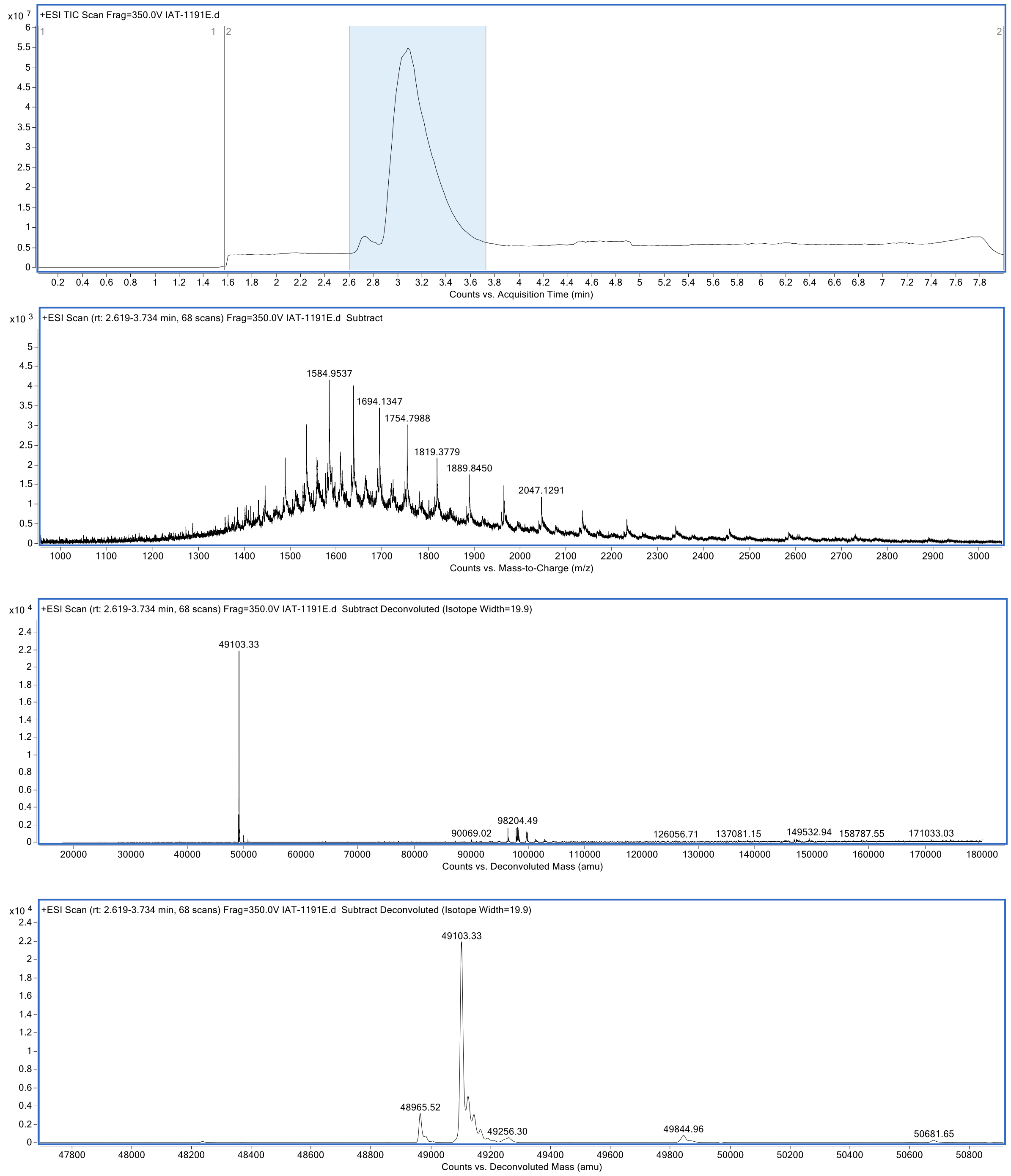

Figure S98: (i) TIC LC-MS trace (top), (ii) non-deconvoluted LC-MS trace (upper middle), (iii) deconvoluted MS data (lower middle, wide range), (iv) bottom (zoom in mass range) for conjugation step. Masses in the ca. 98,000 Da region are spurious mass spectrometry artefacts that are the double masses of the conjugates observed in the Fab region; these higher mass species are not observed by SDS-PAGE.

b) Click step (iii) with BP-Fluor568-DBCO **15**

Conjugate **S57**: Expected mass: 50056.38 Da, observed mass: 50057.18 Da

Conjugate **S55**: Expected mass: 48963.11 Da, observed mass: 48966.20 Da

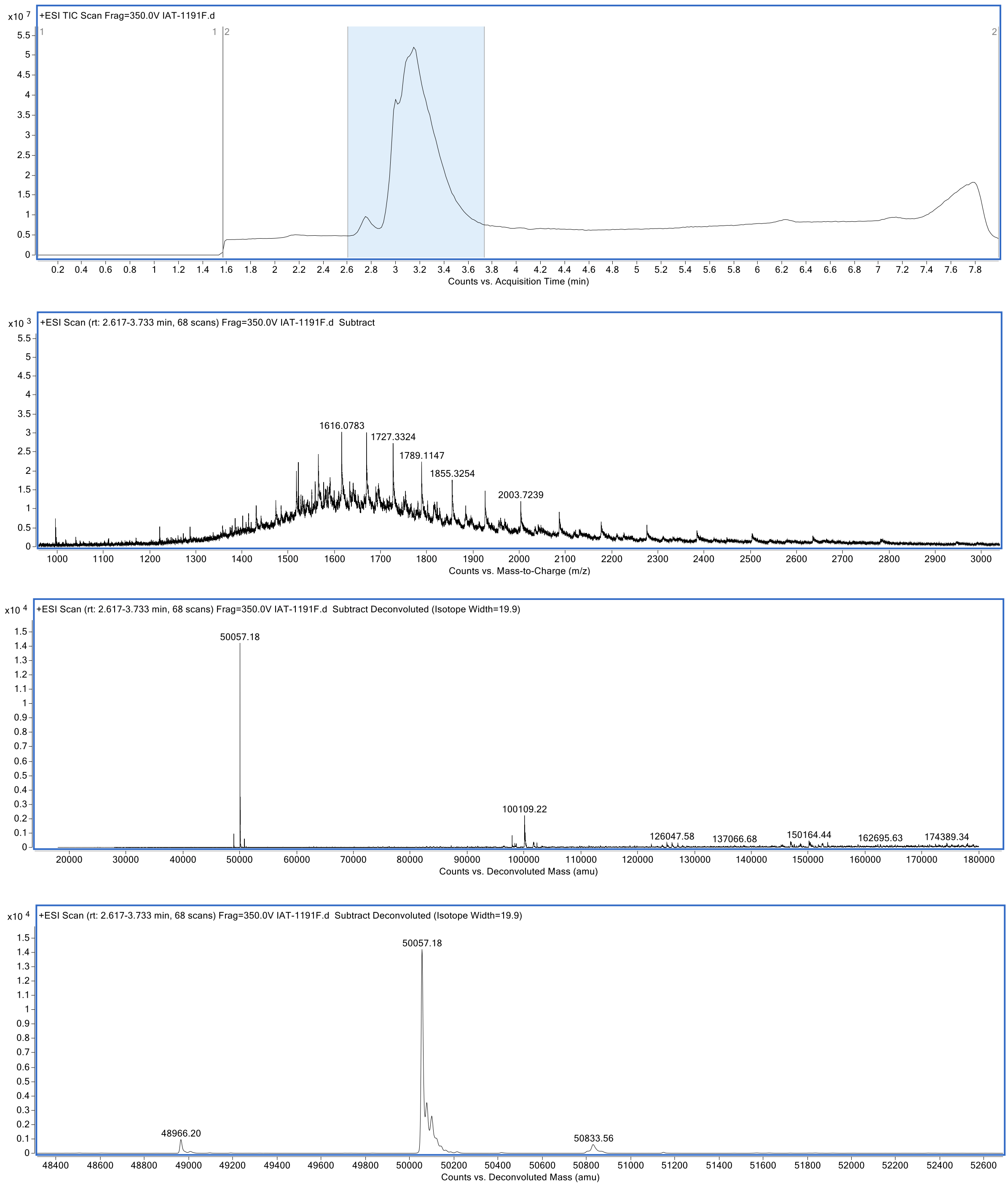

Figure S99: (i) TIC LC-MS trace (top), (ii) non-deconvoluted LC-MS trace (upper middle), (iii) deconvoluted MS data (lower middle, wide range), (iv) bottom (zoom in mass range) for click step.

d) Restoration step

Dually-labelled conjugate **16**: Expected mass: 49599.32 Da, observed mass: 49597.86 Da

Mono-labelled conjugate **14**: Expected mass: 48506.44 Da , observed mass: 48507.13 Da

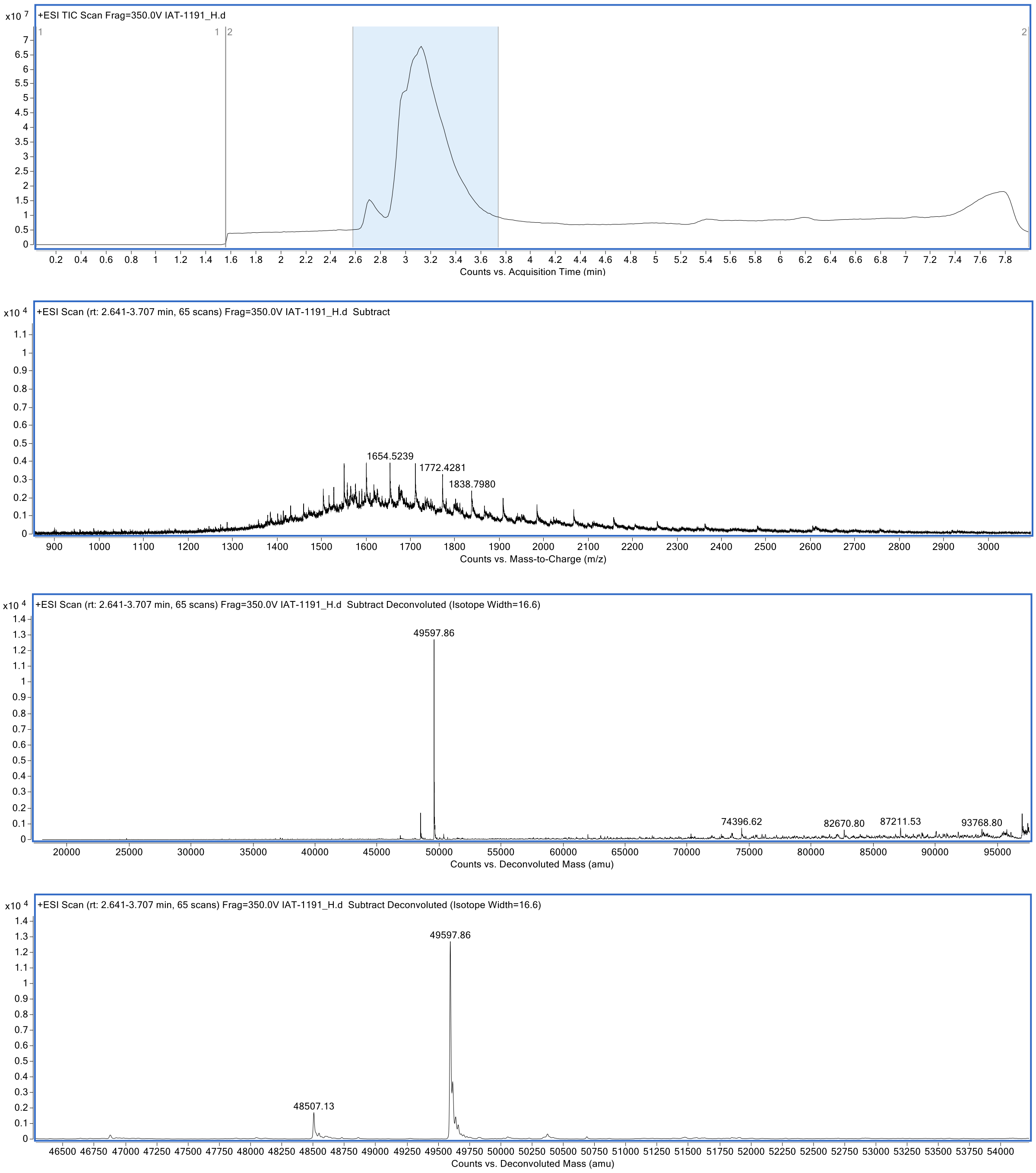

Figure S100: (i) TIC LC-MS trace (top), (ii) non-deconvoluted LC-MS trace (upper middle), (iii) deconvoluted MS data (lower middle, wide range), (iv) bottom (zoom in mass range) for restoration step.

Fluorophores: 5-FAM-PEG3-BCN (exo), using FITC as a reference, BP Fluor 568 DBCO using Alexa Fluor 568 as a reference on ThermoFischer Scientific Fluorescence SpectraViewer[5]

Excitation max: FITC: 499 nm, 5-FAM-PEG3-BCN (exo): 493 nm

Excitation max: Alexa Fluor 568: 578 nm, BP Fluor 568 DBCO 578 nm

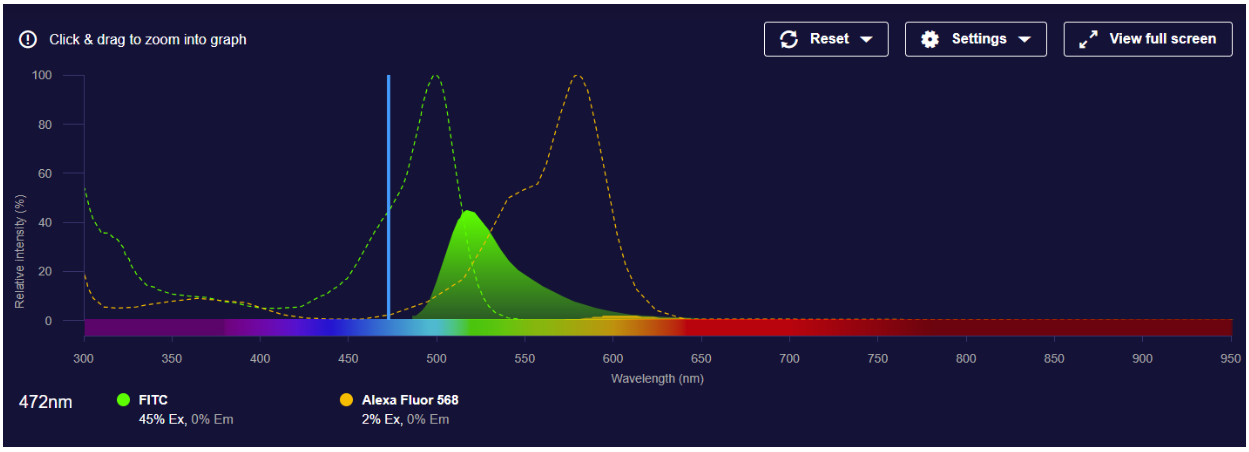

Figure S101: ThermoFischer Scientific Fluorescence SpectraViewer showing the excitation (dashed green) and emission profile of FITC as a reference, light source of 472 nm.[5]

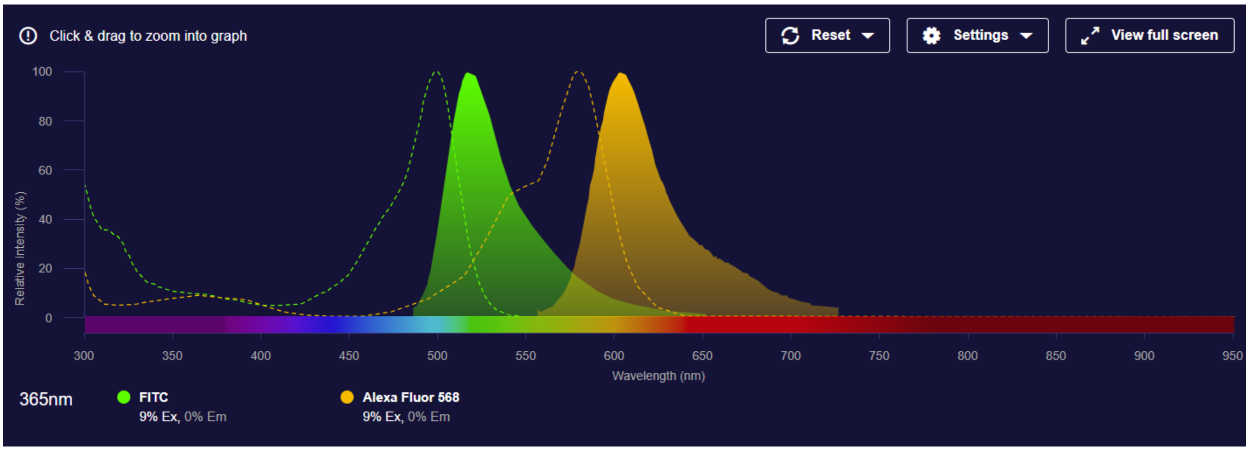

Figure S102: ThermoFischer Scientific Fluorescence SpectraViewer showing the excitation (dashed green) and emission profile of FITC as a reference, light source of 365 nm.[5]

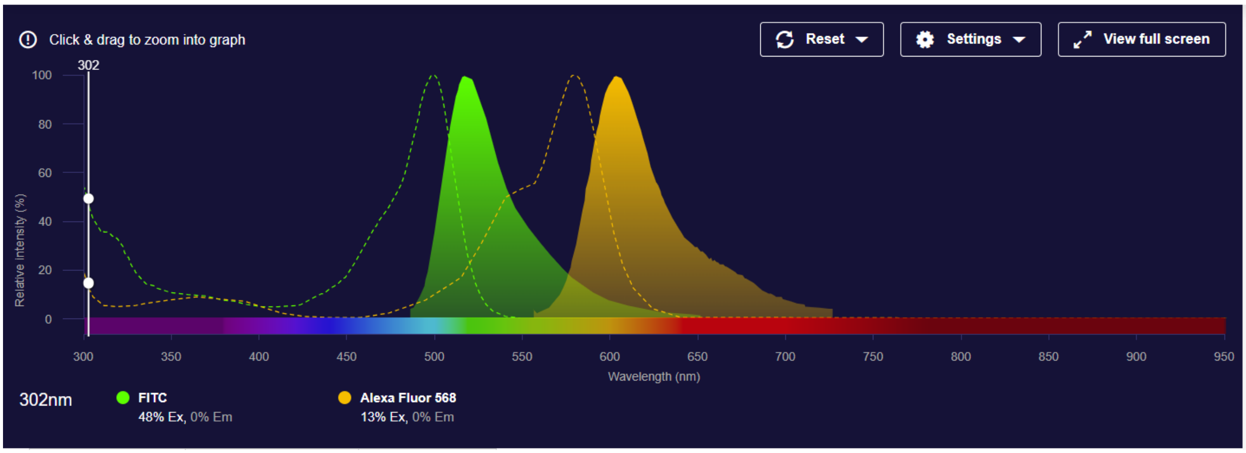

Figure S103: ThermoFischer Scientific Fluorescence SpectraViewer showing the excitation (dashed green) and emission profile of FITC as a reference, light source of 302 nm.[5]

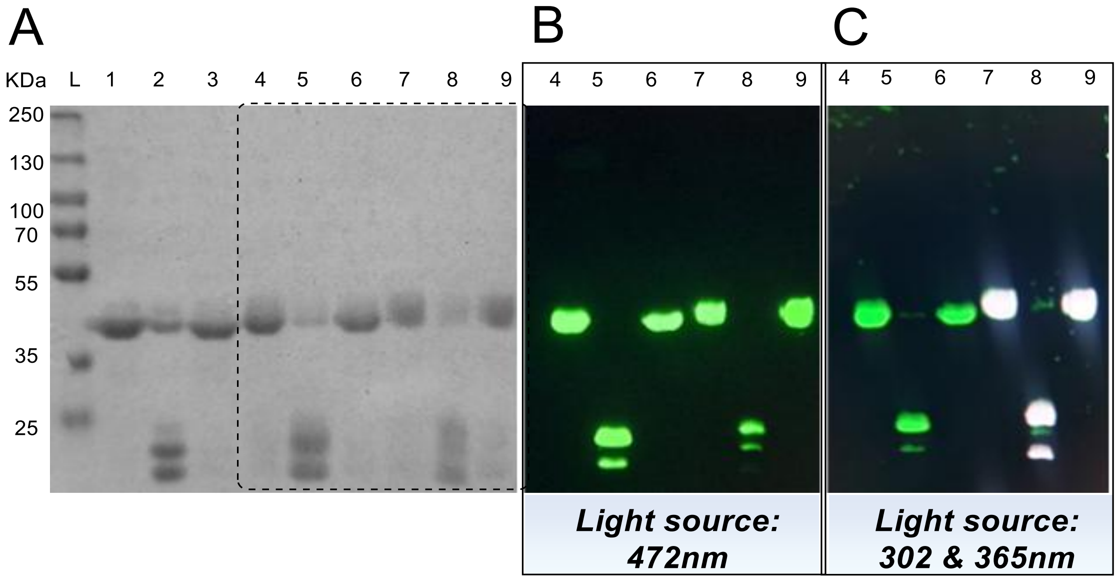

Figure S104: A) L: Ladder, 1: Ontruzant Fab **1**, 2: Reduction step, 3: Conjugation step, 4: Click reaction step, 5: Deprotection step, 6: Addition of reagent **4**, 7: Click reaction with BP-Fluor568-DBCO **15**, 8: Deprotection step, 9: Restoration step, B) Gel visualisation of lanes 4) - 9) using a light source of 472 nm (Blue LED) and filter**:** 595 nm (Orange EM), C) Gel visualisation of lanes 4) - 9) using a light source of 302 nm (Trans UV) & 365 nm (Trans UV) and filter**:** 595 nm (Orange EM).

3.2 Reaction of conjugate **10** (made using reagent **4** and BCN-PEG2-amine **9**) with reagent **4** and DBCO-biotin to make dually modified Ontruzant Fab conjugate **17**

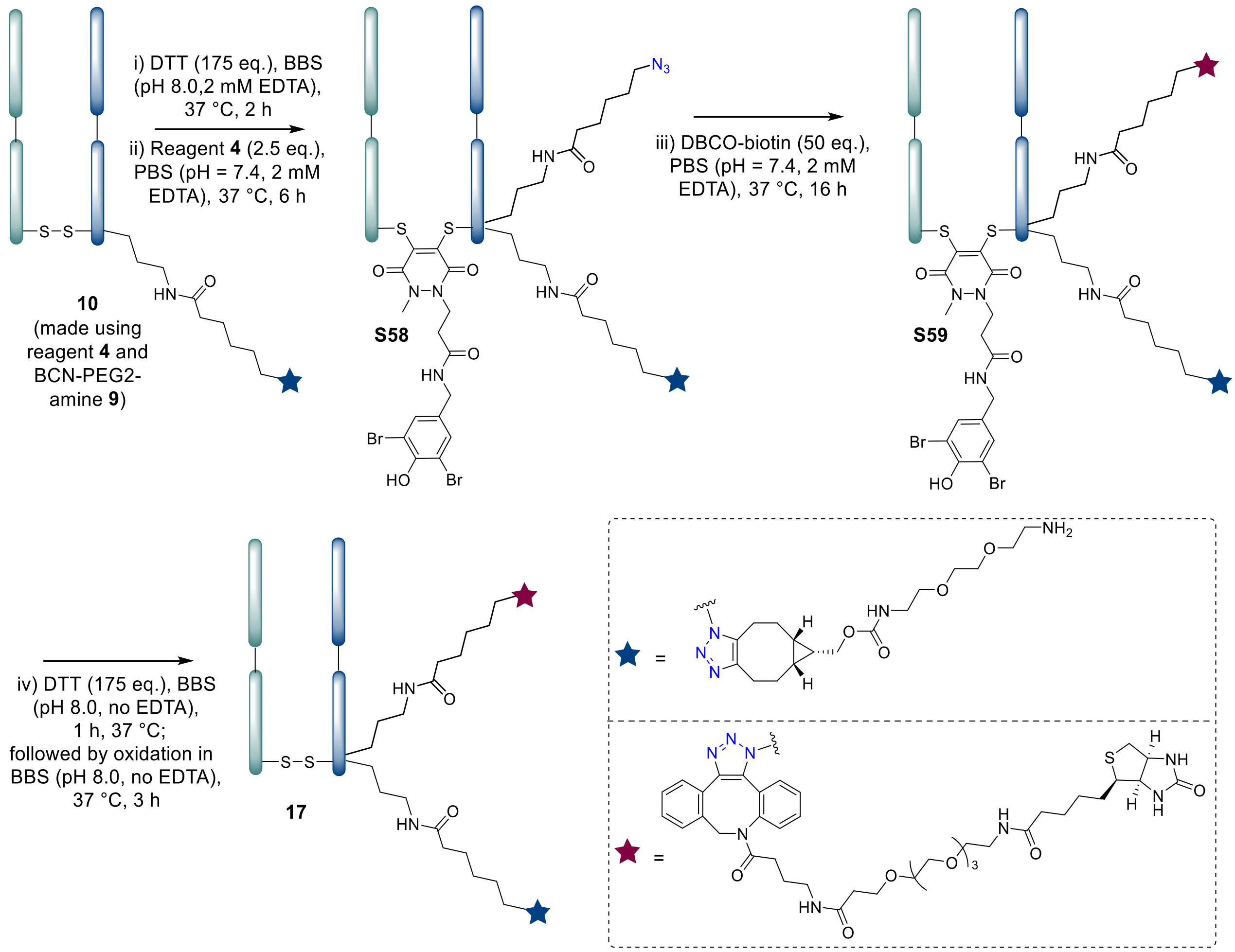

Application of the optimised protocol for mono-labelling and clicking of a Fab (see Figure 6 in the manuscript for details) on Ontruzant Fab **1** in two cycles using BCN-Amine **9** (10 mM in DMSO, 10 eq.) (in cycle 1) (ESI 2.7.2) and DBCO-biotin (in cycle 2) as the strained alkyne components and bypassing the disulfide restoration step at the end of cycle 1 to afford dually-labelled Fab conjugate **17**.

a) Conjugation step (ii)

Conjugate **S58**: Expected mass: 48700.93 Da, observed mass: 48700.39 Da

Conjugate **S33**: 48237.34 Da, observed mass: 48237.24 Da

Conjugate **S40**: Expected mass: 48561.76 Da, observed mass: 48562.62 Da

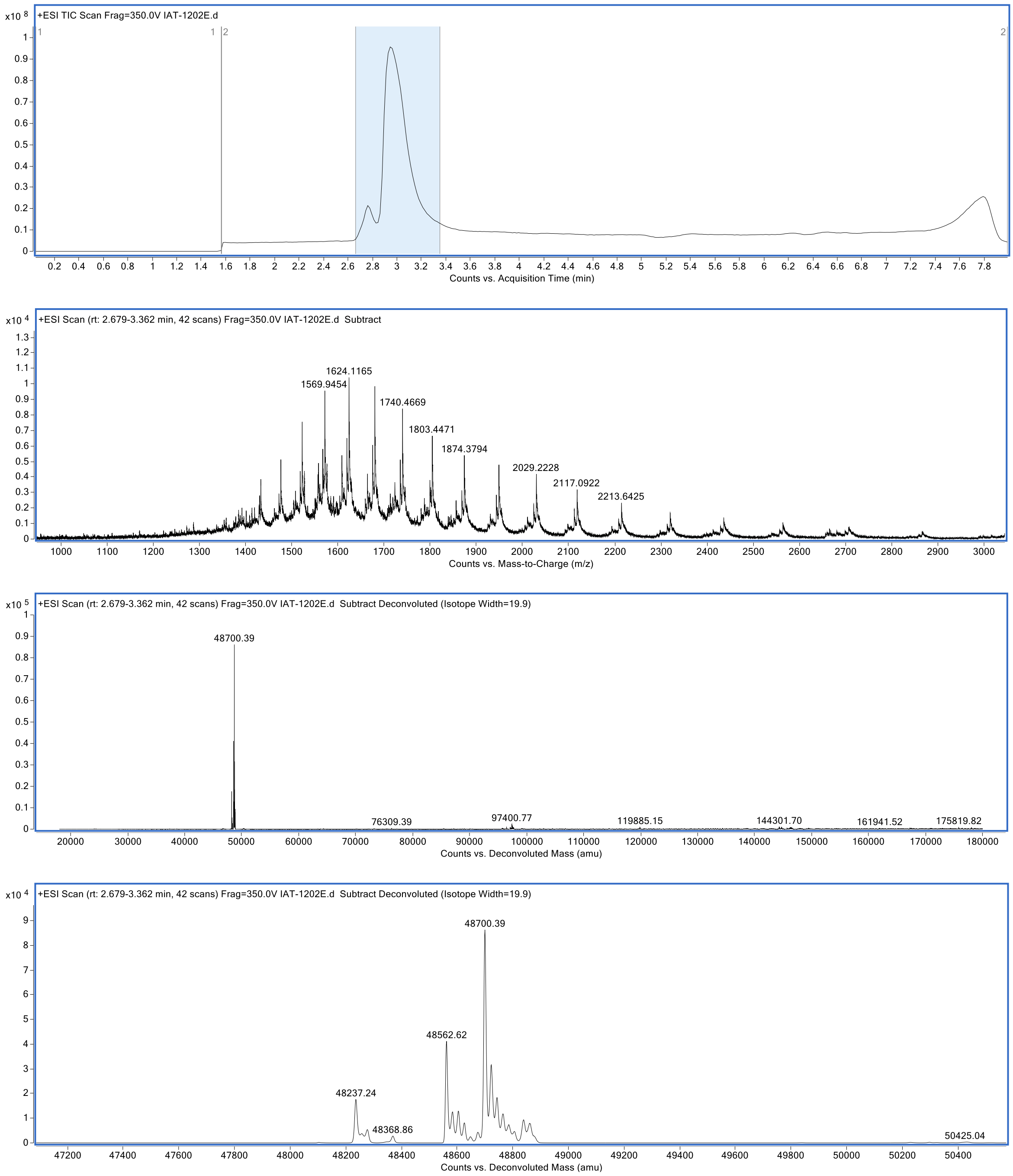

Figure S105: (i) TIC LC-MS trace (top), (ii) non-deconvoluted LC-MS trace (upper middle), (iii) deconvoluted MS data (lower middle, wide range), (iv) bottom (zoom in mass range) for conjugation step.

c) Click step (iii) with DBCO-biotin

Conjugate **S59**: Expected mass: 49450.85 Da, observed mass: 49451.35 Da

Conjugate **S38**: Expected mass: 48561.76 Da, observed mass: 48563.51 Da

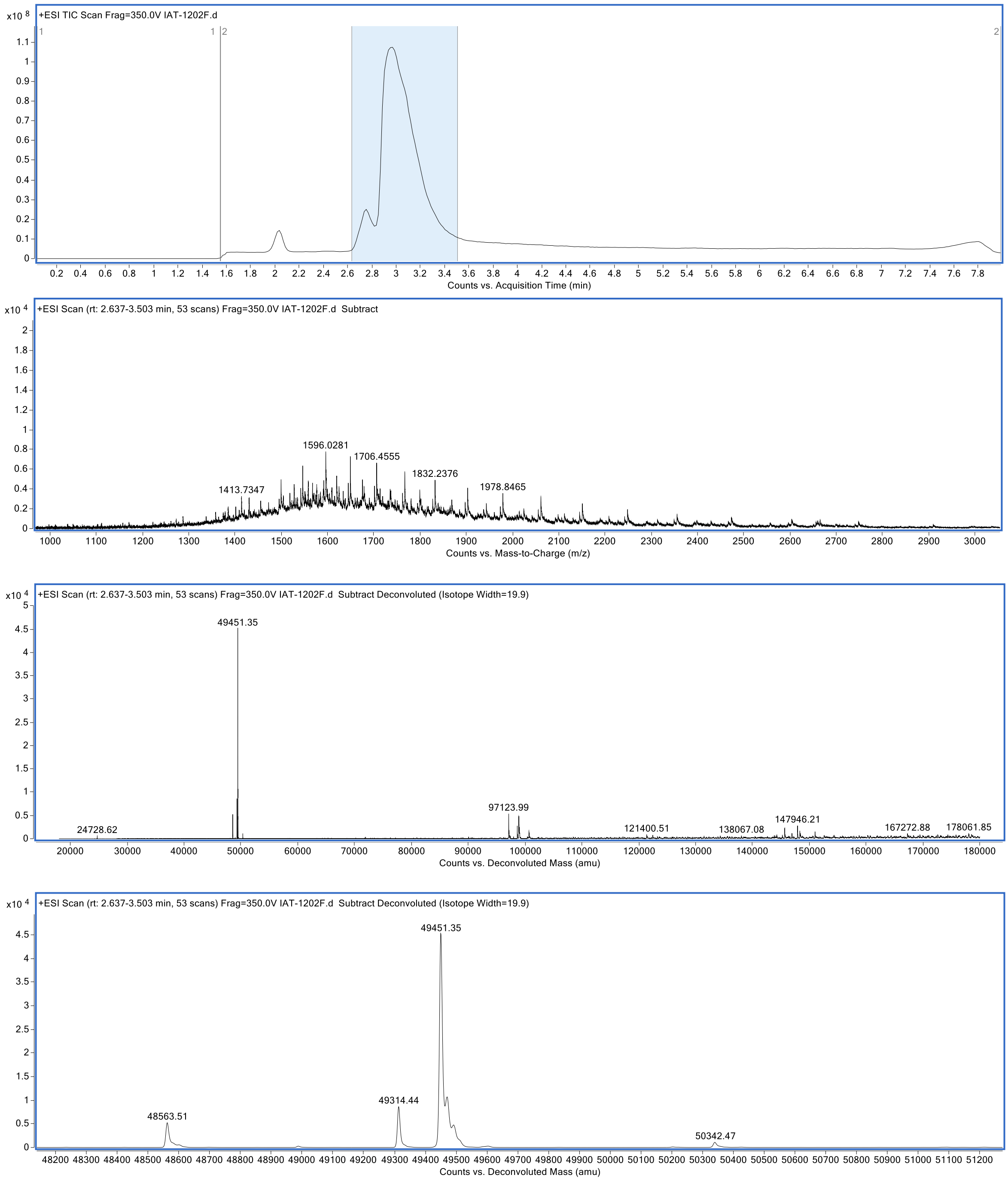

Figure S106: (i) TIC LC-MS trace (top), (ii) non-deconvoluted LC-MS trace (upper middle), (iii) deconvoluted MS data (lower middle, wide range), (iv) bottom (zoom in mass range) for click step. Masses in the ca. 97,000-99,000 Da region are spurious mass spectrometry artefacts that are the double masses of the conjugates observed in the Fab region; these higher mass species are not observed by SDS-PAGE.

d) Restoration step

Conjugate **17**: Expected mass: 48993.78 Da, observed mass 48994.51 Da

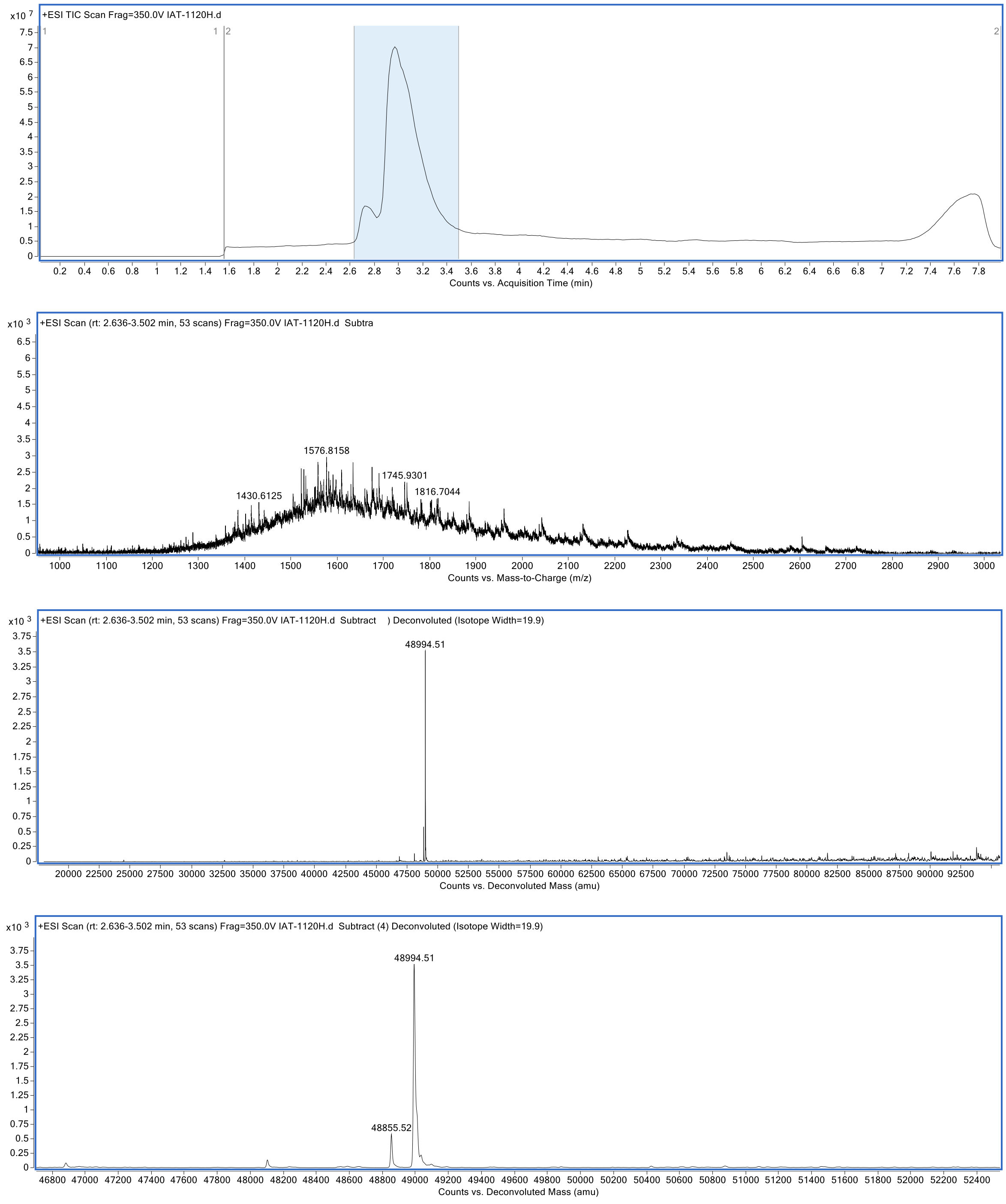

Figure S107: (i) TIC LC-MS trace (top), (ii) non-deconvoluted LC-MS trace (upper middle), (iii) deconvoluted MS data (lower middle, wide range), (iv) bottom (zoom in mass range) for restoration step.

4.0 Stability study on reagent **4**

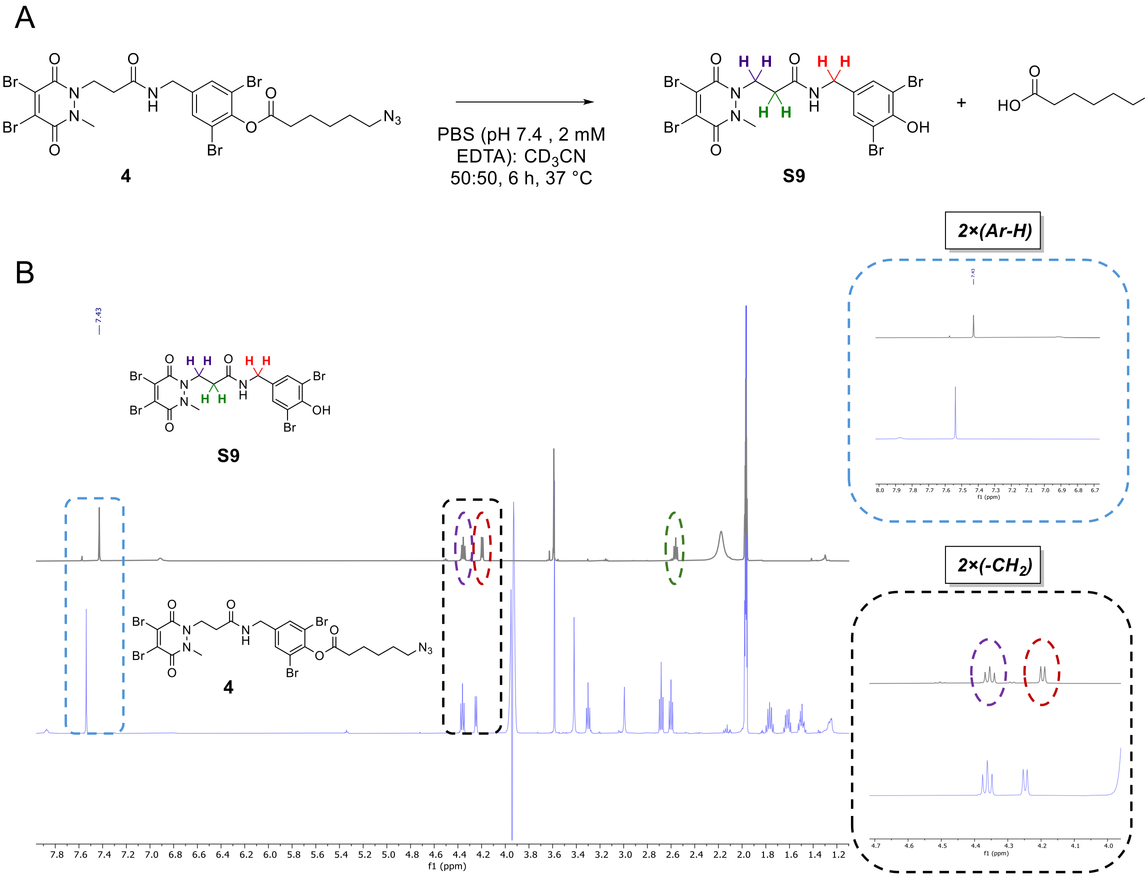

Figure S108: Stability study of reagent **4** via NMR spectroscopy. A) Reaction scheme of reagent **4** (5 mM final solution) incubated in PBS (pH 7.4, 2 mM EDTA):CD_3CN (50:50), 6 h, 37 °C. B) Stacked NMR spectra of reagent **S9** and reagent **4** at the t = 6 h timepoint of interest.

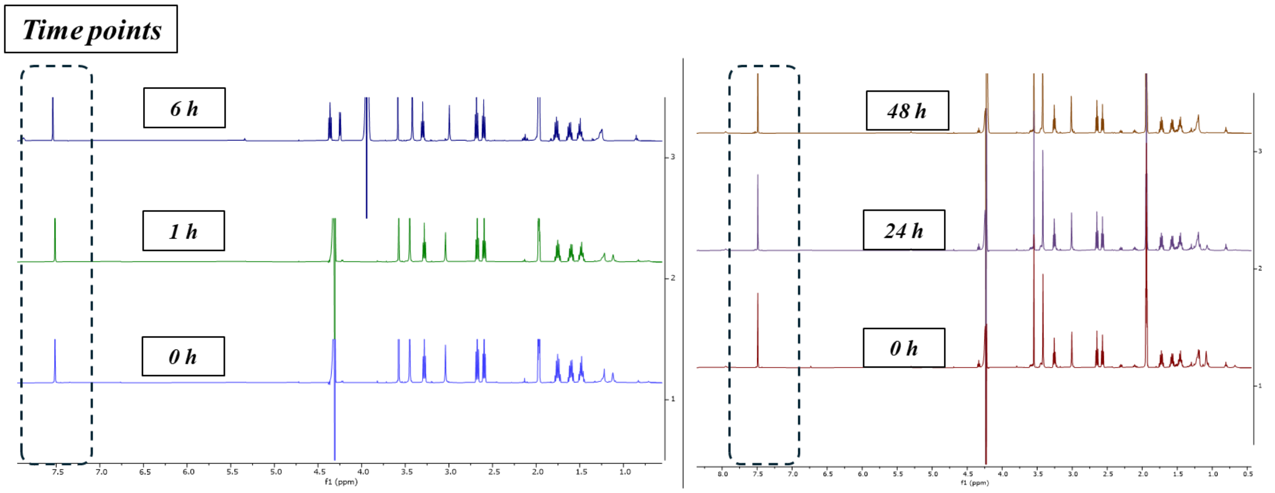

Figure S109: Stability study on reagent **4** via NMR spectroscopy at timepoints collected at t = 0, 1, 6, 24 and 48 h.

4.1 Chymotrypsin digestion of conjugate **10** (made using reagents **4**, **7** and **8** respectively)

In three separate reactions, conjugate **10** was buffer exchanged into H_2O (10 kDa MWCO). The concentration was determined by UV/Vis absorbance and adjusted to 450 μM (21.4 mg/mL). 12 μL of this solution were diluted to 60 μL with a solution of 6M Guanidine·HCl and 2 mM EDTA. DTT (2.6 μL, 100 mM solution in 100 mM Tris buffer, pH 8.0) was then added. After 60 min at 37 °C, iodoacetamide (5.2 μL, 100 mM solution in 100 mM Tris buffer, pH 8.0) was added and the mixture was incubated for a further 75 min at 37 °C, in the dark. The reaction was diluted with 240 μL H2O and 60 μL Tris buffer (50 mM, pH 8.0), before chymotrypsin (Promega Chymotrypsin, sequencing grade, 2.6 μL, 1 mg/mL solution in 1 mM HCl) was added. The resultant mixture was incubated for 14 h at 22 °C with shaking (300 rpm). After this period, the reaction was centrifuged and stopped by the addition of TFA (1.9 μL). The peptide mixture was then purified using a C18 cartridge (Sep-Pak C18, 360 mg sorbent); the sample was first washed with 1.5 mL of H_2O, 0.1% formic acid solution and eluted with 3.0 mL of 30% H_2O, 70% MeCN, 0.1% formic acid solution. It was then concentrated using a Speedvac concentrator and analyzed by LC-MS and LC-MS/MS.

4.1.1 LC-MS/MS analysis – Peptide mapping

The LC-MS/MS system consisted of a Q Exactive™ Plus mass spectrometer equipped with a HESI probe coupled to a Vanquisher UHPLC system (Thermo Fisher Scientific, UK). Chromatographic separation of peptides was achieved on a Hypersil Gold C18 (100 mm × 2.1 mm, 1.9 μm particles, P/N 25002-101130) column from Thermo Fisher Scientific, UK. Analysis was performed using a binary gradient of 0.1% (v/v) formic acid in water (A) and 0.1% (v/v) formic acid in acetonitrile (B). Gradient conditions were as follows: %B was held at 2% for 2 min. %B was then increased linearly to 40% over 48 min, then to 95% in 0.1 min. %B was held at 95% for a further 4.9 min. %B was decreased to 2% B in 0.1 min and the column was finally re-equilibrated for 4.9 min at 2% B prior to the next injection. The LC-MS/MS analysis time was 60 min. The flow rate was 0.2 mL/min. The column temperature was maintained at 30 °C throughout.

The HESI source was operated as follows: spray voltage was 3.8 kV, sheath gas flow rate was 25 AU, auxiliary gas flow rate was 5 AU, capillary temperature was 270 °C, Auxiliary gas temperature was 300 °C and S-lens RF voltage set to 55, and operated in positive ionisation mode. A Full MS/dd-MS2 (Top5) HCD method was set for the identification. Profile data was collected in the full MS scan and data dependent MS/MS modes. A resolution setting of 70,000 at m/z 200 was used for full MS scan. Mass range was set to 290–1800 m/z and AGC target value of 1.0 × 106 with a maximum injection time of 50 ms and one microscan. In-source CID was set to 0 eV. MS2 settings were as

follows: top 5 precursors selected for fragmentation, a resolution setting of 17,500 (at m/z 200), AGC target value of 1.0 × 105, a maximum injection time of 250 ms, isolation window set to 1.6 m/z, normalised collision energy set to 22, signal intensity threshold of 2.0 × 104, and dynamic exclusion set to 10s.

Peptide identification was performed using Thermo Scientific™ BioPharma Finder™ software version 5.2, according to parameters summarised in the Table below, and verified manually. Payload modification (BCN) was set up as variable modification on lysine residue side chain (Monoisotopic mass = +463.2795, Average mass +463.37)

Analysis was conducted using Biopharma software

**Protease, Protein Modifications**	
Protease Name	Chymotrypsin
N-Term	
C-Term	WYFL
Specificity	High
Static Modifications	Carbamidomethylation(SideChain,C,x10)
Variable Modifications	Deamidation (N)(SideChain), Deamidation (Q)(SideChain), BCN(SideChain), Oxidation, (MW)(SideChain)
**Component Detection Parameters**	
S/N Threshold	11
Typical Chromatographic Peak Width (min)	0.3
Time Limits	0.5 to 55
Relative MS Signal Threshold (% of base peak)	1
Relative Analog Threshold (% of highest peak)	1
Width of Gaussian Filter (represented as 1/n of chromatographic peak width)	3
Minimum Valley to be Considered as Two Chromatographic Peaks (%)	80%
Minimum MS Peak Width (Da)	1.2
Maximum MS Peak Width (Da)	4.2
Mass Tolerance (ppm for high-res or Da for low-res)	4
Maximum Retention Time Shift (min)	1.67
Maximum Mass (Da)	30000
Mass Centroiding Cutoff (% from base)	15
**Identification Parameters**	
Maximum Peptide Mass	11000
Mass Accuracy (ppm)	5
Minimum Confidence	0.8
Maximum Number of Modifications for a Peptide	5
Mass Changes for Unspecified Modifications	-129 to 169
Glycosylation	None
Perform Disulfide Bond Search	No

4.2 Sequence coverage maps of conjugate **10** (made using reagents **4**, **7** and **8** respectively)

4.2.1 Results on conjugate **10** (made using reagent **4**)

Sequence Coverage Map

Created on 06/21/24
Minimum MS Signal = 1.0054e+06
Data File = UCL_IAT-1054_Chymotrypsin_Hypersil_dda_method5_47Grad_10ul_050624.raw
Protease = Chymotrypsin

Proteins	Number of MS Peaks	MS Peak Area	Sequence Coverage	Abundance (mol)
1:Fab_LC	952	36.7%	100.0%	55.12%
2:Fab_HC	1040	31.5%	100.0%	44.88%
Unidentified	3594	31.8%		

Minimum Recovery = 1%
Minimum Recovery of Overlapping Peptides = 0%
Minimum Confidence = 0.8
Maximum Mass = 11000

Color code for peptide recovery

>50.0%	>20.0%	>10.0%	>5.0%	>2.0%	>1.0%	>0.5%	>0.2%	>0.1%	>0.0%
good		fair			low		poor		

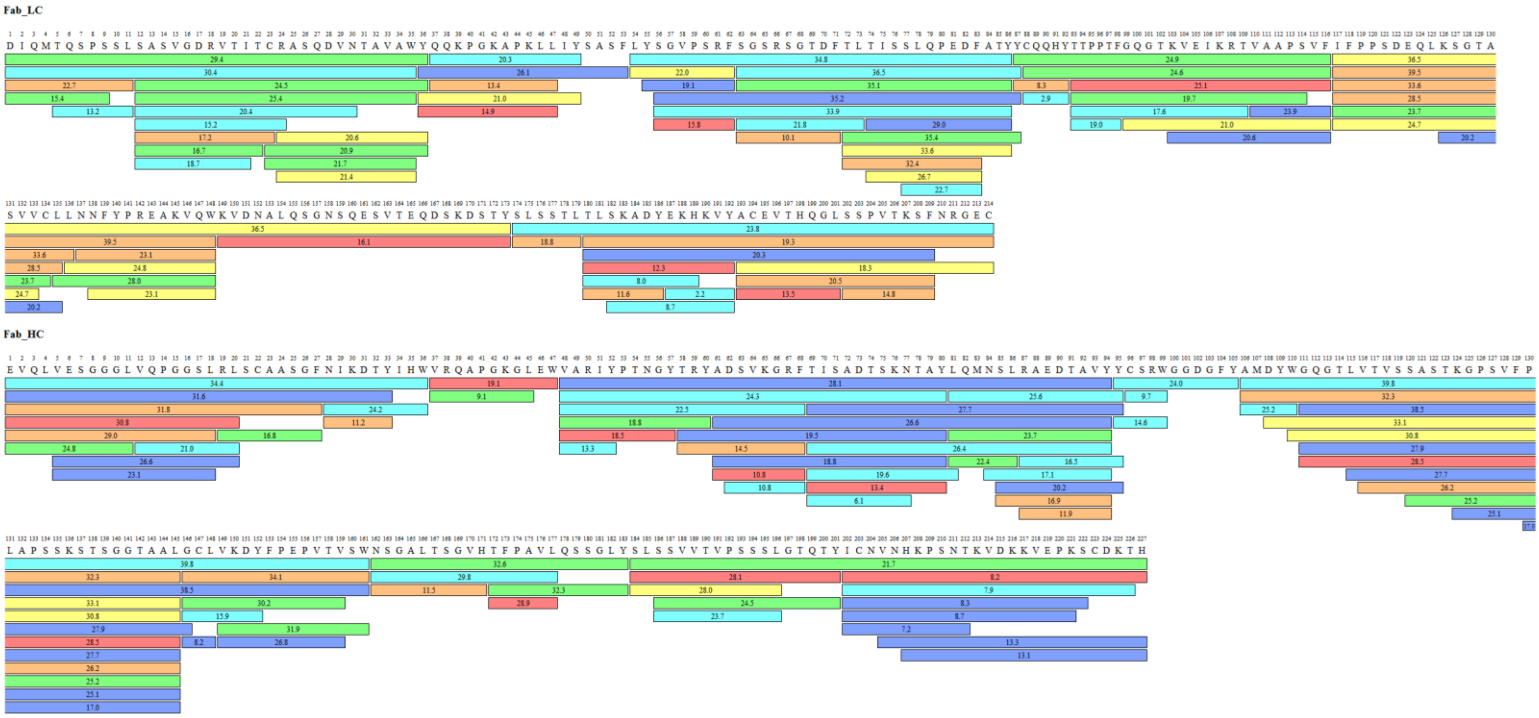

4.2.2 Results on conjugate **10** (made using reagent **7**)

Sequence Coverage Map

Created on 06/21/24
Minimum MS Signal = 1.0054e+06
Data File = UCL_IAT-1046_Chymotrypsin_Hypersil_dda_method5_47Grad_10ul_050624.raw
Protease = Chymotrypsin

Proteins	Number of MS Peaks	MS Peak Area	Sequence Coverage	Abundance (mol)
1:Fab_LC	821	35.4%	94.9%	53.52%
2:Fab_HC	924	33.8%	93.4%	46.48%
Unidentified	3325	30.8%		

Minimum Recovery = 1%
Minimum Recovery of Overlapping Peptides = 0%
Minimum Confidence = 0.8
Maximum Mass = 11000

Color code for peptide recovery

>50.0%	>20.0%	>10.0%	>5.0%	>2.0%	>1.0%	>0.5%	>0.2%	>0.1%	>0.0%
good			fair			low		poor	

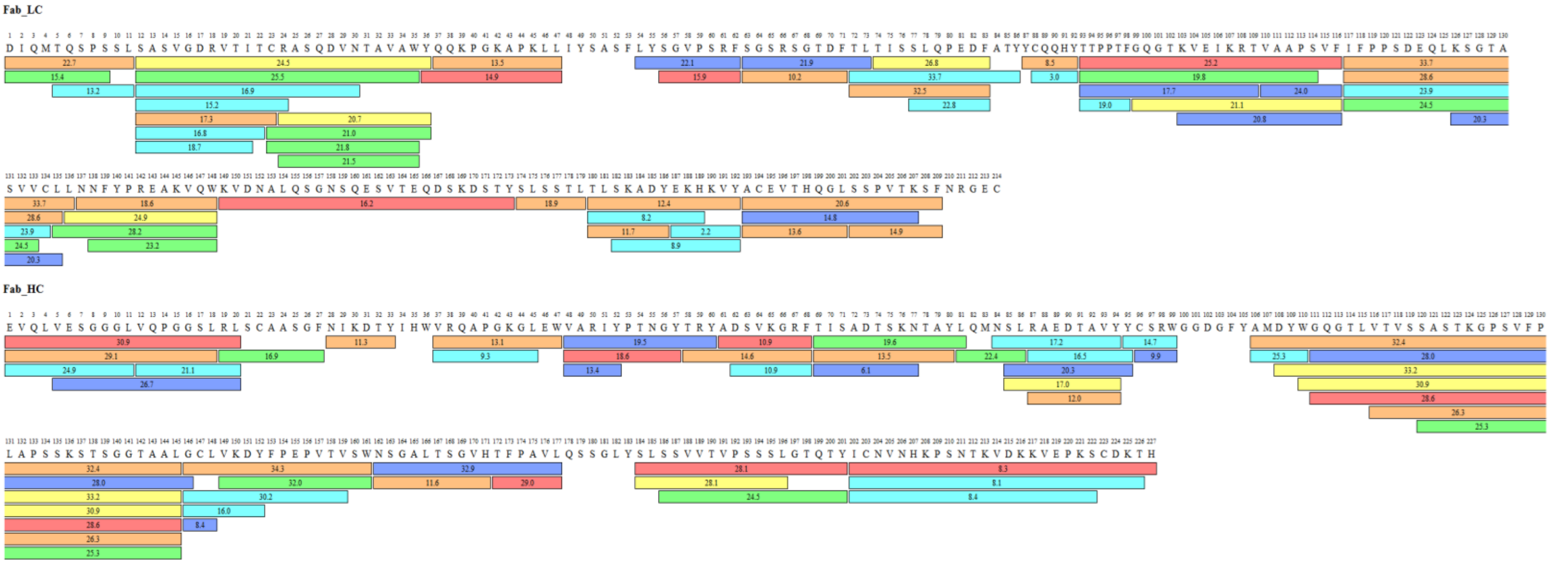

4.2.3 Results on conjugate **10** (made using reagent **8**)

Sequence Coverage Map

Created on 06/21/24
Minimum MS Signal = 1.0054e+06
Data File = UCL_IAT-1047_Chymotrypsin_Hypersil_dda_method5_47Grad_10ul_050624.raw
Protease = Chymotrypsin

Proteins	Number of MS Peaks	MS Peak Area	Sequence Coverage	Abundance (mol)
1:Fab_LC	906	35.5%	98.1%	55.42%
2:Fab_HC	972	29.0%	98.7%	44.58%
Unidentified	3674	35.5%		

Minimum Recovery = 1%
Minimum Recovery of Overlapping Peptides = 0%
Minimum Confidence = 0.8
Maximum Mass = 11000

Color code for peptide recovery

>50.0%	>20.0%	>10.0%	>5.0%	>2.0%	>1.0%	>0.5%	>0.2%	>0.1%	>0.0%
good			fair			low		poor	

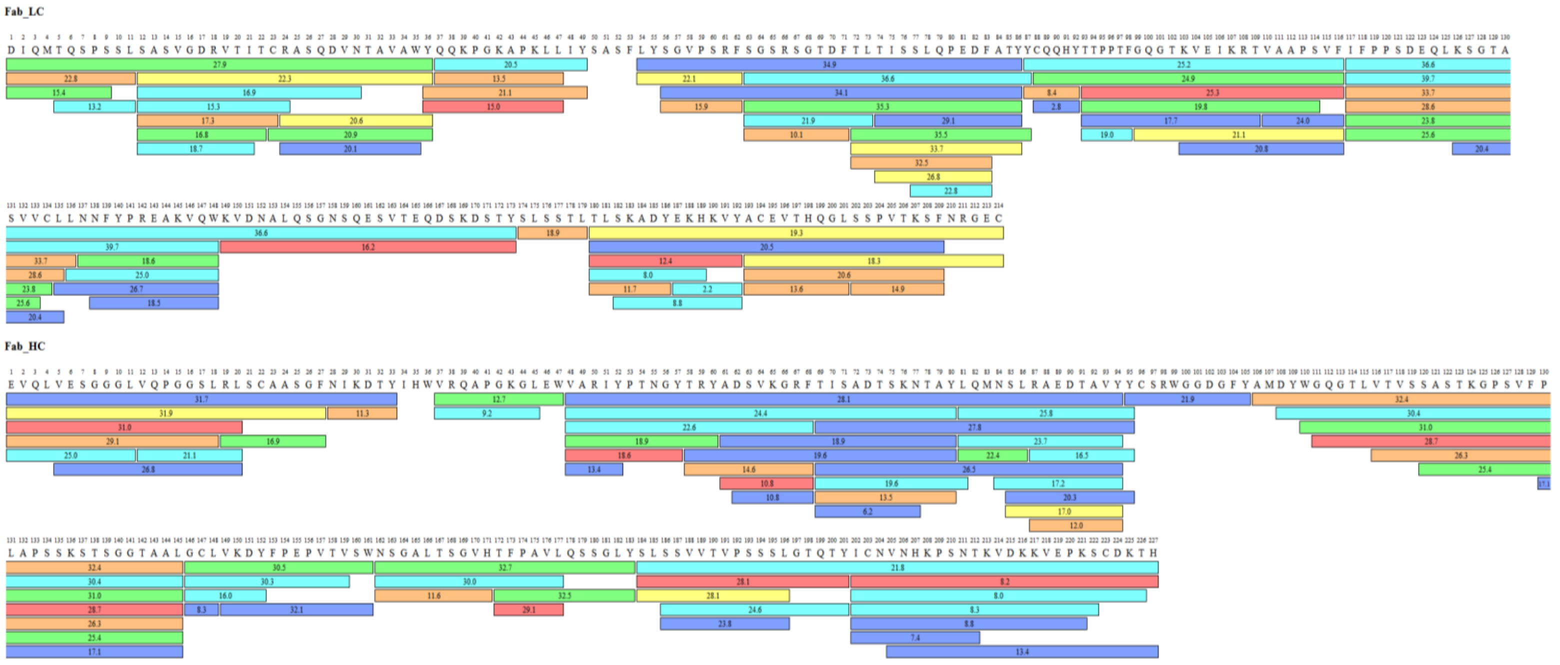

4.3 Sites of modification

- K126 modification

Light chain: IFPPSDEQLK^{126}SGTASVVC^{134}L (BCN-K126, carbamidomethylation C134)

Conjugate **10** (using reagent **4**)

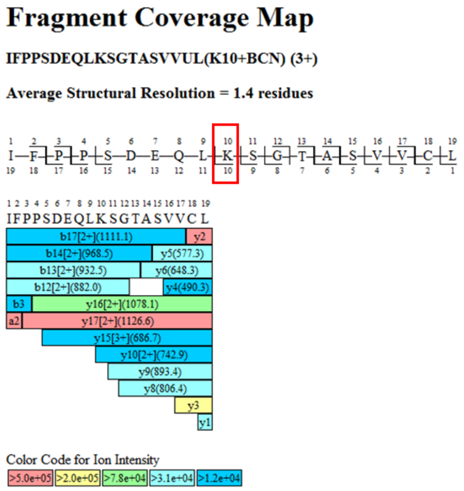

Conjugate **10** (using reagent **7**)

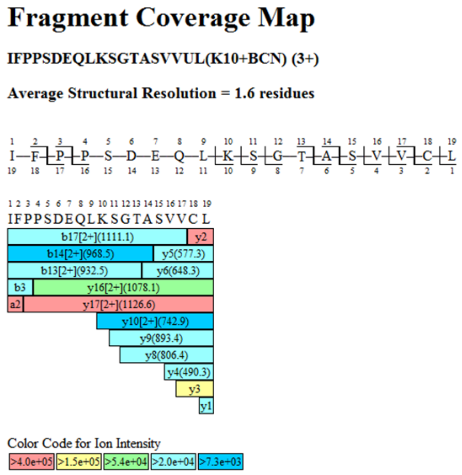

Conjugate **10** (using reagent **8**)

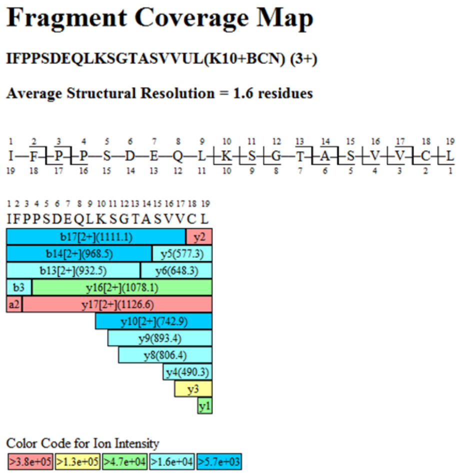

- K183 modification

Light chain: TLSK^{183}ADYEK^{188}HK^{190}VY (K183-BCN)

Conjugate **10** (using reagent **4**)

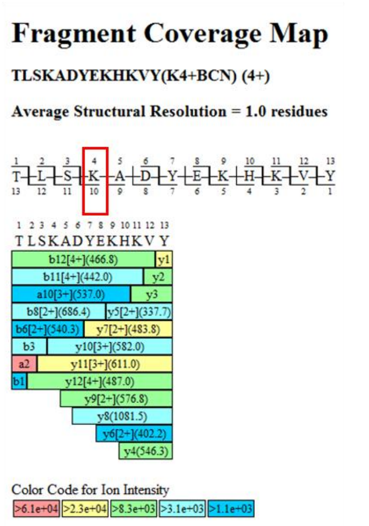

Conjugate **10** (using reagent **7**)

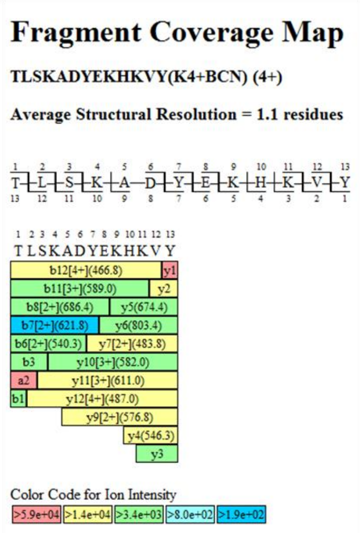

Conjugate **10** (using reagent **8**)

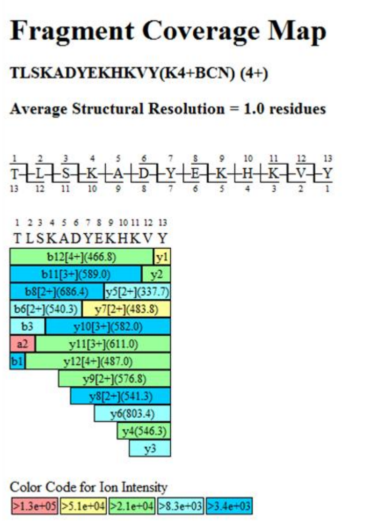

- K190 modification

Light chain: TLSK^{183}ADYEK^{188}HK^{190}VY (K190-BCN)

Conjugate **10** (using reagent **4**)

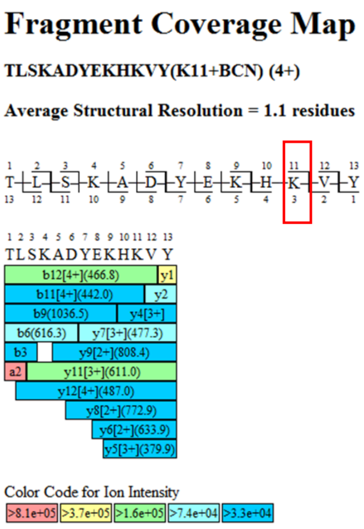

Conjugate **10** (using reagent **7**)

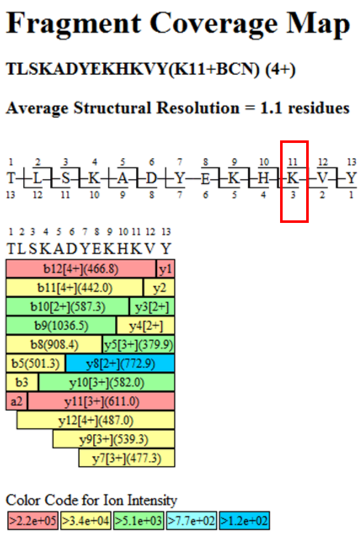

Conjugate **10** (using reagent **8**)

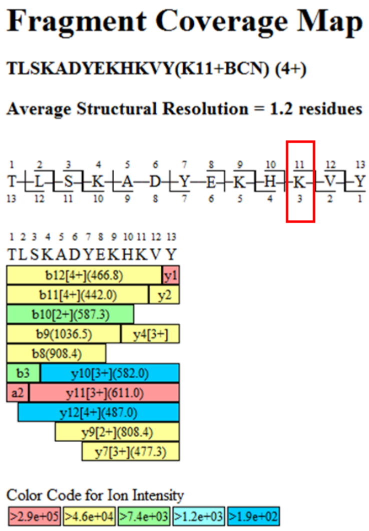

- K207 modification

ACEVTHQGLSSPVTK^{207}SF (K207-BCN, Carbamidomethylation-C194)

Conjugate **10** (using reagent **4**)

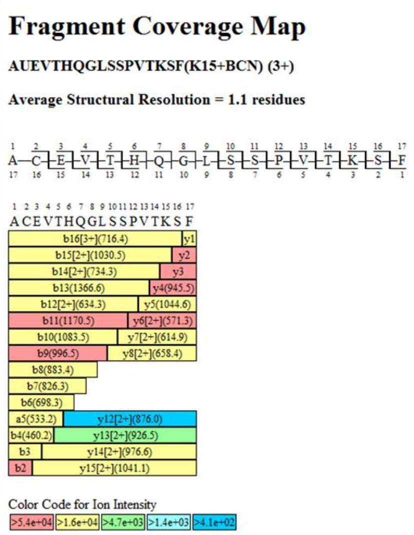

Conjugate **10** (using reagent **7**)

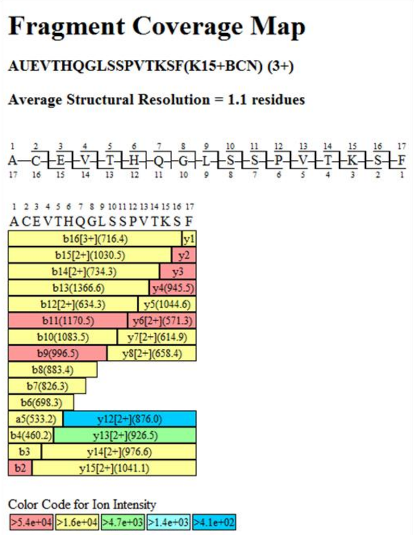

Conjugate **10** (using reagent **8**)

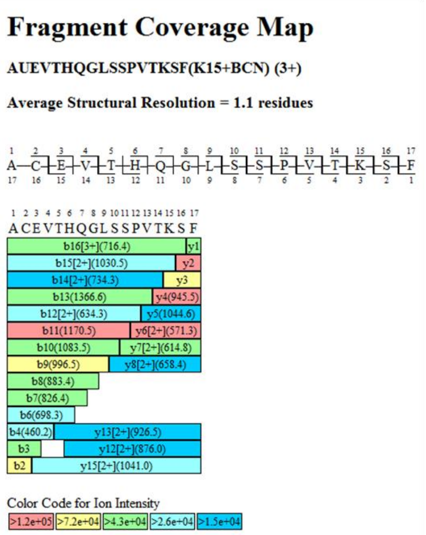

- K136 modification

Heavy chain: PLAPSSK^{136}STSGGTAAL (K136-BCN modified peptide)

Conjugate **10** (using reagent **4**)

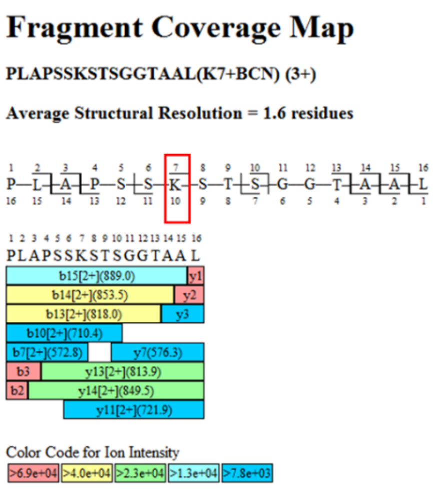

Conjugate **10** (using reagent **7**)

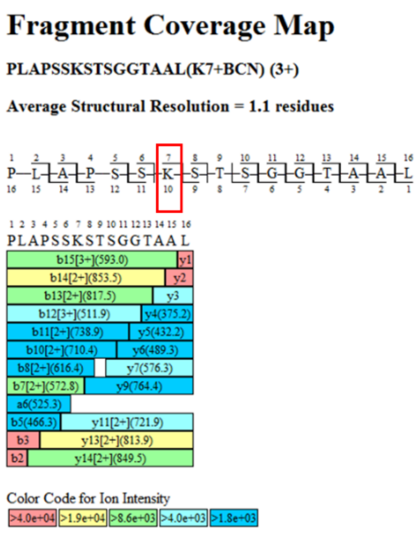

Conjugate **10** (using reagent **8**)

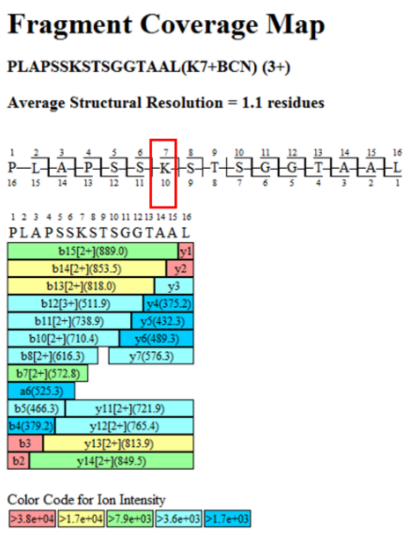

- K221 modification

IC^{203}NVNHK^{208}PSNTK^{213}VDK^{216}K^{217}VEPK^{221}SC^{223}DK^{225}TH (K221-BCN, carbamidometylation on C203 and C223)

Conjugate **10** (using reagent **4**)

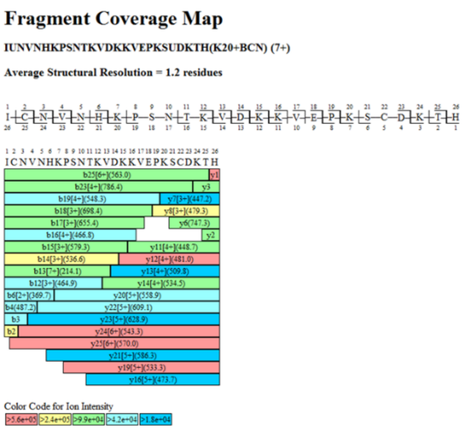

Conjugate **10** (using reagent **7**)

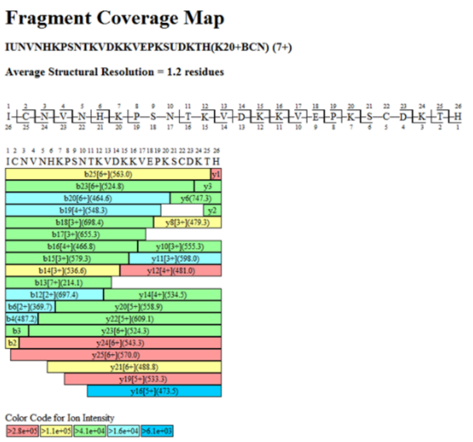

Conjugate **10** (using reagent **8**)

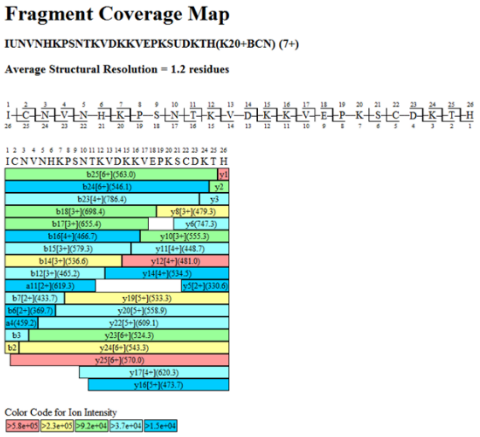

- K225 modification

IC^{203}NVNHK^{208}PSNTK^{213}VDK^{216}K^{217}VEPK^{221}SC^{223}DK^{225}TH (BCN-K225, carbamidomethylation on C203 and C223)

Conjugate **10** (using reagent **4**) | Conjugate **10** (using reagent **7**) | Conjugate **10** (using reagent **8**)

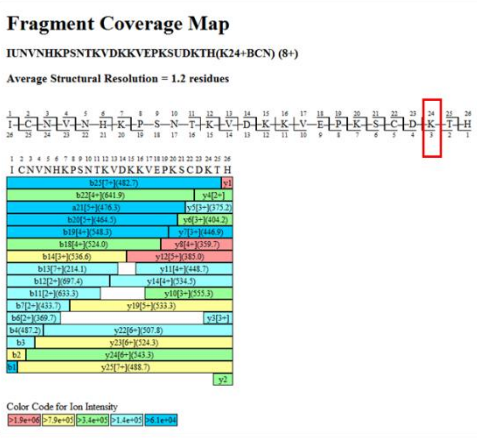

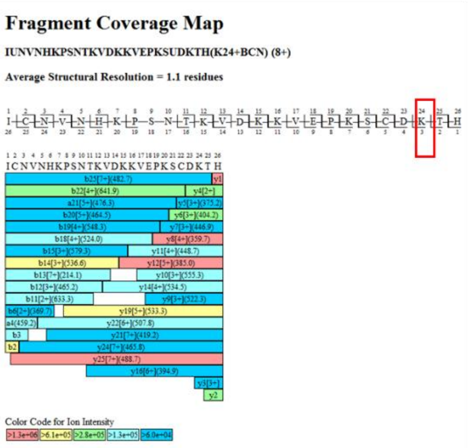

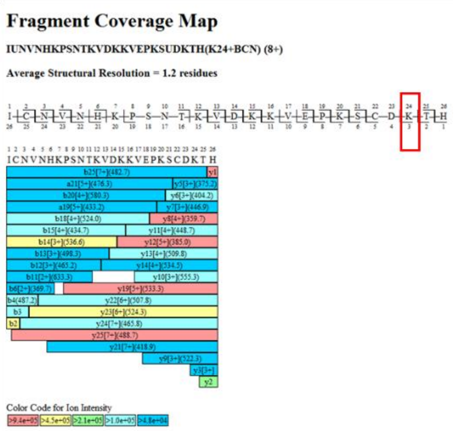

Summary of modifications according to BiopharmaFinder software

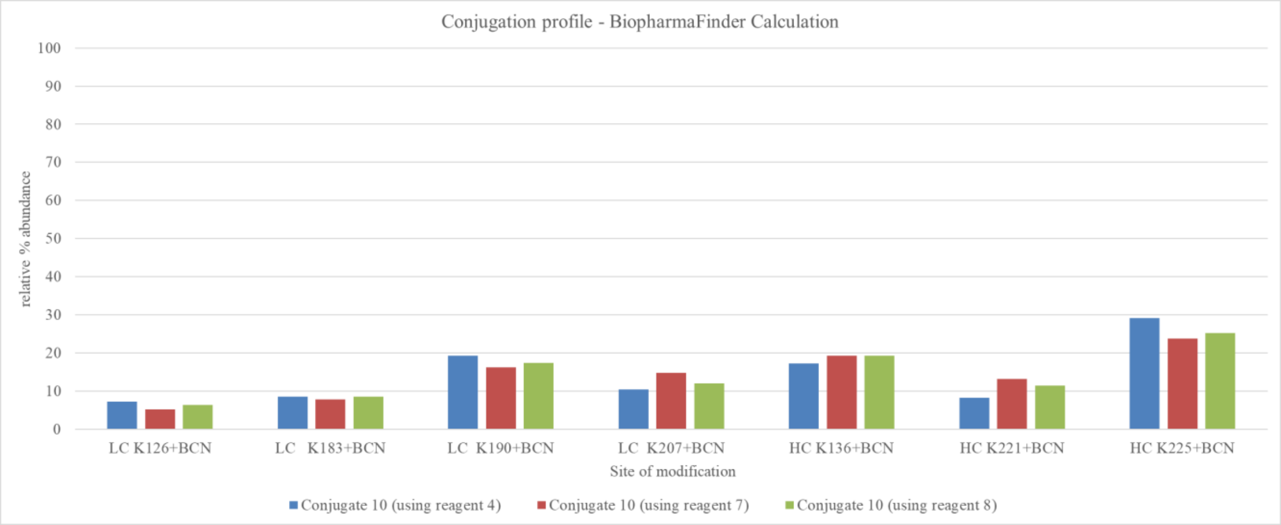

4.3 Enzyme-Linked Immunosorbent Assay (ELISA)

A 96-well plate was coated for 1 h at RT with HER2 (Sino Biological, 100 µL/well, 0.25 µg/mL solution in PBS). After washing (3 × 0.1% Tween® 20 in PBS, followed by 3 × PBS), the wells were blocked for 1 h at RT with 5% Marvel milk powder (Premier foods) in PBS (200 µL/well). The wells were then washed and the following dilutions of native Fab and conjugate **10** (made using reagents **4**, **7** and **8** respectively) were applied: 270 nM, 90 nM, 30 nM, 10 nM, 3.33 nM, 1.11 nM, 0.37 nM, 0.123 nM, 0.0412 nM, 0.0137 nM, prepared in 1% Marvel solution in 0.1% Tween® 20 in PBS (100 µL/well). The assay was then incubated at RT for 1 h, washed and the detection antibody (Anti-Human IgG, Fab specific-HRP antibody, Sigma Aldrich, 1:5000 in 1% Marvel solution in 0.1% Tween® 20 in PBS) was added (100 µL/well). After 1 h at RT, the plates were washed and *o*-phenylenediamine dihydrochloride (Sigma-Aldrich, 100 µL/well, 0.5 mg/mL in a phosphate-citrate buffer with sodium perborate) was added. Once a yellow-orange colour was observed, the reaction was stopped by addition of HCl (4M, 50 µL/well). Absorbance was immediately measured at 450 nm and was corrected by subtracting the average of negative controls (i.e. PBS had been added to some of the wells instead of HER2 or instead of the samples). Each sample was tested in triplicate and errors are shown as the standard deviation of the average. ELISA data were analysed with Graphpad Prism 10.1.24 and the values have been normalized.

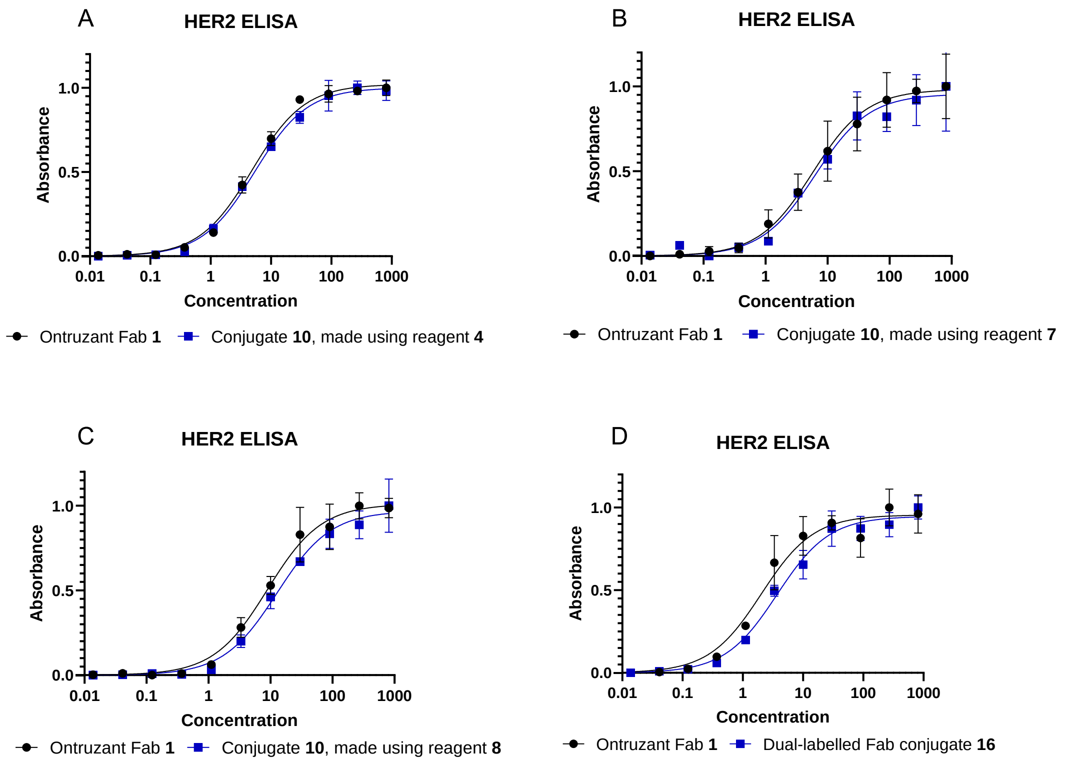

Figure S110: ELISA analysis of A) Conjugate **10**, made using reagent **4** and Ontruzant Fab **1**, B) Conjugate **10**, made using reagent **7** and Ontruzant Fab **1**, C) Conjugate **10**, made using reagent **8** and Ontruzant Fab **1** against HER2.

4.4 Reaction of Ontruzant mAb **S23** with reagent **4**

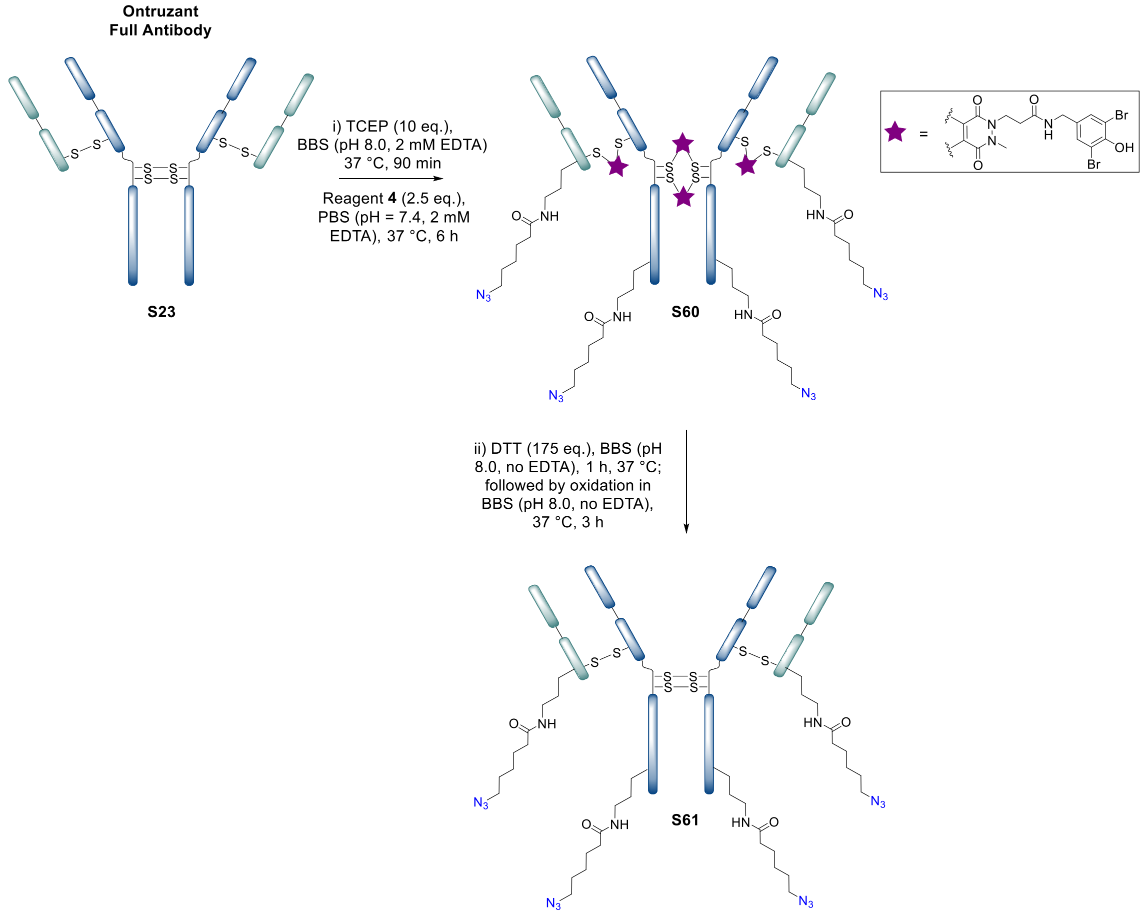

To a solution of Ontruzant mAb **S23** (130 µL, 20 µM) in BBS (25 mM sodium borate, 25 mM NaCl, 2 mM EDTA, pH 8.0) was added TCEP·HCl (20 mM in DI H2O, 10 eq.) The reaction was incubated at 37 °C for 90 min whilst shaking (300 rpm). Upon completion, the conjugate was purified into PBS (50 mM phosphate, 150 mM NaCl, 2 mM EDTA, pH 7.4) by centrifugation (7k Zeba Spin desalting column) and reagent **4** (10 mM in MeCN, 10 eq.) was added, and incubated at 37 °C for 6 h whilst shaking (300 rpm). After this time, the conjugate was purified into BBS (25 mM sodium borate, 25 mM NaCl, no EDTA, pH 8.0), DTT (50 mM in DI H2O, 500 eq.) was added, and the reaction was incubated at 37 °C for 1 h. Upon completion, the conjugate was purified into BBS (25 mM sodium borate, 25 mM NaCl, no EDTA, pH 8.0) at 37 °C for 3 h to allow disulfide restoration. After this time, the conjugate was purified into DI H_2O. The resulting conjugate was analysed by LC-MS.

a) Conjugation step

Ontruzant mAb **S23**: Expected mass: 145176 Da, observed mass: not observed

Conjugate **S60**: Expected mass: 147564.96 Da, observed mass: 147567.52 Da

Whilst the expected mass was observed, we observed a large number of other masses associated with side-reactions.

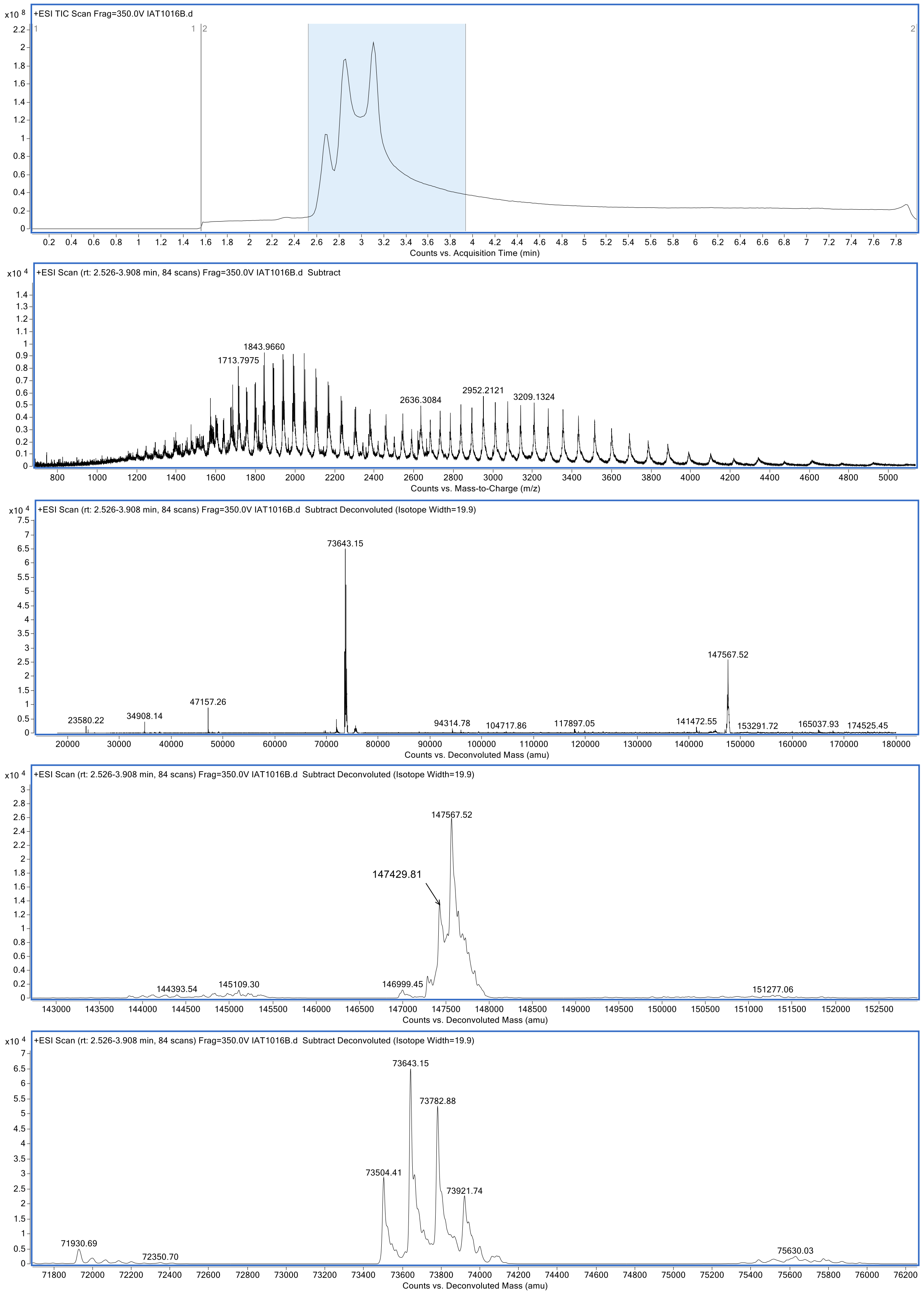

Figure S111: (i) TIC LC-MS trace (top), (ii) non-deconvoluted LC-MS trace (upper middle), (iii) deconvoluted MS data (lower middle, wide range), (iv) zoom in mass range in Fab region (upper bottom), (v) zoom in mass range in half-antibody region (bottom) for conjugation step.

b) Restoration step

Conjugate **S61**: Expected mass: 145736.00 Da, observed mass: 145730.16 Da

Whilst the expected mass was observed, we observed a large number of other masses associated with side-reactions. We have tried to rationalise these below:

Full mAb + 3 lysine modifications: Expected mass: 145594.00 Da, observed mass: 145610.95 Da

Full mAb + 2 lysine modifications: Expected mass: 145454.00 Da, observed mass: 145453.00 Da

Full mAb + 1 lysine modification: Expected mass: 145315.00 Da, observed mass: 145321.00 Da

Half Ontruzant mAb: Expected mass: 72594.00 Da, observed mass: 72590.00 Da

Half Ontruzant mAb + 2 lysine modifications: Expected mass: 72872.00 Da, observed mass: 72865.40 Da

Half Ontruzant mAb + 1 lysine modification: Expected mass: 72733.00 Da, observed mass: 72725.00 Da

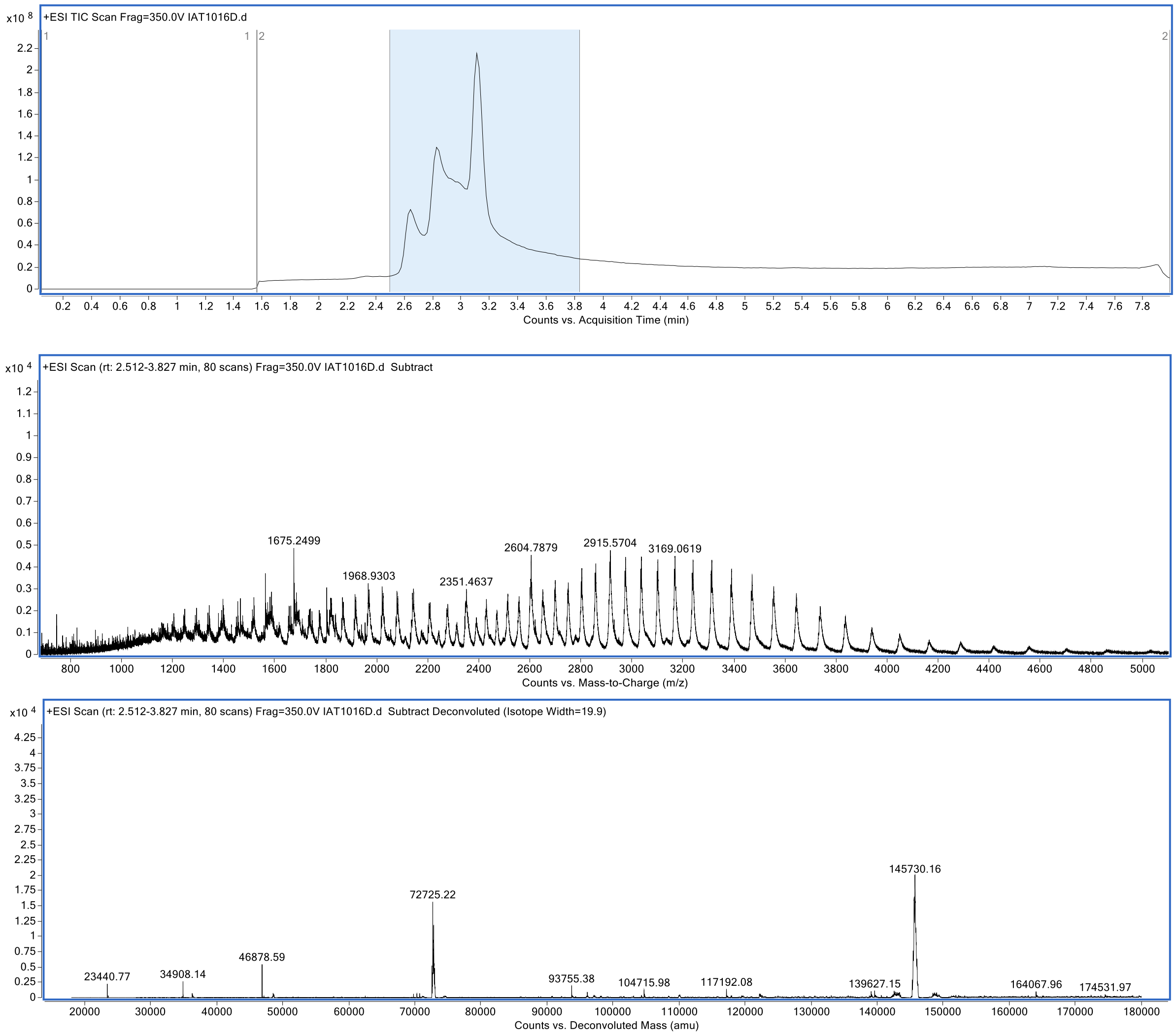

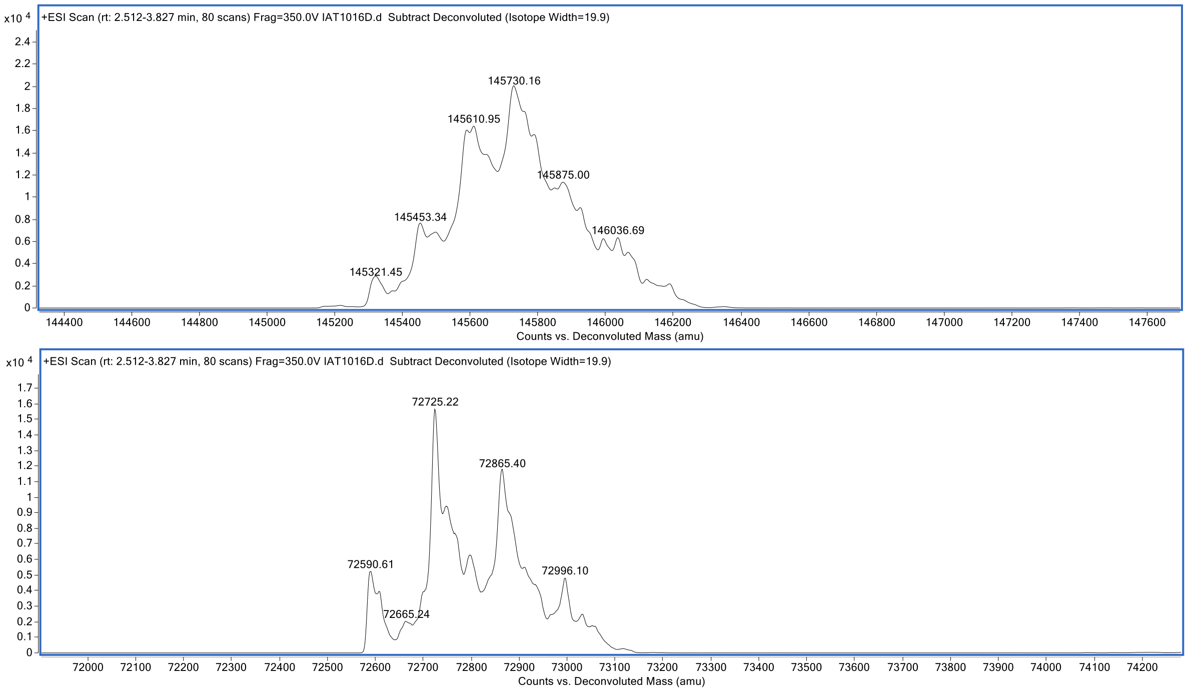

Figure S112: (i) TIC LC-MS trace (top), (ii) non-deconvoluted LC-MS trace (upper middle), (iii) deconvoluted MS data (lower middle, wide range), (iv) zoom in mass range in Fab region (upper bottom), (v) zoom in mass range in half-antibody region (bottom) for restoration step.

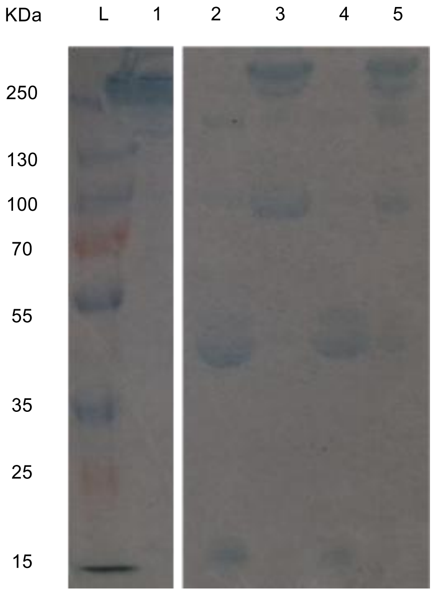

Figure S113: SDS-PAGE gel: L) Ladder, 1) Ontruzant mAb **S23**, 2) Reduction step, 3) Conjugation/Lysine reaction step, 4) Deprotection step, 5) Disulfide restoration step.

1 C. Bahou, D. A. Richards, A. Maruani, E. A. Love, F. Javaid, S. Caddick, J. R. Baker and V. Chudasama, *Org. Biomol. Chem.*, 2018, **16**, 1359–1366.

2 K. Lin, C. Hin Lam, X. Lin, J. Hsu, S. Fan, N. K. Gupta, Y. Lin, B. Khoon Tee, J. Li, J. Chen and K. Tan, *Chem. Asian J.*, 2021, **16**, 937–948.

3 B. H. Fraser, S. Hamilton, A. M. Krause-Heuer, P. J. Wright, I. Greguric, S. P. Tucker, A. G. Draffan, V. V. Fokin and K. B. Sharpless, *Med. Chem. Commun.*, 2013, **4**, 383–386.

4 X. K. Wee, W. K. Yeo, B. Zhang, V. B. C. Tan, K. M. Lim, T. E. Tay and M.-L. Go, *Bioorg. Med. Chem.*, 2009, **17**, 7562–7571.

5 Thermo Fisher Scientific, Fluorescence SpectraViewer, https://www.thermofisher.com/order/fluorescence-spectraviewer/?gclid=EAIaIQobChMIy7vr97jPiAMVN5lQBh06lAGgEAAYASAAEgILKfD_BwE&ef_id=EAIaIQobChMIy7vr97jPiAMVN5lQBh06lAGgEAAYASAAEgILKfD_BwE:G:s&s_kwcid=AL!3652!3!607635415877!e!!g!!thermo%20fisher%20spectra%20viewer!2081760689!127753028375&cid=bid_pca_aup_r01_co_cp1359_pjt0000_bid00000_0se_gaw_bt_pur_con&gad_source=1#!/, (accessed 25 August 2024).
